# An Iron‐Catalyzed Route to Dewar 1,3,5‐Triphosphabenzene and Subsequent Reactivity

**DOI:** 10.1002/anie.202208663

**Published:** 2022-08-09

**Authors:** Adam N. Barrett, Martin Diefenbach, Mary F. Mahon, Vera Krewald, Ruth L. Webster

**Affiliations:** ^1^ Department of Chemistry University of Bath Claverton Down, Bath BA2 7AY UK; ^2^ Department of Chemistry TU Darmstadt 64287 Darmstadt Germany

**Keywords:** Electrophilic Addition, Iron, Nucleophilic Addition, Phosphorus Heterocycles, Reaction Mechanisms

## Abstract

The application of an alkyne cyclotrimerization regime with an [Fe(salen)]_2_‐μ‐oxo (**1**) catalyst to triphenylmethylphosphaalkyne (**2**) yields gram‐scale quantities of 2,4,6‐tris(triphenylmethyl)‐Dewar‐1,3,5‐triphosphabenzene (**3**). Bulky lithium salt LiHMDS facilitates a rearrangement of **3** to the 1,3,5‐triphosphabenzene valence isomer (**3′**), which subsequently undergoes an intriguing phosphorus migration reaction to form the ring‐contracted species (**3′′**). Density functional theory calculations provide a plausible mechanism for this rearrangement. Given the stability of **3**, a diverse array of unprecedented transformations was investigated. We report novel crystallographically characterized products of successful nucleophilic/electrophilic addition and protonation/oxidation reactions.

## Graphical abstract

A mild, scalable iron‐catalyzed route to a Dewar phosphabenzene is presented. This species isomerizes in the presence of base to form the 1,3,5‐phosphabenzene, which then spontaneously rearranges to a novel triphospha(tricyclo) species. Onward coordination chemistry of all three phosphines is explored along with an extensive study into the reactivity of the Dewar phosphabenzene.

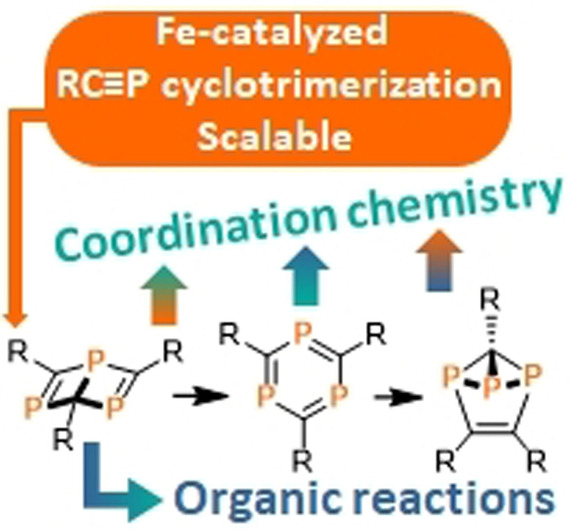

## Introduction

Phosphabenzenes are an important class of heterocycle that find widespread use in coordination chemistry, catalysis and organic transformations, and have therefore been the focus of several reviews over the past decade.^[1]^ Whilst the synthesis of mono‐phosphabenzenes is readily achieved by reaction of tris(trimethylsilyl)phosphine with pyrilium salts,^[2]^ phosphaalkynes have proven invaluable components in synthetic routes to phosphabenzenes with more than one phosphorus atom within the ring skeleton.[^1g^, ^3^] Stoichiometric, metal‐mediated reactions with phosphaalkynes dominate these pathways; early routes resulting in metal bound species^[4]^ soon paved the way for the synthesis of metal‐free 1,3,5‐triphosphabenzenes in reactions mediated by hafnium^[5]^ and vanadium.^[6]^ Grützmacher and co‐workers have recently reported the use of sodium phosphaethynolate to form a boronyl‐substituted phosphaalkyne in situ, which can then trimerize to form the corresponding 1,3,5‐triphosphabenzene without the need for a transition metal.^[7]^ These species have applications in numerous disciplines, for example the increased π‐acidity in comparison to benzenes and mono/di‐phosphabenzenes, due to the increased number of phosphorus atoms, aids 1,3,5‐triphosphabenzenes in their ability to coordinate in an η^6^ fashion to transition metals as opposed to η^1^.^[8]^ Coordination in this manner to an array of transition metal carbonyls has been reported,^[9]^ along with reactivity with organolithium reagents and transition metal complexes.^[10]^ Reactivity of the uncomplexed species extends further, with examples of ring contractions^[11]^ and 1,4‐addition reactions^[12]^ published to date.

**Scheme 1:**
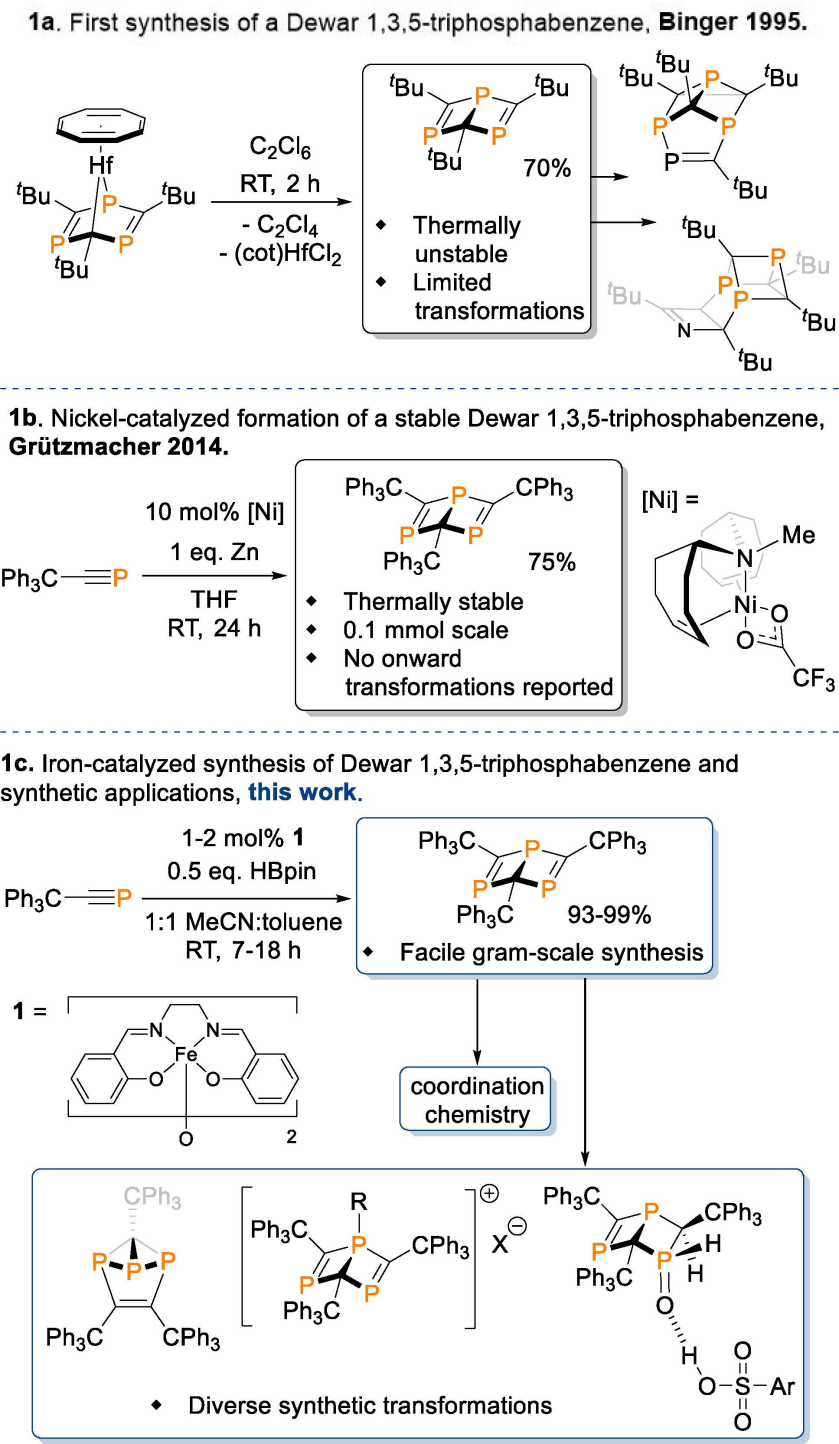
a, b) Past syntheses and transformations of Dewar 1,3,5‐triphosphabenzene species; c) this work.

Dewar 1,3,5‐triphosphabenzenes are a much less explored phosphaalkyne trimer in comparison to their symmetric 1,3,5‐triphosphabenzene valence isomers. The first example of such species, synthesized by Binger and co‐workers in 1995 via elimination from a hafnium complex (Scheme 1a),^[5]^ was thermally unstable with respect to the 1,3,5‐triphosphabenzene analogue. Despite this, further work from Binger proved the Dewar species capable of cycloaddition with terminal and internal alkynes, and with an azacyclobutadiene,^[13]^ proving that exploration of the scope and reactivity of Dewar 1,3,5‐triphosphabenzenes can bear fruit.

However, in order to realize this potential, a Dewar 1,3,5‐triphosphabenzene moiety robust enough to withstand reaction conditions beyond trapping with unsaturated substrates is necessary. Such a species was synthesized by Grützmacher and co‐workers who reported a nickel‐catalyzed cyclotrimerization of a triphenylmethyl‐substituted phosphaalkyne to form a thermally stable Dewar 1,3,5‐triphosphabenzene (Scheme 1b).^[14]^ Whilst select examples of phosphaalkynes as catalytic substrates have been reported in recent years,^[15]^ Grützmacher's work remains the sole example of a catalytic route to a Dewar 1,3,5‐triphosphabenzene. Despite this advancement, the synthesis is thus far limited to a 0.1 mmol scale, and onward reactivity of this species has not been presented.

Having shown that an [Fe(salen)]_2_‐μ‐oxo complex ((μ_2_‐oxo)‐bis(*N*,*N′*‐ethylene‐bis(salicylideneiminato‐*N*,*N′*,*O*,*O′*))‐di‐iron(III), **1**, see Scheme 1c) is a competitive pre‐catalyst for regioselective alkyne cyclotrimerization,^[16]^ we were keen to investigate whether this reactivity could be extended to triphenylmethylphosphaalkyne (**2**) and provide an improved, scalable synthetic route to 2,4,6‐tris(triphenylmethyl)‐Dewar‐1,3,5‐triphosphabenzene (**3**). This would establish a starting point for exploring variants of a stable Dewar 1,3,5‐triphosphabenzene with numerous potential applications in coordination chemistry, catalysis and main group chemistry. Herein, we present such a result: a scalable, Fe‐catalyzed route to 2,4,6‐tris(triphenylmethyl)‐Dewar‐1,3,5‐triphosphabenzene, alongside coordination chemistry and synthetic organic transformations of the Dewar 1,3,5‐triphosphabenzene moiety (Scheme 1c).

## Results and Discussion

Initial investigations were focused on the synthesis and scale up of 2,4,6‐tris(triphenylmethyl)‐Dewar‐1,3,5‐triphosphabenzene (**3**). We hypothesized that use of our air‐stable [Fe(salen)]_2_‐μ‐oxo pre‐catalyst (**1**) in the presence of a sub‐stoichiometric quantity of pinacolborane (HBpin) may prove capable of this transformation, as it was previously shown to be active in alkyne cyclotrimerization.^[16]^ Pleasingly, **3** is formed in a moderate 34 % conversion from triphenylmethylphosphaalkyne (**2**) in the presence of 1 mol % **1** and 0.5 equivalents (eq.) of HBpin after three days at room temperature on a 0.25 mmol scale (Table 1, Entry 1). After a brief optimization (see Supporting Information), it was found that use of a 1 : 1 mixture of MeCN/toluene is highly beneficial to conversion. Increasing the loading of **1** to 2 mol % in this new solvent system gives near quantitative conversion after seven hours at room temperature (Entry 2). No reaction is observed in the absence of either **1** or HBpin (Entries 3 and 4). In addition to this, replacing **1** with the iron salts FeCl_3_ and FeCl_2_ yields no conversion under the reaction conditions (Entries 5 and 6). **3** is air‐stable over short periods of time, allowing for a simple work‐up procedure (see Supporting Information) to give the isolated product in 95 % yield from the optimized reaction.

**Table 1:** Optimization of reaction conditions for the formation of **3**.

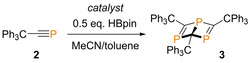					
Entry	Solvent	Catalyst (loading, mol %)	Temp. [°C]	Time [h]	Conv. [%]^[a]^
1	MeCN	**1** (1)	25	72	34
*2*	*MeCN : toluene, 1 : 1*	***1** (2)*	*25*	*7*	*>99 (95)*
3	MeCN : toluene, 1 : 1	None	80	24	0
4^[b]^	MeCN : toluene, 1 : 1	**1** (2)	80	24	0
5	MeCN : toluene, 1 : 1	FeCl_3_ (5)	80	24	0
6	MeCN : toluene, 1 : 1	FeCl_2_ (5)	80	24	0

Conditions: 0.25 mmol **2**, 0.5 eq. HBpin, 0.6 mL solvent. [a] Conversion calculated by integration of product signals against starting phosphaalkyne via ^31^P NMR spectroscopy. Isolated yields shown in parentheses. [b] No HBpin.

Attempts to elucidate the mechanism of the transformation were led by kinetic analysis of the reaction via ^31^P NMR monitoring. Variable time normalization analysis^[17]^ implicates the reaction is approximately first order with respect to **2** and 0.5 order with respect to **1**. A 30‐minute induction period is also observed. The latter two observations are in line with our previous work on alkyne cyclotrimerization, and suggest an initial reduction of pre‐catalyst **1** by HBpin to form an active monomeric Fe^II^ complex, which we propose is then capable of binding an equivalent of **2** to initiate the catalytic cycle.^[16]^

Previously, Grützmacher and co‐workers reported that **3** is thermally stable with respect to the 1,3,5‐triphosphabenzene analogue after five hours at 90 °C,^[14]^ in vast contrast to the *tert*‐butyl analogue reported by Binger and co‐workers.^[5]^ However, we note that heating a solution of **3** in toluene at 110 °C and monitoring via ^31^P NMR spectroscopy reveals the emergence of a singlet at 258.6 ppm over a two‐week period. This chemical shift is analogous to the symmetric heteroarene isomer 2,4,6‐tris(*tert*‐butyl)‐1,3,5‐triphosphabenzene synthesized by Binger and co‐workers (^31^P NMR: *δ*=232.6 (s)). We therefore propose that our newly formed species is the triphenylmethyl substituted heteroarene 1,3,5‐triphosphabenzene, **3′**. This is supported by DFT calculations, which show **3′** to be less entropically burdened than its Dewar benzene isomer **3**, and therefore slightly favoured at higher temperatures, i.e., Δ_r_ *H* ^298^(**3**→**3′**)=+1.3 kcal mol^−1^; Δ_r_ *G* ^298^(**3**→**3′**)=−1.5 kcal mol^−1^. Dewar phosphabenzene **3** is separated by a barrier of Δ^≠^ *G*=32 kcal mol^−1^ from the six‐membered ring isomer **3′**, which translates to a half‐life *t*_1/2_ of about two days at 110 °C according to simple Eyring kinetics (see Supporting Information).

**Scheme 2:**
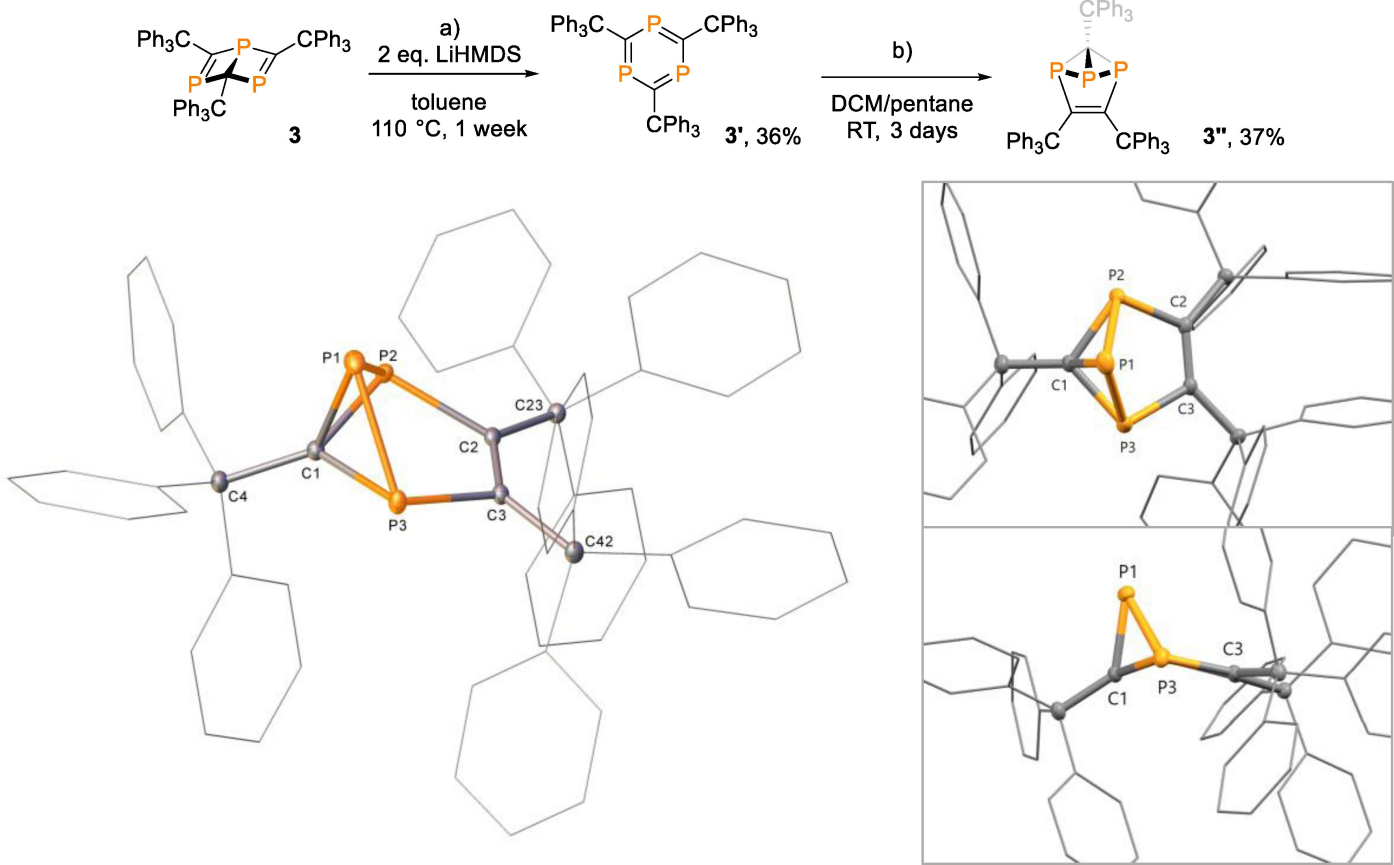
Top: formation of **3′** and **3′′** from **3**. Conditions: a) 0.2 mmol **3**, 0.4 mmol LiHMDS, 2.5 mL toluene, 110 °C, 4 days; b) DCM/pentane, vapor diffusion, 3 days. Bottom left: solid‐state structure of **3′′**. Hydrogen atoms have been omitted and wireframe view employed, for visual clarity. Ellipsoids are shown at 30 % probability.^[18]^ Bottom right: projection from top face showing the shape of the 3‐ and 5‐membered rings and side‐on projection showing apical (P1) phosphorus atom and “envelope” structure of the 5‐membered ring.

Intriguingly, heating **3** under the same conditions in the presence of lithium hexamethyldisilazane (LiHMDS) accelerates the formation of **3′**, which forms as an orange precipitate after four days at 110 °C in toluene (Scheme 2, top). It is worth noting that NaHMDS also facilitates the formation of **3′** after one week at the same temperature. However, a number of unidentified signals were also observed via ^31^P NMR spectroscopy, which are not observed in the reaction with LiHMDS (see Supporting Information). KHMDS only leads to trace conversion to **3′** after one week at 110 °C i.e. there is no discernible improvement compared to heating **3** to 110 °C alone. **3′** can be isolated by filtration from the toluene solution. Attempts to crystallize and confirm the structure of **3′** led to an interesting observation; single‐crystal X‐ray diffraction analysis of yellow crystals formed from a dichloromethane/pentane vapor diffusion reveal that **3′** rearranges under these crystallization conditions, yielding complete conversion to unique species **3′′** in a ring contraction reaction (Scheme 2, top). In **3′′**, one of the phosphorus atoms rearranges onto an adjacent P−C−P face of **3′**, resulting in a new C=C double bond in the space vacated by the migrating phosphorus atom. Single crystal X‐ray diffraction of **3′′** shows a 5‐membered phosphacycle, which has a classical envelope‐type structure that we might anticipate for a cyclopentane molecule (Scheme 2, bottom). Acute bond angles are observed for P2−P1−P3 (80.82(2)°), C1−P1−P2 (52.57(4)°) and C1−P1−P3 (52.94(4)°). P−P bond lengths are in line with that anticipated for a P−P single bond (P1−P2 2.2099(4) and P1−P3 2.1925(5) Å). The C1−P2 (1.831(1) Å) and C1−P3 (1.832(1) Å) bond lengths are slightly shorter than P2−C2 (1.876(1) Å) and P3−C3 (1.881(1) Å). The C2−C3 bond length is what we would expect for a C=C double bond (1.351(2) Å).

**Figure 1:**
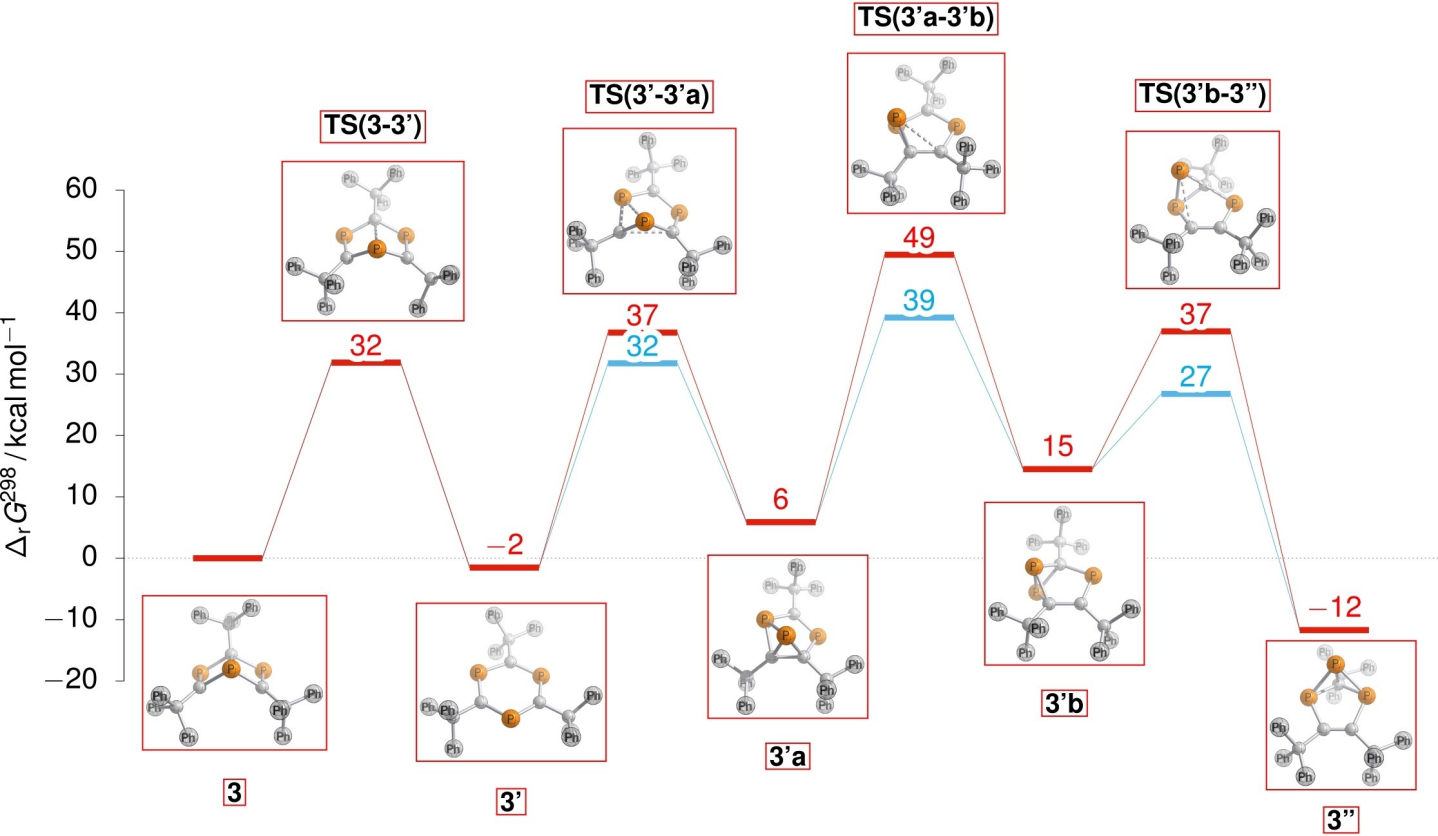
Potential energy profile for the rearrangement of Dewar phosphabenzene **3** to yield **3′′** computed at the PBE0‐D3BJ(SMD)/def2‐TZVP//def2‐SVP level. Relative Gibbs energies for closed‐shell singlet species in red color and for broken‐symmetry species in light blue color are given in kcal mol^−1^. Phenyl groups are not shown.

In accordance with our experimental observations, the mechanism predicted by DFT calculations for the (LiHMDS‐unassisted) rearrangement of Dewar phosphabenzene **3** to yield **3′′** proceeds through formation of **3′** and subsequent ring‐contraction involving concerted P−P and C=C bond formations through **TS(3′**–**3′ a)**, followed by phosphorus ring‐walk via **3′ a** and **3′ b** (Figure 1). For the three transition structures involved in the transformation of triphosphabenzene **3′** to the ring‐contraction product **3′′**, broken‐symmetry species with radical character at the sites where bonds are formed or broken are more stable than the corresponding closed‐shell singlet electromers (see Supporting Information for details). The computed individual barriers along this route range from 32 kcal mol^−1^ to 34 kcal mol^−1^, which corresponds to slow interconversion at elevated temperatures. **3′′** is kinetically and thermochemically stable as it lies 12 kcal mol^−1^ below **3** and is separated by a reverse barrier of 39 kcal mol^−1^. We note in passing that the two highest occupied molecular orbitals (HOMOs) of **3′′** exhibit large coefficients on the phosphorus atoms. In particular, the apical P atom features a marked directional *σ*‐type lobe in HOMO−1, which implies some P donor qualities (also see the Supporting Information).

**Figure 2:**
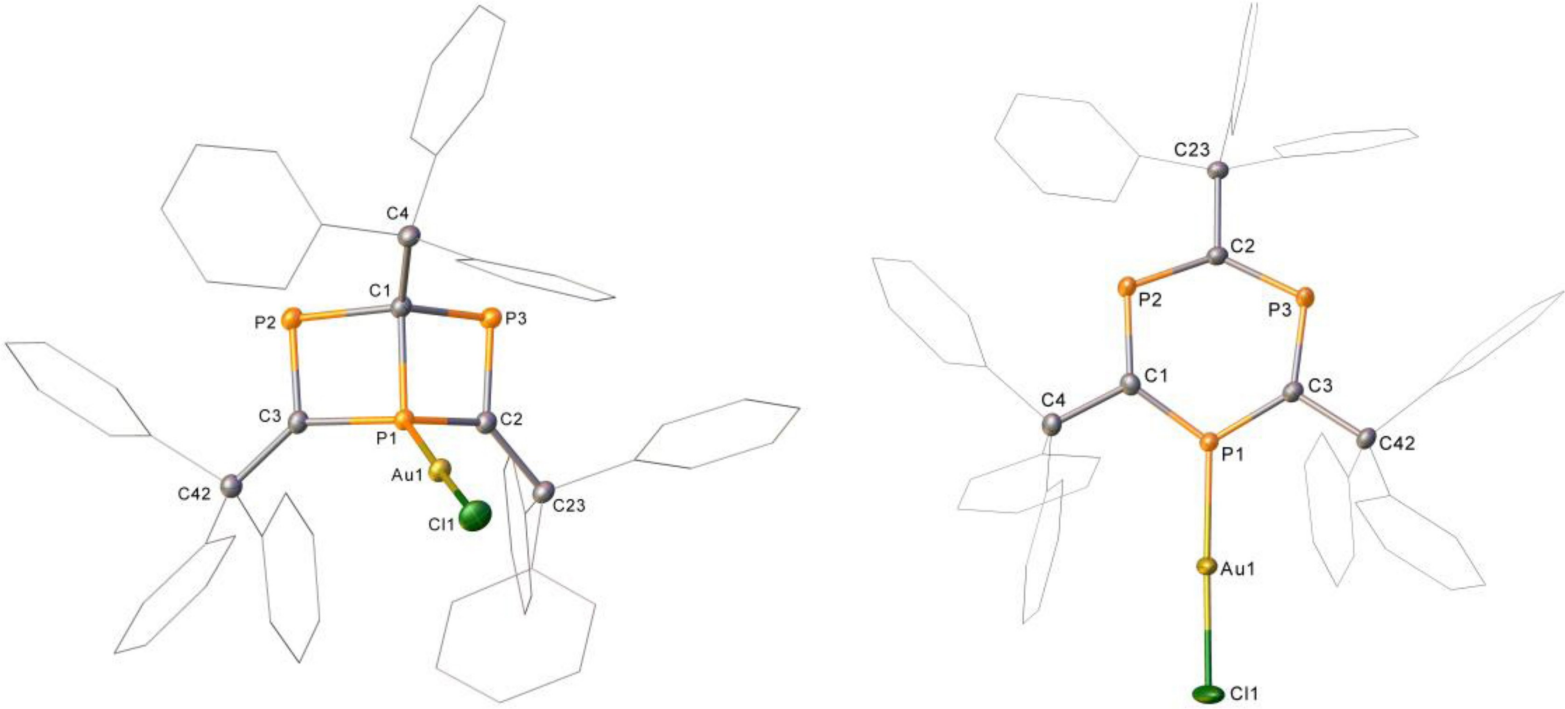
Structures of **ClAu‐3** (left) and **ClAu‐3′** (right). Hydrogen atoms and solvent have been omitted from both structures and wireframe view employed, for clarity. Ellipsoids are shown at 30 % probability. **Cl−Au‐3′** was present at 80 % occupancy in the crystal sample.^[18]^

We were intrigued by the coordination chemistry potential of **3**, **3′** and novel phosphine **3′′**. Adding a pure sample of **3** to an equimolar amount of Me_2_SAuCl in CD_2_Cl_2_ gives new ^31^P NMR resonances at 379.1 and 87.2 ppm and crystals of **ClAu‐3** are obtained after three days (Figure 2, left). Reaction of **3′** with Me_2_SAuCl gives **ClAu‐3′** (Figure 2, right). Reaction of **3′′** with Me_2_SAuCl failed to generate crystals, but analysis by NMR spectroscopy indicates coordination via P2/P3 as opposed to the apical phosphorus (P1). DFT calculations for this species substantiate that the “non‐apical” coordination is favored with respect to BDFE over Au−P(apical) coordination (see the Supporting Information).

**Scheme 3:**
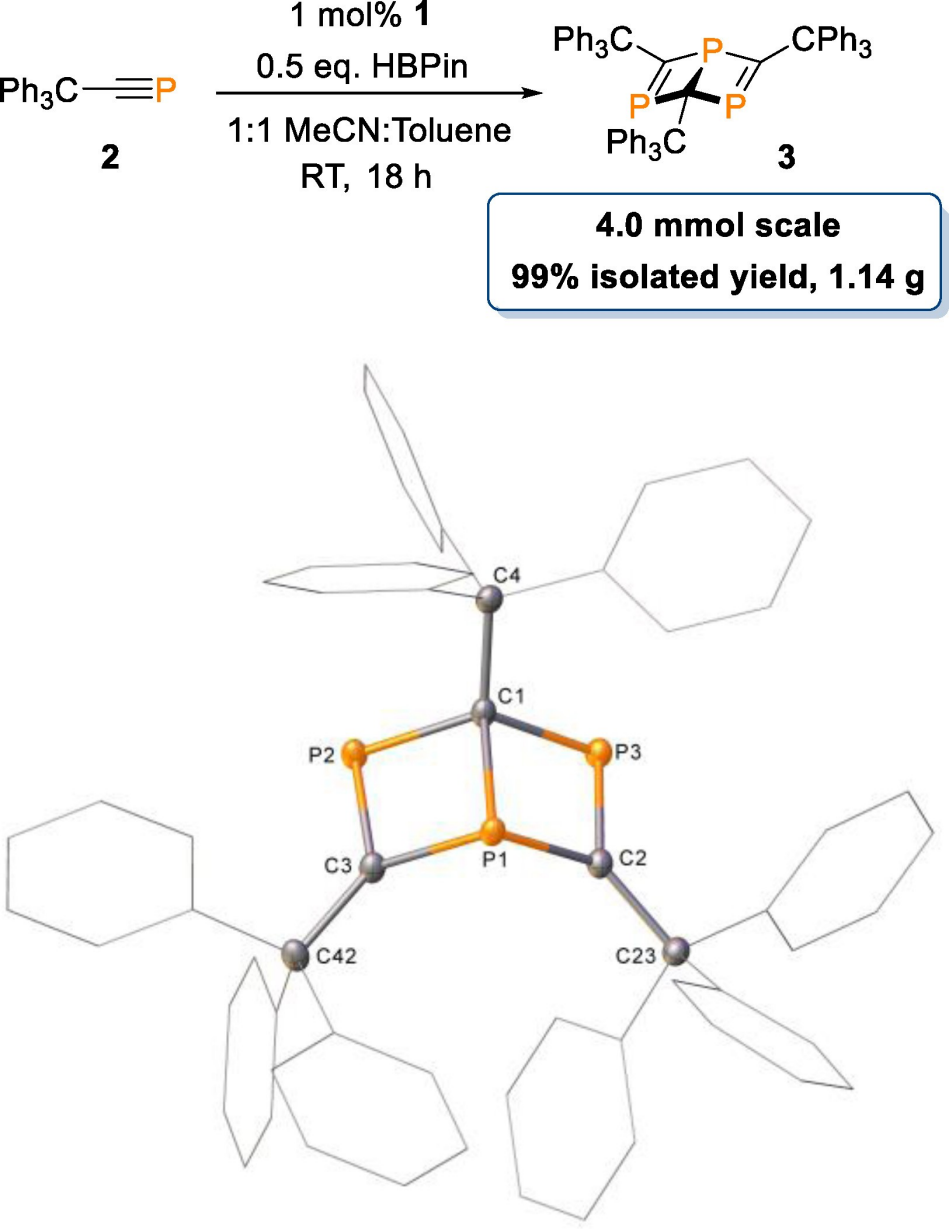
Scale‐up synthesis of **3**. Hydrogen atoms have been omitted and wireframe view employed, for visual clarity. Ellipsoids are shown at 30 % probability.^[18]^

Having found a simple and efficient synthesis for **3**, we then looked towards scale‐up development to obtain gram quantities of the product. On a 4 mmol scale, the reaction proceeds to near quantitative conversion with a lower catalyst loading (1 mol % **1**) and a slightly extended reaction time (Scheme 3). After reaction work‐up, **3** is isolated in 99 % yield, giving 1.14 g of product; proving our methodology is perfectly suited to produce **3** on a large scale without loss of conversion or selectivity.

**Scheme 4:**
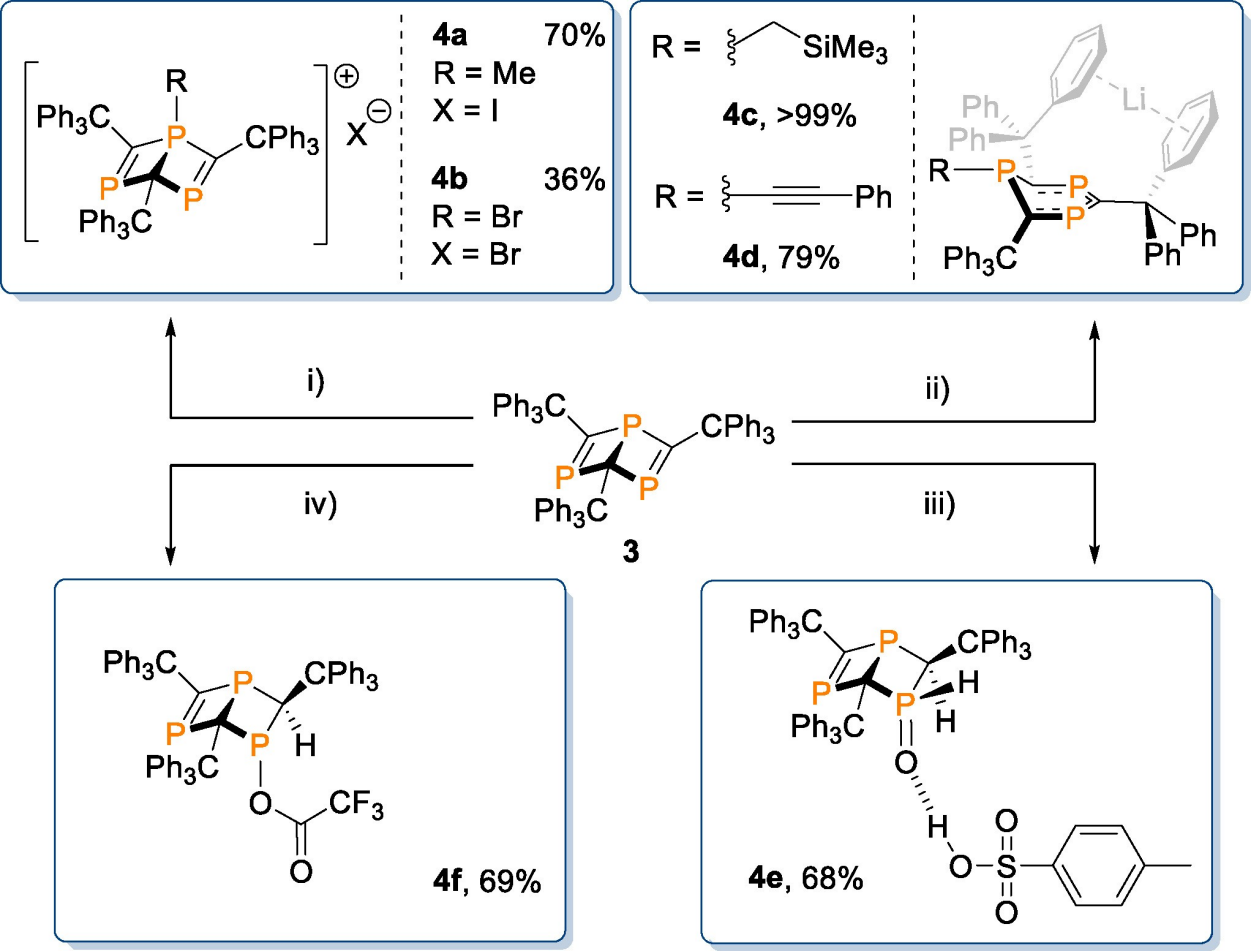
Synthetic transformations of **3**. Conditions: i) 0.05 mmol **3**, 0.1 mmol MeI/CBr_4_, 0.6 mL C_6_D_6_, 80 °C, 1 h (80 °C, 3d for **4 a**). ii) 0.05 mmol **3**, 0.05 mmol LiCH_2_TMS/LiCCPh, 0.6 mL C_6_D_6_, RT, 1 h. Spectroscopic yield shown, determined by ^31^P NMR spectroscopy. iii) 0.05 mmol **3**, 0.1 mmol *p*‐TSA monohydrate, 0.6 mL C_6_D_6_, 80 °C, 18 h. iv) 0.05 mmol **3**, 0.2 mmol TFA, 0.6 mL C_6_D_6_, 80 °C, 3 d.

Next, we began investigations into the functionalization of the Dewar benzene species. Previous functionalizations of the ^*t*^Bu‐substituted analogue of **3** have focused on trapping the moiety in cycloaddition reactions with unsaturated substrates,[^13a^, ^19^] most likely due to the instability of the Dewar species. With the inherent stability of **3**, we looked to diversify this functionality, hypothesizing that the right choice of nucleophile or electrophile may give access to direct addition reactions at the bridgehead phosphorus atom. Gratifyingly, stoichiometric reactions of **3** with methyliodide and tetrabromomethane both proceed smoothly to give dark red solutions at 80 °C. Analysis by ^31^P NMR spectroscopy reveals complete loss of starting material signals and the emergence of two new signals in each spectrum after three days (**4 a**: *δ*=292.8 (s), 67.0 (q, ^2^J_P‐H_=12.2 Hz)) and one hour (**4 b**: *δ*=289.5 (d, ^2^J_P‐P_=24.4 Hz), 26.3 (t, ^2^J_P‐P_=24.4 Hz)) respectively. The ^1^H NMR spectrum of **4 a** also shows a new signal at 0.02 ppm (d, ^2^J_H‐P_=12.0 Hz), indicating that nucleophilic attack from the bridgehead phosphorus atom of **3** onto the electrophilic methyl carbon of MeI occurs to form a new P−C bond. The resulting cationic phosphorus site carries an iodide counterion (Scheme 4i). Contrary to this reactivity, single‐crystal X‐ray diffraction analysis of **4 b** reveals that a new P−Br bond is formed at the bridgehead site rather than P−C bond formation. The differing reactivity observed between **4 a** and **4 b** is presumably due to the increased steric bulk of the CBr_3_ fragment, which disfavors P−C bond formation.

Organolithium reagents react with **3** instantly at room temperature, forming dark purple solutions in each case (Scheme 4ii). In situ ^31^P NMR spectroscopic analysis of the reaction of **3** with (trimethylsilyl)methyllithium (LiCH_2_TMS) reveals complete conversion of starting material signals and the clean formation of a broad singlet at 239.3 ppm and a sharp peak at −73.9 ppm. New signals in the ^1^H NMR spectrum of the reaction at 0.01 ppm (s, 2H) and −0.20 (s, 9H) imply that alkylation with CH_2_TMS has occurred, resulting in a resonant anionic charge in the ring system. Crystals grown of **4 c** suitable for analysis by single‐crystal X‐ray diffraction (vide infra) reveal that the bridging P−C bond is broken as a result of nucleophilic attack at the bridgehead phosphorus atom by LiCH_2_TMS, yielding a similar product to that obtained in the reaction of 2,4,6‐tris(*tert*‐butyl)‐1,3,5‐triphosphabenzene with lithium salts.^[11d]^ The residual Li cation is situated between two phenyl rings of adjacent triphenylmethyl groups. Reaction of **3** with lithium phenylacetylide yields new ^31^P NMR signals at 232.2 (d, ^2^J_P‐P_=24 Hz) and 123.0 ppm (t, ^2^J_P‐P_=24 Hz). We propose that similar reactivity is observed in this case to give **4 d**, and that the coupling between phosphorus environments observed in this case is due to stabilization of the Li cation with THF present in the reaction mixture (see Supporting Information). The contrasting reactivity of **3** to form **4 a/b** and **4 c/d** demonstrates the ambiphilic nature of the bridgehead phosphorus site. Unfortunately, species **4 c** and **4 d** could not be isolated in the solid state, as removal of volatiles from the crystallized material results in the formation of multiple new unidentified species observable by ^31^P NMR spectroscopy.

Reaction of **3** with an excess of *para*‐toluene sulfonic acid (*p*‐TSA) monohydrate yields a yellow color change along with complete consumption of starting material by ^31^P NMR spectroscopy after 18 h at 80 °C (Scheme 4iii). Clean conversion to three new ^31^P environments is observed, with signals at 389.0 (d, ^2^J_P‐P_=24 Hz), 23.3 (apparent t (dd), ^2^J_P‐P_=^2^J_P‐H_=26.9 Hz) and 13.6 ppm (apparent dt (ddd), ^1^J_P‐H_=516.6 Hz, ^2^J_P‐P_=^2^J_P‐H_=24.4 Hz). The new ^31^P‐^1^H couplings suggest **3** is protonated in two separate positions, which is further enforced by new signals at 7.39 (dd, 1H, ^1^J_P‐H_=516.6 Hz, ^3^J_H‐H_=4.7 Hz) and 5.09 ppm (dd, 1H, ^2^J_H‐P_=24.4 Hz, ^3^J_H‐H_=4.7 Hz) in the ^1^H NMR spectrum. The ^31^P‐^1^H couplings of these signals were confirmed by a ^31^P‐^1^H COSY NMR experiment (see Supporting Information). Crystals grown via dichloromethane/pentane slow diffusion and analyzed by single‐crystal X‐ray diffraction reveal that the new protons are added over one of the P=C bonds of **3**, with the phosphorus atom oxidized to give **4 e**. The resulting P=O bond forms a hydrogen bond with the acidic proton of a molecule of *p*‐TSA. The *p*‐TSA moiety is present in a 1 : 1 ratio to the ring structure of **4 e** in solution (confirmed by ^1^H NMR spectroscopy). As the acid moiety retains its acidic proton, we propose that both the new hydrogen and oxygen atoms originate from the water from the monohydrate component of the starting acid. Interestingly, no reaction between **3** and an excess of water is observed in benzene or THF at 80 °C, or with **3** and other hydrated acids (oxalic acid dihydrate, citric acid monohydrate). Additionally, no reactivity is observed between **3** and *ortho‐*fluorobenzoic acid and water. This implicates that the addition of water over the phosphaalkene moiety is facilitated exclusively by the *p*‐TSA (see Supporting Information).

The reactivity of **3** with acids was further probed by the addition of an excess of trifluoroacetic acid (TFA) to a C_6_D_6_ solution of **3**. After heating at 80 °C for three days, new ^31^P NMR signals are observed at 403.4 (d, ^2^J_P‐P_=33.5 Hz), 127.0 (d, ^2^J_P‐P_=33.5 Hz) and 78.4 ppm (at, ^2^J_P‐P_=33.5 Hz) alongside complete conversion of **3**. A new signal is observed in the ^1^H NMR spectrum which shows coupling to two phosphorus environments: 3.95 (dd, 1H, ^2^J_H‐P_=6.5 Hz, ^2^J_H‐P_=2.5 Hz). The loss of symmetry of the P=C moieties as well as a new proton environment indicate that reactivity over a single P=C site has again occurred, with protonation at the carbon along with formation of a (trifluoromethyl)acetoxyphosphine group^[20]^ (**4 f**, Scheme 4iv). Single crystal X‐ray diffraction analysis of crystals grown from a dichloromethane/pentane slow diffusion confirm this reactivity (vide infra). In contrast to the reaction with *p‐*TSA monohydrate, no oxidation of the phosphorus environments is observed in this case with the use of an anhydrous acidic species.

**Figure 3:**
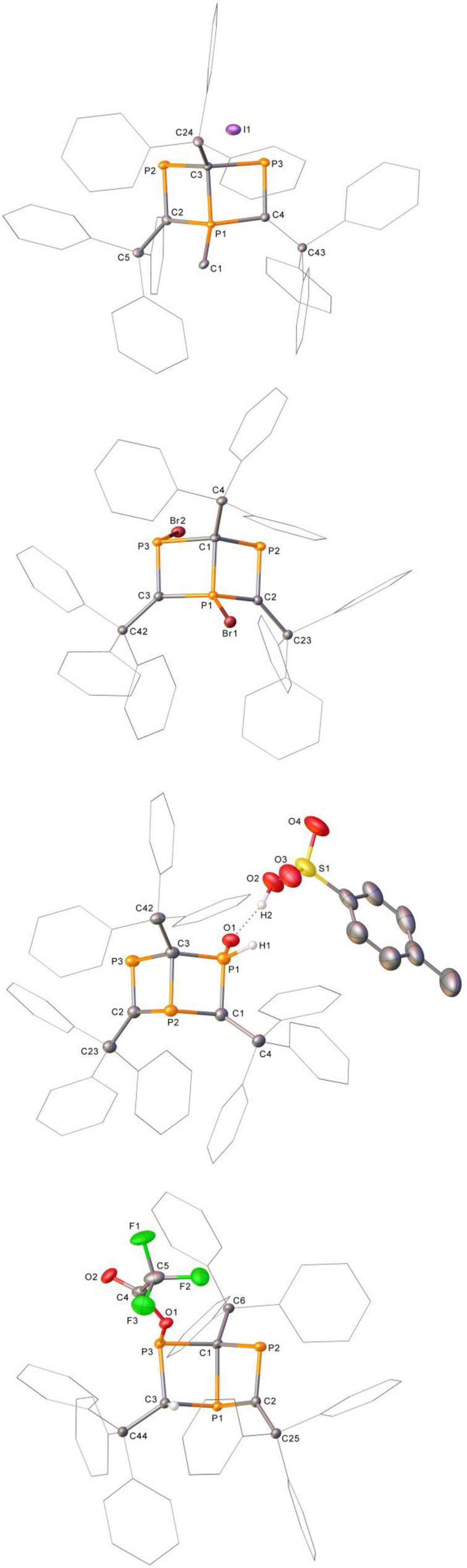
Single‐crystal X‐ray structures of **4 a**, **4 b**, **4 e** and **4 f** (top to bottom). Hydrogen atoms have been omitted throughout, with the exceptions of H1 and H2 in **4 e** and H3 in **4 f**. Disorder (**4 f**) and solvent have been omitted (**4 a**, **4 f**) for clarity. Wireframe view has also been employed, again, for clarity and ellipsoids are shown at 30 % probability.^[18]^

**4 a**, **4 b**, **4 c**, **4 e** and **4 f** are readily crystallized from toluene or dichloromethane/pentane vapour diffusions (Figure 3). Relevant bond lengths/angles for Dewar benzene products **4 a**, **4 b**, **4 e** and **4 f**, in comparison to **3**, are shown in the Supporting Information. **4 a** has bond lengths similar to **3**, though phosphorus‐carbon bonds to the cationic phosphorus of **4 a** (P1−C2, P1−C3, P1−C4) show a reduction in length to the comparative bonds of **3** (1.79 Å average in **4 a**, 1.85 Å in **3**). More distinct is the expansion of the C2−P1−C3 bond angle from 108.78(7)° in **3** to 122.2(3)° in **4 a** (labelled C2−P1−C4). There is a disparity in the bond angles of **4 a** and **4 b**; the latter possesses a significantly constricted angle between the bridging P1−C1 bond and the new Br substituent in comparison to the new methyl unit in **4 a** (C1−P1−Br1=117.70(8)° in **4 b**, C3−P1−C1=125.5(3)° in **4 a**). **4 e** possesses a slightly elongated bridging P2−C3 bond (1.920(3) Å in **4 e**, 1.871(1) Å for P1−C1 in **3**) as well as an expected lengthened P1−C1 bond length in **4 e** due to the breaking of the pre‐existing phosphorus‐carbon double bond (1.814(3) Å in **4 e**, 1.697(1) and 1.689(1) Å for P2−C3 and P3−C2 in **3**). In addition to this, a reduction in the C1−P2−C2 bond angle is observed for **4 e** (98.4(1)°) compared to 108.78(7)° for C2−P1−C3 in **3**. **4 f** closely mirrors the same distortions of the Dewar 1,3,5‐triphosphabenzene structure as **4 e**.

**Figure 4:**
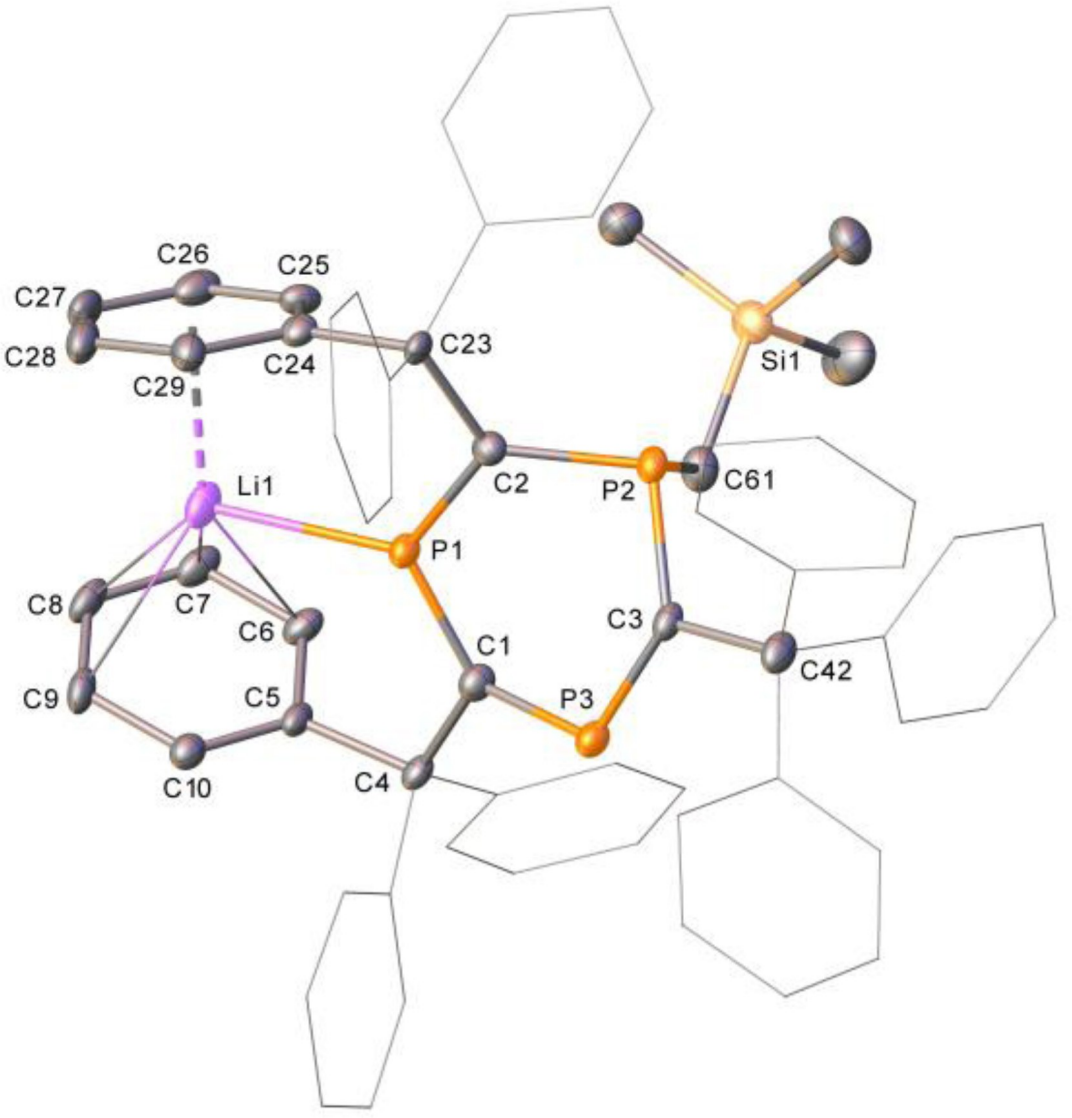
Single‐crystal X‐ray structure of **4 c**. One of the four molecules in the asymmetric unit of the single‐crystal X‐ray structure of **4 c**. Solvent, hydrogen atoms and disorder have been omitted for clarity. Ellipsoids are shown at 30 % probability.^[18]^

**4 c** (Figure 4) possesses very similar P−C bond lengths to **3**, despite the bridging bond between P1 and C1 being broken. An expected widening of P−C−P bond angles is observed from **3** to **4 c** (96.99(7)°, 95.90(7)° and 118.63(8)° in **3** and 120.6(3)°, 126.5(3)° and 122.8(3)° in **4 c**).

## Conclusion

To summarize, an iron‐catalyzed cyclotrimerization of triphenylmethylphosphaalkyne (**2**) has enabled access to gram‐scale quantities of 2,4,6‐tris(triphenylmethyl)‐Dewar‐1,3,5‐triphosphabenzene (**3**) in a simple, mild procedure. Formation of the 1,3,5‐triphopshabenzene valence isomer of **3**, **3′**, can be facilitated by a simple lithium salt (LiHMDS), which upon isolation and crystallization rearranges in a ring contraction transformation, resulting in the migration of a phosphorus atom onto an adjacent P−C−P face of **3′** to form **3′′**. Density functional theory calculations provide a four‐step mechanism for this rearrangement involving open‐shell transition structures that is consistent with the experimental information. The coordination chemistry potential of **3**, **3′** and **3′′** was demonstrated by formation of gold complexes. The synthetic capabilities of **3** have been demonstrated for the first time; nine novel species, illustrating the versatile reactivity of the Dewar benzene compound as both an electrophile and nucleophile, have been synthesized and characterized. Solid‐state structures have been presented for reactions of **3** with methyliodide (**4 a**), tetrabromomethane (**4 b**), (trimethylsilyl)methyllithium (**4 c**), *para*‐toluene sulfonic acid (**4 e**) and trifluoroacetic acid (**4 f**).

## Conflict of interest

The authors declare no conflict of interest.

1

## Supporting information

As a service to our authors and readers, this journal provides supporting information supplied by the authors. Such materials are peer reviewed and may be re‐organized for online delivery, but are not copy‐edited or typeset. Technical support issues arising from supporting information (other than missing files) should be addressed to the authors.

Supporting Information

## Data Availability

The data that support the findings of this study are available in the supplementary material of this article.
