# Supplementary material for: An Iron‐Catalyzed Route to Dewar 1,3,5‐Triphosphabenzene and Subsequent Reactivity
Source: Angew Chem Int Ed Engl. 2022 Aug 9;61(37):e202208663. doi: 10.1002/anie.202208663 (PMC9540597; doi:10.1002/anie.202208663)
Supplement: Supplementary file 1 — Supporting Information [file ANIE-61-0-s001.pdf]

Angewandte Chemie
Eine Zeitschrift der Gesellschaft Deutscher Chemiker

Supporting Information

An Iron-Catalyzed Route to Dewar 1,3,5-Triphosphabenzene and Subsequent Reactivity

A. N. Barrett, M. Diefenbach, M. F. Mahon, V. Krewald*, R. L. Webster**

An Iron-Catalysed Route to Dewar 1,3,5-Triphosphabenzene and Subsequent Reactivity

Adam N. Barrett[a], Martin Diefenbach,[b] Mary F. Mahon,[a] Vera Krewald,[b] and Ruth L. Webster[a]*

[a]Department of Chemistry, University of Bath, Claverton Down, Bath, BA2 7AY, United Kingdom
[b]Department of Chemistry, TU Darmstadt, 64287 Darmstadt, Germany

rw498@bath.ac.uk

Contents

1. General Considerations 1
2. Starting Material and Substrate Syntheses 2
3. Optimization of 2,4,6-tris(triphenylmethyl)-Dewar-1,3,5-triphosphabenzene (3) Synthesis 4
4. Synthesis, Scale-up Synthesis and Spectroscopic Data for 2,4,6-tris(triphenylmethyl)-Dewar-1,3,5-triphosphabenzene (3) 5
5. Synthesis and Spectroscopic Data Scheme 2 7
6. Synthesis and Spectroscopic Data for Gold Complexes 12
7. Synthesis and Characterisation Data Scheme 4 16
8. Kinetic Data for the Formation of 3 30
9. Reactions of 3 with NaHMDS and KHMDS 34
10. Investigations into the Reactivity of 1 with Further Phosphaalkynes 35
11. Crystallographic Information 39
12. Quantum-chemical Details 50
12.1 Computational Methods 50
12.2 Energies 51
12.3 Spin Densities 52
12.4 Frontier Molecular Orbitals for 3 53
12.5 Frontier Molecular Orbitals for 3’ 53
12.6 Frontier Molecular Orbitals for 3’’ 53
12.7 Au Complexation with 3 54
12.8 Au Complexation with 3’ 55
12.9 Au Complexation with 3’’ 56
13. References 57
A. Appendix 59

1. General Considerations

All manipulations were carried out under an inert atmosphere using standard Schlenk and glovebox techniques, unless otherwise stated. Phosphorus trichloride, *n*-Butyllithium, triphenylmethane, 1,4-diazabicyclo[2.2.2]octane, *p*-Toluenesulfonic acid monohydrate, iodomethane, tetrabromomethane and phenyldisulfide were purchased from commercial sources and used as supplied. Pinacolborane was purchased from commercial sources and distilled before use. $LiCH_2TMS$ was purchased from a commercial source as a solution in hexanes and was concentrated in vacuo to be used in a glovebox as a solid. $[Fe(salen)]_2$-μ-oxo (**1**) was prepared via a literature procedure.[1] Pentane, diethyl ether, THF, and toluene were dried over sodium/benzophenone and distilled before use. Dichloromethane, dibromomethane and acetonitrile were dried over calcium hydride and distilled before use. NMR data was collected at 400 or 500 MHz on Bruker or Agilent instruments in $C_6D_6/CD_2Cl_2/CDCl_3/CD_3CN/C_7D_8$ at 298 K and referenced to residual protic solvent. Crystal structures were obtained from either a Rigaku Oxford Diffraction Xcalibur (MoKα (λ = 0.71073)) or Supernova (CuKα (λ = 1.54184)) diffractometer. HRMS analyses were performed using an Agilent QTOF 6545 with Jetstream ESI spray source coupled to an Agilent 1260 Infinity II Quat pump HPLC with 1260 autosampler, column oven compartment and variable wavelength detector (VWD). Melting point analyses were conducted on a Stuart SMP10 melting point apparatus. Infrared spectra were recorded at ambient temperature on a Perkin Elmer Spectrum 100 FT-IR spectrometer using a diamond ATR unit. Intensities are reported relative to the most intense signal as vw (very weak), w (weak), s (strong) or vs (very strong).

2. Starting Material and Substrate Syntheses

Ph_3CCH_2Cl

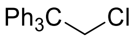

Following a literature procedure,[2] *n*-Butyllithium (42.0 mmol, solution in hexanes) was added dropwise to a THF (100 mL) and diethyl ether (40 mL) solution of triphenylmethane (10.0 g, 40.9 mmol) at 0 °C before warming to room temperature and stirring for one hour. The resulting dark red solution was then transferred dropwise via cannula to a Schlenk flask containing DCM (100 mL). Any remaining precipitate was redissolved in THF and added to the DCM vessel. The resulting solution was stirred for one hour at room temperature before being quenched with water (100 mL). The organic layer was separated, and the aqueous layer extracted with ethyl acetate (2 x 25 mL) before the combined organic extracts were dried over magnesium sulphate. Filtration and removal of volatiles in vacuo yielded a dark red crude material. The crude product was crystallised from hot cyclohexane and washed with further cyclohexane to yield Ph_3CCH_2Cl as a white solid (6.25 g, 52%).

^{1}H NMR (300 MHz, 298 K, $CDCl_3$): δ 7.20-7.35 (m, 15H, Ar-*H*), 4.63 (s, 2H, CH_2Cl). The values are in accordance to the literature.[2]

$Ph_3CCH_2PCl_2$

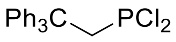

In an adaption of a literature procedure,[3] magnesium turnings (5.18 g, 213 mmol) were added to a Schlenk flask with a water-cooled reflux condenser attached. The magnesium turnings were flame-dried under vacuum, before being suspended in THF (20 mL). A small crystal of iodine was then added to activate the magnesium turnings, resulting in a brown suspension. The suspension was stirred at room temperature until the brown colour dissipated, before addition of a THF solution (20 mL) of Ph_3CCH_2Cl (6.25g, 21.3 mmol). The suspension was stirred at reflux for 6 hours to give a red solution before being cooled to room temperature. The Grignard reagent was then transferred via cannula filter to a THF solution (10 mL) of PCl_3 (5.58 mL, 63.9 mmol) at -10 °C. The reaction was then allowed to warm to room temperature over 18 hours before removal of all volatiles in *vacuo*. The residue was then extracted with diethyl ether (3 x 20 mL) and filtered through a pad of celite. Volatiles were then removed in vacuo to give a solid yellow crude material. The crude product was crystallised from hot acetonitrile and washed with further acetonitrile to yield $Ph_3CCH_2PCl_2$ as white crystals (3.2 g, 42%).

^{1}H NMR (500 MHz, 298 K, C_6D_6): δ 7.14 (d, 6H, J = 8.0 Hz, Ar-*H*) 7.03-6.98 (m, 6H, Ar-*H*), 6.97-6.93 (m, 3H, Ar-*H*), 3.80 (d, 2H, $^2J_{H-P}$ = 3.9 Hz, CH_2PCl_2). **^{31}P{^{1}H} NMR** (162 MHz, 298 K, C_6D_6): δ 185.6 (s). The values are in accordance to the literature.[3]

Ph_3CCP (2)

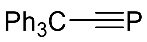

In an adaptation of a literature procedure,[3] $Ph_3CCH_2PCl_2$ (3.0 g, 8.35 mmol) was added to a Schlenk flask along with acetonitrile (50 mL). 1,4-diazabicyclo[2.2.2]octane (DABCO) (12.0 g, 107 mmol) and acetonitrile (40 mL) were added to a separate Schlenk flask and both vessels were heated to 75 °C until all material was dissolved. The DABCO solution was then added, in portions via cannula, to the solution of $Ph_3CCH_2PCl_2$ at 75 °C over a 30 min period. Upon addition, a white vapour was observed, and the solution turned pale yellow. After complete addition, the solution was stirred for a further 4 h at 75 °C before being allowed to cool to room temperature. Volatiles were removed in vacuo, and toluene (50 mL) and an O_2-free saturated NH_4Cl aqueous solution (50 mL) were added. The toluene layer was

extracted via cannula and the aqueous layer extracted with further toluene (2 x 20 mL). The organic extracts were then filtered through a pad of basic alumina, eluting with toluene (40 mL). The yellow solution was then concentrated in vacuo to give a tarry yellow solid. The solid was dissolved in a minimum amount of acetonitrile at 75 °C and left to warm to room temperature overnight. The resulting pale yellow crystals were washed with acetonitrile (2 x 5 mL) and dried under vacuo to give pure Ph_3CCP (**2**) (1.42 g, 59%).

^{1}H NMR (500 MHz, 298 K, C_6D_6): δ 7.42-7.38 (m, 6H, Ar-*H*) 7.03-6.98 (m, 6H, Ar-*H*). **^{31}P{^{1}H} NMR** (162 MHz, 298 K, C_6D_6): δ -48.3 (s). The values are in accordance to the literature.[3]

3. Optimization of 2,4,6-tris(triphenylmethyl)-Dewar-1,3,5-triphosphabenzene (3) Synthesis

Table S1: Optimization of conditions for the formation of **3**.

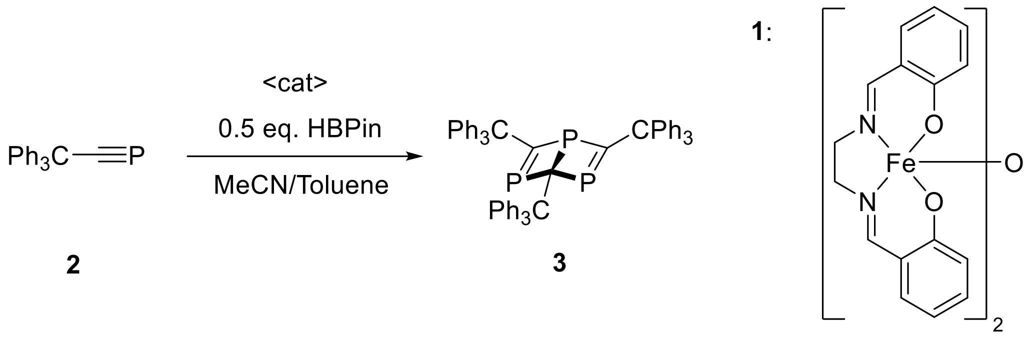

Entry	Solvent	Catalyst	Temperature / °C	Time / h	Conversion / %
1	MeCN	1 mol% 1	25	72	34
2	MeCN	1 mol% 1	80	24	90
3	Toluene	1 mol% 1	80	72	0
4	1:10 MeCN:Toluene	1 mol% 1	80	72	56
5	1:1 MeCN Toluene	1 mol% 1	80	5	97
6	2:1 MeCN:Toluene	1 mol% 1	80	5	71
7	1:2 MeCN:Toluene	1 mol% 1	80	5	95
8	1:1 MeCN Toluene	2 mol% 1	60	2	>99
9	1:1 MeCN Toluene	2 mol% 1	25	7	>99 (95)
10	1:1 MeCN Toluene	None	80	24	0
11[c]	1:1 MeCN Toluene	2 mol% 1	80	24	0
12	1:1 MeCN Toluene	5 mol% $FeCl_3$	80	24	0
13	1:1 MeCN Toluene	5 mol% $FeCl_2$	80	24	0

Conditions: 0.25 mmol **2,** 0.6 mL solvent. Approximate conversions calculated by integration of product signals against starting material in the ^{31}P NMR spectrum. Isolated yield given in parenthesis.

4. Synthesis, Scale-up Synthesis and Spectroscopic Data for 2,4,6-tris(triphenylmethyl)-Dewar-1,3,5-triphosphabenzene (3)

0.25 mmol Scale:

In a J-Young's NMR tube, $[Fe(salen)]_2$-µ-oxo (3.6 mg, 2 mol%) was added, along with Ph_3CCP (72 mg, 0.25 mmol). The solids were then dissolved in a 1:1 mixture of acetonitrile/toluene (600 µL). HBpin (20.5 µL, 0.6 mmol) was then added and the reaction was left for seven hours at room temperature. During this time, a subtle change in colour of the solution from burgundy to auburn was observed. Volatiles were removed from the reaction mixture in vacuo, before the resulting dark solid was exposed to air and dissolved in wet dichloromethane (1 mL). The dichloromethane solution was then filtered through a small silica column, eluting with more dichloromethane (2 mL). Volatiles were again removed in vacuo to yield 2,4,6-tris(triphenylmethyl)-Dewar-1,3,5-triphosphabenzene (**3**) as an orange solid (65.6 mg, 92%).

4.0 mmol Scale:

In a J-Young's ampule, $[Fe(salen)]_2$-µ-oxo (**1**) (26.0 mg, 1 mol%) was added, along with Ph_3CCP (1.15 g, 4 mmol). The solids were then dissolved in a 1:1 mixture of acetonitrile/toluene (10 mL). HBpin (328 µL, 9.6 mmol) was then added and the reaction was stirred for 18 hours at room temperature. During this time, an orange precipitate crashed out of solution. Volatiles were removed from the reaction mixture in vacuo before the resulting orange solid was exposed to air and dissolved in wet dichloromethane (5 mL). The dichloromethane solution was then filtered through a silica column, eluting with more dichloromethane (30 mL). Volatiles were again removed in vacuo to yield 2,4,6-tris(triphenylmethyl)-Dewar-1,3,5-triphosphabenzene (**3**) as an orange solid (1.13 g, 99%).

2,4,6-tris(triphenylmethyl)-Dewar-1,3,5-triphosphabenzene:

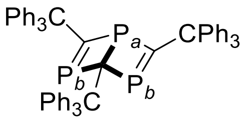

^{1}H NMR (500 MHz, 298 K, C_6D_6): δ 7.60 (s (br), 6H, Ar-*H*) 7.15-7.12 (m, 12H, Ar-*H*), 7.04-6.97 (m, 9H, Ar-*H*), 6.95-6.86 (m, 18H, Ar-*H*) . **^{31}P{^{1}H} NMR** (162 MHz, 298 K, C_6D_6): δ 346.0 (d, 2P, $^2J_{P-P}$ = 37.2 Hz, P_b), 86.3 (t, 1P, $^2J_{P-P}$ = 37.2 Hz, P_a). The values are in accordance to the literature.[4]

^{1}H NMR (500 MHz, 298 K, C_6D_6):

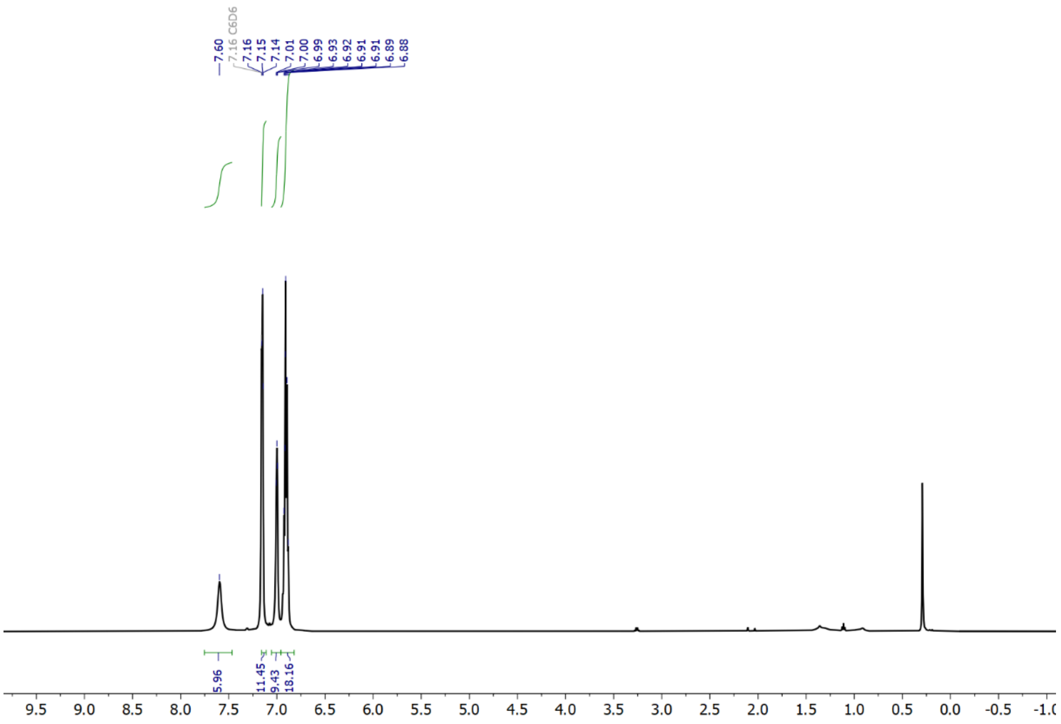

^{31}P{^{1}H} NMR (162 MHz, 298 K, C_6D_6):

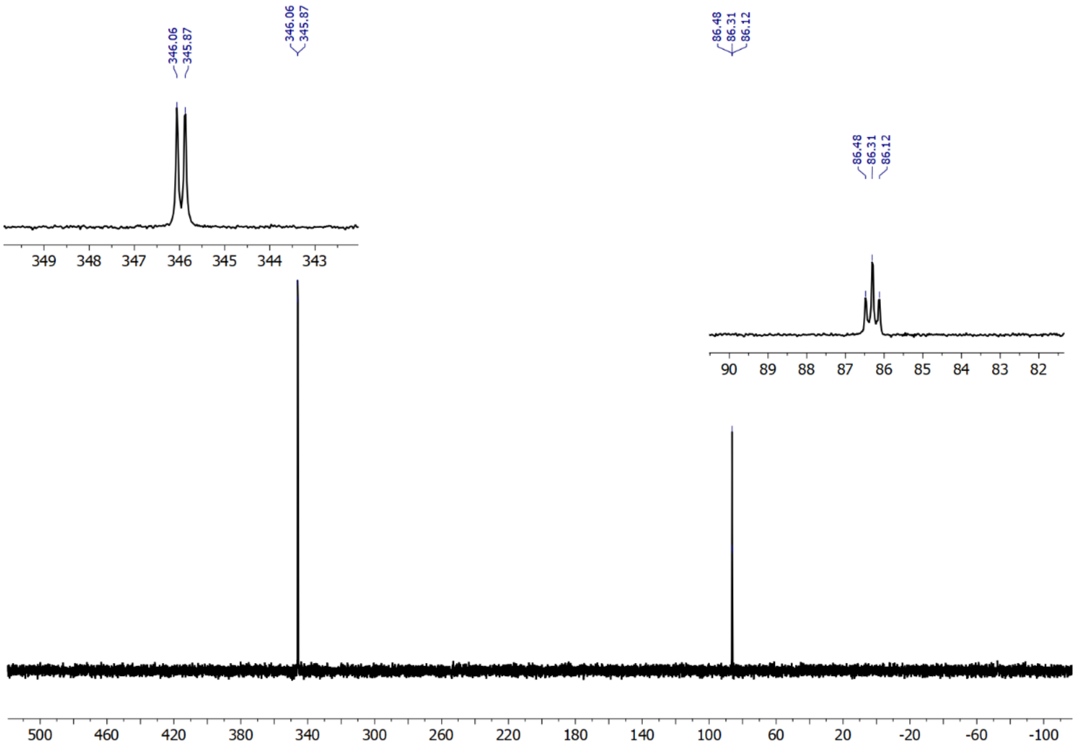

5. Synthesis and Spectroscopic Data Scheme 2

Note on NMR spectra:

Despite running $^{13}C\{^1H\}$ analysis on a spectrometer equipped with a cryoprobe, in multiple cases, quaternary carbon environments, especially those showing coupling to phosphorus environments, were poorly resolved/not observed. A previous reports of **3** gave similar observations for the quaternary -*C*=P, environments, where the C-P coupling could not be resolved.[4]

In the case of **3'**, the sample was also very poorly soluble in a range on NMR solvents tested, and minimal signals in the $^{13}C\{^1H\}$ spectrum were observed despite multiple attempts at collecting better spectra.

NMR spectra of **3''** and **ClAu-3** were undertaken using CD_2Cl_2 contaminated with silicone grease carried through from solvent purification processes, hence high quantities are observed in the respective ^1H NMR spectra. This does not represent the purity of the prior-isolated crystalline solids. As mentioned above, **3'** and **3''** only showed small amounts of solubility in CD_2Cl_2. Therefore, relative integrals of solvent impurities are not fully representative of the isolated product. Correction of yield via ^1H NMR spectroscopy is consequently not applicable in these cases.

3'

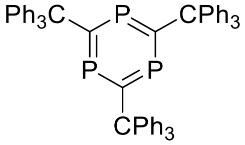

2,4,6-tris(triphenylmethyl)-Dewar-1,3,5-triphosphabenzene (0.2 mmol, 172 mg) was added to a J-Young's ampule along with toluene (2.5 mL). Lithium hexamethyldisilazane (66.96 mg, 0.4 mmol) was then added. The reaction was then heated to 110 °C, upon which the colour of the solution changed from orange to dark red. The reaction was left at this temperature for four days, and formation of an orange precipitate is observed. The precipitate was isolated by filtration and washed with toluene* (3 x 1 mL) before being dried in vacuo to give **3'** as an orange-yellow solid (62 mg, 36%).

^{1}H NMR (500 MHz, 298 K, CD_2Cl_2): δ 7.29 (d, 18H, $^3J_{H-H}$ = 7.8 Hz, -C*Ph3*), 7.16 (at, 18 H, $^3J_{H-H}$ = 7.7 Hz, -C*Ph3*), 7.10 (at, $^3J_{H-H}$ = 7.1 Hz, 9H, -C*Ph3*). **^{31}P NMR** (162 MHz, 298 K, CD_2Cl_2): δ 256.8 (s,). **^{13}C{^{1}H} NMR** (101 MHz, 298 K, CD_2Cl_2): 131.8 (C*Ph3*), 128.0 (C*Ph3*), 126.1 (C*Ph3*). **Melting point:** 274-278 °C (decomp., yellow to dark red). **IR:** (ATR, 298 K): 3082 (vw), 3054 (vw), 3026 (vw), 1597 (vw), 1489 (w), 1440 (w), 1186 (vw), 1166 (vw), 1103 (w), 1085 (w), 1037 (w), 997 (w), 924 (vw), 880 (vw), 792 (w), 747 (w), 723 (s), 695 (vs), 661 (s), 636 (s), 627 (s), 618 (s). **HRMS** (ESI): 858.2800 m.z $[M]^+$ (calculated: 858.2734). **Elemental analysis:** calcd. for $C_{60}H_{45}P_3$: C: 83.9%, H: 5.28%; found: C: 83.82, H: 5.14%.

*Prior to this, the crude precipitate was found to be insoluble in crystallisation attempts with benzene, toluene and THF (and only partially soluble in DCM), therefore the product was isolated by washing with toluene.

^{1}**H NMR** (500 MHz, 298 K, CD_2Cl_2):

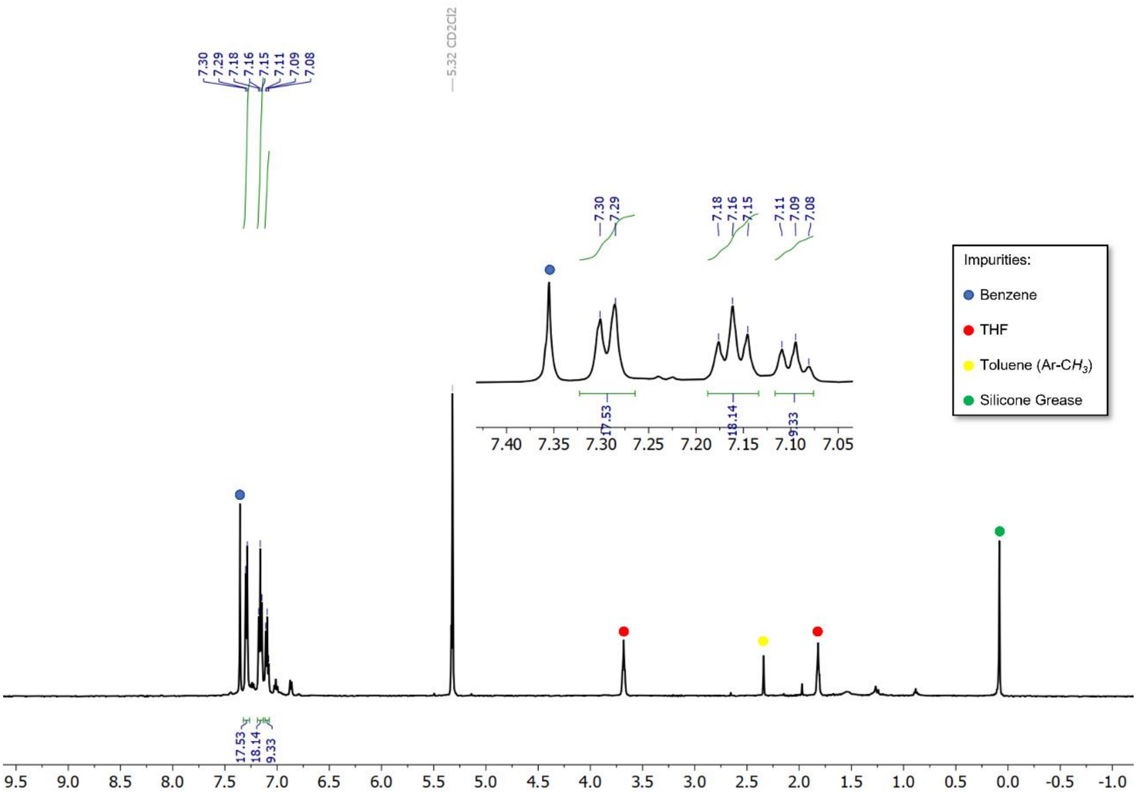

^{31}**P NMR** (162 MHz, 298 K, CD_2Cl_2):

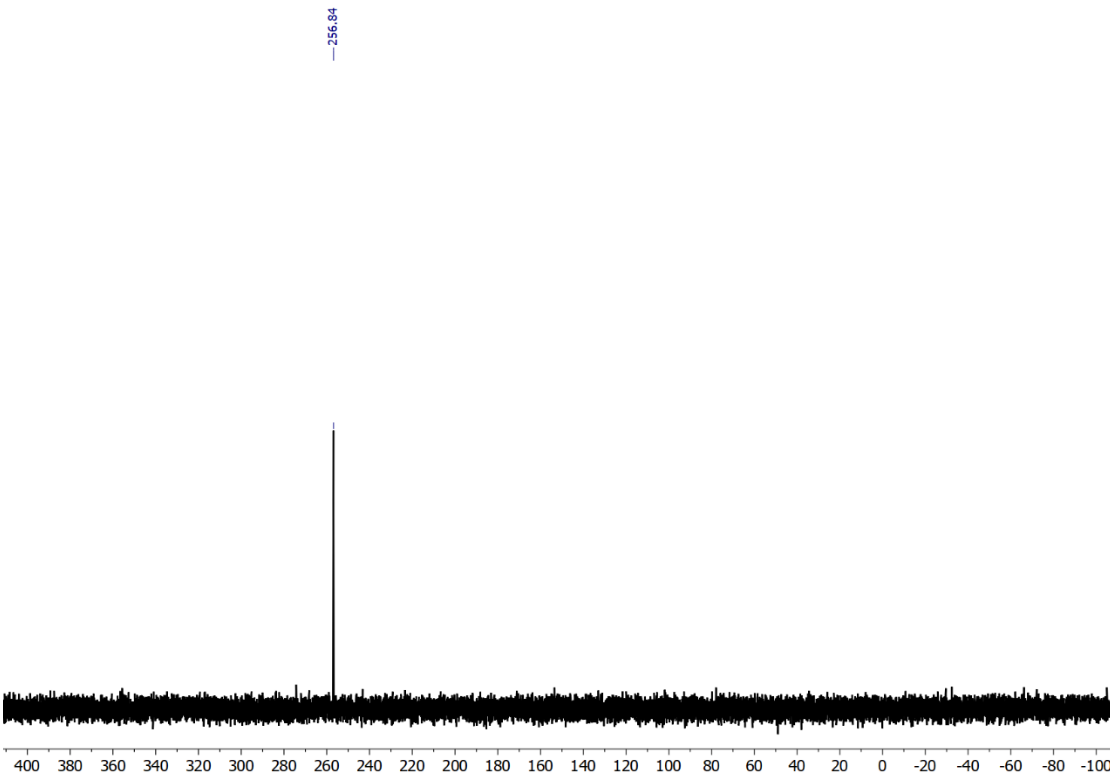

$^{13}C\{^1H\}$ NMR (101 MHz, 298 K, CD_2Cl_2):

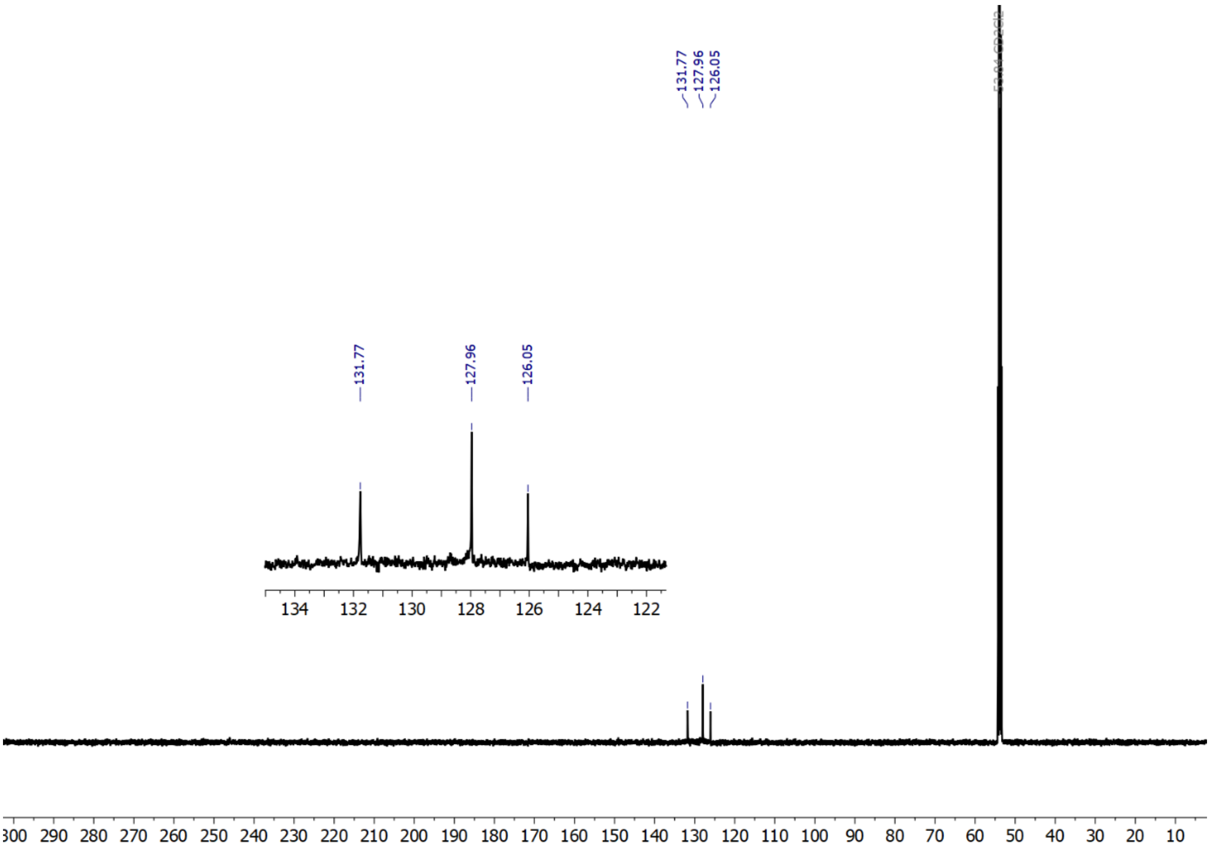

3''

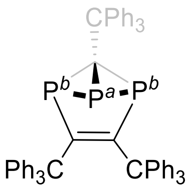

3' (62 mg, 0.072 mmol) was added to a J-Young's ampule, and dichloromethane (5 mL) was added. The suspension was stirred and heated to 50 °C for 10 mins to allow for maximum dissolution. The yellow solution was then filtered into a separate ampule, and pentane (20 mL) was carefully layered onto the filtrate. The vessel was sealed and left to diffuse over a 3 day period, after which the formation yellow crystals was observed. The supernatant was filtered into the initial ampule containing residual undissolved **3'** and the solvent removed. The process was repeated twice more with the undissolved **3'**, adding the subsequent 5 mL solutions of **3'** onto the newly formed crystals and layering with more pentane. The resulting total of yellow crystals was filtered and dried under vacuum, giving **3''** (23 mg, 37%).

^{1}H NMR (500 MHz, 298 K, CD_2Cl_2): δ 7.34-7.25 (m, 15H, -C*Ph$_3$*), 7.07 (at, 6 H, $^3J_{H-H}$ = 7.4 Hz, -C*Ph$_3$*), 6.97 (at, $^3J_{H-H}$ = 7.6 Hz, 12H, -C*Ph$_3$*), 6.85 (d, $^3J_{H-H}$ = 7.9 Hz, 12H, -C*Ph$_3$*). **^{31}P NMR** (162 MHz, 298 K, CD_2Cl_2): δ 100.3 (d, $^1J_{P-P}$ = 162.0 Hz, P^b), -80.8 (t, $^1J_{P-P}$ = 162.0 Hz, P^a). **^{13}C{^{1}H} NMR** (101 MHz, 298 K, CD_2Cl_2): 148.3 (C*Ph$_3$*), 146.0 (C*Ph$_3$*), 131.6 (C*Ph$_3$*), 131.0 (C*Ph$_3$*), 129.6 (C*Ph$_3$*), 128.1 (d, C*Ph$_3$*), 128.0 (C*Ph$_3$*), 127.6 (C*Ph$_3$*), 126.9 (C*Ph$_3$*), 126.6 (C*Ph$_3$*), 126.5 (C*Ph$_3$*). **Melting point:** 276-280 °C (decomp., yellow to brown). **IR:** (ATR, 298 K): 3086 (vw), 3053 (vw), 3028 (vw), 1594 (w), 1489 (s), 1440 (s), 1186 (w), 1163 (w), 1103 (s), 1086 (w), 1033 (s), 1001 (w), 926 (vw), 911 (vw), 878 (vw), 790 (w), 747 (s), 723 (s), 695 (vs), 661 (s), 636 (s), 627 (s), 619 (s). **HRMS** (ESI): 859.2792 m.z [M]$^+$ (calculated: 859.2812).

^{1}H NMR (500 MHz, 298 K, CD_2Cl_2):

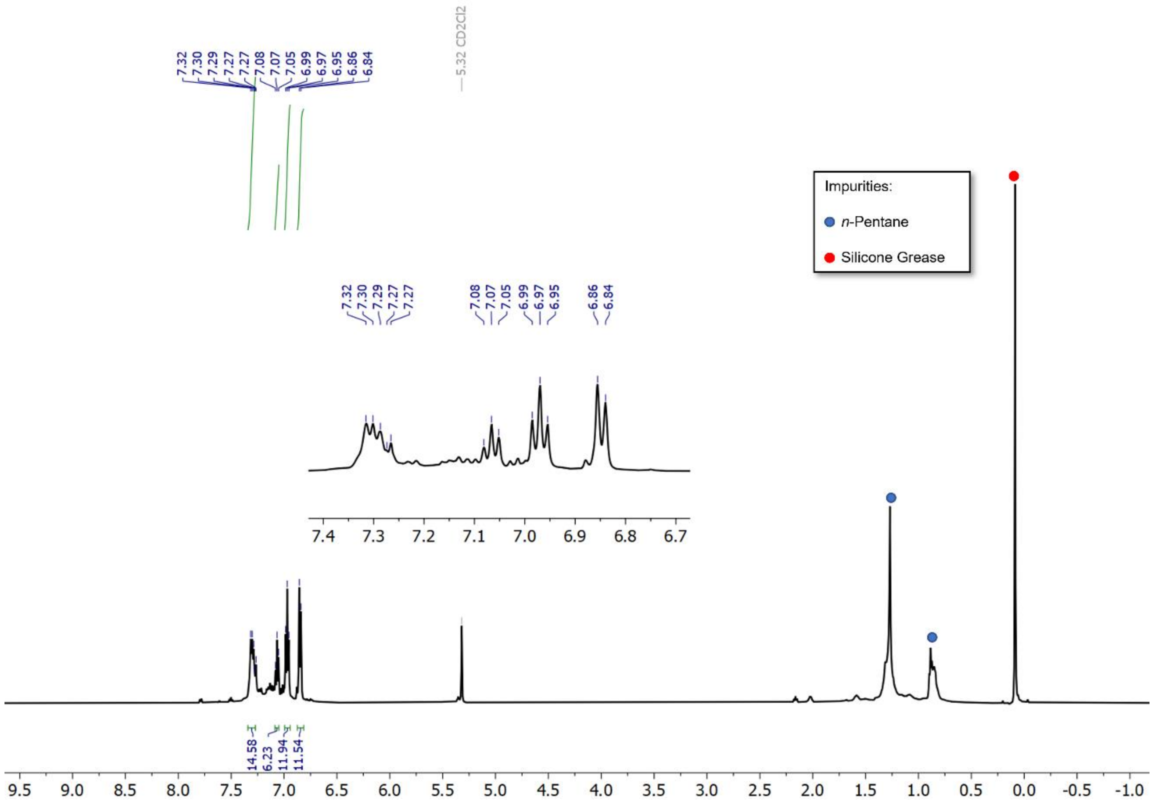

^{31}P NMR (162 MHz, 298 K, CD_2Cl_2):

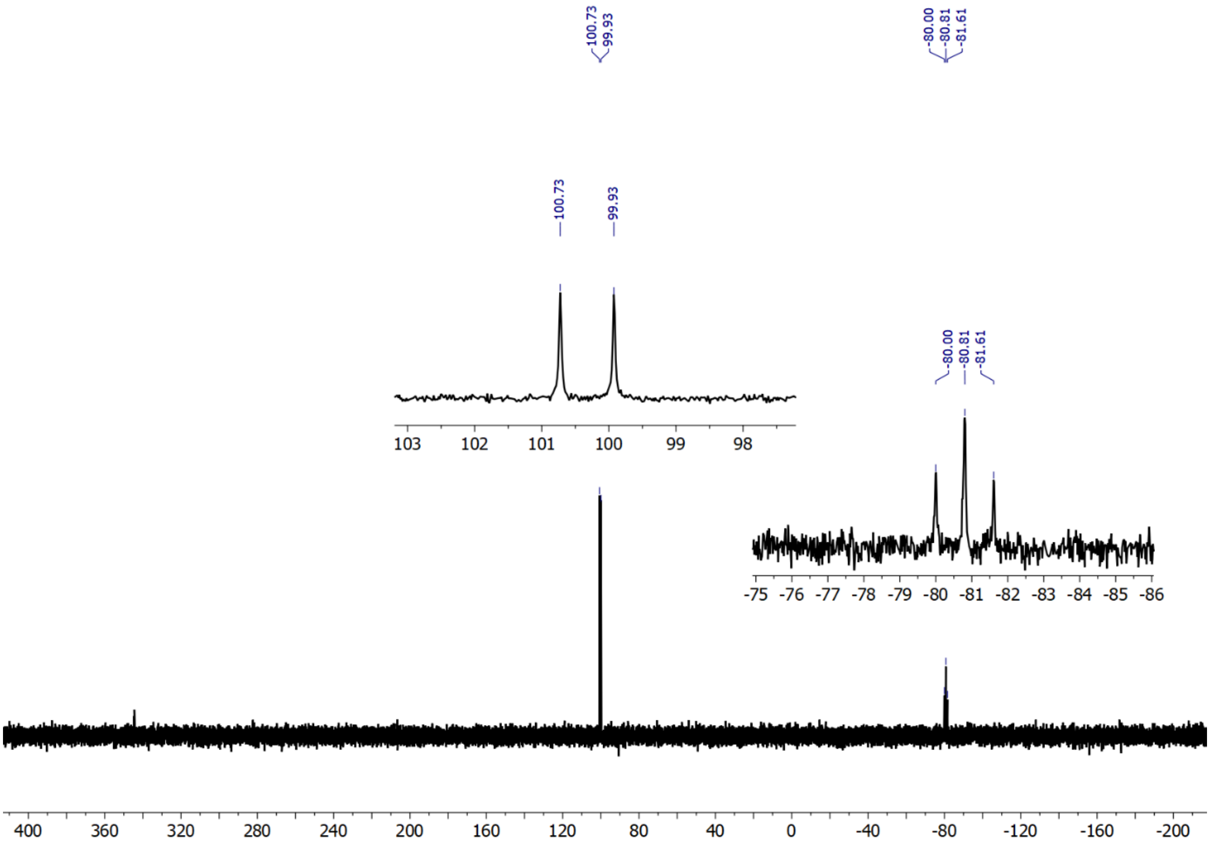

^{13}C{^{1}H} NMR (101 MHz, 298 K, CD_2Cl_2):

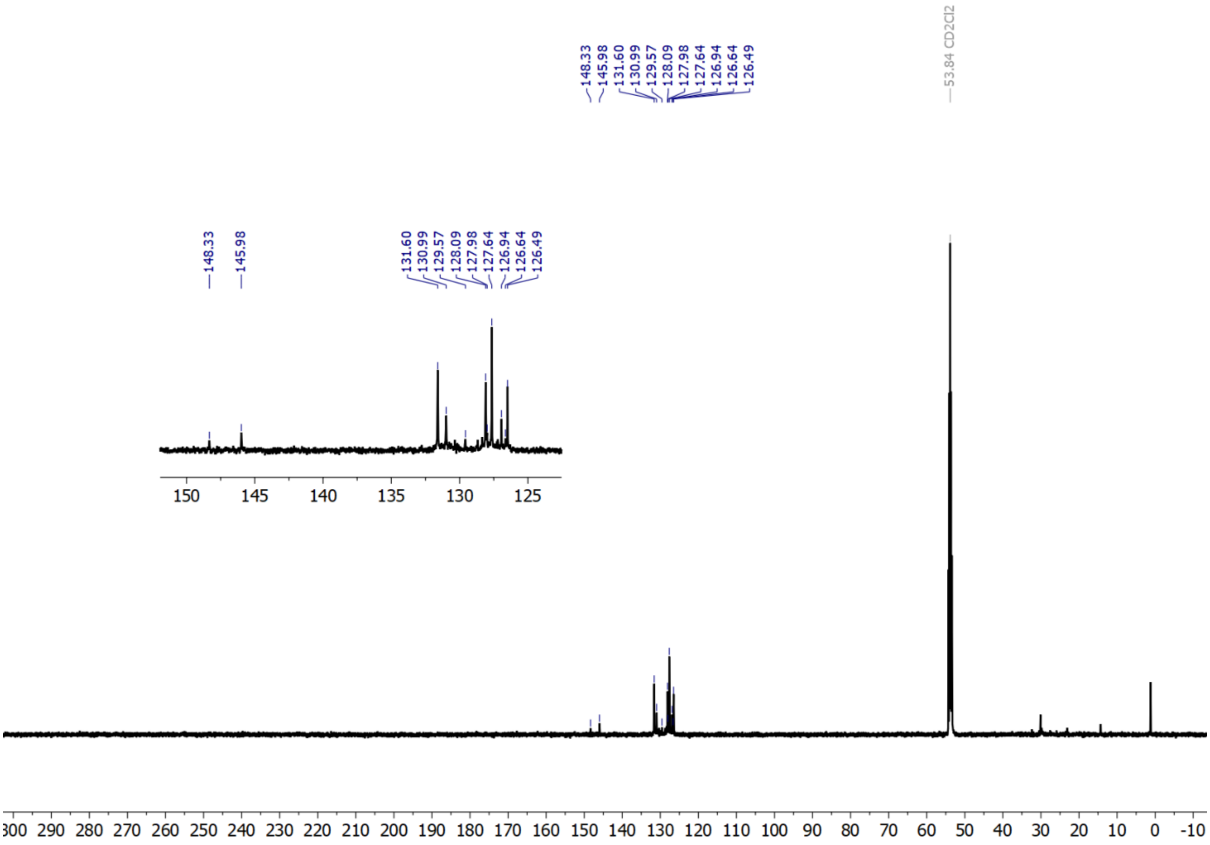

6. Synthesis and Spectroscopic Data for Gold Complexes

ClAu-3

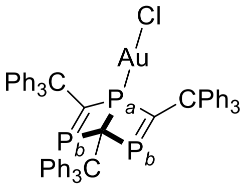

2,4,6-tris(triphenylmethyl)-Dewar-1,3,5-triphosphabenzene (0.05 mmol, 43 mg) and chloro(dimethylsulfide)gold(I) (15 mg, 0.05 mmol) were added to a J-Young's NMR tube along with CD_2Cl_2 (0.6 mL). The reaction was left for 1 h at RT. Crystallisation via a dichloromethane/pentane vapour diffusion gives **ClAu-3** as yellow crystals after 3 days (15 mg, 27%).

^{1}H NMR (500 MHz, 298 K, CD_2Cl_2): δ 7.48-7.32 (m, 8H, -C*Ph₃*), 7.30-7.15 (m, 15H, -C*Ph₃*), 7.11 (t, 6H, $^3J_{H-H}$ = 7.6 Hz, -C*Ph₃*), 6.99 (d, 11H, $^3J_{H-H}$ = 7.6 Hz, -C*Ph₃*). **^{31}P NMR** (162 MHz, 298 K, CD_2Cl_2): δ 381.4 (s, 2P, P_b), 88.3 (s, 1P, P_a). **^{13}C{^{1}H} NMR** (101 MHz, 298 K, CD_2Cl_2): δ 144.5 (-C*Ph₃*), 143.8 (-C*Ph₃*), 130.4 (-C*Ph₃*), 130.1 (-C*Ph₃*), 129.7 (br, -C*Ph₃*), 129.0 (-C*Ph₃*), 128.3 (-C*Ph₃*), 128.1 (-C*Ph₃*), 128.0 (-C*Ph₃*), 127.8 (-C*Ph₃*), 127.8 (-C*Ph₃*), 127.6 (br, -C*Ph₃*), 127.2 (-C*Ph₃*), 67.1 (-*C*Ph₃). **Melting point:** 222-225 °C (orange->back decomp.). **IR:** (ATR, 298 K): 3056 (vw), 2963 (vw), 1594 (vw), 1489 (w), 1441 (w), 1319 (vw), 1260 (w), 1187 (vw), 1157 (vw), 1083 (s), 1014 (s), 913 (w), 875 (w), 798 (vs), 733 (vs), 695 (vs), 666 (s), 642 (s), 625 (s).

^{1}H NMR (500 MHz, 298 K, CD_2Cl_2):

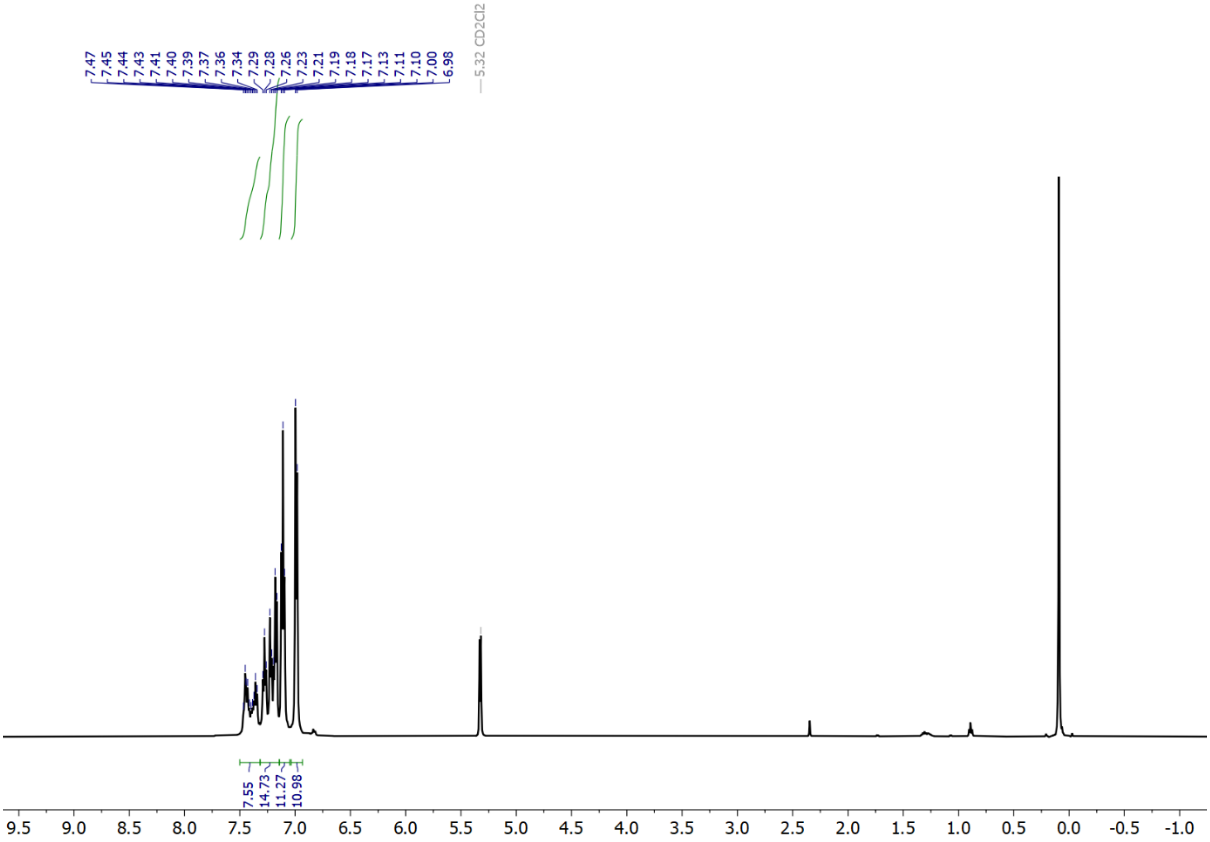

^{31}P NMR (162 MHz, 298 K, CD_2Cl_2):

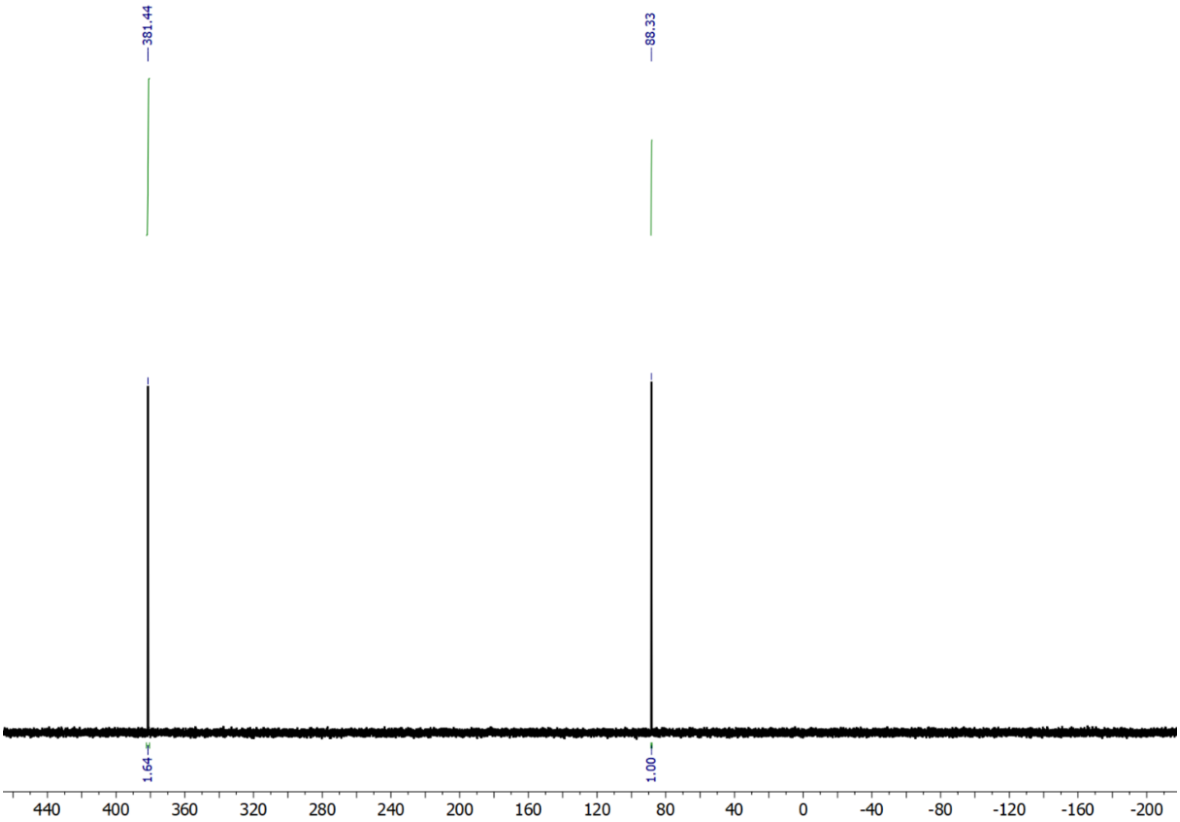

^{13}C{^{1}H} NMR (101 MHz, 298 K, CD_2Cl_2):

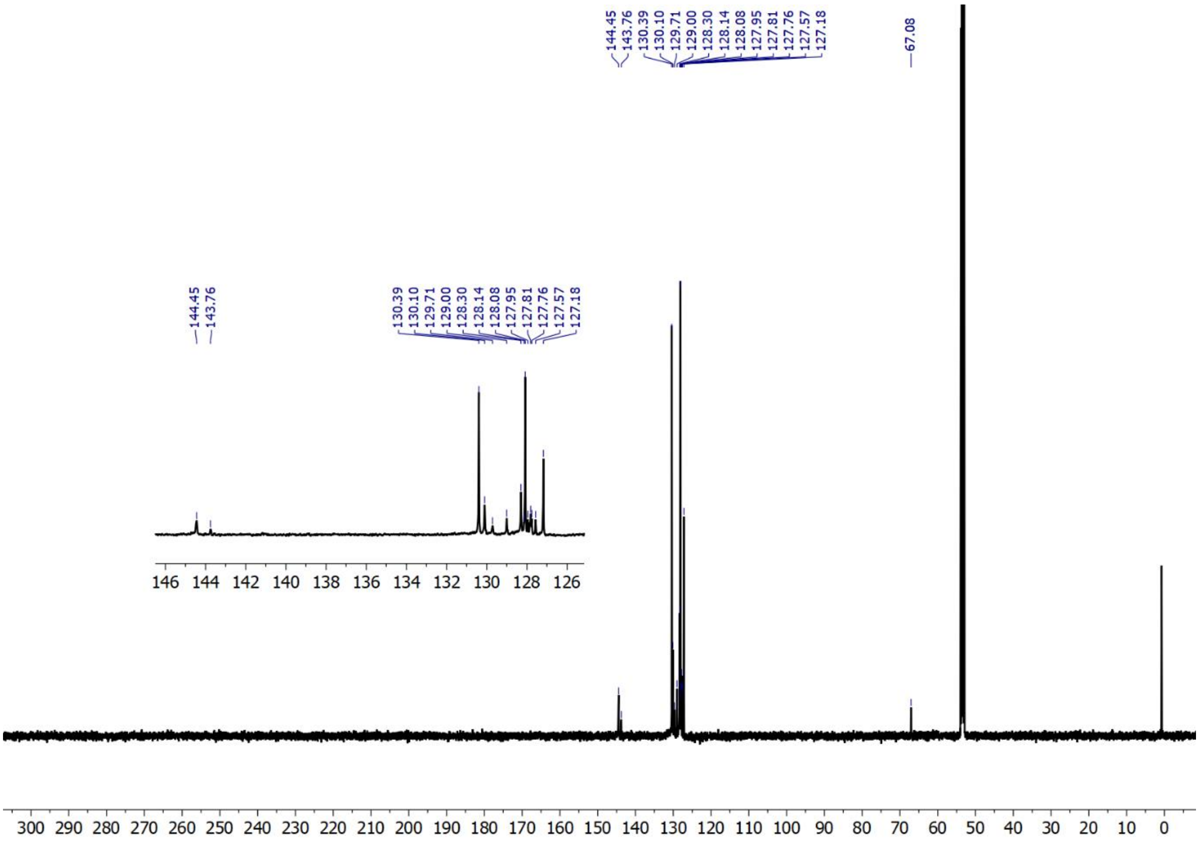

ClAu3’

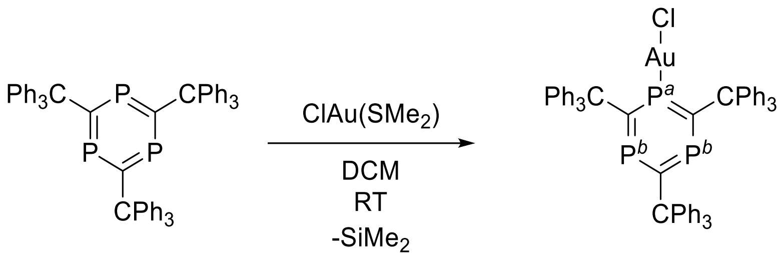

3’ (0.006 mmol, 5 mg) and chloro(dimethylsulfide)gold(I) (1.8 mg, 0.006 mmol) were added to a J-Young’s NMR tube along with CD_2Cl_2 (0.6 mL). The reaction was left for 1 h at RT. Analysis by ^{31}P NMR spectroscopy revealed complete conversion of **3’** to new signals at δ 280.2 (d, 2P, $^2J_{P\text{-}P}$ = 36.5 Hz, P^b) and 217.9 (t, 1P, $^2J_{P\text{-}P}$ = 36.4 Hz, P^a) (see spectrum below). 95% conversion to **ClAu-3’** was calculated by ^{31}P NMR integrals.

^{31}P NMR (162 MHz, 298 K, CD_2Cl_2):

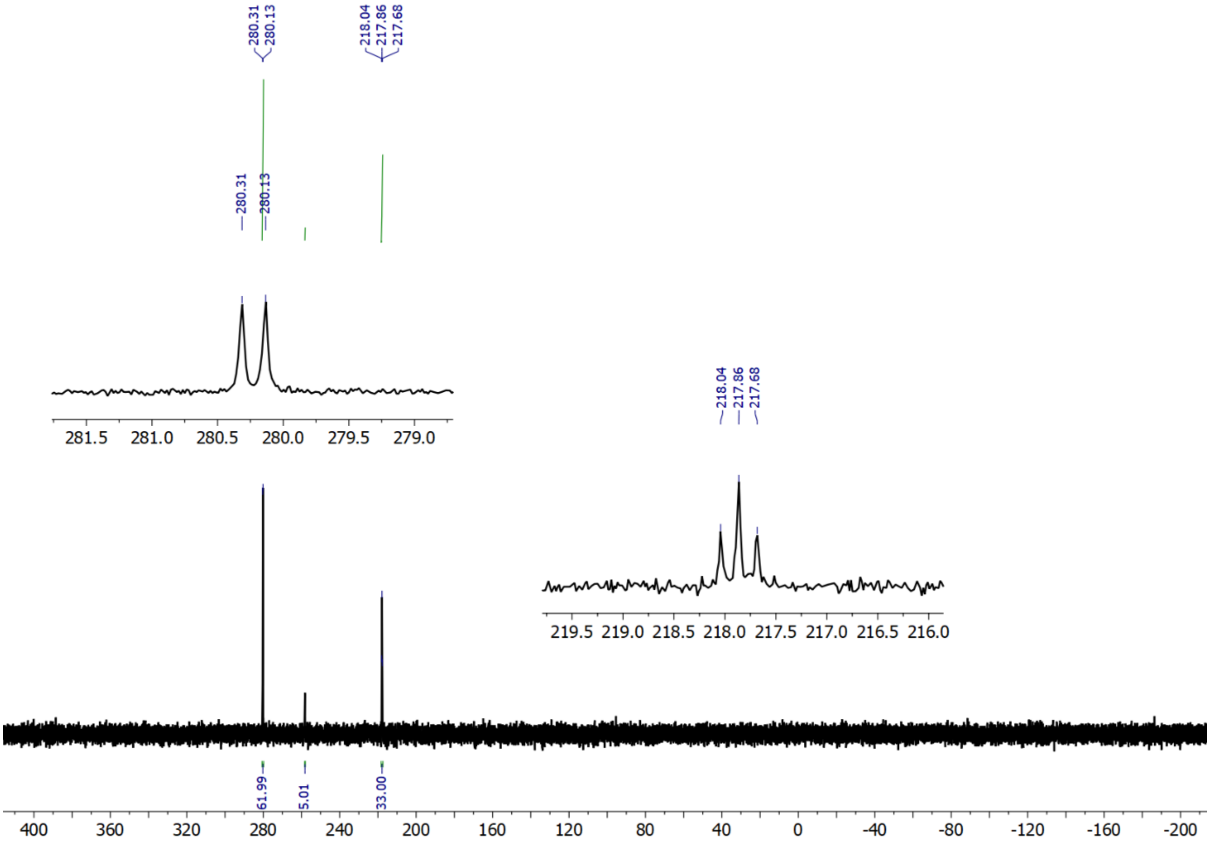

ClAu3''

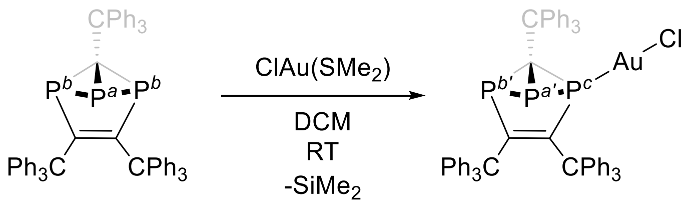

3'' (0.006 mmol, 5 mg) and chloro(dimethylsulfide)gold(I) (1.8 mg, 0.006 mmol) were added to a J-Young's NMR tube along with CD_2Cl_2 (0.6 mL). The reaction was left for 1 h at RT and a yellow->orange colour change was observed. Analysis by ^{31}P NMR spectroscopy revealed complete conversion of **3''** to new signals at δ 65.4 (d, 1P, $^1J_{P\text{-}P}$ = 207.2 Hz, $P^{b'}$), 8.0 (d, 1P, $^1J_{P\text{-}P}$ = 162.2 Hz, P^c) and -89.8 (dd, 1P, $^1J_{P\text{-}P}$ = 157.0 Hz, $^1J_{P\text{-}P}$ = 205.3 Hz, $P^{a'}$) (see spectrum below).

In comparison to the ^{31}P NMR signals of **3''** (δ 100.3 (d, $^1J_{P\text{-}P}$ = 162.0 Hz, P^b), -80.8 (t, $^1J_{P\text{-}P}$ = 162.0 Hz, P^a), the signal shifts observed suggest de-symmetrisation of the non-apical P^b environments, alluding to coordination of AuCl at either one of these positions. This is supported by DFT, whereby coordination at the non-apical P site (P^b) is found to be energetically favourable relative to coordination at the apical site (P^a) (see computational SI).

^{31}P NMR (162 MHz, 298 K, CD_2Cl_2):

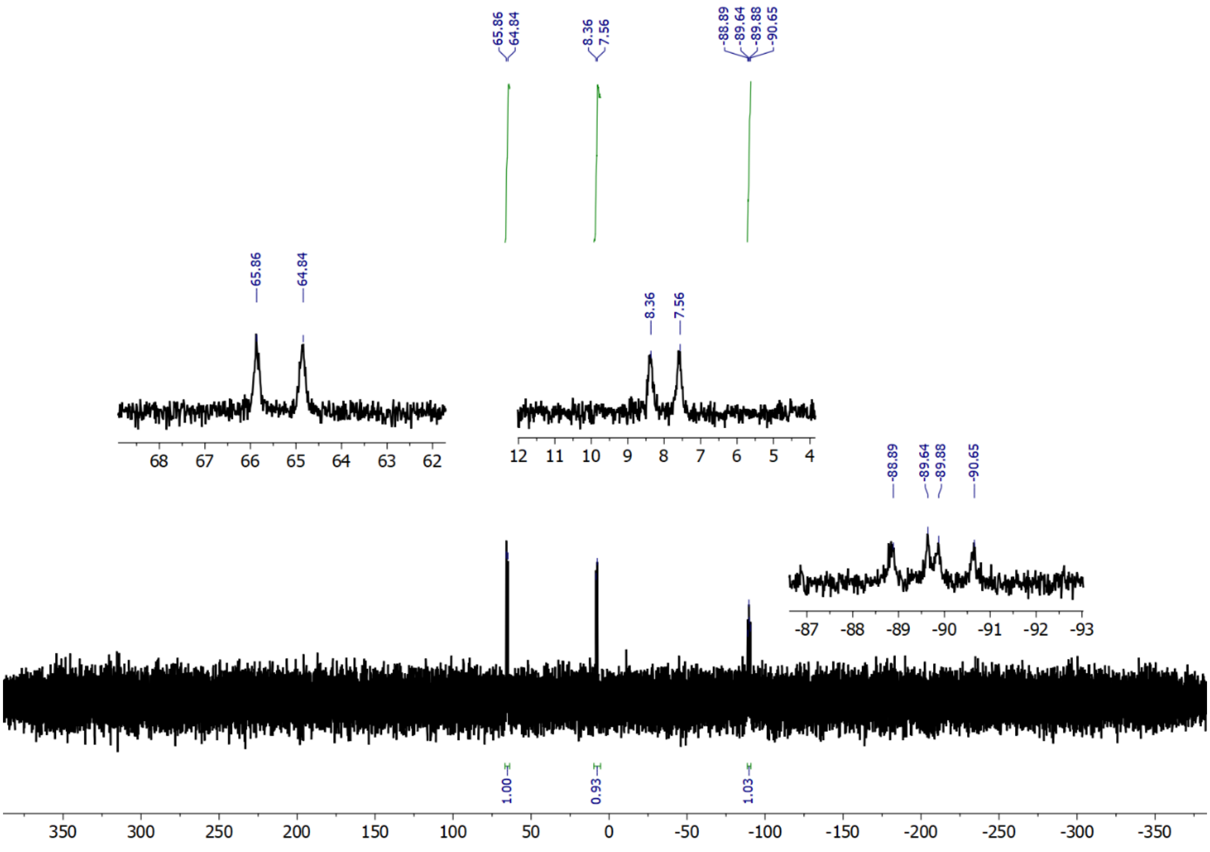

7. Synthesis and Characterisation Data Scheme 4

4a

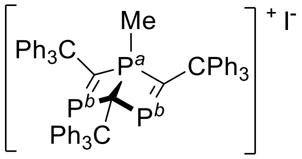

2,4,6-tris(triphenylmethyl)-Dewar-1,3,5-triphosphabenzene (0.05 mmol, 43 mg) was added to a J-Young's NMR tube along with C_6D_6 (0.6 mL). Iodomethane (6.22 µL, 0.1 mmol) was then added. The reaction was then heated to 80 °C, upon which the colour of the solution changed from orange to dark red. The reaction was left at this temperature for 3 days, before being cooled to room temperature. Volatiles were removed in vacuo to give a dark red solid. The crude product was crystalised via a dichloromethane/pentane vapour diffusion to give **4a** as red crystals (35 mg, 70%).

^{1}H NMR (500 MHz, 298 K, CD_2Cl_2): δ 7.34-7.74 (m, 10H, -C*Ph$_3$*), 7.21-7.05 (m, 30H, -C*Ph$_3$*), 7.03-6.73 (m (br), 5H), -0.12 (d, 3H, $^2J_{H\text{-}P}$ = 12.1 Hz, P-*Me*). **^{31}P NMR** (162 MHz, 298 K, CD_2Cl_2): δ 298.2.(s, 2P, P$_b$), 64.6 (q, 1P, $^2J_{P\text{-}H}$ = 12.1 Hz, P$_a$). **^{13}C{^{1}H} NMR** (101 MHz, 298 K, CD_2Cl_2): 146.2 (t, $^3J_{C\text{-}P}$ = 5.8 Hz, C*Ph$_3$*), 144.4 (C*Ph$_3$*), 131.6 (br, C*Ph$_3$*), 131.0 (C*Ph$_3$*), 129.7 (C*Ph$_3$*), 128.7 (d, $^3J_{C\text{-}P}$ = 3.9 Hz, C*Ph$_3$*), 128.3 (C*Ph$_3$*), 128.3 (C*Ph$_3$*), 128.1 (C*Ph$_3$*), 127.1 (C*Ph$_3$*), 126.7 (C*Ph$_3$*), 64.0 (m, P$_b$-*C*-P$_b$) 63.4 (-*C*Ph$_3$), 12.8 (d, $^1J_{C\text{-}P}$ = 22.1 Hz, -P-*Me*). **Melting point:** 268-272 °C (orange -> dark brown decomp.). **IR:** (ATR, 298 K): 3055 (vw), 3021 (vw), 1594 (vw), 1489 (w), 1442 (w), 1395 (vw), 1281 (vw), 1261 (vw), 1187 (vw), 1157 (vw), 1083 (vw), 1034 (vw), 1014 (w), 1002 (w), 974 (w), 952 (w), 916 (w),899 (w), 843 (w), 737 (s), 695 (vs), 639 (s), 624 (w). **HRMS** (ESI): 873.2979 m.z [M]$^+$ (calculated: 873.2969).

^{1}H NMR (500 MHz, 298 K, CD_2Cl_2):

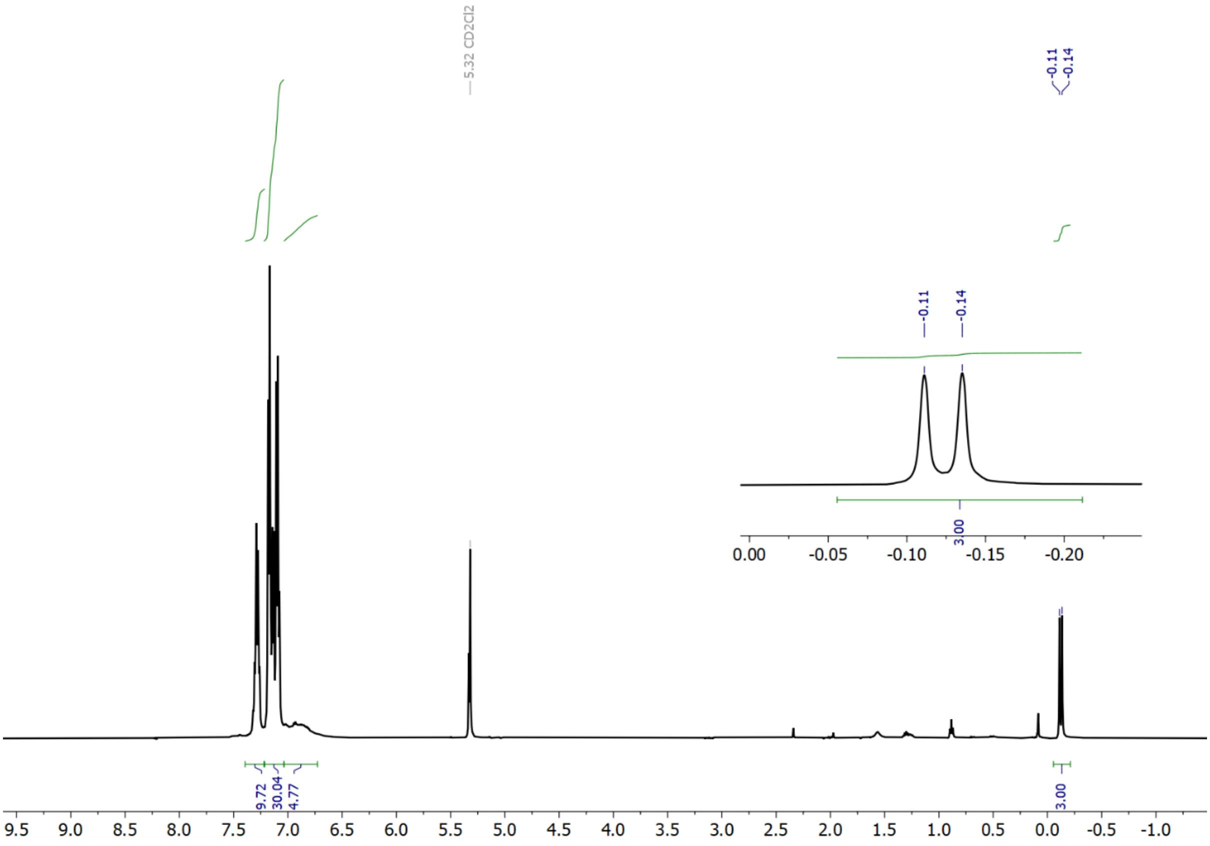

^{31}**P NMR** (162 MHz, 298 K, CD_2Cl_2):

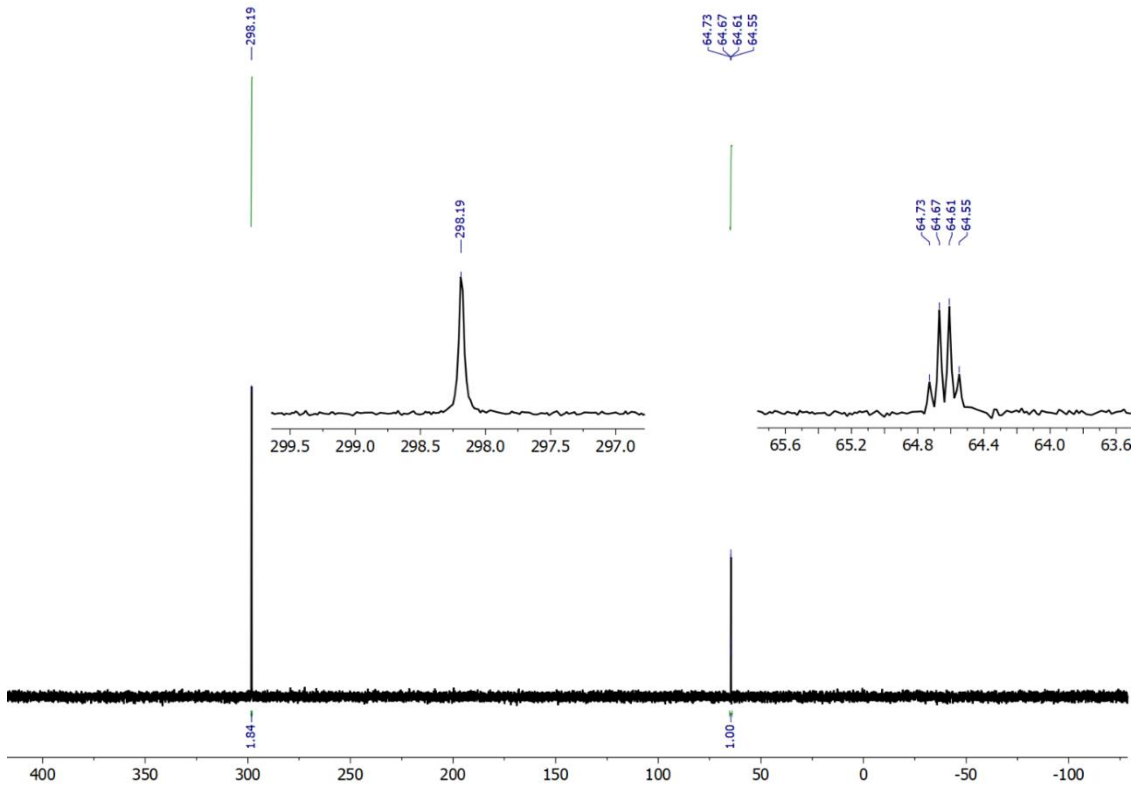

^{13}**C{^{1}H} NMR** (101 MHz, 298 K):

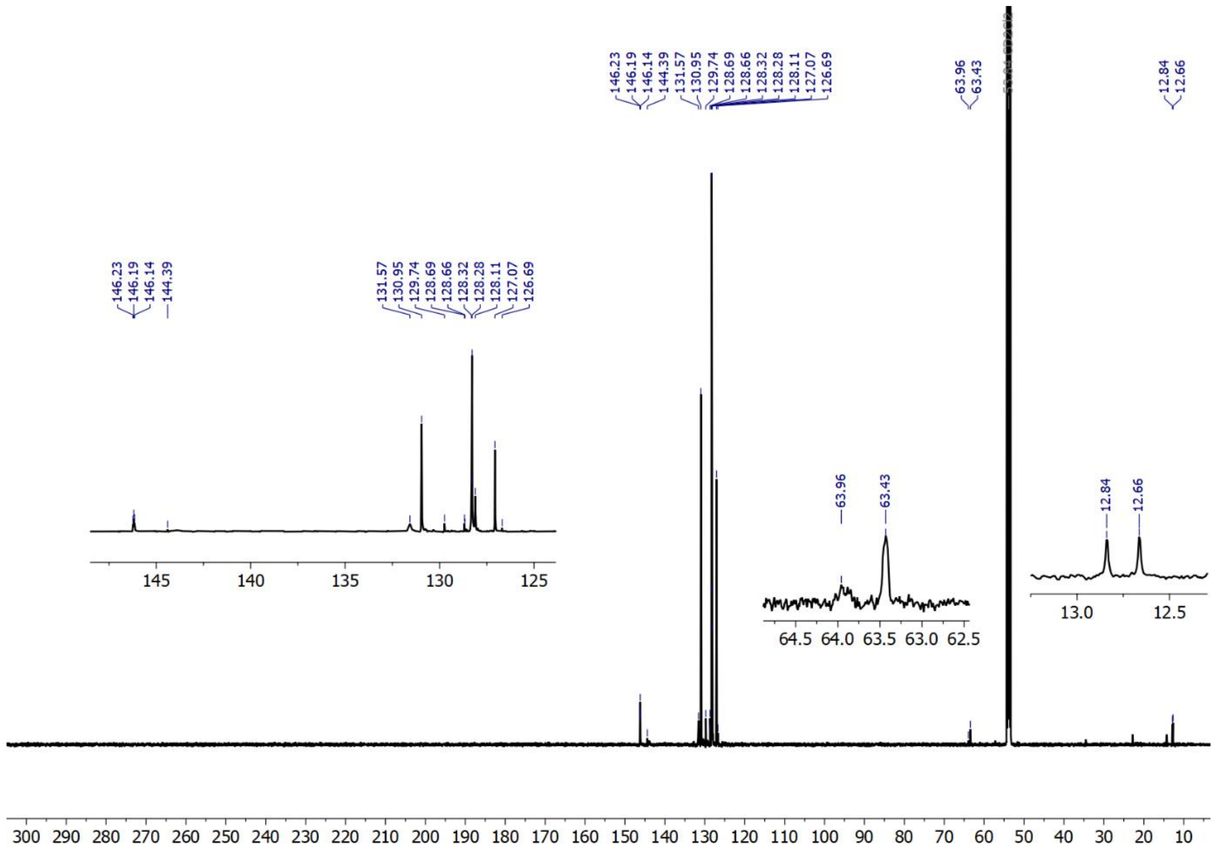

4b

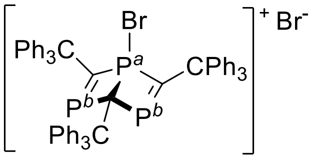

2,4,6-tris(triphenylmethyl)-Dewar-1,3,5-triphosphabenzene (0.05 mmol, 43 mg) was added to a J-Young's NMR tube along with C_6D_6 (0.6 mL). Tetrabromomethane (24.9 mg, 0.075 mmol) was then added. The reaction was then heated to 80 °C, upon which the colour of the solution changed from orange to dark red. The reaction was left at this temperature for 1 hour, before being cooled to room temperature. Volatiles were removed in vacuo to give a dark red solid. The crude product was crystalised via a dichloromethane/pentane vapour diffusion to give **4b** as red crystals (18 mg, 36%).

^{1}H NMR (500 MHz, 298 K, CD_2Cl_2): δ 7.39-7.23 (m, 12H, -C*Ph$_3$*), 7.19-7.05 (m, 27H, -C*Ph$_3$*), 7.00-6.89 (m, 6H, -C*Ph$_3$*). **^{31}P NMR** (162 MHz, 298 K, CD_2Cl_2): δ 294.2 (d, 2P, $^2J_{P\text{-}P}$ = 24.0 Hz, P$_b$), 28.5 (t, 1P, $^2J_{P\text{-}P}$ = 24.0 Hz, P$_a$). **^{13}C{^{1}H} NMR** (101 MHz, 298 K, CD_2Cl_2): 144.8 (t, $^3J_{C\text{-}P}$ = 6.7 Hz, -C*Ph$_3$*), 142.7 (-C*Ph$_3$*), 131.5 (br, -C*Ph$_3$*), 130.0 (-C*Ph$_3$*), 130.4 (-C*Ph$_3$*), 128.6 (-C*Ph$_3$*), 128.2 (-C*Ph$_3$*), 128.1 (-C*Ph$_3$*), 127.9 (-C*Ph$_3$*), 127.7 (-C*Ph$_3$*), 127.1 (-C*Ph$_3$*), 64.5 (t, $^3J_{C\text{-}P}$ = 4.6 Hz, -*C*Ph$_3$). **Melting point:** 170-174 °C (orange -> dark red decomp.). **IR:** (ATR, 298 K): 3055 (vw), 3019 (vw), 1595 (vw), 1490 (w), 1442 (w), 1262 (vw), 1188 (vw), 1158 (vw), 1083 (vw), 1033 (vw), 1002 (vw), 964 (vw), 952 (w), 912 (w), 821 (w), 737 (s), 695 (vs), 639 (s), 625 (w), 588 (w). **HRMS** (ESI): 960.1855 [M+Na]$^+$ (calculated: 960.1815).

^{1}H NMR (500 MHz, 298 K, CD_2Cl_2):

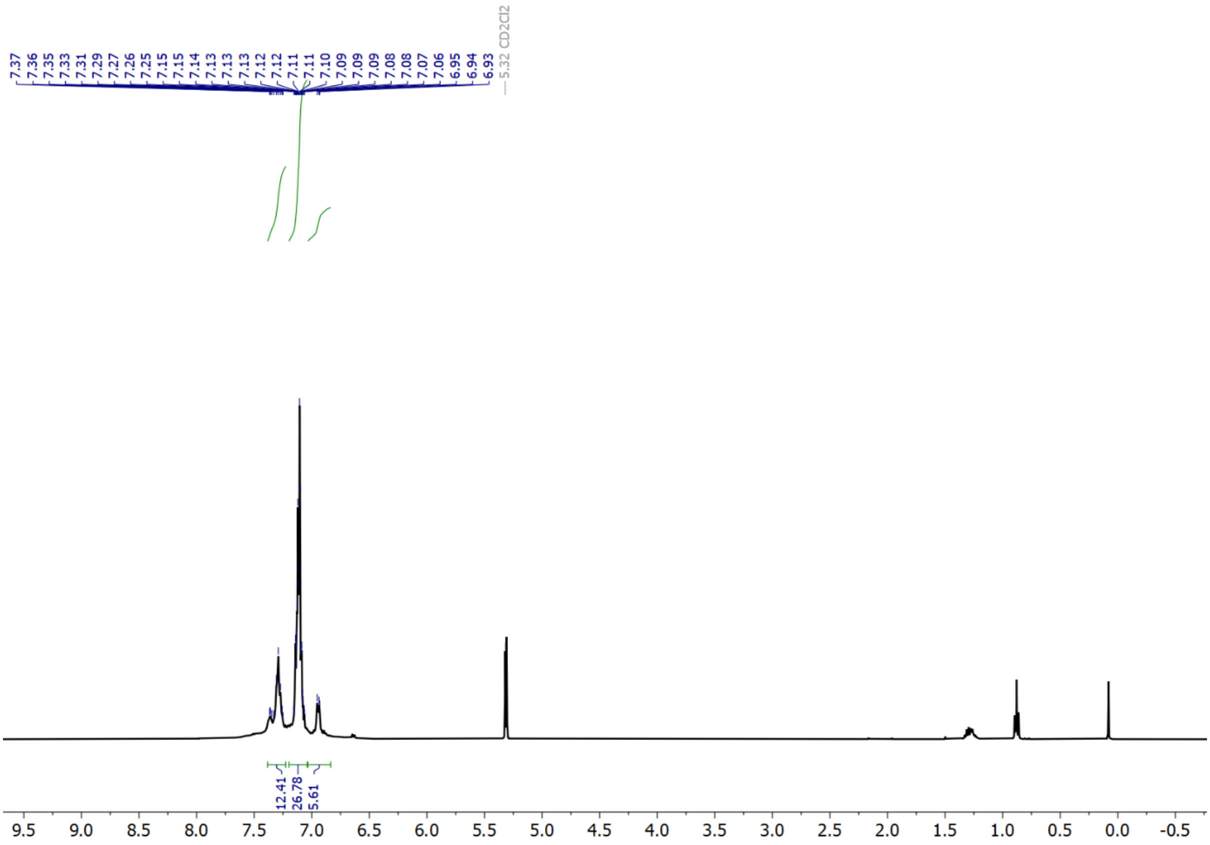

^{31}P NMR (162 MHz, 298 K, CD_2Cl_2):

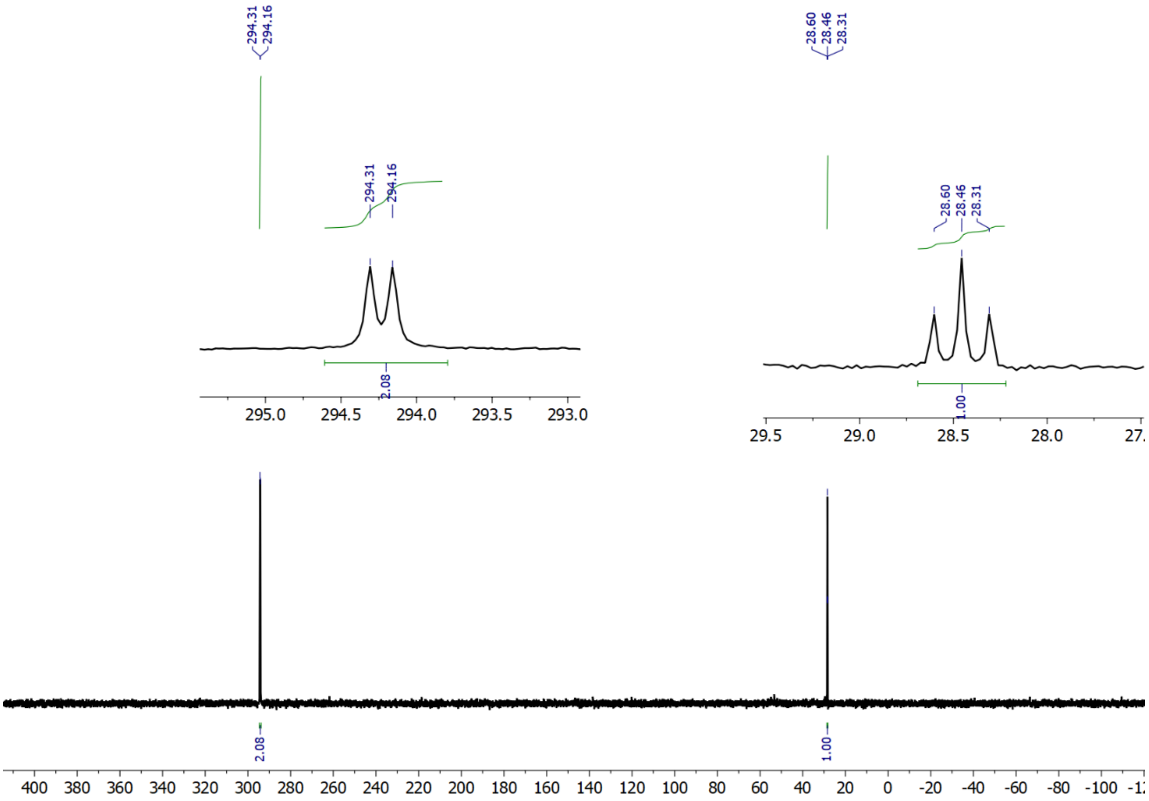

^{13}C{^{1}H} NMR (101 MHz, 298 K, CD_2Cl_2):

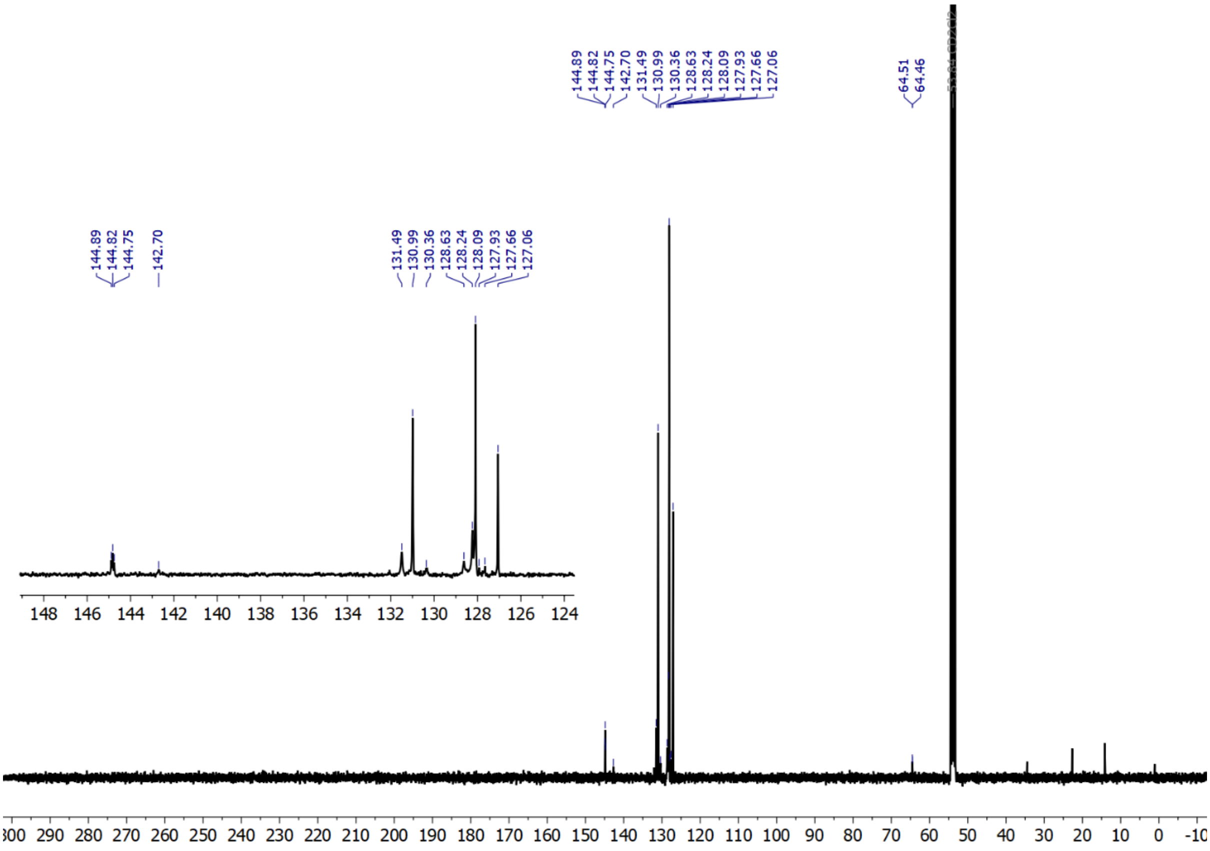

4c

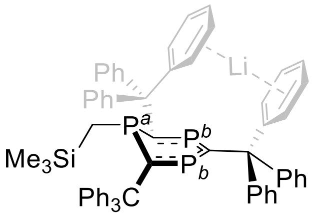

2,4,6-tris(triphenylmethyl)-Dewar-1,3,5-triphosphabenzene (0.05 mmol, 43 mg) was added to a J-Young's NMR tube along with C_6D_6 (0.6 mL). $LiCH_2TMS$ (4.7 mg, 0.05 mmol) was then added and an orange to dark purple colour change was observed. Characterised by in-situ NMR, not isolated. Spectroscopic yield: >99%.

^{1}H NMR (500 MHz, 298 K, C_6D_6): δ 7.68 (d, 6H, $^3J_{H\text{-}H}$ = 7.8 Hz, -C*Ph*$_3$), 7.54 (d, 6H, $^3J_{H\text{-}H}$ = 7.8 Hz, -C*Ph*$_3$), 6.98-6.90 (m, 18H, -C*Ph*$_3$), 6.89-6.80 (m, 9H, -C*Ph*$_3$), -0.01 (s, 2H, -C*H*$_2$SiMe$_3$), -0.20 (s, 9H, Si*Me*$_3$). **^{31}P NMR** (162 MHz, 298 K, C_6D_6): δ 239.3 (s (br), 2P, P_b), -73.9 (s, 1P, P_a).

^{1}H NMR (500 MHz, 298 K, C_6D_6):

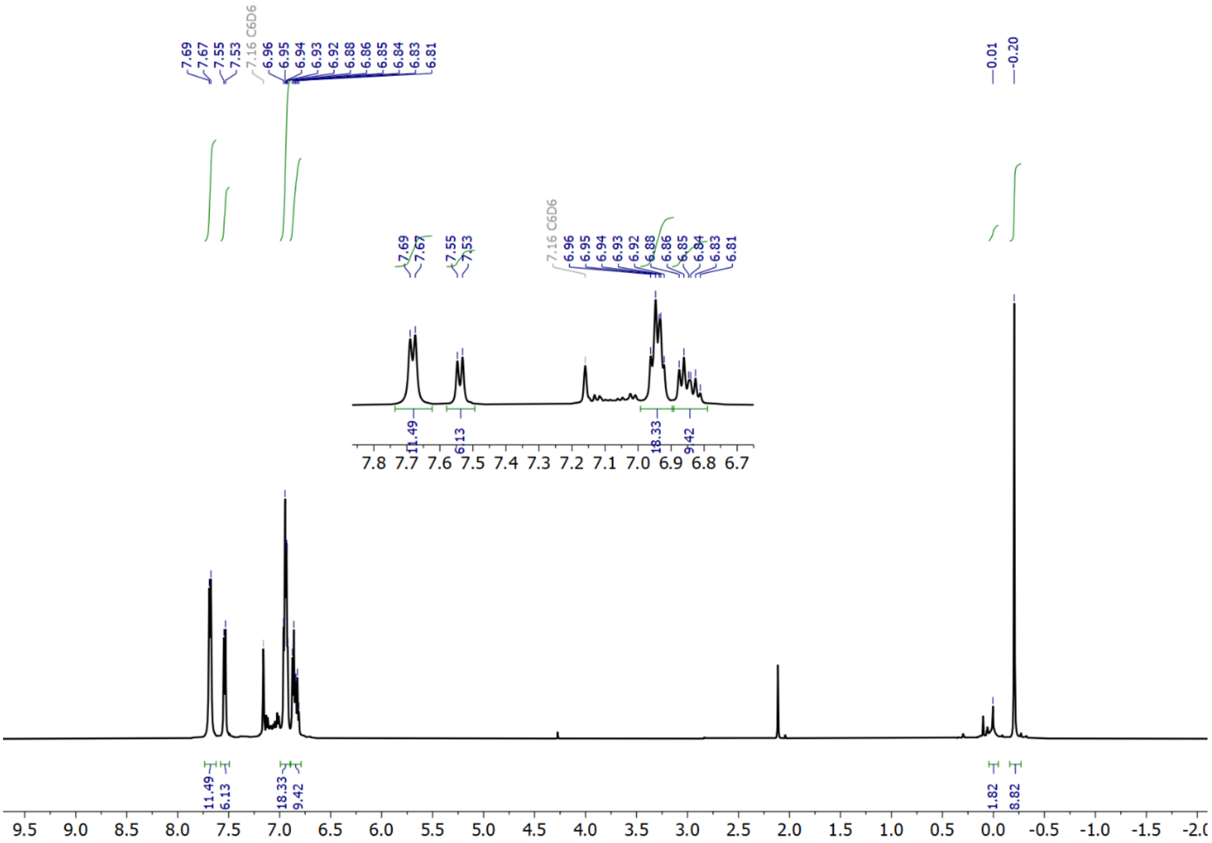

^{31}P NMR (162 MHz, 298 K, C_6D_6):

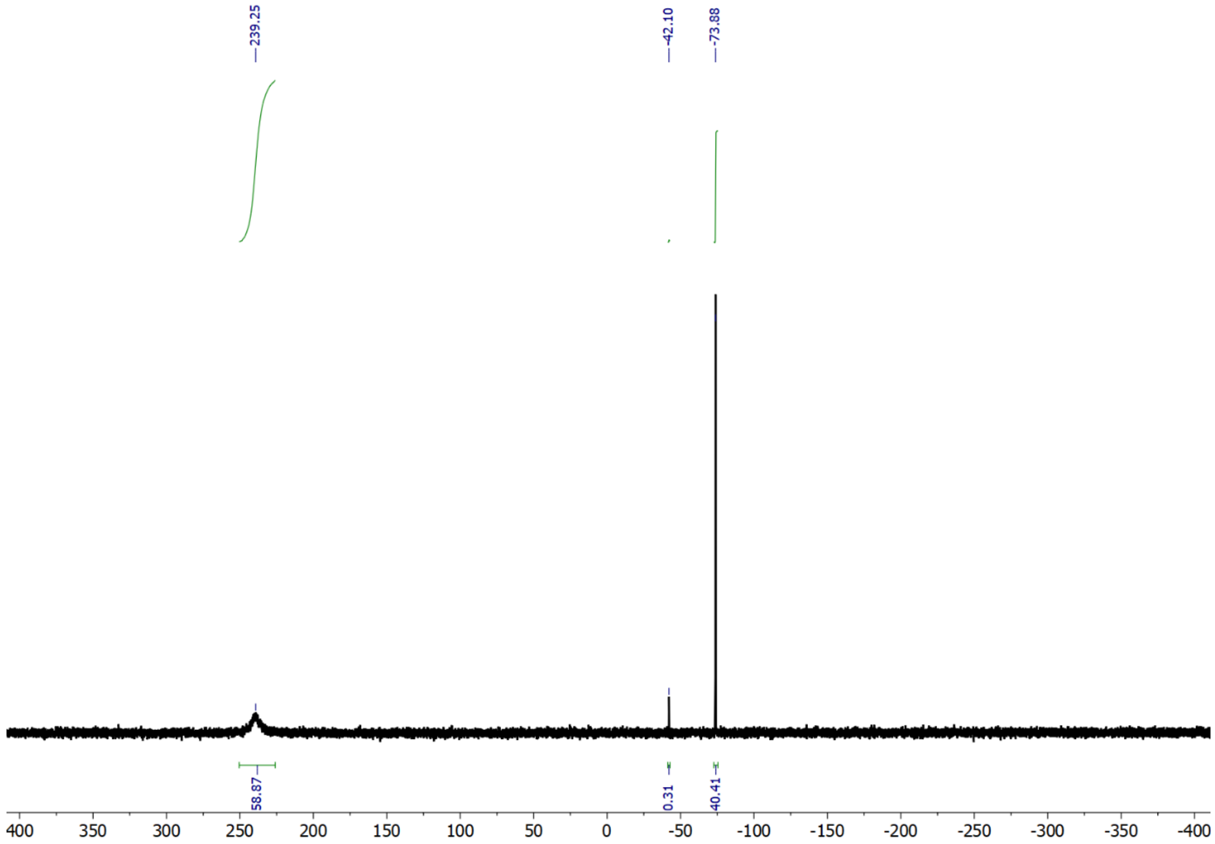

4d

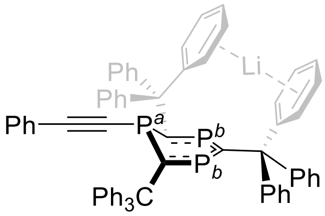

Phenylacetylene (109.8 µL, 1.0 mmol) was added to a small J-Young's Schlenk tube alongside THF (2 mL). The solution was cooled to -78 °C before *n*-butyllithium (0.44 mL, 2.5 M, 1.1 mmol) was added dropwise. The solution was stirred at this temperature for 30 mins, before being warmed to room temperature and stirred for a further 30 mins. A 100 µL aliquot of this reaction was taken and added to a J-Young's NMR tube containing 2,4,6-tris(triphenylmethyl)-Dewar-1,3,5-triphosphabenzene (0.05 mmol, 43 mg) and C_6D_6 (0.6 mL). An immediate dark purple colour change was observed. Characterised by in-situ NMR, <u>not isolated.</u> Spectroscopic yield: 77%.

^{1}H NMR (500 MHz, 298 K, C_6D_6): δ 7.84 (d, 9H, $^3J_{H-H}$ = 7.7 Hz, -C*Ph*$_3$), 7.58 (d, 6H, $^3J_{H-H}$ = 7.8 Hz, -C*Ph*$_3$), 7.23-7.18 (m, 6H, -C*Ph*$_3$), 7.16-7.01 (m, 11H, -C*Ph*$_3$, -CC*Ph*), 6.95-6.88 (m, 12H, -C*Ph*$_3$), 6.85 (t, $^3J_{H-H}$ = 7.2 Hz, -C*Ph*$_3$). **^{31}P NMR** (162 MHz, 298 K, C_6D_6): δ 232.2 (d, 2P, $^2J_{P-P}$ = 24.4 Hz, P$_b$), 122.9 (t, 1P, $^2J_{P-P}$ = 24.4 Hz, P$_a$).

^{1}H NMR (500 MHz, 298 K, C_6D_6):

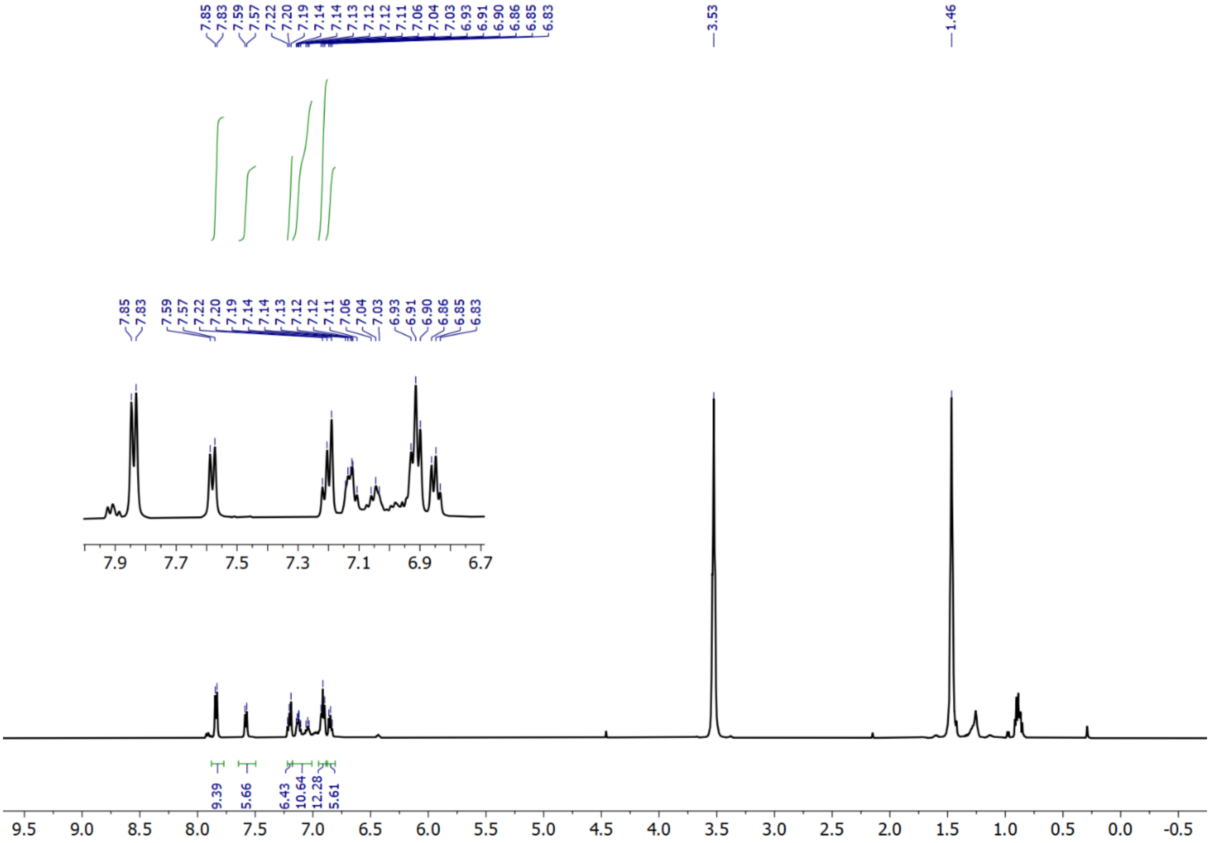

^{31}**P NMR** (162 MHz, 298 K, C_6D_6):

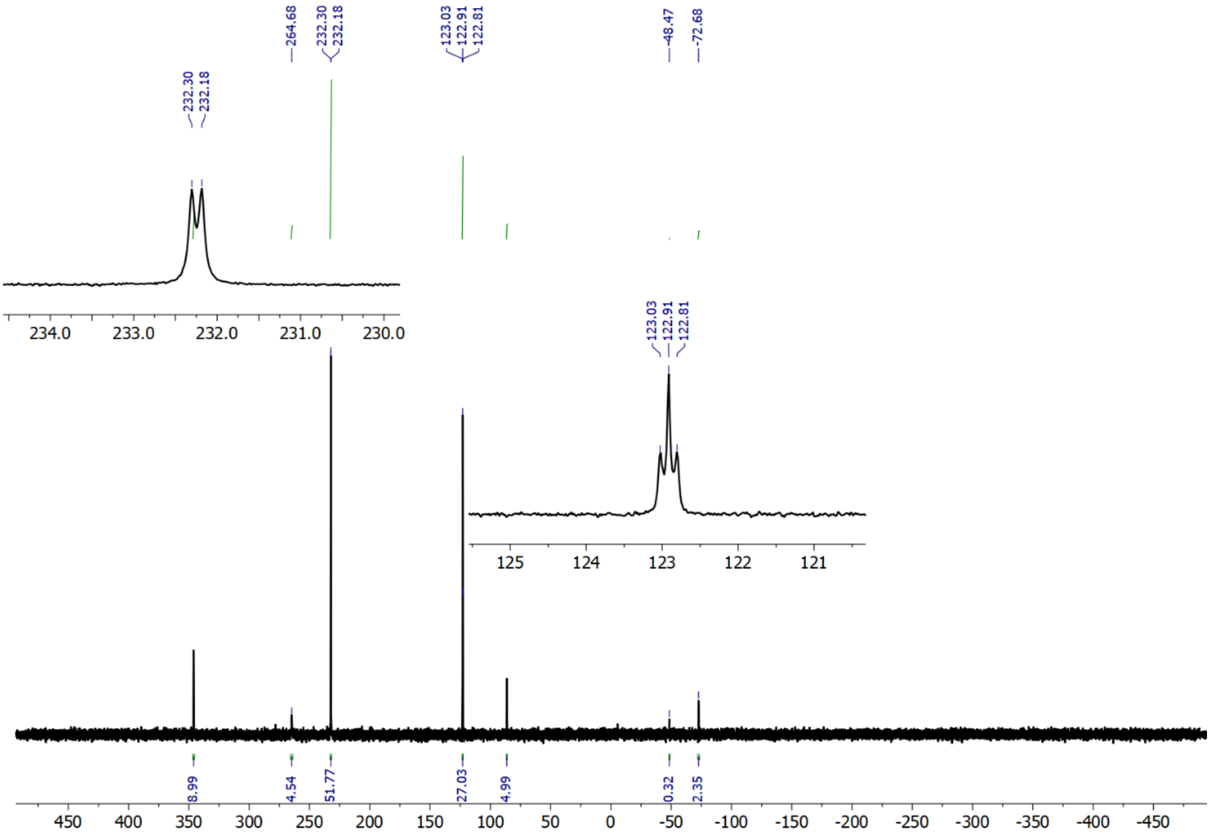

4e

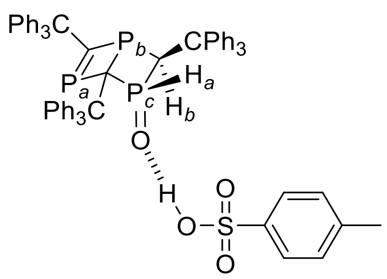

2,4,6-tris(triphenylmethyl)-Dewar-1,3,5-triphosphabenzene (0.05 mmol, 43 mg) was added to a J-Young's NMR tube along with C_6D_6 (0.6 mL). *p*-Toluenesulfonic acid monohydrate (19 mg, 0.1 mmol) was then added. The reaction was then heated to 80 °C, upon which the colour of the solution changed from orange to yellow. The reaction was left at this temperature for 18 hours, before being cooled to room temperature. The reaction was filtered to remove any unreacted *p*-Toluenesulfonic acid monohydrate before the volatiles were removed in vacuo to give a yellow solid. The crude product was dissolved in a minimum amount of dichloromethane, and a few drops of pentane were added. The solution was left to crystallise overnight at -30 °C, giving **4b** as pale yellow microcrystals (36 mg, 69%).

^{1}H NMR (500 MHz, 298 K, C_6D_6): δ 13.81 (s (br), 1H, O-*H*), 8.08 (d, 2H, $^3J_{H-H}$ = 7.8 Hz, *p*TSA-Ar-*H*), 7.45 (s (br), 6H, -C*Ph₃*), 7.40 (dd, 1H, $^1J_{P-H}$ = 516.6 Hz, $^3J_{H-H}$ = 4.7 Hz, *H$_a$*) 7.07-6.98 (m, 18H, -C*Ph$_3$*), 6.95-6.83 (m, 21H, -C*Ph$_3$*), 6.73 (d, 2H, $^3J_{H-H}$ = 7.8 Hz, *p*TSA-Ar-*H*), 5.09 (dd, 1H, $^2J_{H-P}$ = 24.4 Hz, $^3J_{H-H}$ = 4.7 Hz, *H$_b$*), 1.87 (s, 3H, *p*TSA-C*H$_3$*). **^{31}P NMR** (162 MHz, 298 K, C_6D_6): δ 389.0 (d, 1P, $^2J_{P-P}$ = 29.7 Hz, P$_a$), 23.3 (at, 1P, $^2J_{P-P}$ = 26.9 Hz, P$_b$), 13.6 (adt, 1P, $^1J_{P-H}$ = 516.6 Hz, $^2J_{P-P}$ = $^2J_{P-H}$ = 24.4 Hz, P$_c$). **^{13}C{^{1}H} NMR** (101 MHz, 298 K, C_6D_6): δ 220.4 (P=*C*), 145.8 (d, $^3J_{P-P}$ = 6.0 Hz, -C*Ph$_3$*), 144.2 (-C*Ph$_3$*), 142.9 (-C*Ph$_3$*), 142.6 (*p*-TSA Ar-*C*), 141.5 (-C*Ph$_3$*), 139.2 (Ar-*C* *p*-TSA), 131.5 (d, $^3J_{P-P}$ = 3.5 Hz, -C*Ph$_3$*), 130.9 (-C*Ph$_3$*), 129.9 (d, $^3J_{P-P}$ = 3.2 Hz, -C*Ph$_3$*), 129.5 (-C*Ph$_3$*), 128.8 (*p*-TSA Ar-*C*), 128.4 (-C*Ph$_3$*), 127.5 (*p*-TSA Ar-*C*), 127.4 (-C*Ph$_3$*), 127.3 (-C*Ph$_3$*), 127. 2(-C*Ph$_3$*), 68.2 (*C*Ph$_3$), 63.2 (*C*Ph$_3$), (59.9 (d, $^2J_{P-P}$ = 9.6 Hz, -*C*Ph$_3$), 54.4 (m, P$_b$-*C*(H$_a$)-P$_c$), 40.2 (m, P$_a$-*C*-P$_c$), 21.2 (*p*-TSA -*Me*). **Melting point:** 188-192 °C. **IR:** (ATR, 298 K): 3055 (vw), 1596 (w), 1491 (s), 1443 (s), 1344 (w), 1176 (s), 1086 (s), 1034 (s), 1002 (s), 901 (s), 813 (w), 786 (s), 748 (s), 699 (vs), 668 (vs). **Elemental analysis:** calcd. for $C_{67}H_{55}O_4P_3S$: C: 76.70%, H: 5.28%; found: C: 76.80, H: 5.21%.

^{1}H NMR (500 MHz, 298 K, C_6D_6):

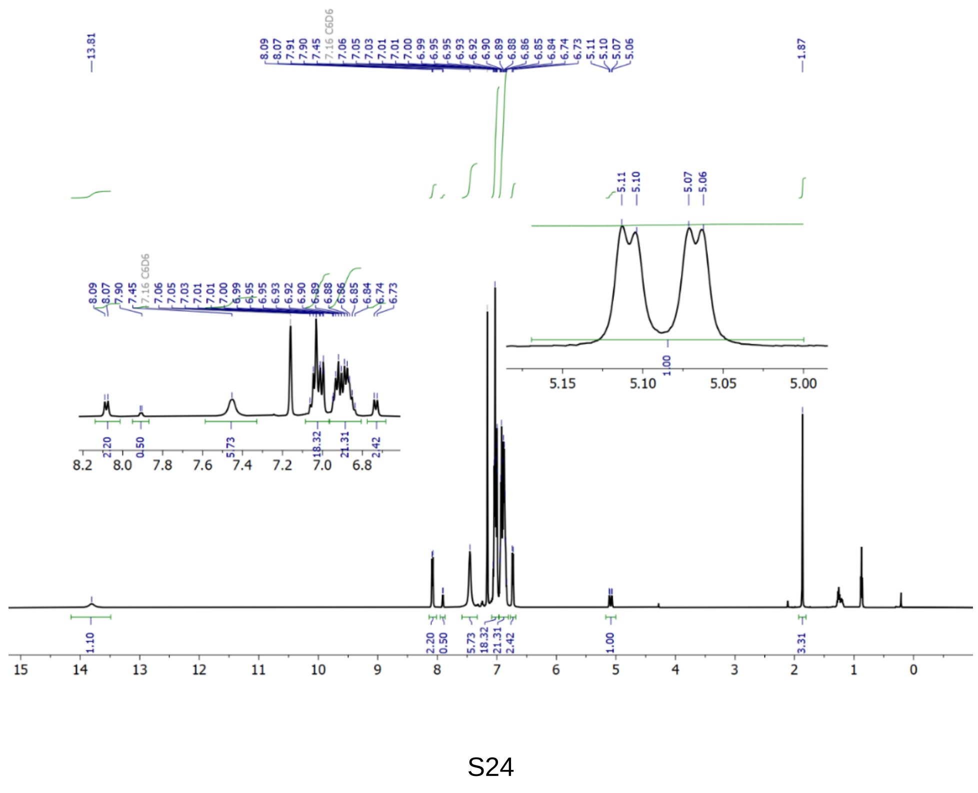

^{31}P NMR (162 MHz, 298 K, C_6D_6):

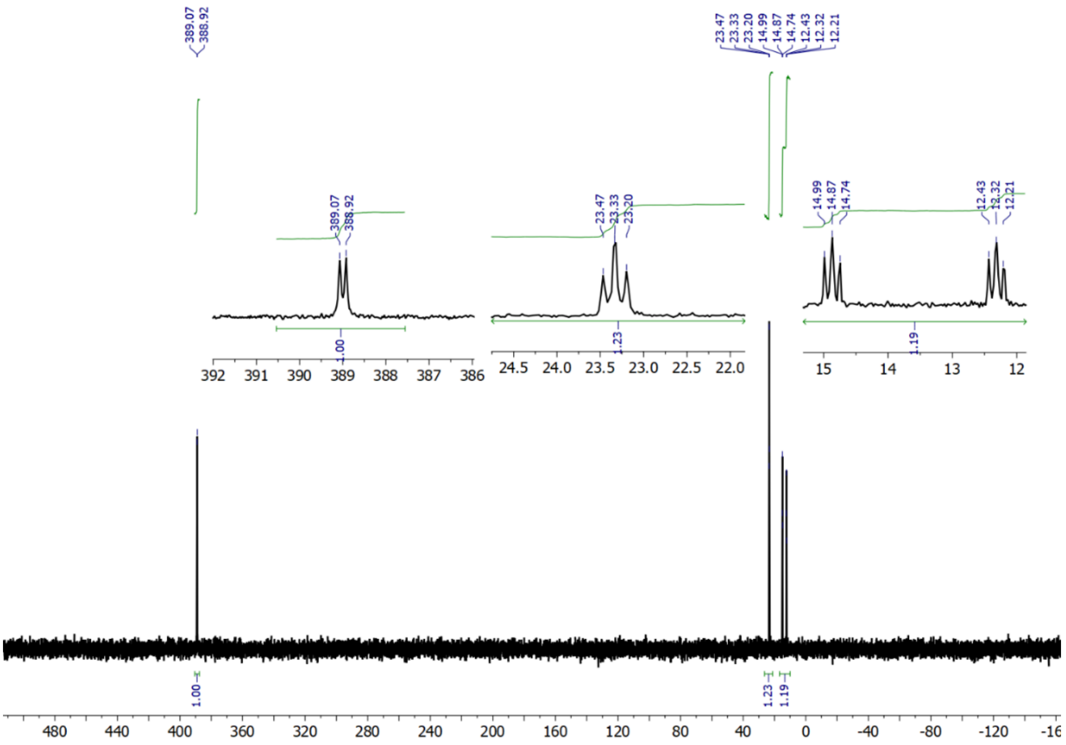

^{13}C{^{1}H} NMR (101 MHz, 298 K, C_6D_6):

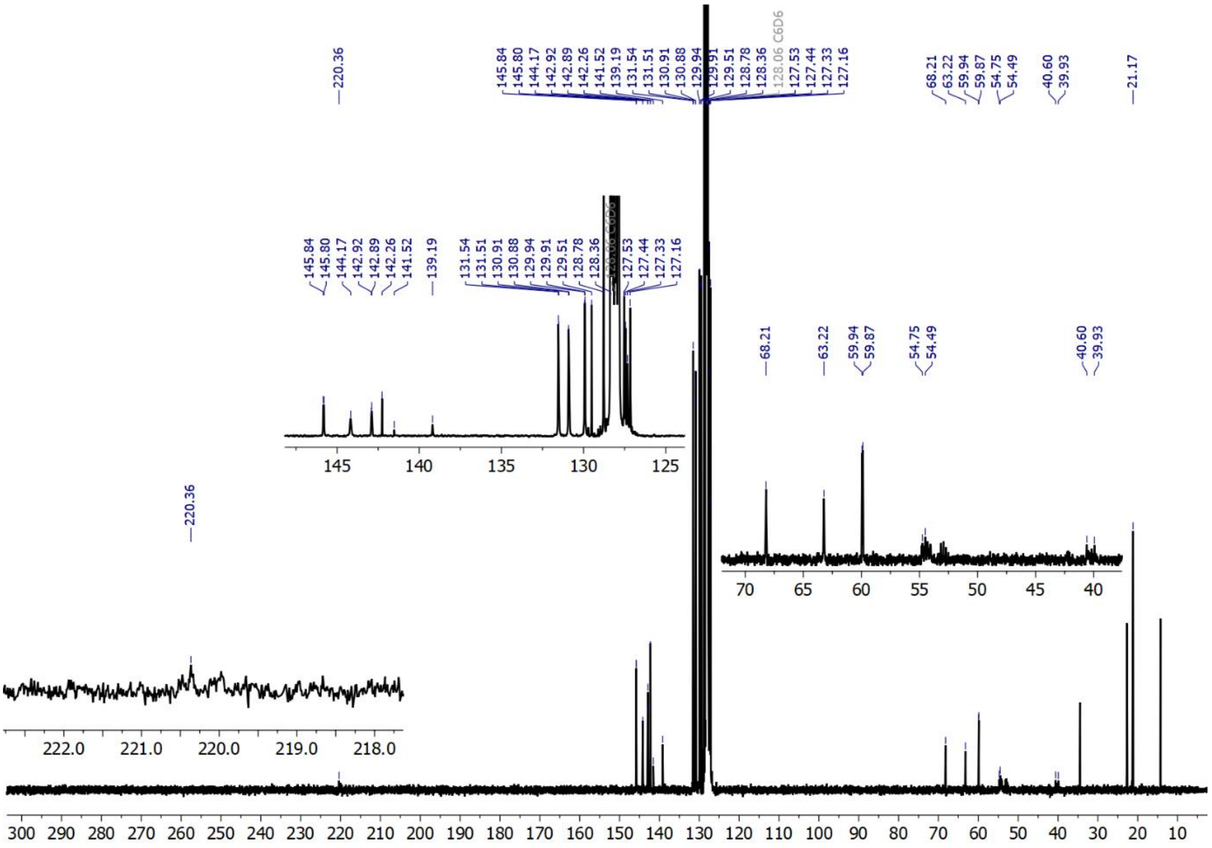

^{31}P-^{1}H COSY (298 K, C_6D_6):

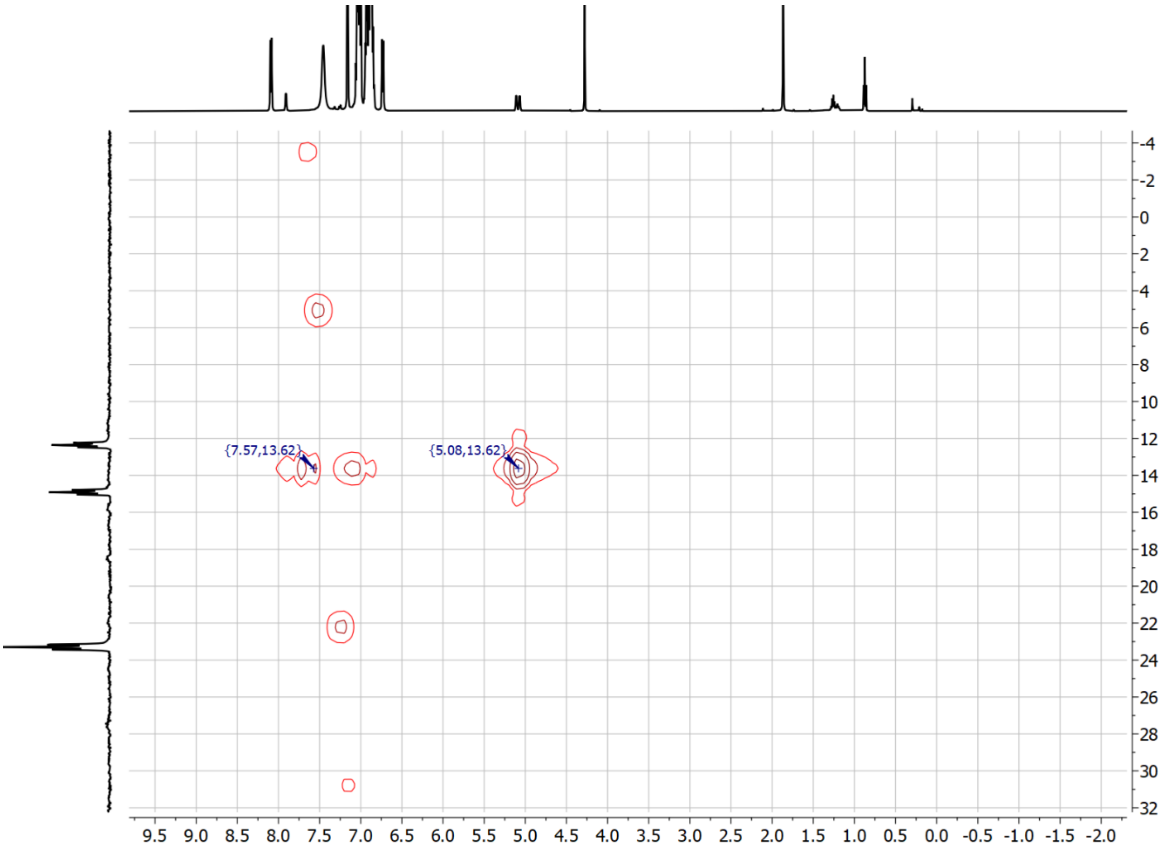

4f

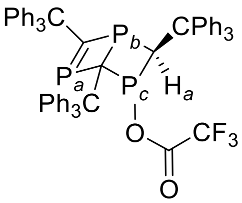

2,4,6-tris(triphenylmethyl)-Dewar-1,3,5-triphosphabenzene (0.05 mmol, 43 mg) was added to a J-Young's NMR tube along with C_6D_6 (0.6 mL). An excess of trifluoroacetic acid (16 µL, 0.2 mmol) was then added. The reaction was then heated to 80 °C, and the reaction was left at this temperature for 18 hours, before being cooled to room temperature. Volatiles were removed in vacuo to give a yellow solid. The crude product was crystalised via a dichloromethane/pentane vapour diffusion at -30 °C to give **4f** as yellow crystals (13 mg, 27%).

^{1}H NMR (500 MHz, 298 K, CD_2Cl_2): δ 7.25-7.07 (m, 32H, -C*Ph*$_3$), 6.97 (t, 6H, $^3J_{H\text{-}H}$ = 7.8 Hz, -C*Ph*$_3$), 6.83 (d, 6H, $^3J_{H\text{-}H}$ = 7.8 Hz, -C*Ph*$_3$), 3.95 (dd, 1H, $^2J_{H\text{-}P}$ = 6.5 Hz, $^2J_{H\text{-}P}$ = 2.5 Hz, H$_a$). **^{31}P NMR** (162 MHz, 298 K, CD_2Cl_2): δ 399.9 (d, 1P, $^2J_{P\text{-}P}$ = 33.6 Hz, P$_a$), 126.1 (d, 1P, $^2J_{P\text{-}P}$ = 30.0 Hz, P$_c$), 78.6 (at, 1P, $^2J_{P\text{-}P}$ 31.6 Hz, P$_b$). **^{13}C{^{1}H} NMR** (101 MHz, 298 K, CD_2Cl_2): δ 145.8 (d, $^3J_{P\text{-}P}$ = 3.3 Hz, -C*Ph*$_3$), 144.4 (d, $^3J_{P\text{-}P}$ = 3.3 Hz, -C*Ph*$_3$), 130.7 (d, $^3J_{P\text{-}P}$ = 3.8 Hz, -C*Ph*$_3$), 127.8 (-C*Ph*$_3$), 129.6 (br, -C*Ph*$_3$), 129.2 (-C*Ph*$_3$), 129.0 (-C*Ph*$_3$), 128.3 (-C*Ph*$_3$), 128.2 (-C*Ph*$_3$), 128.0 (-C*Ph*$_3$), 127.8 (-C*Ph*$_3$), 127.6 (br, -C*Ph*$_3$), 127.2 (-C*Ph*$_3$), 126.6 (d, $^3J_{P\text{-}P}$ = 3.3 Hz, -C*Ph*$_3$), 66.7 (*C*Ph$_3$), 59.3 (m, P$_b$-*C*(H$_a$)-P$_c$). **^{19}F NMR** (470 MHz, 298 K, CD_2Cl_2): δ -75.0 (s). **Melting point:** 219-223 °C (yellow->dark red decomp.). **IR:** (ATR, 298 K): 3056 (vw), 2964 (vw), 1782 (s), 1595 (w), 1492 (s), 1444 (s), 1346 (w), 1261 (s), 1216 (s), 1157 (s), 1118 (vs), 1086 (s), 1055 (s), 1033 (s), 1017 (s), 891 (w), 844 (w), 795 (vs), 730 (s), 696 (vs), 642 (s). **HRMS** (ESI): 973.2718 [M+H]$^+$ (calculated: 973.2743).

^{1}H NMR (500 MHz, 298 K, CD_2Cl_2):

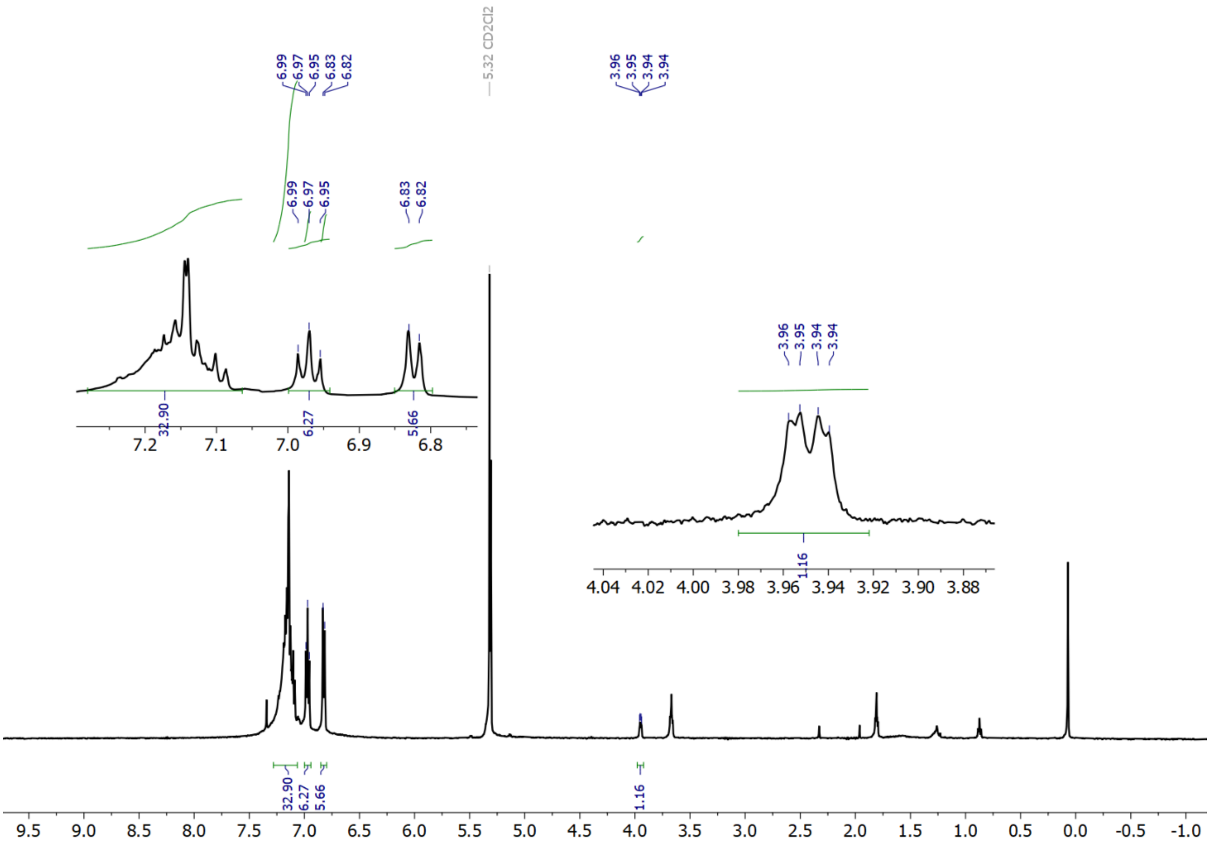

^{31}P NMR (162 MHz, 298 K, CD_2Cl_2):

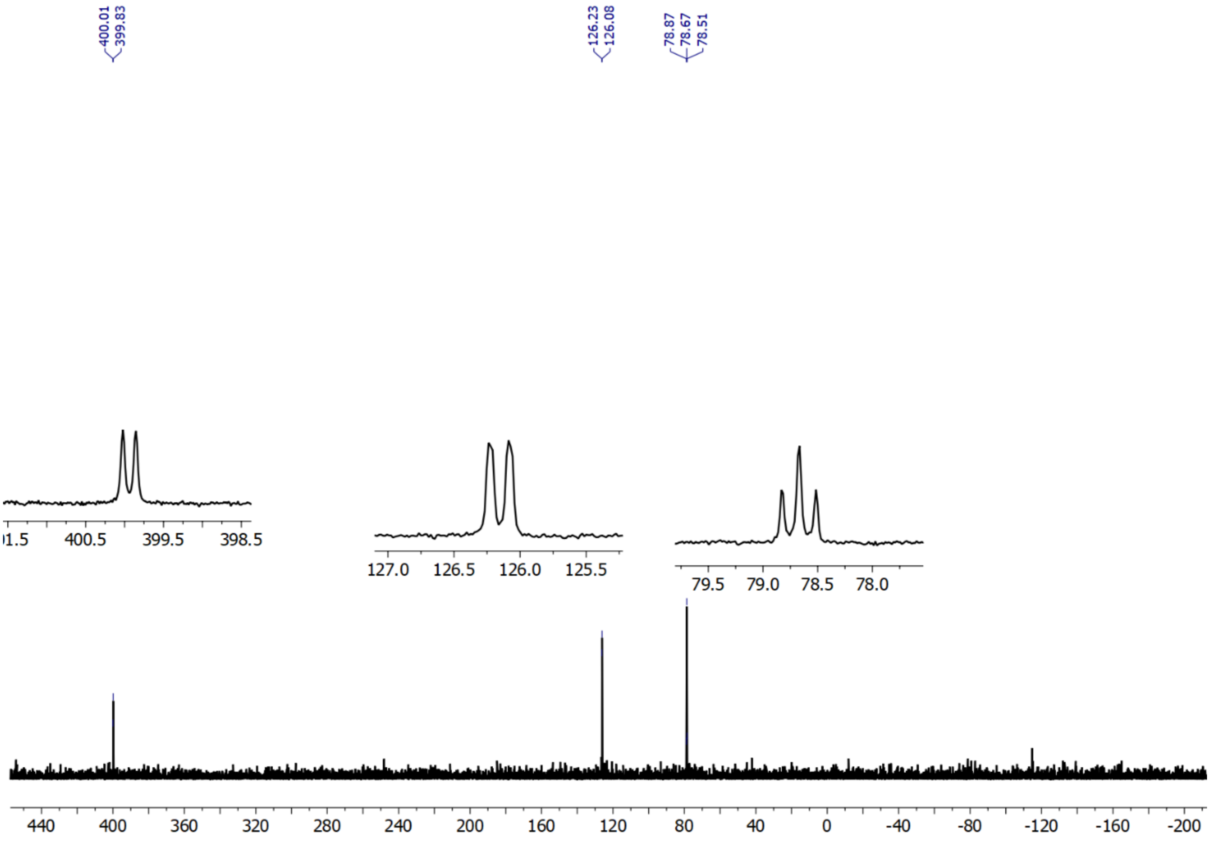

^{13}C{^{1}H} NMR (101 MHz, 298 K, CD_2Cl_2):

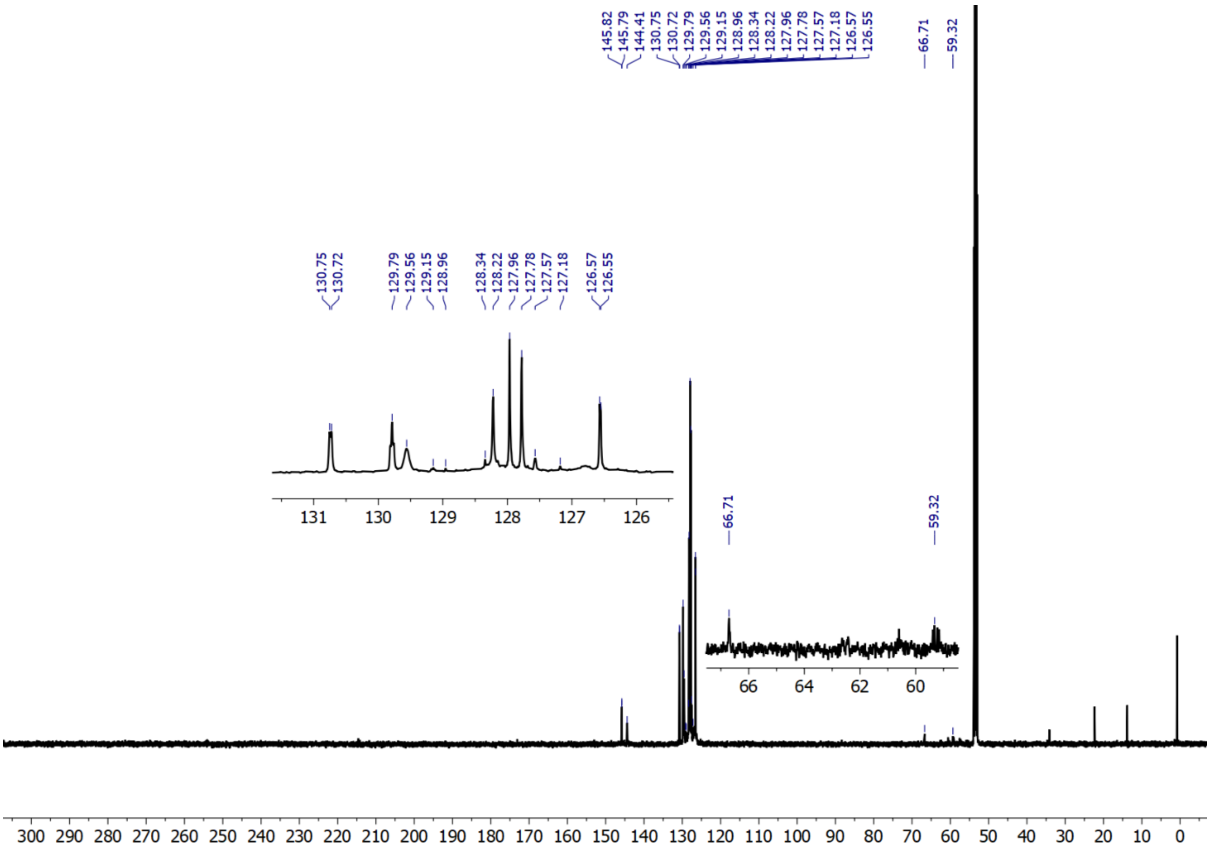

^{19}F NMR (470 MHz, 298 K, CD_2Cl_2):

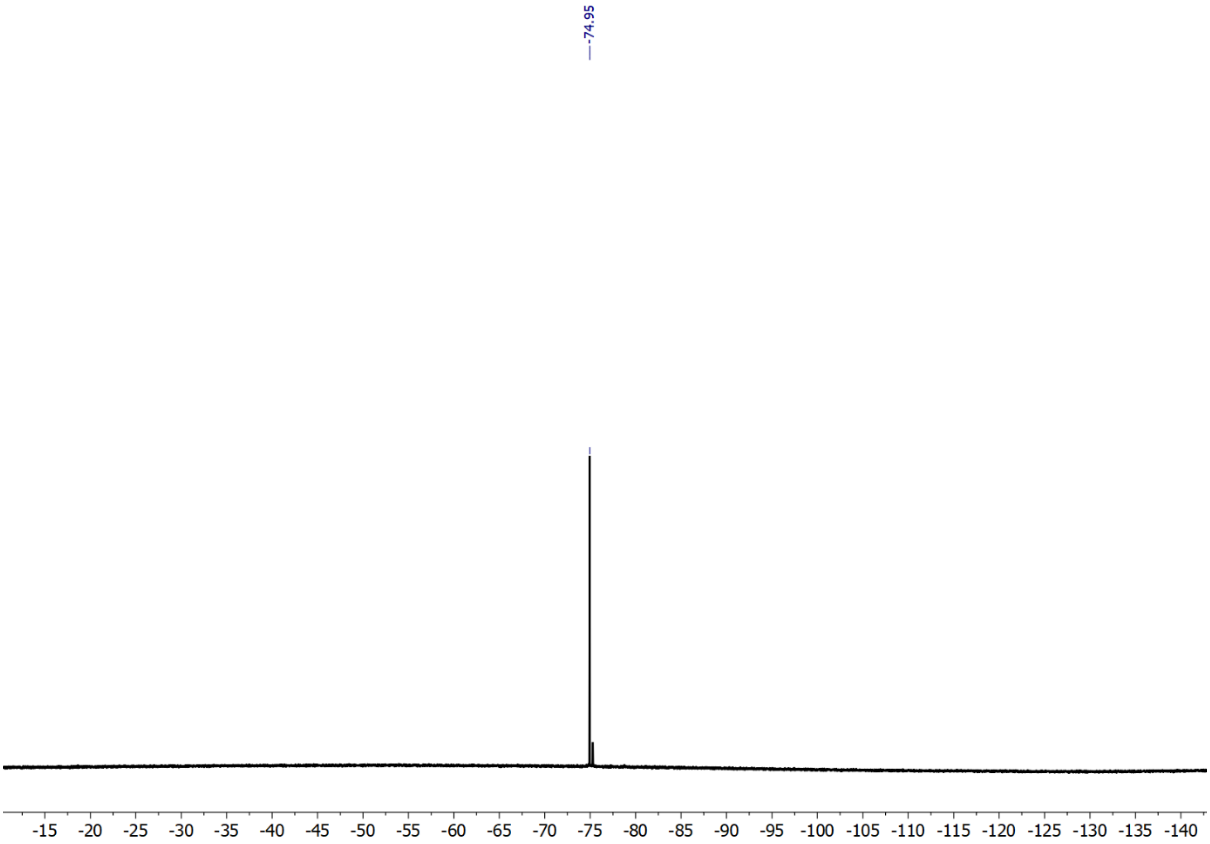

8. Kinetic Data for the Formation of 3

Separate NMR scale reactions were undertaken using the following deviations from the general procedure to form **3**:

0.375 mmol (108 mg) **2**

0.3125 mmol (90 mg) **2**

0.1875 mmol (54 mg) **2**

3 mol% (4.8 mg) **1**

1 mol% (1.6 mg) **1**

Each reaction was then monitored over 14 hours at room temperature, with ^{31}P NMR spectra recorded at ten minute intervals. The data was then processed and analysed by VTNA.[5-6] For **1**, the best overlap is clearly found with half order. For **2**, both half order and first order gave reasonable overlaps, therefore the reaction rate at each concentration of **2** was plotted against starting concentrations of **2**, where a significantly better trend was found for first order with respect to **2**.

VTNA graphs for order in **1**:

First order

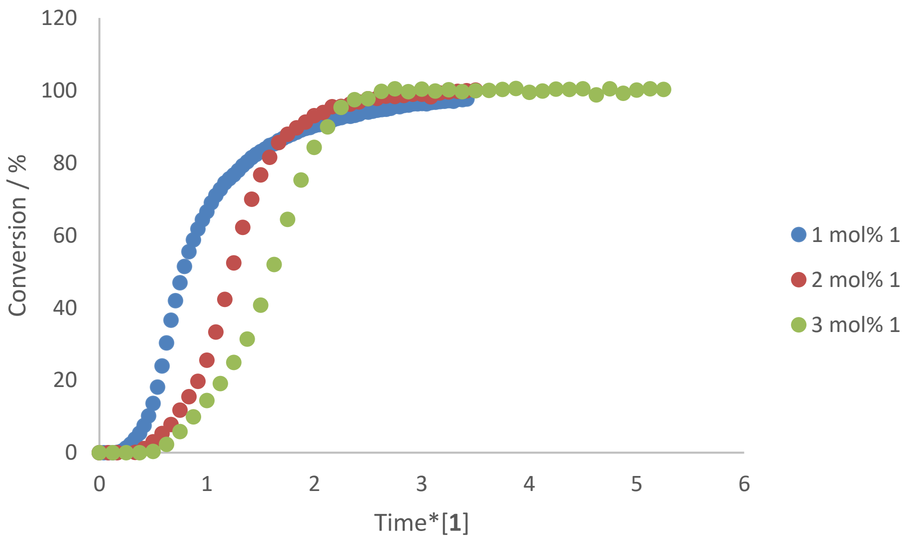

Second order

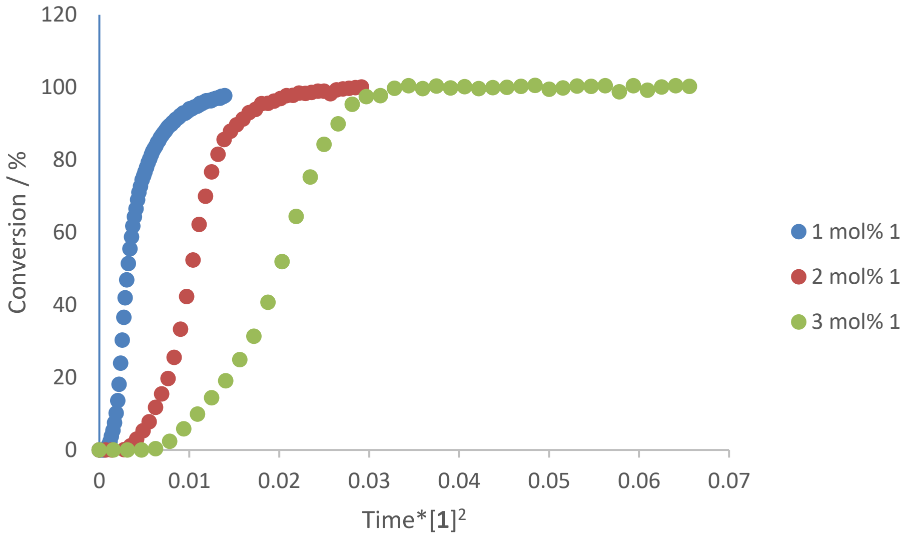

Half order

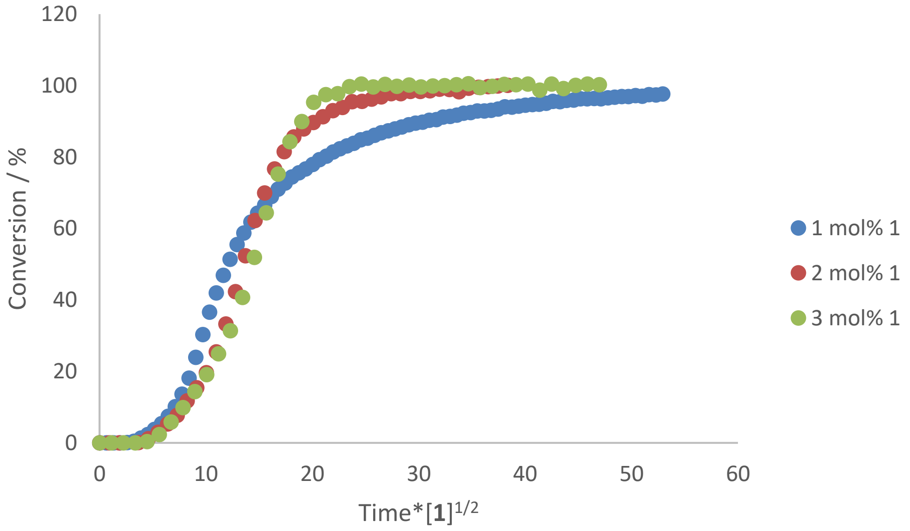

<u>VTNA Graphs for Order in **2**:</u>

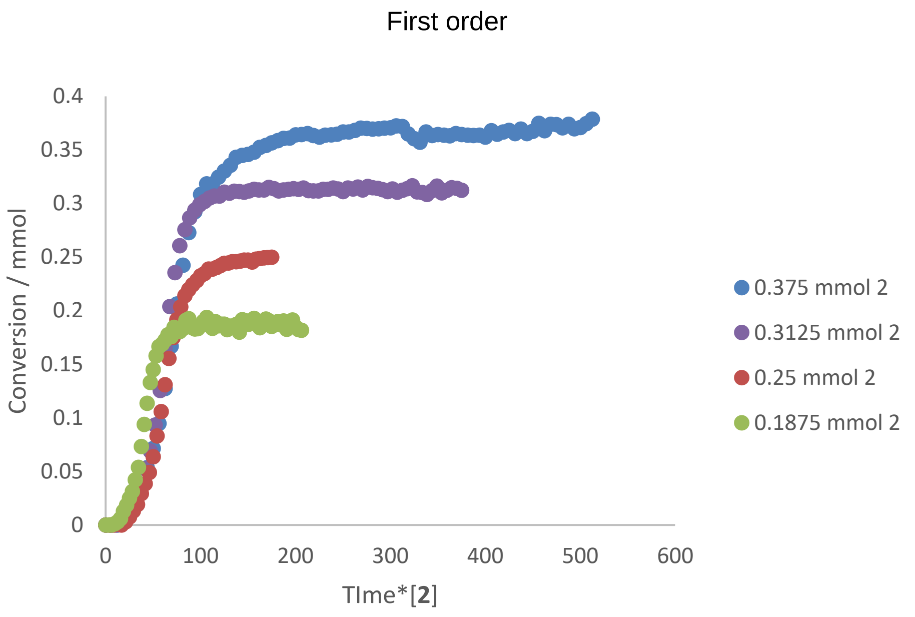

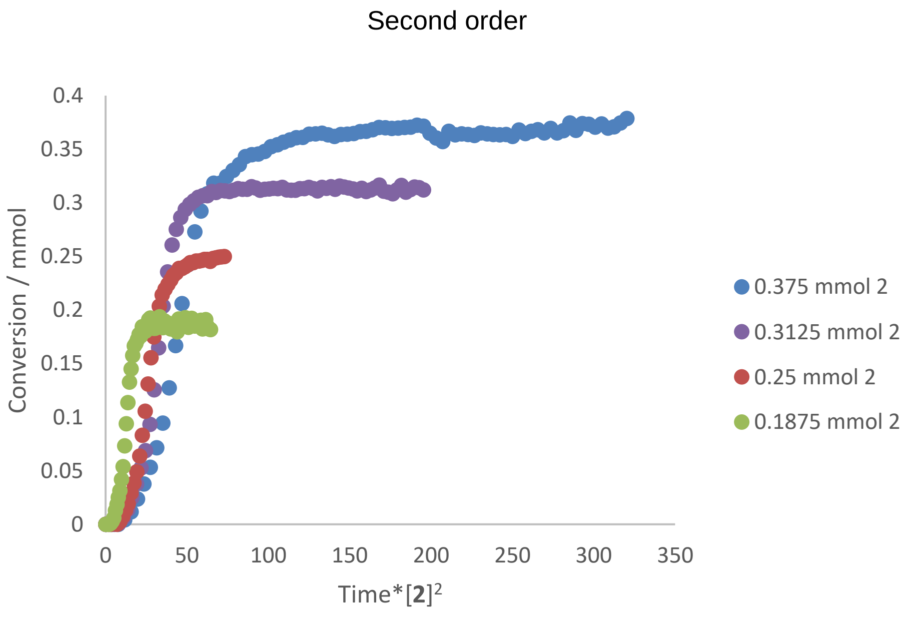

Half order

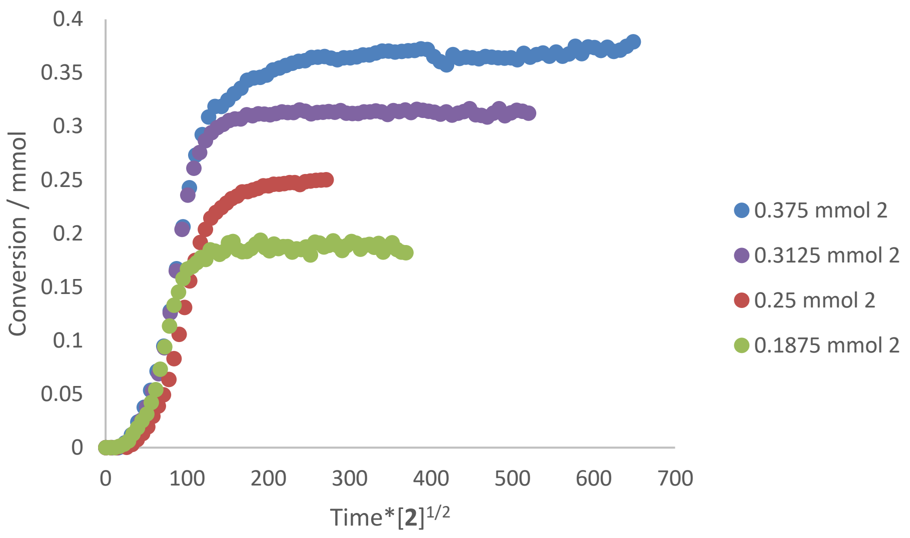

First order vs half order in **2** – Rate vs Concentration

First order

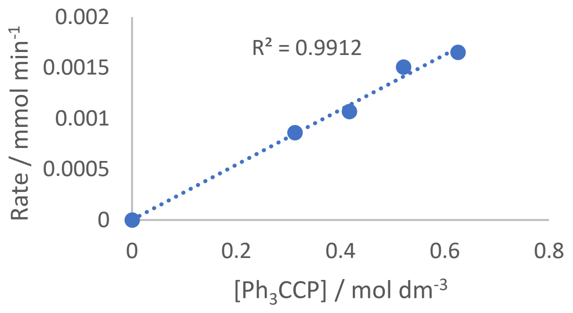

Half order

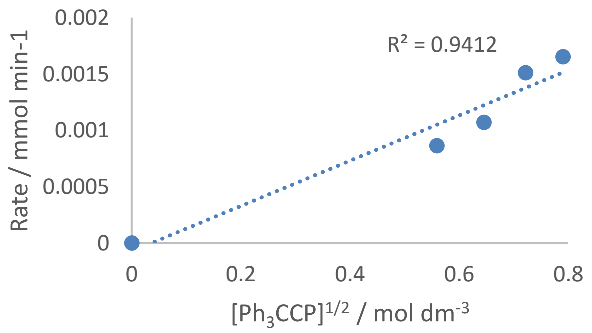

9. Reactions of 3 with NaHMDS and KHMDS

Reactions were set up between **3** and NaHMDS and KHMDS to see if isomerisation to **3’** could also be facilitated by these salts. Conditions: 0.05 mmol **3**, 0.1 mmol Na/KHMDS, 0.6 mL Tol-d_8.

After 1 week at 110 °C, no change was observed in the ^{31}P NMR spectrum of the reaction with **3** and KHMDS. In the reaction with **3** and NaHMDS, conversion to **3’** was observed after the same time and temperature. However, new signals relating to unknown impurities at 169.5 (br) and -17.2 ppm were also observed in the ^{31}P NMR spectrum (shown below).

^{31}P NMR: (162 MHz, 298 K, Tol-d_8):

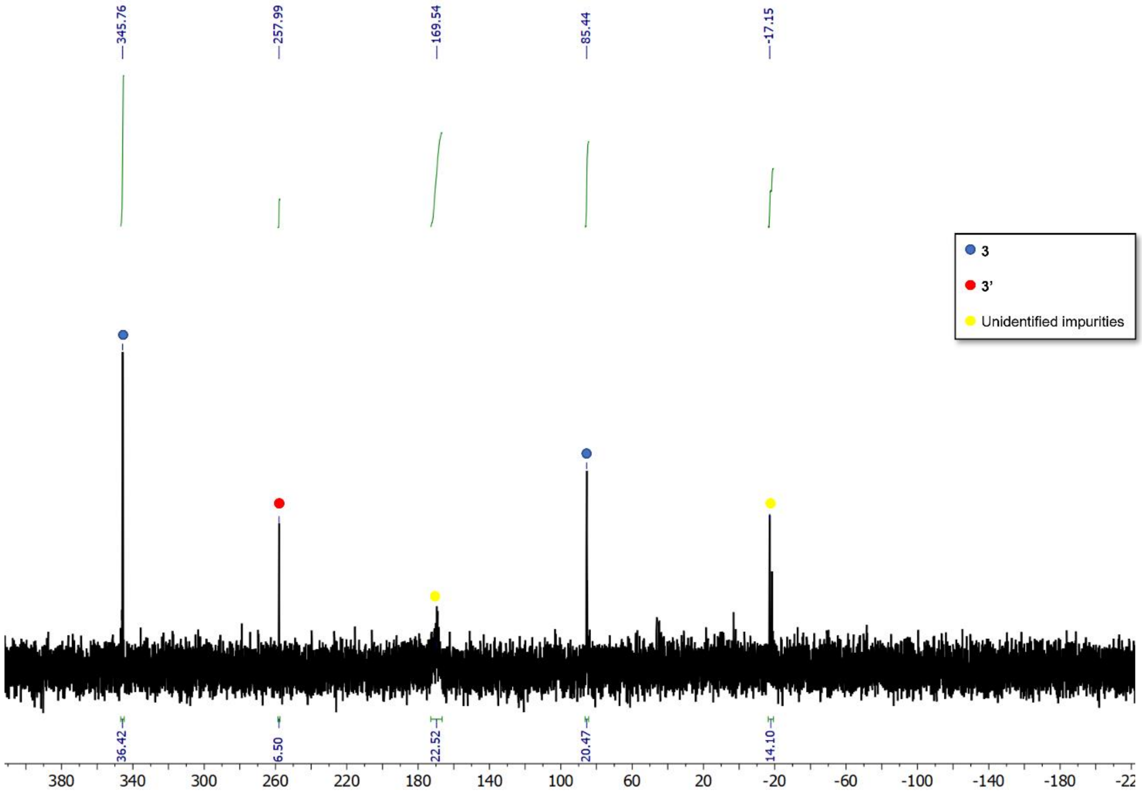

10. Investigations into the Reactivity of 1 with Further Phosphaalkynes

Adamantylphosphaalkyne was synthesised from tris(trimethylsilyl)phosphine as per the method used by Becker and co-workers.[7] When implemented into the trimerisation conditions (0.5 mmol phosphaalkyne, 2 mol% **1**, 0.5 eq. pinacolborane, 1:1 MeCN:Tol (0.6 mL), no conversion of adamantylphosphaalkyne was observed by ^{31}P NMR spectroscopy after 1 d at RT. A black precipitate had also formed, indicating that the iron catalysed had most likely decomposed. No further conversion was observed after further heating the reaction at 80 °C for 1 day.

Trimethylsilylphosphaalkyne was also synthesised via the method used by Russel and co-workers.[8] When implemented into the trimerisation conditions (1 mL of a 0.17 M stock solution of phosphaalkyne, 5 mol% **1**, 0.5 eq. pinacolborane) no reaction was observed at RT or 50 °C via ^{31}P NMR analysis.

Efforts were also made to synthesise novel phosphaalkynes with substituted -CAr_3 groups via routes analogous to that used to synthesize **2** (S2-S3). Triphenylmethane variants with 4-OMe-phenyl and 2-pyridyl groups were synthesised according to literature procedures.[9-10]

$((4\text{-OMe})Ph)_3CH$ proved difficult to deprotonate, causing issues in the synthesis of $((4\text{-OMe})Ph)_3CCH_2Cl$. *n*BuLi proved insufficient, with no deprotonation of $((4\text{-OMe})Ph)_3CH$ observed in different solvent systems (THF, diethyl ether, 1 : 1 THF : diethyl ether) or a range of addition temperatures / reaction times (-78 – 0 °C, 1 h – 18 h). Reactions of $((4\text{-OMe})Ph)_3CH$ with a large excess of *n*BuLi as well as *n*BuLi in the presence of TMEDA also yielded no deprotonated product upon analysis by ^{1}H NMR spectroscopy. Deprotonation with ^{i}PrMgCl·LiCl was also tested, however no reaction was observed with $((4\text{-OMe})Ph)_3CH$ by ^{1}H NMR at RT or 70 °C.

(2-pyridyl)$_3$CH proved easily deprotonated by *n*BuLi, and the subsequent organolithium reagent was added to an excess of dichloromethane to yield full conversion to (2-pyridyl)$_3$CCH$_2$Cl after 42 h stirring at RT. The product was isolated via flash column chromatography (silica, 1 : 2 ethyl acetate : petroleum ether). Unfortunately, issues arose in completing the next step of the synthesis (addition to PCl_3). Efforts to form the Grignard reagent of (2-pyridyl)$_3$CCH$_2$Cl were unsuccessful, despite activation of the magnesium turnings by flame-drying under vacuum, activation with iodine and refluxing the activated magnesium with the substrate in THF. We therefore sought to use a turbo-Grignard reagent, given their precedence for use in forming pyridyl-containing Grignard reagents. However, no reaction was observed between (2-pyridyl)$_3$CCH$_2$Cl and ^{i}PrMgCl·LiCl by ^{1}H NMR analysis at RT or 60 °C. A reaction with (2-pyridyl)$_3$CCH$_2$Cl with Li granules was also undertaken in hexane, again yielding no reaction after analysis by ^{1}H NMR spectroscopy. We then looked to replace the Cl moiety of (2-pyridyl)$_3$CCH$_2$Cl with a more reactive bromo-substituent. Repeating the synthesis of (2-pyridyl)$_3$CCH$_2$Cl with the use of dibromomethane instead of dichloromethane yielded pure (2-pyridyl)$_3$CCH$_2$Br after crystallisation from hot cyclohexane. Despite the more reactive bromo group, no reactivity was observed in reactions with activated magnesium, ^{i}PrMgCl·LiCl as well as a further reaction with sodium metal and a catalytic quantity of naphthalene each at reflux in THF. Full synthetic details and characterisations of (2-pyridyl)$_3$CCH$_2$Cl and (2-pyridyl)$_3$CCH$_2$Br are given below.

(2-pyridyl)$_3$CCH$_2$Cl

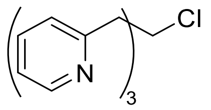

(2-pyridyl)$_3$CH[9] (1.07 g, 4.3 mmol) was added to a Schlenk flask along with THF (15 mL). The reaction was cooled to 0 °C before the dropwise addition of *n*BuLi (4.3 mmol), giving a red colour change. The reaction was warmed to RT and stirred for 1 h. The reaction mixture was then transferred dropwise via canula into another flask containing DCM (20 mL). The reaction was stirred at RT for 42 h until full conversion of starting material was observed via ^{1}H NMR analysis of a taken aliquot. The reaction was then quenched with de-ionised water (20 mL) and the organic layer was extracted and washed with further de-ionised water (2 x 20 mL). The organic layer was then dried over magnesium sulfate before being concentrated under vacuum, and the resulting crude product was purified by column chromatography (silica, 1 : 2 ethyl acetate : petroleum ether, ramping to 1 : 1) to yield (2-pyridyl)$_3$CCH$_2$Cl as a pale yellow oil which solidified overnight (0.89 g, 70%).

^{1}H NMR (500 MHz, 298 K, CDCl$_3$): δ 8.56 (ddd, 3H, J = 4.8, 1.9, 0.9 Hz, Ar-*H*), 7.62 (ddd, 3H, J = 8.1, 7.5, 1.9 Hz, Ar-*H*), 7.38 (adt, 3H, J = 8.0, 1.0 Hz, Ar-*H*), 7.14 (ddd, 3H, J = 7.5, 4.8, 1.1 Hz, Ar-*H*), 5.05 (s, 2H, -C*H$_2$*Cl). **^{13}C{^{1}H} NMR** (101 MHz, 298 K, CDCl$_3$): δ 162.6 (Ar-*C*), 148.8 (Ar-*C*), 136.0 (Ar-*C*), 125.0 (Ar-*C*), 121.8 (Ar-*C*), 64.7 (Ar$_3$*C*-CH$_2$Cl), 51.7 (-*C*H$_2$Cl). **Melting point:** 83-85 °C. **IR:** (ATR, 298 K): 3058 (vw), 3005 (vw), 2982 (vw), 2925 (vw), 1586 (s), 1566 (s), 1464 (s), 1425 (s), 1296 (w), 1273 (w), 1261 (w), 1157 (w), 1097 (vw), 1052 (w), 993 (s), 954 (w), 935 (w), 841 (w), 782 (s), 774 (s), 741 (vs), 717 (s), 656 (vs). **HRMS** (ESI): 296.0950 m.z [M+H]$^+$ (calculated: 296.0955).

^{1}H NMR (500 MHz, 298 K, CDCl$_3$):

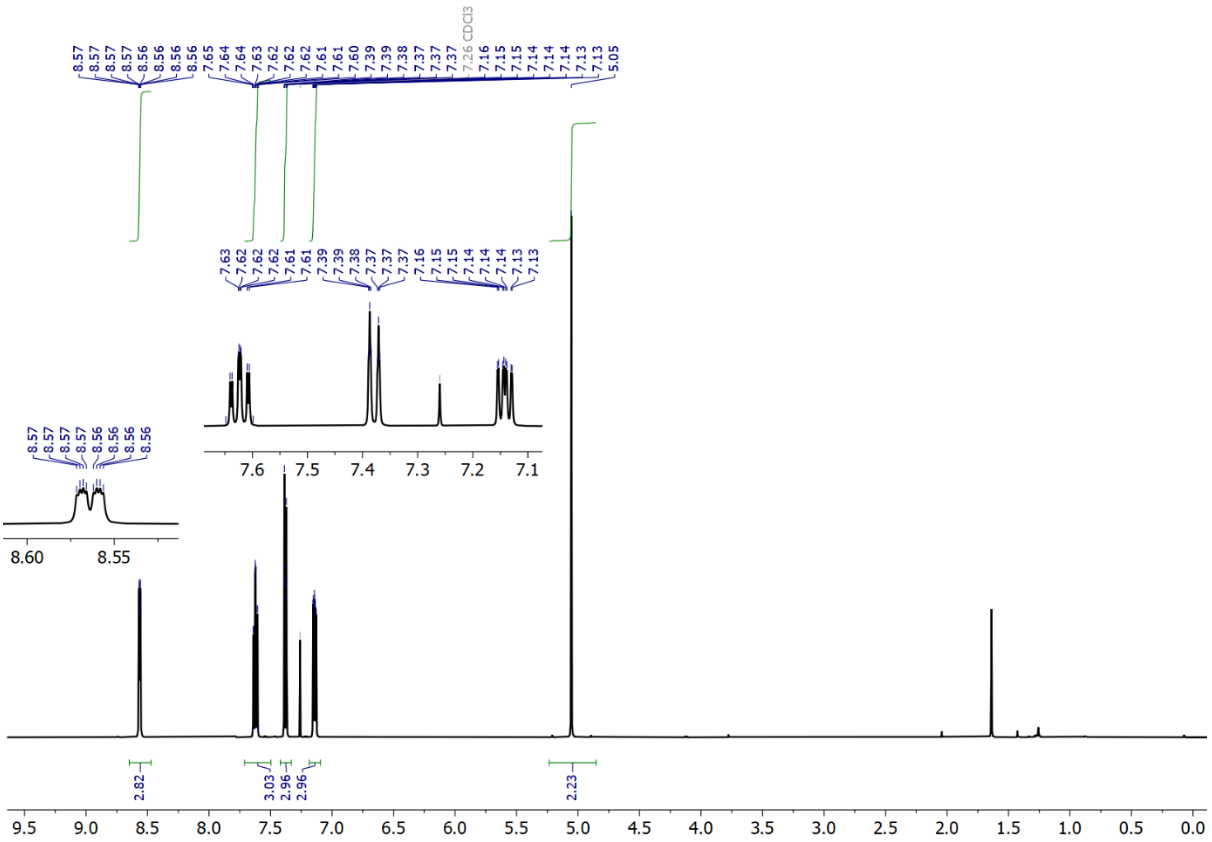

$^{13}C\{^1H\}$ NMR (101 MHz, 298 K, $CDCl_3$):

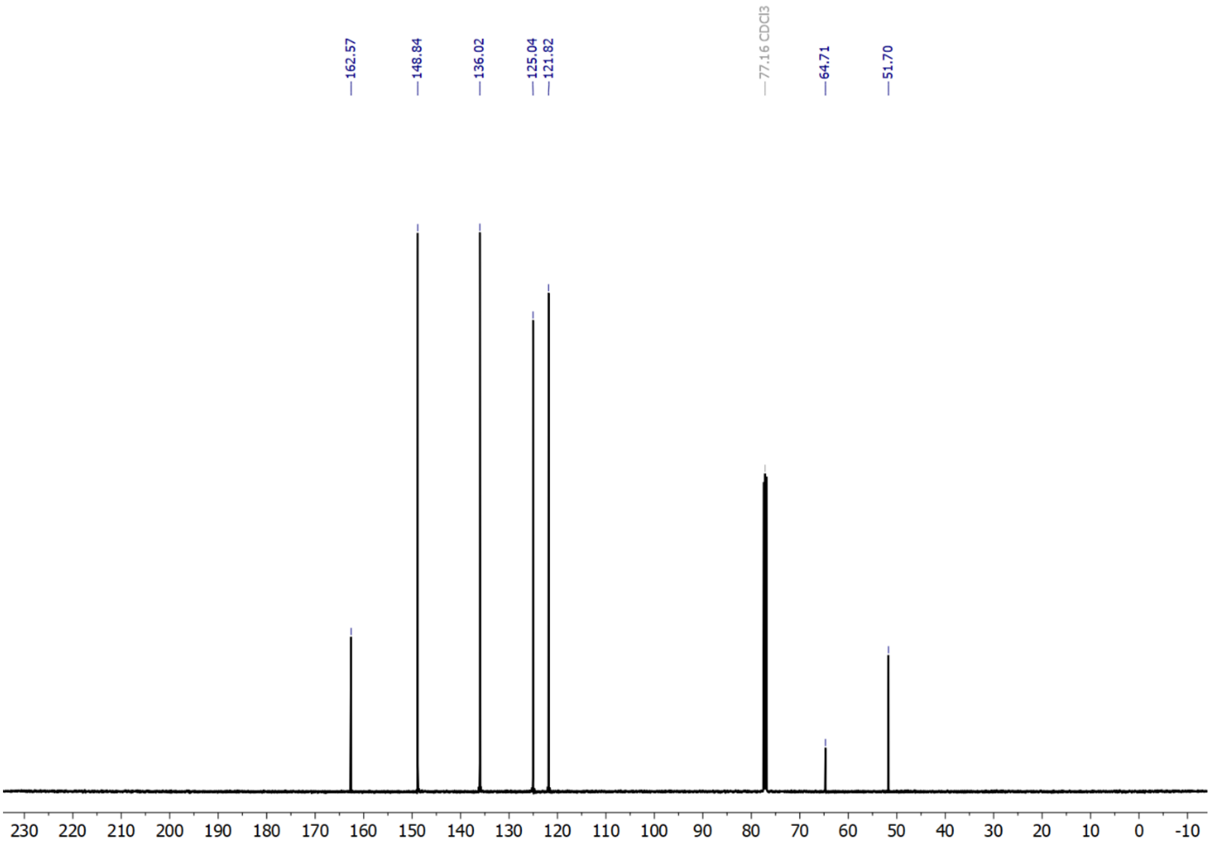

$(2\text{-pyridyl})_3CCH_2Br$

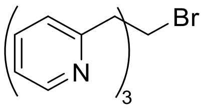

$(2\text{-pyridyl})_3CH$[9] (1.28 g, 5.1 mmol) was added to a Schlenk flask along with THF (15 mL). The reaction was cooled to 0 °C before the dropwise addition of *n*BuLi (5.1 mmol), giving a red colour change. The reaction was warmed to RT and stirred for 1 h. The reaction mixture was then transferred dropwise via canula into another flask containing dibromomethane (20 mL). The reaction was stirred at RT for 1 h until full conversion of starting material was observed via ^1H NMR analysis of a taken aliquot. The reaction was then quenched with de-ionised water (20 mL) and the organic layer was extracted and washed with further de-ionised water (2 x 20 mL). The organic layer was then dried over magnesium sulfate before being concentrated under vacuum to give a red/brown crude oil. The resulting crude product was crystallized from hot cyclohexane to yield $(2\text{-pyridyl})_3CCH_2Br$ as orange crystals (1.29 g, 74%).

^1H NMR (500 MHz, 298 K, $CDCl_3$): δ 8.56 (ddd, 3H, J = 4.8, 1.9, 1.1 Hz, Ar-*H*), 7.62 (atd, 3H, J = 7.8, 1.9 Hz, Ar-*H*), 7.40 (dm, 3H, J = 8.0, Ar-*H*), 7.14 (ddd, 3H, J = 7.6, 4.8, 1.1 Hz, Ar-*H*), 4.92 (s, 2H, -C*H*$_2$Br). **$^{13}C\{^1H\}$ NMR** (101 MHz, 298 K, $CDCl_3$): δ 162.7 (Ar-*C*), 148.8 (Ar-*C*), 136.0 (Ar-*C*), 125.0 (Ar-*C*), 121.8 (Ar-*C*), 64.0 (Ar_3C-CH_2Br), 42.0 (-*C*H_2Br). **Melting point:** 129-131 °C. **IR:** (ATR, 298 K): 3065 (vw), 3004 (vw), 1585 (s), 1567 (s), 1464 (vs), 1428 (vs), 1402 (w), 1297 (vw), 1230 (w), 1154 (w), 1110 (w), 1053 (vw), 994 (s), 928 (vw), 879 (vw), 830 (s), 777 (vs), 755 (vs), 726 (w), 687 (w). **HRMS** (ESI): 340.0446 m.z $[M+H]^+$ (calculated: 340.0449).

^{1}H NMR (500 MHz, 298 K, $CDCl_3$):

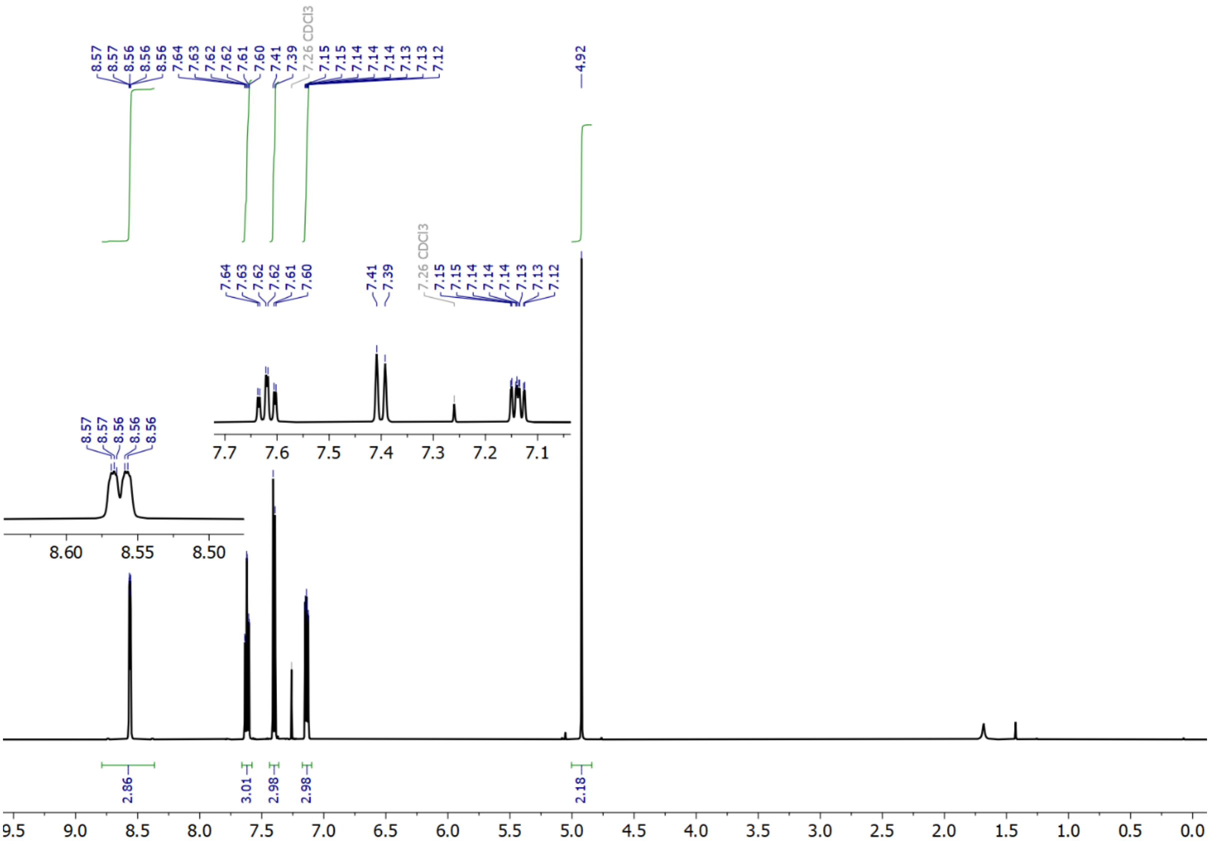

^{13}C{^{1}H} NMR (101 MHz, 298 K, $CDCl_3$):

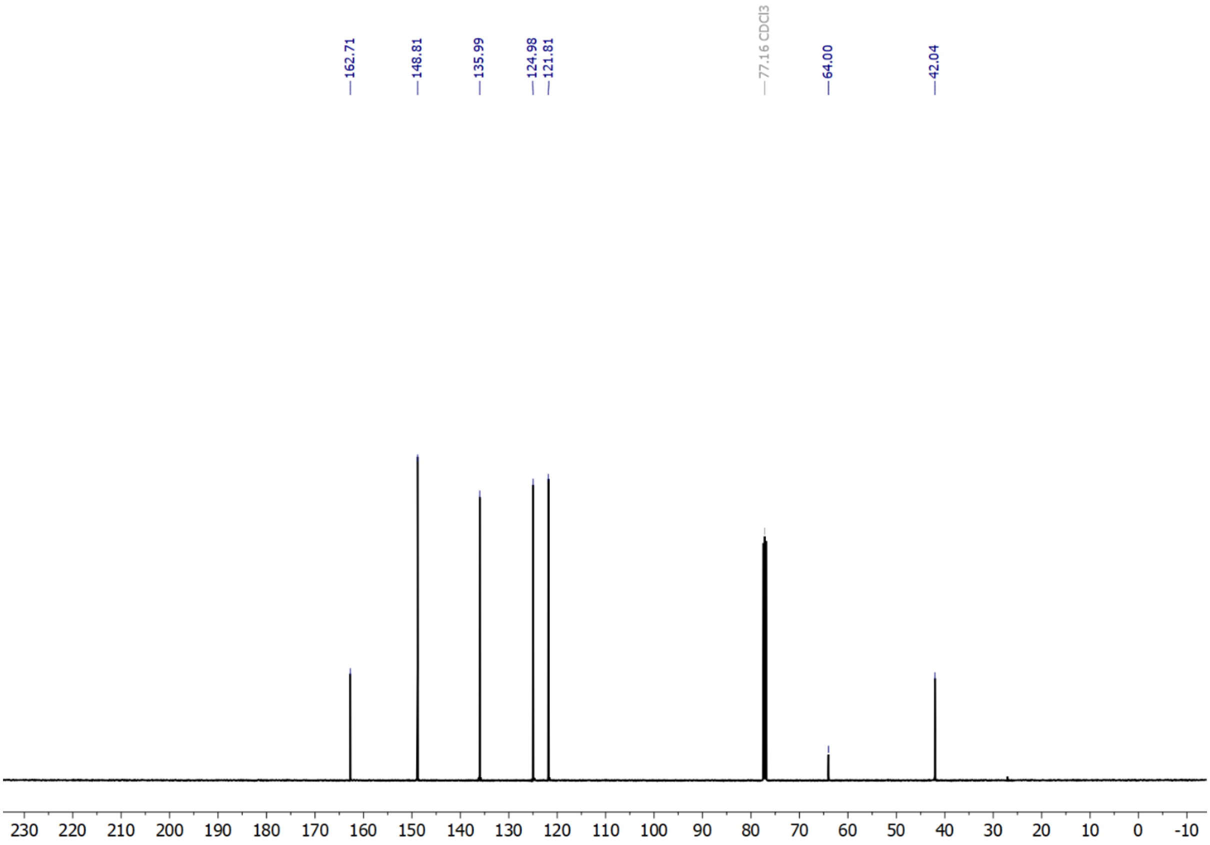

11. Crystallographic Information

Data for **3, 3'', ClAu-3, ClAu-3', 4a, 4c, 4e,** and **4f** were collected an Agilent SuperNova diffractometer (using a Cu-K□ radiation) while those for **4b** were obtained using an Agilent Xcalibur instrument and an Mo-K□ source. All experiments were conducted at 150 K, solved using SHELXT[11] and refined using SHELXL[12] via the Olex-2[13] interface. Noteworthy points in the individual X-ray diffraction experiments follow along with crystal structure and refinement details.

Crystallographic data for all compounds have been deposited with the Cambridge Crystallographic Data Centre as supplementary publications CCDC 2177646-2177653 for **3'', ClAu-3, 3, 4a, 4b, 4e, 4f** and **4c**, respectively. The comparative code for **ClAu-3'** is CCDC 2178718. Copies of these data can be obtained free of charge on application to CCDC, 12 Union Road, Cambridge CB2 1EZ, UK [fax(+44) 1223 336033, e-mail: deposit@ccdc.cam.ac.uk.

Crystal structure data and refinement details are collated in Tables **S2**-**S10** below. Comparisons of select bond distances/angles for solid-state structures **3**, **4a**, **4b**, **4e** and **4f** are shown in Table **S11**.

Table S2: Crystal Data and Structure Refinement for **3**.

Compound	**3**
Empirical formula	$C_{61}H_{47}Cl_2P_3$
Formula weight	943.79
Temperature/K	150.01(10)
Crystal system	monoclinic
Space group	$P2_1/c$
a/Å	9.4715(1)
b/Å	17.7797(1)
c/Å	28.8562(3)
α/°	90
β/°	93.699(1)
γ/°	90
Volume/Å^3	4849.27(8)
Z	4
ρ_{calc}g/cm^3	1.293
μ/mm^{-1}	2.444
F(000)	1968.0
Crystal size/mm^3	0.242 × 0.103 × 0.087
Radiation	CuKα (λ = 1.54184)
2Θ range for data collection/°	7.902 to 146.528
Index ranges	-8 ≤ h ≤ 11, -21 ≤ k ≤ 21, -35 ≤ l ≤ 35
Reflections collected	45757
Independent reflections	9663 [R_{int} = 0.0343, R_{sigma} = 0.0245]
Data/restraints/parameters	9663/0/568
Goodness-of-fit on F^2	1.040
Final R indexes [I>=2σ (I)]	R_1 = 0.0369, wR_2 = 0.0943
Final R indexes [all data]	R_1 = 0.0414, wR_2 = 0.0976
Largest diff. peak/hole / e Å^{-3}	0.33/-0.27

Solvent present in the structure of **3** appeared to be predominantly dichloromethane, but this is disordered with a small amount of pentane (20%). As such the lattice guests were treated with the solvent mask algorithm available in Olex-2, and an allowance of one molecule of dichloromethane per asymmetric unit made in the formula as presented.

Table S3: Crystal Data and Structure Refinement for **3''**.

Compound	**3''**
Empirical formula	$C_{60}H_{45}P_3$
Formula weight	858.87
Temperature/K	150.00(10)
Crystal system	triclinic
Space group	P-1
a/Å	9.9174(2)
b/Å	13.4028(3)
c/Å	16.8939(4)
α/°	87.126(2)
β/°	84.096(2)
γ/°	86.692(2)
Volume/Å^3	2227.71(9)
Z	2
ρ_{calc}g/cm^3	1.280
μ/mm^{-1}	1.532
F(000)	900.0
Crystal size/mm^3	0.289 × 0.264 × 0.124
Radiation	Cu Kα (λ = 1.54184)
2Θ range for data collection/°	8.27 to 145.254
Index ranges	-11 ≤ h ≤ 12, -14 ≤ k ≤ 16, -20 ≤ l ≤ 20
Reflections collected	26615
Independent reflections	8823 [R_{int} = 0.0241, R_{sigma} = 0.0278]
Data/restraints/parameters	8823/0/568
Goodness-of-fit on F^2	1.024
Final R indexes [I>=2σ (I)]	R_1 = 0.0328, wR_2 = 0.0835
Final R indexes [all data]	R_1 = 0.0346, wR_2 = 0.0850
Largest diff. peak/hole / e Å^{-3}	0.34/-0.24

Table S4: Crystal Data and Structure Refinement for **ClAu-3**.

Identification code	**ClAu-3**
Empirical formula	$C_{61}H_{47}AuCl_3P_3$
Formula weight	1176.21
Temperature/K	150.01(10)
Crystal system	monoclinic
Space group	$P2_1/n$
a/Å	12.7512(2)
b/Å	23.7151(2)
c/Å	17.7285(2)
α/°	90
β/°	110.896(1)
γ/°	90
Volume/Å^3	5008.43(11)
Z	4
ρ_{calc}g/cm^3	1.560
μ/mm^{-1}	8.208
F(000)	2352.0
Crystal size/mm^3	0.136 × 0.042 × 0.015
Radiation	Cu Kα (λ = 1.54184)
2Θ range for data collection/°	7.456 to 145.272
Index ranges	$-15 \le h \le 15$, $-19 \le k \le 29$, $-21 \le l \le 21$
Reflections collected	65177
Independent reflections	9925 [R_{int} = 0.0459, R_{sigma} = 0.0298]
Data/restraints/parameters	9925/0/613
Goodness-of-fit on F^2	1.079
Final R indexes [I>=2σ (I)]	R_1 = 0.0320, wR_2 = 0.0760
Final R indexes [all data]	R_1 = 0.0363, wR_2 = 0.0782
Largest diff. peak/hole / e Å^{-3}	2.00/-0.96

Analysis of the raw diffraction frames pertaining to **ClAu-3** revealed the presence of very minor crystal twinning. However, efforts to deconvolute this actually degraded the integrated data and were, thus, abandoned. The residual electron density maxima are chemically insignificant and are ripples in the region of the Au−Cl region. The asymmetric unit in this structure is also home to one molecule of dichloromethane.

Table S5: Crystal Data and Structure Refinement for **ClAu-3’**.

Identification code	**ClAu-3’**
Empirical formula	$C_{60.15}H_{45.3}Au_{0.8}Cl_{1.1}P_3$
Formula weight	1057.54
Temperature/K	150.01(10)
Crystal system	orthorhombic
Space group	$P2_12_12_1$
a/Å	9.37912(11)
b/Å	18.3184(2)
c/Å	27.2528(3)
α/°	90
β/°	90
γ/°	90
Volume/Å^3	4682.34(10)
Z	4
ρ_{calc}g/cm^3	1.500
μ/mm^{-1}	6.617
F(000)	2132.0
Crystal size/mm^3	0.29 × 0.101 × 0.075
Radiation	Cu Kα (λ = 1.54184)
2Θ range for data collection/°	8.086 to 144.594
Index ranges	-6 ≤ h ≤ 11, -22 ≤ k ≤ 22, -31 ≤ l ≤ 33
Reflections collected	49005
Independent reflections	9216 [R_{int} = 0.0347, R_{sigma} = 0.0256]
Data/restraints/parameters	9216/12/613
Goodness-of-fit on F^2	1.132
Final R indexes [I>=2σ (I)]	R_1 = 0.0236, wR_2 = 0.0550
Final R indexes [all data]	R_1 = 0.0237, wR_2 = 0.0550
Largest diff. peak/hole / e Å^{-3}	0.62/-0.49
Flack parameter	-0.022(3)

The motif in the structure of **ClAu-3’** contains an average of **ClAu-3’** (80%), **3’**(20%) and a CH_2Cl_2 moiety with 15% occupancy. Distance and ADP restraints were employed in the solvent area, in order to achieve a chemically sensible convergence.

Table S6: Crystal Data and Structure Refinement for **4a**.

Compound	**4a**
Empirical formula	$C_{63}H_{52}Cl_4IP_3$
Formula weight	1170.65
Temperature/K	150.00(10)
Crystal system	monoclinic
Space group	$P2_1$
a/Å	10.29173(15)
b/Å	20.8617(3)
c/Å	12.96787(18)
α/°	90
β/°	108.4400(16)
γ/°	90
Volume/Å^3	2641.28(7)
Z	2
ρ_{calc}g/cm^3	1.472
μ/mm^{-1}	7.799
F(000)	1192.0
Crystal size/mm^3	0.142 × 0.095 × 0.07
Radiation	Cu Kα (λ = 1.54184)
2Θ range for data collection/°	7.186 to 146.466
Index ranges	$-12 \le h \le 12$, $-25 \le k \le 25$, $-14 \le l \le 16$
Reflections collected	32380
Independent reflections	9331 [R_{int} = 0.0492, R_{sigma} = 0.0473]
Data/restraints/parameters	9331/67/668
Goodness-of-fit on F^2	1.046
Final R indexes [I>=2σ (I)]	R_1 = 0.0427, wR_2 = 0.1175
Final R indexes [all data]	R_1 = 0.0441, wR_2 = 0.1192
Largest diff. peak/hole / e Å^{-3}	0.89/-1.21
Flack parameter	-0.002(3)

Two molecules of dichloromethane in addition to one formula unit of the iodide salt comprise the asymmetric unit in the structure of **4a**. The solvent molecule based on C62 was treated for 50:50 disorder, with the inclusion of ADP and distance restraints. The highest residual electron density peak lies 0.88 Å from I1 and, as such, is chemically insignificant

Table S7: Crystal Data and Structure Refinement for **4b**.

Identification code	**4b**
Empirical formula	$C_{61}H_{47}Br_2Cl_2P_3$
Formula weight	1103.61
Temperature/K	150.04(18)
Crystal system	monoclinic
Space group	$P2_1/n$
a/Å	15.5670(2)
b/Å	11.08568(13)
c/Å	28.6941(4)
α/°	90
β/°	90.9787(11)
γ/°	90
Volume/Å^3	4951.05(11)
Z	4
ρ_{calc}g/cm^3	1.481
μ/mm^{-1}	1.884
F(000)	2248.0
Crystal size/mm^3	0.442 × 0.250 × 0.147
Radiation	Mo Kα (λ = 0.71073)
2Θ range for data collection/°	5.912 to 59.148
Index ranges	-20 ≤ h ≤ 19, -12 ≤ k ≤ 14, -39 ≤ l ≤ 39
Reflections collected	49620
Independent reflections	13168 [R_{int} = 0.0395, R_{sigma} = 0.0510]
Data/restraints/parameters	13168/0/586
Goodness-of-fit on F^2	1.021
Final R indexes [I>=2σ (I)]	R_1 = 0.0421, wR_2 = 0.0869
Final R indexes [all data]	R_1 = 0.0656, wR_2 = 0.0950
Largest diff. peak/hole / e Å^{-3}	1.36/-0.67

The asymmetric unit in the structure of **4b** comprises one molecule of the phosphorus containing complex and one molecule of dichloromethane. While disorder in the latter was not beyond modelling, the electron density remained somewhat smeared in that region of the difference Fourier electron density map. Thus, said solvent was ultimately treated using the Olex-2 solvent mask. The highest residual electron density peak lies 0.87 Å from Br2 and may even suggest some very minor disorder of this halide. However, efforts to model same rendered a disorder ratio of 98:2, and hence were not pursued.

Table S8: Crystal Data and Structure Refinement for **4c**.

Identification code	**4c**
Empirical formula	$C_{375}H_{361}Li_4P_{12}Si_4$
Formula weight	5379.38
Temperature/K	150.00(10)
Crystal system	triclinic
Space group	P-1
a/Å	17.3535(6)
b/Å	28.8721(5)
c/Å	29.9804(9)
α/°	90.3440(19)
β/°	90.654(3)
γ/°	90.586(2)
Volume/Å^3	15019.1(7)
Z	2
ρ_{calc}g/cm^3	1.190
μ/mm^{-1}	1.231
F(000)	5718.0
Crystal size/mm^3	0.271 × 0.22 × 0.071
Radiation	Cu Kα (λ = 1.54184)
2Θ range for data collection/°	7.75 to 146.526
Index ranges	-21 ≤ h ≤ 21, -35 ≤ k ≤ 35, -37 ≤ l ≤ 37
Reflections collected	61313
Independent reflections	61313 [R_{int} = 0.07781,[1] R_{sigma} = 0.0990]
Data/restraints/parameters	61313/396/2966
Goodness-of-fit on F^2	0.990
Final R indexes [I>=2σ (I)]	R_1 = 0.0861, wR_2 = 0.2251
Final R indexes [all data]	R_1 = 0.1153, wR_2 = 0.2410
Largest diff. peak/hole / e Å^{-3}	0.80/-0.35

[1]Recorded during integration of twinned dataset.

The structure of **4c** was a challenge. The asymmetric unit in the structure of **4c** comprises four molecules of the lithium based complex and a pool of toluene. Sample handling was exceedingly difficult because of the solvent content, as the crystals tended to crack and lose solvent once removed from the mother liquor leading to some degradation of the sample. Six of the toluene entities in the motif were order and included in the refinement while the remaining eleven were addressed using the SQUEEZE algorithm available in Platon.[14] This latter route was chosen in favour of inevitable, model over-parameterization that would have ensued with from the restraints and constraints needed to address extensive toluene disorder in the masked solvent regions of the electron-density map in the masked solvent regions of the electron-density map. Additionally, 50:50 disorder was accommodated in two of the target molecules in the structure, namely, the phenyl rings based on C11C and C36B. Distance and ADP restraints were employed, on merit, in disordered regions to assist convergence. Despite the challenges with sample handling, the experiment has rendered an unambiguous, solid-state characterisation of this compound.

Table S9: Crystal Data and Structure Refinement for **4e**.

Compound	**4e**
Empirical formula	$C_{69}H_{60}O_4P_3SCl_4$
Formula weight	1219.94
Temperature/K	150.00(10)
Crystal system	monoclinic
Space group	$P2_1/c$
a/Å	17.7622(4)
b/Å	9.7235(2)
c/Å	37.4661(9)
α/°	90
β/°	91.982(2)
γ/°	90
Volume/Å^3	6466.9(3)
Z	4
ρ_{calc}g/cm^3	1.253
μ/mm^{-1}	3.033
F(000)	2540.0
Crystal size/mm^3	0.25 × 0.1 × 0.05
Radiation	Cu Kα (λ = 1.54184)
2Θ range for data collection/°	9.398 to 146.604
Index ranges	-21 ≤ h ≤ 21, -12 ≤ k ≤ 11, -46 ≤ l ≤ 46
Reflections collected	96867
Independent reflections	12835 [R_{int} = 0.0848, R_{sigma} = 0.0477]
Data/restraints/parameters	12835/1/685
Goodness-of-fit on F^2	1.049
Final R indexes [I>=2σ (I)]	R_1 = 0.0759, wR_2 = 0.2140
Final R indexes [all data]	R_1 = 0.0935, wR_2 = 0.2262
Largest diff. peak/hole / e Å^{-3}	0.51/-0.64

In compound **4e**, the asymmetric unit was seen to contain one molecule of the phosphine complex, one molecule of *p*-toluenesulfonic acid and some solvent (dichloromethane). The latter was disordered in the main and was addressed using the solvent mask mentioned previously. Allowance has been made for two solvent molecules per asymmetric unit, in the formula as presented. H1 and H2 were located and refined without restraints.

Table S10: Crystal Data and Structure Refinement for **4f**.

Compound	**4f**
Empirical formula	$C_{63.5}H_{49}Cl_3F_3O_2P_3$
Formula weight	1100.28
Temperature/K	150.00(10)
Crystal system	monoclinic
Space group	$P2_1/n$
a/Å	14.4850(2)
b/Å	17.0162(2)
c/Å	21.8394(3)
α/°	90
β/°	99.526(1)
γ/°	90
Volume/Å^3	5308.74(12)
Z	4
ρ_{calc}g/cm^3	1.377
μ/mm^{-1}	2.878
F(000)	2276.0
Crystal size/mm^3	0.158 × 0.132 × 0.09
Radiation	Cu Kα (λ = 1.54184)
2Θ range for data collection/°	6.62 to 146.58
Index ranges	-17 ≤ h ≤ 17, -21 ≤ k ≤ 20, -26 ≤ l ≤ 27
Reflections collected	52154
Independent reflections	10602 [R_{int} = 0.0444, R_{sigma} = 0.0334]
Data/restraints/parameters	10602/127/709
Goodness-of-fit on F^2	1.043
Final R indexes [I>=2σ (I)]	R_1 = 0.0641, wR_2 = 0.1861
Final R indexes [all data]	R_1 = 0.0731, wR_2 = 0.1970
Largest diff. peak/hole / e Å^{-3}	1.70/-0.85

In the structure of **4f**, the asymmetric unit is host to one molecule of the phosphorus containing species, an ordered molecule of dichloromethane and a disordered region of solvent that approximate to half of an additional CH_2Cl_2 entity. Disorder prevailed in the main feature, with the fluorine atoms each split over 2 sites in a 63:37 ratio and the phenyl ring based on C45 being modelled to take account of 83:17 disorder. This latter split of electron density impacted on the half-occupancy solvent moiety, to the extent that the associated (smeared) electron-density was ultimately treated using the Olex-2 solvent mask, with allowance being made for the squeezed solvent in the formula as presented. Distance and ADP restraints were employed, on merit, in disordered regions to assist convergence. The highest residual electron density peak is spurious.

Table S11. Summary of select bond distances/angles for solid-state structures **3**, **4a**, **4b**, **4e** and **4f**. Atom labelling has been generalized for ease of comparison.

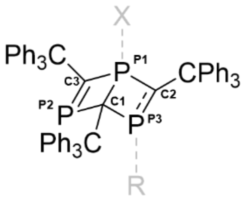

Structure	P1-C1 / Å	P1-C2 / Å	P1-C3 / Å	P2-C3 / Å	P3-C2 / Å	C2-P1-C3 / °	C1-P1-X / °
**3**	1.871(1)	1.829(1)	1.848(2)	1.689(1)	1.697(1)	108.78(7)	-
**4a**	1.818(5)	1.782(6)	1.771(6)	1.706(5)	1.711(5)	122.2(3)	125.5(3)
**4b**	1.798(2)	1.791(2)	1.738(2)	1.708(2)	1.747(2)	125.4(1)	117.70(8)
**4e**	1.920(3)	1.830(3)	1.926(3)	1.690(3)	1.814(3)	98.4(1)	-
**4f**	1.908(3)	1.829(3)	1.906(3)	1.686(3)	1.860(3)	97.2(1)	-

12. Quantum-Chemical Details

12.1. Computational Methods

Optimizations of molecular geometries and Hessian calculations were carried out with the Gaussian 16 program[15] in conjunction with the SMD continuum solvent model[16] uitlizing acetonitrile as the solvent. The PBE0[17,18] level of hybrid density functional theory (DFT) and the split-valence double-zeta def2-SVP[19] basis set were employed together with D3 atom-pairwise dispersion corrections including Becke-Johnson damping,[20–24] abbreviated PBE0-D3BJ(SMD)/def2-SVP. The 60 core electrons of Au were described by the quasi-relativistic effective core potential of Andrae *et al.*[25] Thermal contributions to enthalpies and Gibbs energies at 298.15 K were obtained at this level of DFT within the ideal gas, rigid-rotor, and harmonic oscillator approximations. All stationary points were characterized as minima or transition structures by eigenvalue analysis of the computed Hessians. Connections between transition states and the corresponding minima were confirmed by intrinsic reaction coordinate (IRC) calculations or by distortion of the transition state structures along the eigenvectors of imaginary frequency modes followed by unconstrained structure optimization. For all transition structures, additional broken-symmetry (BS) unrestricted Kohn–Sham (UKS) geometry optimizations were performed to obtain a molecular structure for these species, which were also used for subsequent single-point calculations. Singlet energies for these species were obtained after spin-projection according to Yamaguchi:[26–28]

$$E_\mathrm{S} = E_\mathrm{T} - 2\,\frac{E_\mathrm{T} - E_\mathrm{BS}}{\langle S^2\rangle_\mathrm{T} - \langle S^2\rangle_\mathrm{BS}} \tag{1}$$

Single-point energy calculations with the triple-zeta def2-TZVP basis set[19] were employed for improved relative energies. The final Gibbs energies are thus obtained at the PBE0-D3BJ(SMD)/def2-TZVP // def2-SVP level.

The half-life $t_{1/2}$ of an intermediate was estimated from simple considerations according to the Eyring equation assuming first-order kinetics.

$$k = \kappa \cdot \frac{k_\mathrm{B}\,T}{h} \cdot \exp -\frac{\Delta^\ddagger G}{R\,T} \tag{2}$$

$$t_{\frac{1}{2}} = \frac{\ln(2)}{k} \tag{3}$$

Employing a temperature of 110 °C (T = 383 K) along with a transmission coefficient set to unity ($\kappa = 1$), the half-life for the intermediate associated with a barrier of $\Delta^\ddagger G(\mathbf{3} \rightarrow \mathbf{3}')$ = 32 kcal mol^{-1} amounts to $t_{1/2}$ = 1.8 days, while that for the reverse barrier of $\Delta^\ddagger G(\mathbf{3} \leftarrow \mathbf{3}')$ = 34 kcal mol^{-1} results in $t_{1/2}$ = 25.3 days.

12.2. Energies

Table S12: Total energies (E_{tot}), thermal contributions to enthalpies (H_{tot}) and Gibbs energies (G_{tot}), and $\langle S^2 \rangle$ expectation values of species studied. Relative energies E_{rel}, enthalpies H_{rel} and Gibbs energies G_{rel} given in kcal mol^{-1}.

	PBE0-D3BJ(SMD)/def2-SVP							PBE0-D3BJ(SMD)/def2-TZVP // def2-SVP				
Species[a]	$\langle S^2 \rangle$	E_{tot}	H_{tot}	G_{tot}	E_{rel}	H_{rel}	G_{rel}	$\langle S^2 \rangle$	E_{tot}	E_{rel}	H_{rel}	G_{rel}
^{1}**3**	0.000	−3332.649 437	0.923 14	0.783 28	0.0	0.0	0.0	0.000	−3335.379 600	0.0	0.0	0.0
^{1}**TS(3–3′)**	0.000	−3332.590 913	0.920 33	0.779 80	36.7	35.0	34.5	0.000	−3335.325 295	34.1	32.3	31.9
^{3}**TS(3–3′)**	2.016	−3332.585 035						2.018	−3335.314 145			
^{BS}**TS(3–3′)**	0.954	−3332.589 151	0.920 47	0.781 21	37.8	36.2	36.5	0.955	−3335.318 107	38.6	36.9	37.3
^{S}**TS(3–3′)**	0.954	−3332.592 782	0.920 47	0.781 21	35.6	33.9	34.3	0.955	−3335.321 601	36.4	34.7	35.1
^{1}**3′**	0.000	−3332.646 325	0.922 10	0.777 76	2.0	1.3	−1.5	0.000	−3335.376 531	1.9	1.3	−1.5
^{1}**TS(3′–3′a)**	0.000	−3332.583 794	0.920 46	0.779 76	41.2	39.5	39.0	0.000	−3335.317 492	39.0	37.3	36.8
^{3}**TS(3′–3′a)**	2.065	−3332.580 754						2.065	−3335.313 084			
^{BS}**TS(3′–3′a)**	0.711	−3332.587 487	0.920 19	0.778 87	38.9	37.0	36.1	0.691	−3335.320 273	37.2	35.4	34.5
^{S}**TS(3′–3′a)**	0.711	−3332.590 697	0.920 19	0.778 87	36.9	35.0	34.1	0.691	−3335.323 551	35.2	33.3	32.4
^{1}**3′a**	0.000	−3332.645 181	0.923 12	0.784 39	2.7	2.7	3.4	0.000	−3335.371 355	5.2	5.2	5.9
^{1}**TS(3′a–3′b)**	0.000	−3332.574 627	0.920 82	0.784 08	46.9	45.5	47.5	0.000	−3335.301 586	49.0	47.5	49.5
^{3}**TS(3′a–3′b)**	2.033	−3332.587 968						2.033	−3335.312 571			
^{BS}**TS(3′a–3′b)**	0.958	−3332.590 624	0.921 14	0.784 29	36.9	35.7	37.5	0.951	−3335.315 571	40.2	38.9	40.8
^{S}**TS(3′a–3′b)**	0.958	−3332.592 909	0.921 14	0.784 29	35.5	34.2	36.1	0.951	−3335.318 119	38.6	37.3	39.2
^{1}**3′b**	0.000	−3332.635 753	0.923 55	0.788 03	8.6	8.9	11.6	0.000	−3335.361 209	11.5	11.8	14.5
^{1}**TS(3′b–3″)**	0.000	−3332.594 755	0.921 44	0.784 55	34.3	33.3	35.1	0.000	−3335.321 941	36.2	35.1	37.0
^{3}**TS(3′b–3″)**	2.012	−3332.602 188						2.015	−3335.326 985			
^{BS}**TS(3′b–3″)**	0.883	−3332.608 925	0.921 34	0.785 15	25.4	24.3	26.6	0.882	−3335.333 670	28.8	27.7	30.0
^{S}**TS(3′b–3″)**	0.883	−3332.614 122	0.921 34	0.785 15	22.2	21.0	23.3	0.882	−3335.338 786	25.6	24.5	26.8
^{1}**3″**	0.000	−3332.672 471	0.923 61	0.785 81	−14.5	−14.2	−12.9	0.000	−3335.400 785	−13.3	−13.0	−11.7
^{1}**AuCl**	0.000	−595.663 134	0.004 44	−0.024 30				0.000	−595.832 458			
^{1}**ClAu-3**	0.000	−3928.409 259	0.929 91	0.782 95	−60.7[b]	−59.2[b]	−45.6[b]	0.000	−3931.310 190	−61.6[b]	−60.1[b]	−46.5[b]
^{1}**ClAu-3a**	0.000	−3928.399 861	0.929 84	0.779 45	−54.8[b]	−53.4[b]	−41.9[b]	0.000	−3931.302 769	−56.9[b]	−55.5[b]	−44.1[b]
^{1}**ClAu-3b**	0.000	−3928.399 328	0.929 63	0.779 77	−54.4[b]	−53.2[b]	−41.4[b]	0.000	−3931.301 282	−56.0[b]	−54.7[b]	−42.9[b]
^{1}**ClAu-3′**	0.000	−3928.398 065	0.928 75	0.776 09	−55.6[c]	−54.2[c]	−41.4[c]	0.000	−3931.299 390	−56.7[c]	−55.3[c]	−42.5[c]
^{1}**ClAu-3″**	0.000	−3928.412 292	0.929 86	0.780 01	−48.1[d]	−47.0[d]	−36.5[d]	0.000	−3931.312 903	−50.0[d]	−48.9[d]	−38.4[d]
^{1}**ClAu-3″a**	0.000	−3928.421 648	0.930 20	0.783 94	−54.0[d]	−52.6[d]	−39.9[d]	0.000	−3931.321 269	−55.2[d]	−53.9[d]	−41.2[d]
^{1}**ClAu-3″b**	0.000	−3928.425 972	0.929 92	0.782 26	−56.7[d]	−55.5[d]	−43.7[d]	0.000	−3931.325 736	−58.0[d]	−56.9[d]	−45.0[d]

[a] State denoted by superscript: ^{1} (RKS singlet); ^{3} (UKS triplet); ^{BS} (UKS/broken-symmetry singlet); ^{S} (spin-projected BS singlet); triplet energies were computed for spin projection, i.e., at the corresponding UKS/broken-symmetry singlet geometries.
[b] Relative energy with respect to ^{1}**AuCl** + ^{1}**3**. [c] Relative energy with respect to ^{1}**AuCl** + ^{1}**3′**. [d] Relative energy with respect to ^{1}**AuCl** + ^{1}**3″**.

Table S13: Comparison of the effect of 2,4,6-substituents, R, on the relative stabilities of Dewar phosphabenzene **3**, with respect to **3′**, and **3″**, computed for R = H, Ph, CPh_3 — i.e., isomers of 1,3,5-triphosphabenzene (R = H), 2,4,6-triphenyl-1,3,5-triphosphabenzene (R = Ph), and 2,4,6-tris(triphenylmethyl)-1,3,5-triphosphabenzene (R = CPh_3). Total energies (E_{tot}), thermal contributions to enthalpies (H_{tot}) and Gibbs energies (G_{tot}), and $\langle S^2 \rangle$ expectation values for ^{R}**3**, ^{R}**3′**, and ^{R}**3″**. Relative energies E_{rel}, enthalpies H_{rel} and Gibbs energies G_{rel} given in kcal mol^{-1}.

	PBE0-D3BJ(SMD)/def2-SVP							PBE0-D3BJ(SMD)/def2-TZVP // def2-SVP				
Species	$\langle S^2 \rangle$	E_{tot}	H_{tot}	G_{tot}	E_{rel}	H_{rel}	G_{rel}	$\langle S^2 \rangle$	E_{tot}	E_{rel}	H_{rel}	G_{rel}
^{H}**3**	0.000	−1139.184 981	0.058 21	0.021 47	0.0	0.0	0.0	0.000	−1139.671 163	0.0	0.0	0.0
^{H}**3′**	0.000	−1139.221 662	0.059 01	0.023 28	−23.0	−22.5	−21.9	0.000	−1139.701 092	−18.8	−18.3	−17.7
^{H}**3″**	0.000	−1139.224 243	0.059 64	0.023 76	−24.6	−23.7	−23.2	0.000	−1139.704 938	−21.2	−20.3	−19.8
^{Ph}**3**	0.000	−1831.135 136	0.317 95	0.244 44	0.0	0.0	0.0	0.000	−1832.328 838	0.0	0.0	0.0
^{Ph}**3′**	0.000	−1831.146 601	0.317 69	0.244 65	−7.2	−7.4	−7.1	0.000	−1832.337 337	−5.3	−5.5	−5.2
^{Ph}**3″**	0.000	−1831.168 232	0.318 51	0.246 11	−20.8	−20.4	−19.7	0.000	−1832.361 218	−20.3	−20.0	−19.3
^{CPh_3}**3**	0.000	−3332.649 437	0.923 14	0.783 28	0.0	0.0	0.0	0.000	−3335.379 600	0.0	0.0	0.0
^{CPh_3}**3′**	0.000	−3332.646 325	0.922 10	0.777 76	2.0	1.3	−1.5	0.000	−3335.376 531	1.9	1.3	−1.5
^{CPh_3}**3″**	0.000	−3332.672 471	0.923 61	0.785 81	−14.5	−14.2	−12.9	0.000	−3335.400 785	−13.3	−13.0	−11.7

12.3. Spin densities

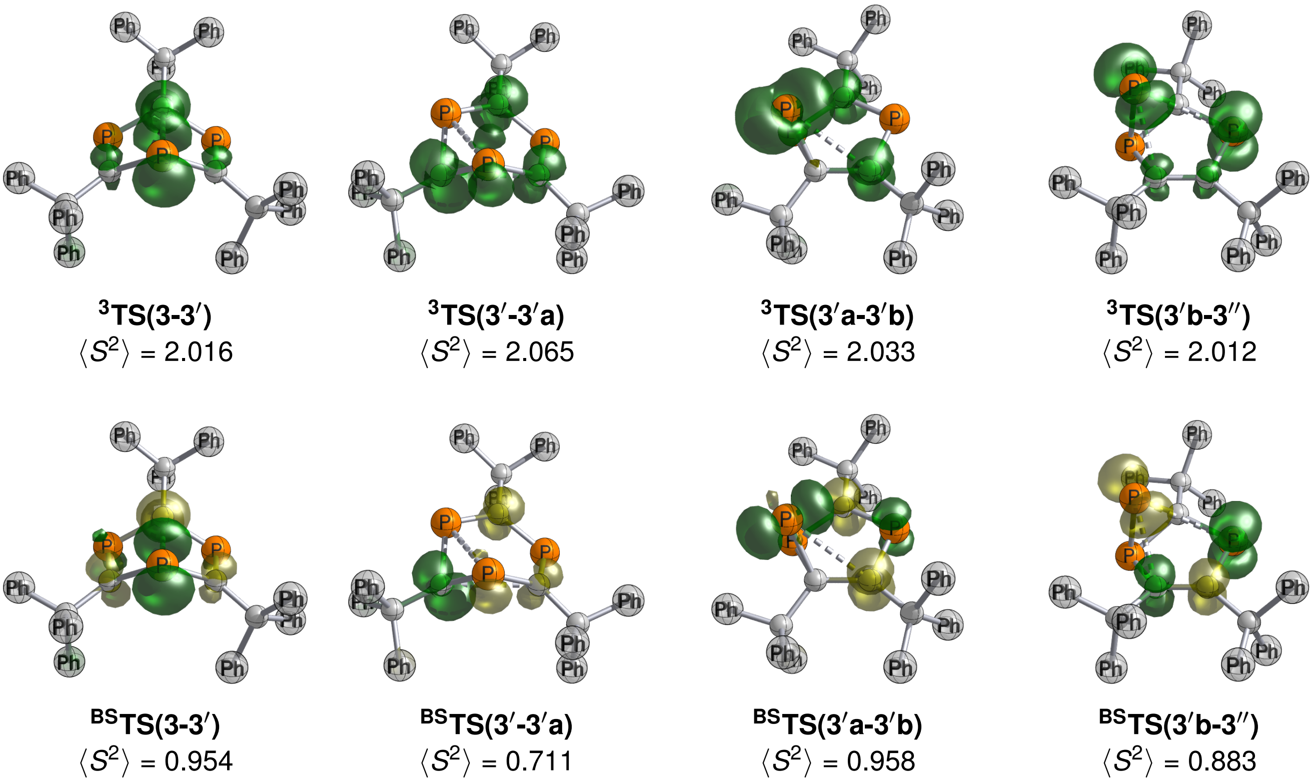

Figure S1: Triplet (top) and broken-symmetry singlet (bottom) spin density distributions computed at the PBE0-D3BJ(SMD)/def2-TZVP level for **TS(3-3′)**, **TS(3′-3′a)**, **TS(3′a-3′b)**, **TS(3′b-3″)**; α-spin: green, β-spin: yellow; isocontour surfaces at 0.01 a_0^{-3}; phenyl groups are not shown; PBE0-D3BJ(SMD)/def2-SVP optimized broken-symmetry singlet structures.

12.4. Frontier molecular orbitals for 3

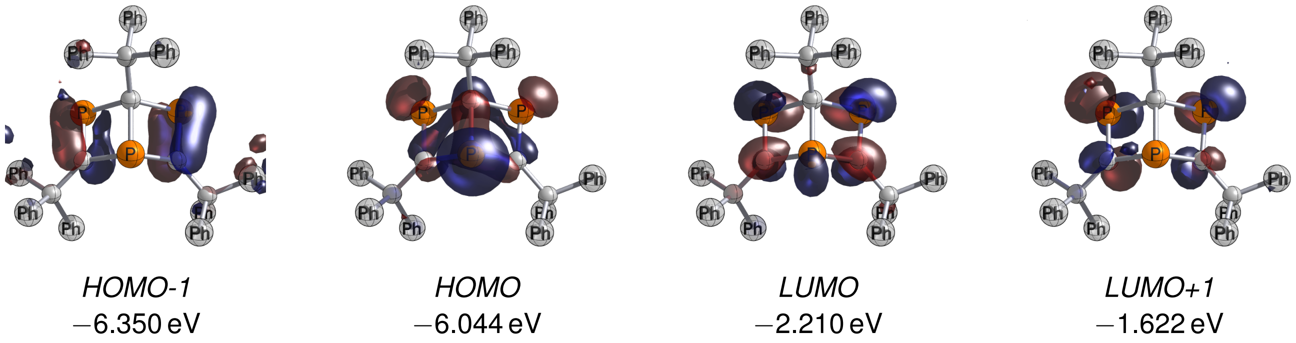

Figure S2: Selected frontier molecular orbitals for **3** with orbital energy eigenvalues computed at the PBE0-D3BJ(SMD)/def2-TZVP level; isocontour surfaces at 0.05 $a_0^{-3/2}$; phenyl groups are not shown; PBE0-D3BJ(SMD)/def2-SVP optimized singlet structure.

12.5. Frontier molecular orbitals for 3′

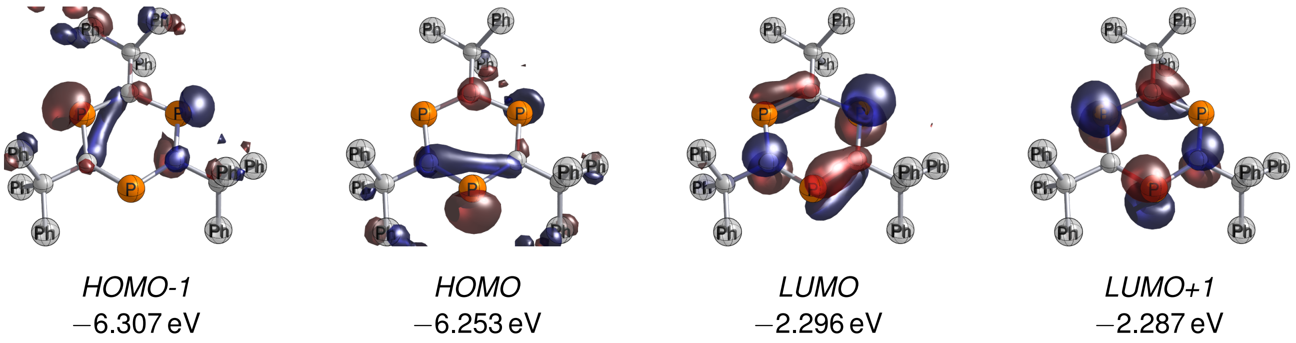

Figure S3: Selected frontier molecular orbitals for **3′** with orbital energy eigenvalues computed at the PBE0-D3BJ(SMD)/def2-TZVP level; isocontour surfaces at 0.05 $a_0^{-3/2}$; phenyl groups are not shown; PBE0-D3BJ(SMD)/def2-SVP optimized singlet structure.

12.6. Frontier molecular orbitals for 3″

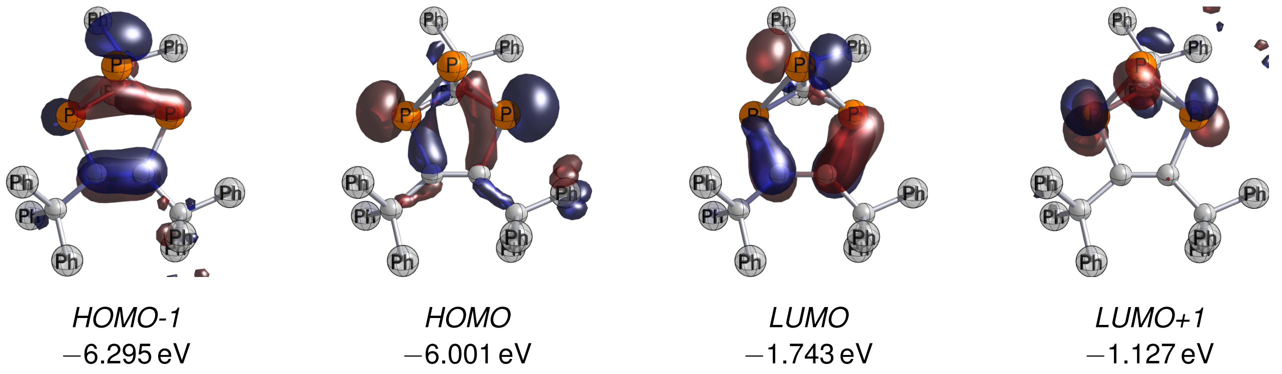

Figure S4: Selected frontier molecular orbitals for **3″** with orbital energy eigenvalues computed at the PBE0-D3BJ(SMD)/def2-TZVP level; isocontour surfaces at 0.05 $a_0^{-3/2}$; phenyl groups are not shown; PBE0-D3BJ(SMD)/def2-SVP optimized singlet structure.

12.7. Au complexation with 3

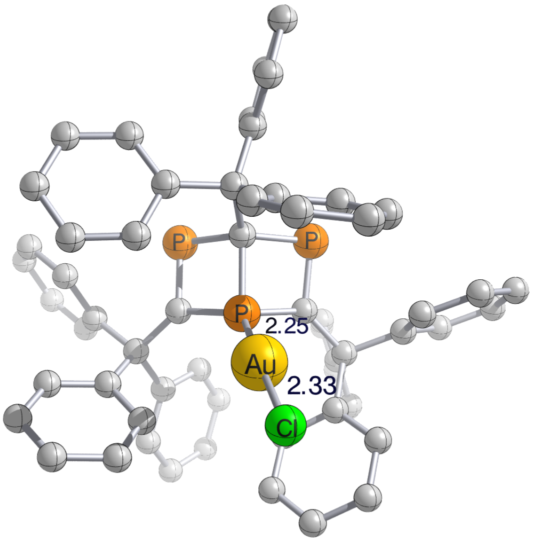

BDFE(**ClAu–3**) = 46.5 kcal mol^{-1}

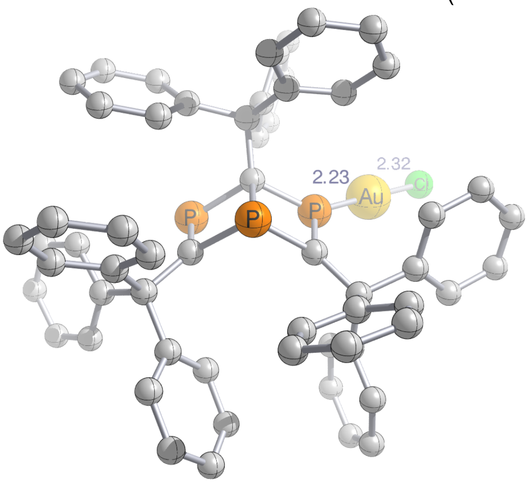

BDFE(**ClAu–3a**) = 44.1 kcal mol^{-1}

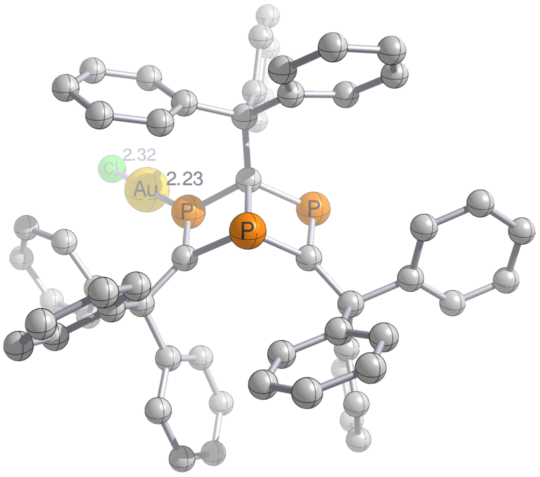

BDFE(**ClAu–3b**) = 42.9 kcal mol^{-1}

Figure S5: PBE0-D3BJ(SMD)/def2-SVP optimized singlet structures of (Cl)Au(**3**) complexes with Au–P bond dissociation free energy, BDFE ($\Delta_r G^{298}$), computed at the PBE0-D3BJ(SMD)/def2-TZVP // def2-SVP level; hydrogen atoms are not shown.

12.8. Au complexation with 3′

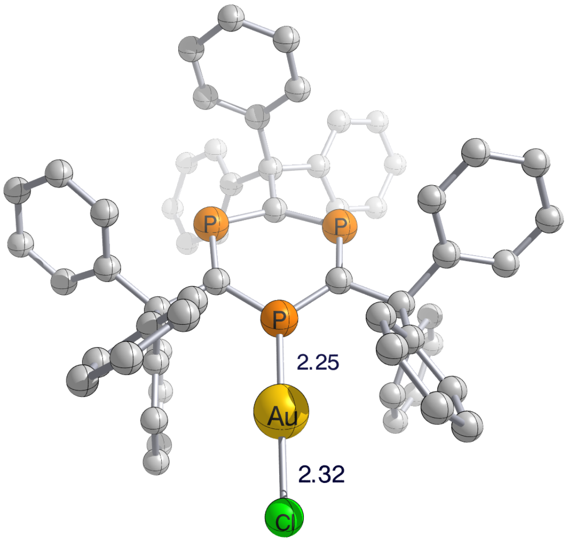

BDFE(**ClAu–3′**) = 42.5 kcal mol^{-1}

Figure S6: PBE0-D3BJ(SMD)/def2-SVP optimized singlet structure of (Cl)Au(**3′**) complexes with Au–P bond dissociation free energy, BDFE ($\Delta_r G^{298}$), computed at the PBE0-D3BJ(SMD)/def2-TZVP // def2-SVP level; hydrogen atoms are not shown.

12.9. Au complexation with 3″

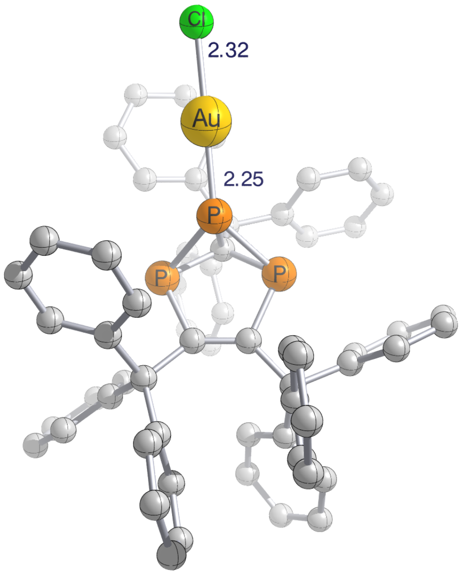

BDFE(**ClAu–3″**) = 38.4 kcal mol^{-1}

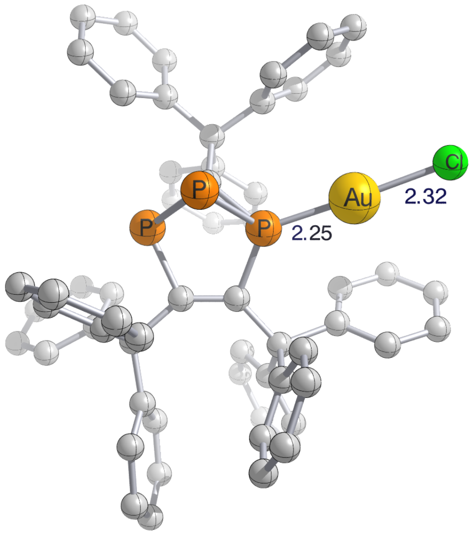

BDFE(**ClAu–3″a**) = 41.2 kcal mol^{-1}

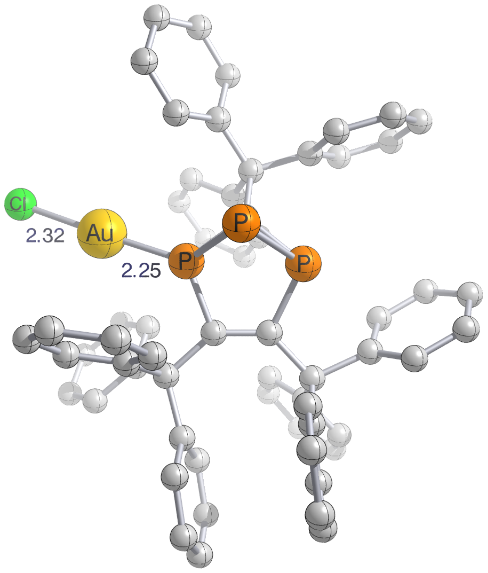

BDFE(**ClAu–3″b**) = 42.9 kcal mol^{-1}

Figure S7: PBE0-D3BJ(SMD)/def2-SVP optimized singlet structures of (Cl)Au(**3″**) complexes with Au–P bond dissociation free energy, BDFE ($\Delta_r G^{298}$), computed at the PBE0-D3BJ(SMD)/def2-TZVP // def2-SVP level; hydrogen atoms are not shown.

13. References

[1] K. J. Gallagher, R. L. Webster, “Room temperature hydrophosphination using a simple iron salen pre-catalyst”, *Chem. Commun.* **2014**, *50*, 12 109–12 111.

[2] J. J. Eisch, C. A. Kovacs, P. Chobe, “Carbon-Skeletal [1,2] Anionic Rearrangements and the π-Orbital Overlap Constraint: The Question of Nucleophilic Attack versus Electron Transfer”, *J. Org. Chem.* **1989**, *54* (6), 1275–1284.

[3] J. G. Cordaro, D. Stein, H. Grützmacher, “A Synthetic Cycle for the Ruthenium-Promoted Formation of 1*H*-Phosphindoles from Phosphaalkynes”, *J. Am. Chem. Soc.* **2006**, *128* (46), 14 962–14 971.

[4] M. Trincado, A. J. Rosenthal, M. Vogt, H. Grützmacher, “η^2-Coordination of a Phosphaalkyne to an Amino Olefin Nickel Complex and Regioselective Catalyzed Cyclooligomerization to Dewar 1,3,5-Triphosphabenzene”, *Eur. J. Inorg. Chem.* **2014**, *2014* (10), 1599–1604.

[5] J. Burés, “Variable Time Normalization Analysis: General Graphical Elucidation of Reaction Orders from Concentration Profiles”, *Angew. Chem.* **2016**, *128* (52), 16 318–16 321; *Angew. Chem. Int. Ed.*, **2016**, *55* (52), 16 084–16 087.

[6] J. Burés, “A Simple Graphical Method to Determine the Order in Catalyst”, *Angew. Chem.* **2016**, *128* (6), 2068–2071; *Angew. Chem. Int. Ed.*, **2016**, *55* (6), 2028–2031.

[7] T. Allspach, M. Regitz, G. Becker, W. Becker, “Adamant-1-ylmethylidynephosphine, A New, Stable Phosphaalkyne”, *Synthesis* **1986**, *1986* (1), 31–36.

[8] S. M. Mansell, M. Green, C. A. Russell, “Coordination chemistry of trimethylsilylphosphaalkyne: a phosphaalkyne bearing a reactive substituent”, *Dalton Trans.* **2012**, *41*, 14 360–14 368.

[9] A. Maleckis, J. W. Kampf, M. S. Sanford, “A Detailed Study of Acetate-Assisted C–H Activation at Palladium(IV) Centers”, *J. Am. Chem. Soc.* **2013**, *135* (17), 6618–6625.

[10] J. Zhang, L. Hu, K. Zhang, J. Liu, X. Li, H. Wang, Z. Wang, H. H. Y. Sung, I. D. Williams, Z. Zeng, J. W. Y. Lam, H. Zhang, B. Z. Tang, “How to Manipulate Through-Space Conjugation and Clusteroluminescence of Simple AIEgens with Isolated Phenyl Rings”, *J. Am. Chem. Soc.* **2021**, *143* (25), 9565–9574.

[11] G. M. Sheldrick, “*SHELXT* — Integrated space-group and crystal-structure determination”, *Acta Cryst. A* **2015**, *71* (1), 3–8.

[12] G. M. Sheldrick, “Crystal structure refinement with *SHELXL*”, *Acta Cryst. C* **2015**, *71* (1), 3–8.

[13] O. V. Dolomanov, L. J. Bourhis, R. J. Gildea, J. A. K. Howard, H. Puschmann, “OLEX2: a complete structure solution, refinement and analysis program”, *J. Appl. Cryst.* **2009**, *42* (2), 339–341.

[14] A. L. Spek, “Single-crystal structure validation with the program *PLATON*”, *J. Appl. Cryst.* **2003**, *36* (1), 7–13.

[15] “Gaussian 16, Revision C.01”, M. J. Frisch, G. W. Trucks, H. B. Schlegel, G. E. Scuseria, M. A. Robb, J. R. Cheeseman, G. Scalmani, V. Barone, G. A. Petersson, H. Nakatsuji, X. Li, M. Caricato, A. V. Marenich, J. Bloino, B. G. Janesko, R. Gomperts, B. Mennucci, H. P. Hratchian, J. V. Ortiz, A. F. Izmaylov, J. L. Sonnenberg, D. Williams-Young, F. Ding, F. Lipparini, F. Egidi, J. Goings, B. Peng, A. Petrone, T. Henderson, D. Ranasinghe, V. G. Zakrzewski, J. Gao, N. Rega, G. Zheng, W. Liang, M. Hada, M. Ehara, K. Toyota, R. Fukuda, J. Hasegawa, M. Ishida, T. Nakajima, Y. Honda, O. Kitao, H. Nakai, T. Vreven, K. Throssell, J. A. Montgomery, Jr., J. E. Peralta, F. Ogliaro, M. J. Bearpark, J. J. Heyd, E. N. Brothers, K. N. Kudin, V. N. Staroverov, T. A. Keith, R. Kobayashi, J. Normand, K. Raghavachari, A. P. Rendell, J. C. Burant, S. S. Iyengar, J. Tomasi, M. Cossi, J. M. Millam, M. Klene, C. Adamo, R. Cammi, J. W. Ochterski, R. L. Martin, K. Morokuma, Ö. Farkas, J. B. Foresman, D. J. Fox (Gaussian, Inc., Wallingford, CT), **2019**, see `http://www.gaussian.com`.

[16] A. V. Marenich, C. J. Cramer, D. G. Truhlar, “Universal Solvation Model Based on Solute Electron Density and on a Continuum Model of the Solvent Defined by the Bulk Dielectric Constant and Atomic Surface Tensions”, *J. Phys. Chem. B* **2009**, *113* (18), 6378–6396.

[17] J. P. Perdew, M. Ernzerhof, K. Burke, “Rationale for mixing exact exchange with density functional approximations”, *J. Chem. Phys.* **1996**, *105* (22), 9982–9985.

[18] C. Adamo, V. Barone, “Toward reliable density functional methods without adjustable parameters: The PBE0 model”, *J. Chem. Phys.* **1999**, *110* (13), 6158–6170.

[19] F. Weigend, R. Ahlrichs, “Balanced basis sets of split valence, triple zeta valence and quadruple zeta valence quality for H to Rn: Design and assessment of accuracy”, *Phys. Chem. Chem. Phys.* **2005**, *7* (18), 3297–3305.

[20] S. Grimme, J. Antony, S. Ehrlich, H. Krieg, “A consistent and accurate *ab initio* parametrization of density functional dispersion correction (DFT-D) for the 94 elements H–Pu”, *J. Chem. Phys.* **2010**, *132* (15), 154 104.

[21] A. D. Becke, E. R. Johnson, “A density-functional model of the dispersion interaction”, *J. Chem. Phys.* **2005**, *123* (15), 154 101.

[22] E. R. Johnson, A. D. Becke, “A post-Hartree–Fock model of intermolecular interactions”, *J. Chem. Phys.* **2005**, *123* (2), 024 101.

[23] E. R. Johnson, A. D. Becke, “A post-Hartree–Fock model of intermolecular interactions: Inclusion of higher-order corrections”, *J. Chem. Phys.* **2006**, *124* (17), 174 104.

[24] S. Grimme, S. Ehrlich, L. Goerigk, “Effect of the damping function in dispersion corrected density functional theory”, *J. Comput. Chem.* **2011**, *32* (7), 1456–1465.

[25] D. Andrae, U. Häußermann, M. Dolg, H. Stoll, H. Preuß, “Energy-adjusted *ab initio* pseudopotentials for the second and third row transition elements”, *Theor. Chem. Acc.* **1990**, *77* (2), 123–141.

[26] T. Soda, Y. Kitagawa, T. Onishi, Y. Takano, Y. Shigeta, H. Nagao, Y. Yoshioka, K. Yamaguchi, “*Ab initio* computations of effective exchange integrals for H–H, H–He–H and Mn_2O_2 complex: comparison of broken-symmetry approaches”, *Chem. Phys. Lett.* **2000**, *319* (3-4), 223–230.

[27] F. Neese, “Prediction of molecular properties and molecular spectroscopy with density functional theory: From fundamental theory to exchange-coupling”, *Coord. Chem. Rev.* **2009**, *253* (5), 526–563.

[28] J. P. Malrieu, R. Caballol, C. J. Calzado, C. de Graaf, N. Guihéry, “Magnetic Interactions in Molecules and Highly Correlated Materials: Physical Content, Analytical Derivation, and Rigorous Extraction of Magnetic Hamiltonians”, *Chem. Rev.* **2014**, *114* (1), 429–492.

A. Appendix

Cartesian coordinates of PBE0-D3BJ(SMD)/def2-SVP geometries (Å)

108

3 (^{1}A, C_1): E_{tot}(RPBE0-D3(BJ)(Acetonitrile)/def2SVP) = -3332.64943731 $\langle S^2 \rangle$ = 0

P	-1.782129036541	0.214166244749	-1.300342525840
P	1.053894669545	1.723037569847	-1.226713671583
P	0.018622628927	0.183313192067	0.670997473860
C	-0.650943869022	1.485912167467	-0.495295922149
C	1.555511202508	0.402879816784	-0.286645610835
C	-1.133457170650	-0.941177938836	-0.250035950499
C	-1.411791118879	2.745634265793	0.025440084141
C	-1.694366900154	3.719289664995	-1.128612801739
C	-2.749741996076	2.241898619951	0.613900307826
C	-0.521797247133	3.488800291085	1.046012731489
C	-2.149995732006	5.011667254580	-0.824453621472
C	-2.772691224756	1.347849421775	1.699762492943
C	0.579141699932	4.216491765979	0.561313065683
C	-1.546494240883	3.374266375439	-2.473843493859
C	-3.977419861016	2.541482417226	0.006974420079
C	-0.746330432959	3.506943901123	2.426406466445
C	-2.448647591720	5.924785155440	-1.829869796310
C	-3.961814168045	0.790380537775	2.161584274998
C	1.440398999743	4.895501793452	1.418343631799
C	-1.840239084315	4.290665174631	-3.488126807537
C	-5.172614509621	1.984307270552	0.466753226583
C	0.116981808515	4.185003140267	3.291423961015
C	-2.292235139623	5.567878269426	-3.171854579840
C	-5.173903898401	1.107692683068	1.547802827918
C	1.219715805361	4.874606213959	2.795788268199
H	-2.275500233556	5.303433466227	0.221403757412
H	-1.843748438413	1.052518668391	2.190468138222
H	0.751982559263	4.287320663847	-0.514933507115
H	-1.199704931616	2.379998107563	-2.762668495548
H	-4.012231169936	3.209327972153	-0.853787727547
H	-1.614078826572	3.006539343897	2.852864707426
H	-2.803537122083	6.924233981556	-1.565193761916
H	-3.934509763621	0.093005956632	3.002825422296
H	2.283003626579	5.452421604219	1.001953351211
H	-1.710726067460	3.994531224037	-4.532165336617
H	-6.108853003496	2.239241545801	-0.036492140135
H	-0.089146128912	4.175761957082	4.364863114616
H	-2.520796866434	6.285064792953	-3.964263968578
H	-6.108149708662	0.667773226327	1.905479370604
H	1.892629979788	5.406447867429	3.473212177898
C	2.956064404685	-0.156541868224	-0.085296036291
C	3.934509319757	1.035875329502	0.008666761917
C	2.987206040508	-0.973987938949	1.217976421934
C	3.388314918057	-1.061749513529	-1.259963048564
C	5.116806522969	1.105042083542	-0.732512543213
C	2.091033774960	-2.041626251546	1.352761981751
C	3.038211582422	-0.762532933765	-2.580567479439
C	3.654433181491	2.076706705178	0.908659734756
C	3.890714462019	-0.735360831268	2.256502600074
C	4.263585088661	-2.132295045226	-1.037544528083
C	5.993853847214	2.183792502399	-0.584596499427
C	2.075931679479	-2.833200194633	2.497586901357
C	3.552226049683	-1.499429509651	-3.646827558924
C	4.532340156286	3.143759009679	1.067681840433
C	3.880973485388	-1.529328149367	3.406633451750
C	4.777720278217	-2.874030426712	-2.100972324956
C	5.708591512875	3.205159198020	0.316136305131
C	2.971786855792	-2.576408301944	3.536490700991
C	4.427789289968	-2.559330821036	-3.413069312461
H	5.368786445754	0.313174222809	-1.438880147531
H	1.412249760014	-2.280998278544	0.531723735230
H	2.361427853553	0.067437971514	-2.793144416875
H	2.735759400641	2.046060852052	1.499708459308
H	4.618996949414	0.072535325487	2.177708862888
H	4.563839583210	-2.386510097858	-0.019532381570
H	6.908106899844	2.217004184717	-1.182920663386
H	1.362290252441	-3.657283065397	2.572243306548
H	3.262215121368	-1.240305630419	-4.668363528144

H	4.295012148252	3.934966240634	1.782903901918
H	4.596572128384	-1.321819481380	4.206541819993
H	5.460450732257	-3.703088940719	-1.897444423580
H	6.395946805521	4.046687617241	0.434575861500
H	2.964792334134	-3.194196070843	4.438031071623
H	4.830055259769	-3.139151194614	-4.247604592612
C	-1.588432113709	-2.388997360865	-0.109245104571
C	-0.456447269645	-3.334628888460	-0.543267356771
C	-2.031941613859	-2.728277289329	1.330032209894
C	-2.808621053467	-2.548611696006	-1.045446320059
C	-0.173048963317	-4.521526877744	0.140416456616
C	-1.405741092957	-2.193380067363	2.460041166325
C	-2.814648100714	-3.369420732044	-2.174586608668
C	0.297038232406	-3.027612315136	-1.684655215474
C	-3.048061164853	-3.673775878947	1.537037316928
C	-3.973619482662	-1.818503401609	-0.749448869456
C	0.841121392856	-5.374465422284	-0.298497070405
C	-1.792669434018	-2.565050348368	3.748486219248
C	-3.943996613444	-3.448710210513	-2.995855501313
C	1.296474155883	-3.886208903536	-2.133318794799
C	-3.434478543948	-4.052976468720	2.821096552660
C	-5.097274088689	-1.894835307475	-1.565181701940
C	1.579918607045	-5.061431972849	-1.437085846800
C	-2.813317929007	-3.493865861022	3.937702054724
C	-5.086173295733	-2.710948045807	-2.698974081637
H	-0.740381538613	-4.787361148166	1.033265327412
H	-0.595018982786	-1.472855546235	2.356465343631
H	-1.935460542494	-3.963594936575	-2.426377578192
H	0.096868084266	-2.101411972825	-2.228458589294
H	-3.546434657524	-4.130821858451	0.680417768937
H	-4.001132142901	-1.186256295190	0.141134067810
H	1.052664685520	-6.289850415086	0.260282180058
H	-1.283751812046	-2.120758010239	4.607857410607
H	-3.922994391845	-4.099251350715	-3.874081230750
H	1.870895003599	-3.624091738998	-3.024045630707
H	-4.229938555944	-4.792255079074	2.946293312893
H	-5.987183496573	-1.312586928577	-1.311991588861
H	2.376900514890	-5.726000371094	-1.780276270620
H	-3.119648326837	-3.784156770848	4.945926097125
H	-5.966979805511	-2.772902248616	-3.343226022839

108

3′ (^{1}A, C_1): E_{tot}(RPBE0-D3(BJ)(Acetonitrile)/def2SVP) = -3332.64632469 $\langle S^2 \rangle$ = 0

P	-0.346038227814	1.773380906676	-0.240464352613
P	1.711565135775	-0.573015710518	0.267040729154
P	-1.340721119058	-1.220130488273	-0.226972334117
C	0.367504581471	-1.593069386045	-0.056863215792
C	1.212663140976	1.087655463932	-0.010472885445
C	-1.534320801107	0.484500758805	-0.162310137046
C	0.606954604847	-3.127616347624	0.037492690982
C	0.070230075186	-3.872006654131	-1.199166632743
C	-0.223680412286	-3.221094705178	-2.401886116773
H	-0.127612150673	-2.136007046594	-2.479154356649
C	-0.621281326805	-3.941987470790	-3.530408468632
H	-0.847435105005	-3.406578409547	-4.456183004412
C	-0.721050817677	-5.329557704967	-3.479425000694
H	-1.034680515669	-5.894374469260	-4.360982348895
C	-0.399374301060	-5.993814502898	-2.293650037893
H	-0.454471033611	-7.084304664195	-2.242422322169
C	0.000551451143	-5.273126572061	-1.172302869215
H	0.272088629530	-5.810293563818	-0.260329696933
C	-0.118131890322	-3.513892756882	1.343126855185
C	0.375396520722	-3.022247724770	2.562161979018
H	1.300039579425	-2.438960494829	2.577364821498
C	-0.306619038296	-3.237084936372	3.755616564122
H	0.104275178026	-2.844500697456	4.689302146781
C	-1.516551903285	-3.934770140136	3.757718186986
H	-2.059036389534	-4.097428010605	4.692485465957
C	-2.029216294391	-4.408452065919	2.553101426022
H	-2.982764941232	-4.942415286686	2.532784927309
C	-1.337325733896	-4.199064129672	1.357731556364
H	-1.774354670578	-4.561557677420	0.426815737927
C	2.104613683722	-3.500100048532	0.045334391817
C	2.910448823775	-3.075960170604	-1.025185517032
H	2.475741851226	-2.471452164034	-1.825060584311
C	4.255632521484	-3.421542335813	-1.095375182729
H	4.860848342111	-3.065644767528	-1.933133010160

C	4.826228714443	-4.224385541976	-0.104719549168
H	5.882796469372	-4.499057317453	-0.157209327650
C	4.029941446715	-4.683433916021	0.940225074896
H	4.455126829496	-5.329503268614	1.712699183546
C	2.680196964150	-4.326293447876	1.013206132688
H	2.077937913011	-4.706672493548	1.838996703028
C	2.382260528124	2.096284908309	0.180222480222
C	3.664456789725	1.680082906883	-0.564381619745
C	3.627650160153	0.867957857777	-1.704134505480
H	2.677465104472	0.462783202531	-2.058526935995
C	4.791382794869	0.575677841917	-2.417038915469
H	4.733401626296	-0.067459858608	-3.299002596660
C	6.015115825387	1.103631647216	-2.011289974060
H	6.928491269977	0.871158770134	-2.564643713936
C	6.058855230384	1.947314814967	-0.900262787654
H	7.007486960314	2.386490334543	-0.580874941716
C	4.895125683314	2.238975969407	-0.192547403106
H	4.946192537733	2.917153050391	0.662266527324
C	2.087748331058	3.486196089704	-0.417908171103
C	2.384041646315	4.671921720344	0.260265039633
H	2.780986168776	4.637552118760	1.275804007965
C	2.182887642393	5.913951580723	-0.346551485915
H	2.419014189442	6.825885230791	0.207972148576
C	1.687296324051	5.992559179904	-1.645485061374
H	1.523388231602	6.964841417985	-2.117039242324
C	1.416980495633	4.814617720568	-2.344902959564
H	1.044253083392	4.856027193462	-3.371607149259
C	1.627810478799	3.578660363903	-1.741398650895
H	1.437087548379	2.669205766778	-2.316099482016
C	2.536593598211	2.106289610854	1.713107691756
C	3.505407588054	1.336863444534	2.366777962827
H	4.249216350160	0.786392733000	1.789156734645
C	3.529054187818	1.245890479163	3.760409780991
H	4.296668971909	0.636456086697	4.244255934972
C	2.582735343575	1.920140612496	4.527588924377
H	2.601493221261	1.848592187552	5.618005224970
C	1.600582405592	2.678612872239	3.886785789189
H	0.840724070156	3.203512930482	4.471642028707
C	1.574225527708	2.759811439449	2.497948096696
H	0.774757954200	3.327866236075	2.015046932923
C	-2.987158266592	1.028237733115	-0.268747755290
C	-4.054283746385	-0.036848127946	0.057563274724
C	-3.959182412143	-0.746340188157	1.266772928798
H	-3.116365554820	-0.567430409850	1.938877929955
C	-4.930147655797	-1.671333037459	1.636862068104
H	-4.822340059501	-2.217449193670	2.577501712037
C	-6.037598304939	-1.893237472676	0.815423398846
H	-6.802797989622	-2.618375495844	1.103764977033
C	-6.163899680494	-1.167763279352	-0.366003776940
H	-7.034544309068	-1.313974052359	-1.010557887071
C	-5.182420674059	-0.245824105545	-0.739896631700
H	-5.310537109556	0.315021087106	-1.666591522283
C	-3.053396606057	1.537703265373	-1.721137205242
C	-3.058337248548	0.607071998913	-2.771424482026
H	-3.099488015377	-0.462485032729	-2.548677343753
C	-2.985097767022	1.018086295665	-4.098918430816
H	-2.996467334938	0.270278398957	-4.896123616009
C	-2.881969244164	2.375691161013	-4.409456378941
H	-2.815900996538	2.700736736984	-5.450841255866
C	-2.850064115383	3.308608844612	-3.376232932786
H	-2.753327597041	4.374306526494	-3.599425789660
C	-2.934765428442	2.893535721957	-2.045413313641
H	-2.888430348162	3.643995471321	-1.255373421020
C	-3.271236550212	2.119990401752	0.779777639389
C	-2.521357501719	2.234126089673	1.956419257874
H	-1.668268909641	1.575106126426	2.132048782400
C	-2.858119077098	3.171649680874	2.934734738528
H	-2.251861949632	3.240971356735	3.841700647052
C	-3.960795245859	4.004228819421	2.760348472768
H	-4.223473270325	4.741115003522	3.523523943132
C	-4.737630379530	3.875407767170	1.607280264459
H	-5.617530159755	4.507974455253	1.464312912953
C	-4.401455425651	2.937377381778	0.634804051167
H	-5.033648719471	2.835272569776	-0.250335587887

108

3′a (^{1}A, C_1): E_{tot}(RPBE0-D3(BJ)(Acetonitrile)/def2SVP) = -3332.64518060 $\langle S^2 \rangle$ = 0

P	-0.526000221115	0.214781976788	-2.021718842903
P	0.887938159410	1.343077108260	0.661719935492
P	0.978817038259	-1.050411130442	-1.068477105284
C	1.864306841388	0.178644376953	-0.035288453606
C	-0.710083158986	0.784556821682	-0.206275981510
C	-0.778585340913	-0.723471727252	-0.450536017800
C	3.385308141756	0.143523799987	-0.068903501681
C	3.966264412755	-1.214577183905	0.346523238800
C	3.198665080935	-2.181559773080	0.999849701096
H	2.142108939109	-1.999826061659	1.201079304852
C	3.766148863168	-3.385947912090	1.421611568716
H	3.139911003696	-4.125237423125	1.927944399222
C	5.117833141252	-3.637376853087	1.202263680996
H	5.565007744841	-4.579544138962	1.529288793659
C	5.900791112483	-2.668513880783	0.569054300671
H	6.965914273201	-2.848126979437	0.401222684069
C	5.331757849606	-1.468560841748	0.152606934307
H	5.958305712606	-0.714227319120	-0.330357447580
C	4.013477555413	1.157007729070	0.900327997304
C	5.032515474559	2.035487455289	0.520224209175
H	5.380926776688	2.055412132047	-0.513534163262
C	5.620010828012	2.894531459578	1.451500760099
H	6.412090057894	3.575254381903	1.128701621106
C	5.203458526715	2.884975418247	2.780665023797
H	5.660280046874	3.561121398761	3.507819783574
C	4.205815862772	1.992739867582	3.177229142033
H	3.880139987125	1.959671082413	4.220131709325
C	3.625626434608	1.132932048888	2.248475723938
H	2.867377721753	0.419977653541	2.580237544325
C	3.637800210099	0.470642824736	-1.555683355067
C	3.326185452888	1.754669577870	-2.031675660686
H	2.992065239172	2.522474071380	-1.329508427284
C	3.407952530108	2.057963140376	-3.385859811083
H	3.165059257094	3.067320978976	-3.728292494557
C	3.782036386785	1.073400960888	-4.304791354624
H	3.838035775464	1.306889164908	-5.371052538833
C	4.068875390532	-0.209838172072	-3.849443022972
H	4.349183727570	-0.994788181833	-4.556510593795
C	3.995524649119	-0.510199467233	-2.486220510980
H	4.213658440003	-1.526440754777	-2.154852433832
C	-1.839195225792	1.878191092010	0.023114648311
C	-2.567684818371	1.679485674170	1.358650950561
C	-1.895094346105	1.180349322526	2.481261251846
H	-0.845540290814	0.892789970569	2.413853482003
C	-2.539492042440	1.030871337938	3.707237784780
H	-1.988124237856	0.621003063625	4.557351754345
C	-3.878721830579	1.395213955675	3.844459796657
H	-4.390017336176	1.274502184927	4.802811334923
C	-4.554515703975	1.918480730799	2.743673224673
H	-5.603244516354	2.213620061220	2.832720361721
C	-3.906220539915	2.056657357130	1.516412019226
H	-4.463209696535	2.454420803696	0.667081703473
C	-1.056747101088	3.216429913560	0.064475011067
C	-0.259591188803	3.577584299737	-1.037361960032
H	-0.228572018458	2.937681402147	-1.921746973322
C	0.501494365022	4.741991337186	-1.023994053311
H	1.118969195495	4.991666324953	-1.890746319924
C	0.476024914208	5.584325034461	0.089659552716
H	1.077758959159	6.496452051046	0.106058305908
C	-0.333684789874	5.254727962187	1.173011245938
H	-0.375248408825	5.910318288587	2.046630866664
C	-1.095025213840	4.083110992380	1.159008203217
H	-1.714167021119	3.843357026438	2.024153195698
C	-2.864974269689	1.998939865485	-1.122779715970
C	-3.464682649741	0.877160025372	-1.705781717669
H	-3.207623782236	-0.117524118772	-1.359584109970
C	-4.420687699895	0.996639729356	-2.714363522304
H	-4.855314812195	0.091277966774	-3.145859456753
C	-4.821068018773	2.254355714609	-3.157829070353
H	-5.571804291494	2.353765778341	-3.945960141612
C	-4.254008750265	3.386202787485	-2.573731471358
H	-4.561734964655	4.384462080147	-2.895749162703
C	-3.289792639112	3.258883995568	-1.574877662920
H	-2.873773281496	4.166219143470	-1.136225190513
C	-1.594784167216	-2.000817219798	-0.039868621084
C	-1.270768057020	-2.385397737284	1.415346480522
C	-0.273459608259	-1.768209442579	2.167720298952

H	0.304123428486	-0.948991290741	1.742898619561
C	0.006186405497	-2.172318070460	3.476585752214
H	0.793015723416	-1.660739312089	4.037189596549
C	-0.712281360910	-3.211617914698	4.057893064767
H	-0.498494691712	-3.529735632724	5.081423210323
C	-1.714503889265	-3.843101068213	3.315980755614
H	-2.288452682841	-4.663135183473	3.755322764535
C	-1.989353487821	-3.432829106442	2.015873935047
H	-2.775633051203	-3.939948495699	1.451774147158
C	-1.054601339588	-3.100690986749	-0.985409417876
C	-0.315037372511	-4.192816121070	-0.517065958872
H	-0.151442176355	-4.326419435381	0.552087335943
C	0.256005353752	-5.111789523725	-1.399134544153
H	0.837609838215	-5.946202427805	-0.998668149594
C	0.099632154146	-4.963053680959	-2.774433654502
H	0.551313640227	-5.679870319879	-3.464840437604
C	-0.638108469877	-3.883162602505	-3.259686047426
H	-0.770608969477	-3.744701253368	-4.335743454507
C	-1.202589312445	-2.965831221643	-2.377498948579
H	-1.752670800527	-2.117539427770	-2.788053356947
C	-3.136499545058	-1.905905315713	-0.121149132417
C	-3.826057770080	-1.224278910281	0.892639778596
H	-3.266208889908	-0.770050483333	1.708203379381
C	-5.213708209359	-1.121697262684	0.887976830108
H	-5.712497436514	-0.574716461450	1.691100995079
C	-5.961592945082	-1.715818806467	-0.128232464376
H	-7.051544955388	-1.635435033681	-0.135085062181
C	-5.298209227475	-2.432527973816	-1.119756390330
H	-5.863028943954	-2.931224368773	-1.911666223739
C	-3.905507291104	-2.534159101684	-1.109512344872
H	-3.431906218415	-3.124604609650	-1.890921548471

108

3′b (^{1}A, C_1): E_{tot}(RPBE0-D3(BJ)(Acetonitrile)/def2SVP) = -3332.63575303 $\langle S^2 \rangle$ = 0

P	-1.100860590797	1.445405505180	-0.958886133378
P	-0.887786845980	-1.526711283393	-0.404617504590
P	-1.010318312050	1.166698223207	1.151313098223
C	-1.873839814145	0.002727943984	-0.063410452212
C	0.604929414044	-0.735133198818	-0.219602246108
C	0.439885717898	0.778418427121	-0.034803762202
C	-3.413275170988	-0.148199592315	0.044331795721
C	-4.073792743603	-0.219077764511	-1.341093215262
C	-3.372883182696	-0.570525134018	-2.498547642080
H	-2.302366287529	-0.780709210500	-2.458613227934
C	-4.020388494012	-0.664135956374	-3.732739091316
H	-3.444577505000	-0.938071055049	-4.620480280229
C	-5.384961289915	-0.407083136019	-3.832761598143
H	-5.891455550207	-0.475621500273	-4.798847402974
C	-6.099005728582	-0.058932905451	-2.684615192813
H	-7.171314032131	0.144635230469	-2.744859483509
C	-5.449340273517	0.034001780645	-1.456917805990
H	-6.022886174560	0.306511814832	-0.567838771784
C	-3.670110454390	-1.458370735231	0.818627717354
C	-2.976488911082	-1.705344647624	2.016562332084
H	-2.292688799290	-0.953561127118	2.416020280270
C	-3.139013611340	-2.898121714759	2.715570323125
H	-2.582425433349	-3.058960220404	3.642355238210
C	-3.999565222849	-3.884113324220	2.231101236293
H	-4.124114756945	-4.824795665968	2.773357201303
C	-4.692996029488	-3.655438246242	1.046051938772
H	-5.367395182784	-4.418013393543	0.647873371229
C	-4.528885514868	-2.456421779840	0.347929798476
H	-5.074288159801	-2.313493732006	-0.585300565087
C	-3.971441605703	1.082202711893	0.788542745801
C	-3.968404272051	2.324928374384	0.132387048739
H	-3.646659254924	2.388499858710	-0.910399766440
C	-4.380325591087	3.486047200963	0.778819507909
H	-4.352152730352	4.439129283172	0.244556885500
C	-4.831421676204	3.430350550189	2.098641169927
H	-5.157736612122	4.339650153624	2.609769846750
C	-4.873421342983	2.201396840394	2.751254352725
H	-5.241301310685	2.136643120246	3.778495570119
C	-4.447630400413	1.039331151213	2.102954765131
H	-4.495628224119	0.091581391745	2.639360488533
C	1.796815563149	-1.741824927962	-0.048628673804
C	1.799859383410	-2.312910686898	1.400238433209
C	0.819221583035	-2.040984196012	2.355790102686

H	-0.011847507030	-1.377868621129	2.118130386179
C	0.871503784450	-2.598017373881	3.637505149554
H	0.091194152426	-2.345581673762	4.360196291255
C	1.904560432051	-3.459626101383	3.988868888547
H	1.946591470670	-3.899268168408	4.988591795369
C	2.889831225682	-3.753722673441	3.043154727876
H	3.707758772372	-4.434151213789	3.293923835516
C	2.840231705227	-3.181874405146	1.776520003621
H	3.626572098075	-3.422539848747	1.058281671845
C	1.465528155428	-2.901961793701	-1.033045441473
C	1.286658938075	-4.229356146444	-0.634374472798
H	1.382765740680	-4.508143817979	0.414247358024
C	0.949111398141	-5.223601498430	-1.558613122642
H	0.803487357925	-6.248983249437	-1.208922820753
C	0.787791313802	-4.913042325318	-2.904122068978
H	0.522865384477	-5.690587510449	-3.625184652532
C	0.958254322268	-3.589908086989	-3.320114755451
H	0.827580450681	-3.320890667649	-4.371492200506
C	1.281376195915	-2.603700053832	-2.395984597237
H	1.372283214467	-1.570792786131	-2.733993670368
C	3.256482667543	-1.263275267797	-0.308729506908
C	3.994258563524	-0.690452928267	0.738147607467
H	3.519235035065	-0.520234236909	1.699250938884
C	5.343169811886	-0.374262307059	0.609585872697
H	5.870586086136	0.069854063412	1.458095033645
C	6.015804076416	-0.642750129325	-0.580678829265
H	7.078715536679	-0.410696136645	-0.686599919309
C	5.308363502713	-1.222212390590	-1.629716292079
H	5.811488268691	-1.450656011427	-2.572918093065
C	3.953528052122	-1.528588641003	-1.493938451751
H	3.456604825998	-2.004468433130	-2.336546435592
C	1.594916590902	1.836574522433	-0.092156610593
C	0.967607643093	3.252723362864	-0.311853813450
C	0.396837066981	3.935780175820	0.776438811164
H	0.442960852603	3.500900974530	1.776949569297
C	-0.236659768952	5.162887244581	0.611280542202
H	-0.684854365671	5.657309282864	1.476859576118
C	-0.293615749034	5.760647600293	-0.649214862808
H	-0.787003164661	6.726953452976	-0.781232558109
C	0.299760448214	5.117519300624	-1.730892732416
H	0.280987736185	5.576265338545	-2.722757936303
C	0.923680784477	3.878586889109	-1.563851485897
H	1.371507322800	3.398914427686	-2.433959096963
C	2.476315381984	1.539770891238	-1.327550075724
C	3.820747498660	1.917612472753	-1.408597573500
H	4.341863410664	2.292512738813	-0.530745038931
C	4.534708171084	1.817045301979	-2.601844060474
H	5.583912765859	2.122807293570	-2.620692515001
C	3.927992205461	1.325218647851	-3.755213750963
H	4.487911391397	1.251205682897	-4.690849701553
C	2.599579104247	0.912640600405	-3.687234181329
H	2.100027962353	0.506726761351	-4.570802442733
C	1.890119221929	1.018926749441	-2.492058494341
H	0.847519617195	0.701714565315	-2.489170727508
C	2.357924986442	1.956780128168	1.238541863583
C	3.288736494556	2.992868861535	1.426919437924
H	3.479601024919	3.707819182931	0.625297181454
C	3.949038116845	3.163358276054	2.640565521227
H	4.674868759823	3.973624513913	2.747689981363
C	3.667892561216	2.325370974657	3.721449251105
H	4.178963898468	2.464263439613	4.677490468743
C	2.709197938067	1.328537844480	3.568990078322
H	2.456212929453	0.669030008481	4.403257649150
C	2.064879861627	1.154874470199	2.342688936955
H	1.333317009499	0.355693667652	2.255684183483

108

3'' (^{1}A, C_1): E_{tot}(RPBE0-D3(BJ)(Acetonitrile)/def2SVP) = -3332.67247117 $\langle S^2 \rangle$ = 0

P	1.551849477878	-0.373654932350	-2.458281362642
P	0.987599941387	1.263264411967	-1.061813018767
P	0.837772638749	-1.588221816146	-0.754503944302
C	2.028545548021	-0.193468125705	-0.661306317666
C	-0.657771846612	0.657371921154	-0.372452550927
C	-0.758437752771	-0.689141111323	-0.311396419826
C	3.412057481488	-0.118196954557	0.016429308877
C	4.427935923538	-1.099297772058	-0.582067333680
C	4.053131184458	-2.342303389356	-1.104032126490

H	3.004435267693	-2.646649048002	-1.122769684049
C	5.005171715108	-3.239645043153	-1.589295827985
H	4.678039710280	-4.200672767780	-1.994481596931
C	6.359888130585	-2.916155780084	-1.554673110772
H	7.106388768503	-3.617358465086	-1.936341746302
C	6.751157909624	-1.687399533801	-1.023402511159
H	7.809899959786	-1.418629535361	-0.980922012471
C	5.795871644754	-0.792451497840	-0.545196699998
H	6.123815678612	0.162608789087	-0.129946799319
C	3.189994180120	-0.428594974861	1.506218645055
C	2.148655198612	0.208554885731	2.199362078643
H	1.498856498406	0.911602074819	1.675484312413
C	1.927408961586	-0.032480942804	3.552149900166
H	1.110164607154	0.482207731584	4.063525269676
C	2.738928648978	-0.931459105293	4.246756737478
H	2.564572540454	-1.126907820506	5.307818552833
C	3.768465333968	-1.579787247612	3.569548477317
H	4.409452075394	-2.291807408498	4.095926779674
C	3.992777811523	-1.328822719887	2.213497126454
H	4.807775313592	-1.847856663434	1.707141371014
C	3.920849740212	1.319568938961	-0.196410850073
C	4.176328514316	1.747529001972	-1.510072547876
H	4.065710857390	1.043287260696	-2.338900137281
C	4.571302419753	3.054425430391	-1.776155334966
H	4.754140106217	3.364002671355	-2.808405233128
C	4.735046054628	3.965200274944	-0.729842792254
H	5.042742001334	4.993530716982	-0.935724578949
C	4.513395081560	3.544795703100	0.578318708431
H	4.652158089159	4.240659207763	1.409913351633
C	4.110499371971	2.232493232001	0.842820625890
H	3.944057670651	1.929370026876	1.876999479836
C	-1.658055946013	1.799971663003	-0.057853723884
C	-0.924641381156	3.170150522776	-0.201917614272
C	0.048818036372	3.513742658317	0.753252707631
H	0.248510139036	2.838423301496	1.588869705121
C	0.780636749684	4.690510940611	0.647370480269
H	1.543588712134	4.919976211540	1.395328825323
C	0.544594629945	5.570601545550	-0.411771248855
H	1.119281793694	6.495987352544	-0.500173489856
C	-0.439488862438	5.259645223153	-1.344438507560
H	-0.649324062064	5.944085146715	-2.170548790458
C	-1.170425459712	4.071430000387	-1.239035057469
H	-1.931305373592	3.854427120197	-1.988863689754
C	-2.791632214477	1.726743794469	-1.096466032948
C	-4.138235005145	1.967033959167	-0.817404457688
H	-4.472008499362	2.122031568961	0.206533972020
C	-5.093039072509	1.983217467688	-1.835691171699
H	-6.139836176730	2.166813807615	-1.580722191103
C	-4.726550072574	1.747872641420	-3.157535695539
H	-5.477102166665	1.757227466577	-3.951999685141
C	-3.387236343093	1.490386407188	-3.452159155537
H	-3.074713680396	1.296726193729	-4.481567861739
C	-2.437605198720	1.485034943127	-2.434614339113
H	-1.391514575186	1.302389645821	-2.693317934527
C	-2.101294531810	1.809128550627	1.415702854105
C	-2.961668406312	2.820904645684	1.873067258454
H	-3.339545135894	3.571994550460	1.177481213223
C	-3.318794137698	2.912004559586	3.215564456510
H	-4.004643442700	3.700213174166	3.536855718084
C	-2.786139344055	2.021297655632	4.150009062743
H	-3.060515941671	2.096611781742	5.205252172573
C	-1.879115673297	1.056664217078	3.721302655170
H	-1.428444331006	0.363189832932	4.436228193628
C	-1.541727998343	0.958673166920	2.370068952816
H	-0.830960289021	0.197808731551	2.058386453557
C	-1.866038113341	-1.754226106170	-0.086908319510
C	-1.571899796634	-2.573818911000	1.185451103518
C	-0.455926143188	-2.358674041661	1.996961445591
H	0.260363466431	-1.568800744615	1.764948300157
C	-0.233747286635	-3.124049165766	3.145253477283
H	0.648401537182	-2.920871296937	3.757208101709
C	-1.131309069290	-4.123227318231	3.506319719105
H	-0.961041618654	-4.722383356795	4.404358320147
C	-2.259574763299	-4.344219241879	2.712425422473
H	-2.980390351272	-5.119242340936	2.985297474936
C	-2.477318941639	-3.575556857801	1.573925017506
H	-3.374578775291	-3.751654964700	0.976292066567

C	-1.715199242045	-2.647467836598	-1.350990994123
C	-1.539704641815	-4.034740858920	-1.316070044041
H	-1.510262915102	-4.563846935309	-0.364008472659
C	-1.371837582190	-4.765999578386	-2.493706421232
H	-1.229491434639	-5.848109218808	-2.434367471964
C	-1.372477339818	-4.127065196874	-3.731215295743
H	-1.236521407310	-4.702156094654	-4.650653073493
C	-1.539277336619	-2.742618632019	-3.782154625132
H	-1.535099065366	-2.219769610815	-4.741935040229
C	-1.702503713604	-2.016771669059	-2.606785674995
H	-1.814992853705	-0.932485387370	-2.659231237468
C	-3.333621214393	-1.274593609678	0.027904954150
C	-3.826048718448	-0.851156850622	1.271185086205
H	-3.156219141377	-0.805347388823	2.126634938195
C	-5.167470768643	-0.530041606942	1.456627342205
H	-5.508754827104	-0.202714801616	2.442337420918
C	-6.070131506618	-0.649615949861	0.400387145963
H	-7.127664029119	-0.413063387934	0.543517849365
C	-5.604152540069	-1.086584039220	-0.836141663046
H	-6.293898562341	-1.196203582145	-1.676827887072
C	-4.255258254240	-1.393685747757	-1.019262815498
H	-3.938481316990	-1.749277700305	-1.998377153768

108

TS(3-3′) (^{1}A, C_1): E_{tot}(RPBE0-D3(BJ)(Acetonitrile)/def2SVP) = -3332.59091286 $\langle S^2 \rangle$ = 0

P	0.589268863181	0.187679364503	-1.164757102732
P	-0.908501726008	-1.494378776892	0.473348751743
P	-1.179014238294	1.622518927612	0.575362804850
C	-1.775040847925	0.018288396083	0.142777305487
C	0.656075245627	-1.308069373901	-0.169879321027
C	0.392382079418	1.581981285837	-0.050463125771
C	-3.317140839262	-0.165240781831	0.149136227547
C	-4.034179668733	1.129451364730	-0.278883415650
C	-3.785744955500	-1.176372026385	-0.916548044814
C	-3.668245908612	-0.616297941306	1.574686014736
C	-5.213709299046	1.575549198049	0.325460552768
C	-2.978093161792	-1.555692508937	-1.994018911066
C	-3.644125993105	0.313172463536	2.628232137041
C	-3.559596067764	1.833335554450	-1.398403365104
C	-5.103603250778	-1.657391250668	-0.880299309836
C	-3.853654144278	-1.966352485701	1.901398827486
C	-5.875006567556	2.713174189334	-0.143678347023
C	-3.457079643095	-2.416243618573	-2.984696790479
C	-3.818829572440	-0.087117323124	3.949190456230
C	-4.216180300709	2.967904199552	-1.867391209806
C	-5.582822321664	-2.519324157290	-1.862927881680
C	-4.020900226250	-2.372288854724	3.227772478737
C	-5.375171472126	3.421160815147	-1.234125724630
C	-4.757558673451	-2.910104792572	-2.919927813297
C	-4.009835999622	-1.436486614265	4.258092117005
H	-5.630865643191	1.033091106757	1.175419472501
H	-1.962971940947	-1.162016885576	-2.070195581012
H	-3.470611349161	1.370286832713	2.413014847492
H	-2.666758613747	1.476190727339	-1.916876289776
H	-5.767808393244	-1.351016107699	-0.068483317954
H	-3.860546369191	-2.721328369901	1.113888568843
H	-6.791001368125	3.044203178984	0.352864784361
H	-2.802040056031	-2.696960889806	-3.813853283113
H	-3.800758173422	0.661504500015	4.745784433997
H	-3.821461069524	3.498019941428	-2.738233551123
H	-6.610781667999	-2.886755162358	-1.804011894126
H	-4.157910469327	-3.433953453415	3.449980234733
H	-5.890349135298	4.314208333073	-1.597070012213
H	-5.131952380856	-3.587934091198	-3.691404503483
H	-4.144398764277	-1.752552192165	5.295753828448
C	1.766483344997	-2.346847114824	0.007643112352
C	2.914330528603	-1.664025513001	0.778805680895
C	1.212471586130	-3.556319795101	0.801111202117
C	2.233226845454	-2.938557494568	-1.335316334075
C	4.247867600238	-1.664601384096	0.355221991734
C	0.239366149295	-4.361571200035	0.184034396679
C	1.703130860707	-2.557101243235	-2.569460275228
C	2.605784480576	-0.991066756236	1.972589959862
C	1.644231058129	-3.913984459004	2.080166100651
C	3.176320070427	-3.979919076732	-1.322209450893
C	5.243139186965	-1.037333176937	1.108512715418
C	-0.305940662766	-5.461577111992	0.836778864742

C	2.130447960123	-3.157094794940	-3.757491716961
C	3.599301677703	-0.390998516874	2.739020243623
C	1.102047022429	-5.023433058097	2.736695805092
C	3.608293855271	-4.578279306379	-2.501460255073
C	4.927361024588	-0.409726883276	2.309709499989
C	0.121048220067	-5.796815373134	2.124120976951
C	3.091178759612	-4.163070987812	-3.731141505792
H	4.527783626713	-2.145116799330	-0.581986467970
H	-0.094423497870	-4.120831308454	-0.828056045242
H	0.930454482236	-1.788030060183	-2.625310856873
H	1.566494490511	-0.943262023437	2.305536633310
H	2.417838842293	-3.333407786694	2.583051025336
H	3.570638310758	-4.336301017724	-0.367451545441
H	6.274042855668	-1.045244494018	0.745309111941
H	-1.067351375984	-6.064320612244	0.334922146094
H	1.697179492663	-2.832741172560	-4.707135608644
H	3.331213566830	0.109932176770	3.671263410735
H	1.459792145399	-5.280341412372	3.737225657830
H	4.347775745733	-5.382241290631	-2.459362769593
H	5.706120678272	0.074510679740	2.904002456472
H	-0.303742264774	-6.662162547254	2.639321909356
H	3.426179877391	-4.633970551479	-4.658830553003
C	1.453115493548	2.672794548201	-0.077421026092
C	0.799314023853	4.048937920147	-0.313596386272
C	2.322252890027	2.724487561062	1.185355810671
C	2.291668039019	2.252763918385	-1.292050241085
C	1.170350933313	5.188874264614	0.404185796521
C	1.727165051796	2.648653550660	2.448405432146
C	2.551312052895	3.043273829642	-2.400963264628
C	-0.182857842080	4.182501257217	-1.308019248465
C	3.696259384241	2.968868246739	1.104424318631
C	2.681369431989	0.878333271568	-1.322741170263
C	0.565973905818	6.423813088880	0.150749025324
C	2.481792657483	2.837991922093	3.603638489552
C	3.181698571839	2.492682713088	-3.523111621994
C	-0.781024977585	5.411451554316	-1.566983806342
C	4.457157278417	3.150561349768	2.260537470413
C	3.353938701157	0.352115124545	-2.453324753825
C	-0.412812147380	6.540851798660	-0.831635586256
C	3.851610478282	3.092983083127	3.514412456324
C	3.574724454311	1.150234041500	-3.561365698772
H	1.936262967119	5.123797959301	1.178292503037
H	0.654336759056	2.452902504443	2.526907503488
H	2.253998670771	4.092538222465	-2.411832725555
H	-0.481954011736	3.306352120519	-1.887709724112
H	4.182486696227	3.034784517936	0.127633207690
H	2.833519716135	0.361499609108	-0.363566874799
H	0.867174617927	7.298136014605	0.733645586630
H	1.996545076982	2.782221776286	4.581577725237
H	3.370552891524	3.132403770266	-4.389229178408
H	-1.543884659084	5.487537283400	-2.346255097774
H	5.530365019250	3.340612998075	2.176629945333
H	3.696105710479	-0.683803312523	-2.431932539765
H	-0.888317092232	7.505508974768	-1.026676008676
H	4.445341005085	3.237876107341	4.420552703997
H	4.071173402830	0.747566265080	-4.446409263313

108

TS(3′-3′a) (^{1}A, C_1): E_{tot}(RPBE0-D3(BJ)(Acetonitrile)/def2SVP) = -3332.58379417 $\langle S^2 \rangle$ = 0

P	0.803394849377	0.145105491172	1.482507882608
P	-1.134460156995	1.502082897177	-0.543749563795
P	-0.853695973103	-1.315605364112	0.547690034110
C	-1.847494241131	0.016522765943	0.004832710498
C	0.414785141350	1.407771817527	0.194385533096
C	0.910888124040	-1.083053492290	0.242009103120
C	-3.352371308677	-0.274639151572	-0.108545683393
C	-3.999902523176	-0.684025378530	1.229451223062
C	-3.363058469224	-0.548205656973	2.465367216447
H	-2.335933263311	-0.183598040280	2.516986726217
C	-4.029680231574	-0.861551778414	3.653350036313
H	-3.507038198717	-0.746201136716	4.606459286898
C	-5.346355658510	-1.310861006541	3.625559201716
H	-5.866491454987	-1.558856030323	4.554315963673
C	-6.000907252601	-1.429561512312	2.396905458513
H	-7.039254637564	-1.768900175423	2.357642317318
C	-5.337638424546	-1.110223277185	1.216735985446
H	-5.869951287205	-1.189247675706	0.265558149652

C	-3.429131244455	-1.389479412602	-1.166007588948
C	-3.058729676299	-1.096463217971	-2.489587102897
H	-2.774982999555	-0.075297095728	-2.756737830849
C	-3.036531505237	-2.086852884667	-3.465389762688
H	-2.758046953607	-1.828872155314	-4.490416010653
C	-3.354241689760	-3.406910098926	-3.134481924010
H	-3.330611790769	-4.188111883300	-3.898377397319
C	-3.678688071986	-3.719319836344	-1.818066039194
H	-3.906317402295	-4.750954536255	-1.537697716022
C	-3.717223394260	-2.719555933954	-0.842401786374
H	-3.972114864781	-2.990364829879	0.182944350567
C	-4.125290355631	1.001674890447	-0.501419627241
C	-3.990486498739	2.140212721665	0.310933572953
H	-3.338725446894	2.106519554367	1.187579177945
C	-4.689409212079	3.310582531397	0.031638554112
H	-4.554406509849	4.185077582318	0.673445667627
C	-5.562515018833	3.362862245403	-1.056861309345
H	-6.113887291695	4.280006147619	-1.279175771901
C	-5.735129924454	2.228642665704	-1.845568203269
H	-6.430408439391	2.246315936108	-2.688914632847
C	-5.024449247104	1.057304935163	-1.569152409919
H	-5.185506776218	0.180913975620	-2.198027798245
C	1.430398024573	2.559603062274	0.004440965726
C	2.146957325214	2.521257135962	-1.361145183472
C	1.585893357635	1.928326852737	-2.494423994930
H	0.634604855061	1.398763236379	-2.421206331386
C	2.225232184219	1.991718324750	-3.733967014151
H	1.762403646071	1.512926855587	-4.600721708876
C	3.444844139703	2.651802762299	-3.864615254662
H	3.948499560990	2.698537130308	-4.833439599539
C	4.018585159188	3.247659807418	-2.740680440363
H	4.975142060538	3.770203174242	-2.823033267704
C	3.376426643153	3.181387648670	-1.506452982099
H	3.838207734004	3.662444243320	-0.641461799024
C	0.631087162114	3.879439256067	0.124825643475
C	-0.248623848720	4.054012784906	1.208512654236
H	-0.354627588578	3.259037252256	1.950525453894
C	-1.001196649275	5.214699078625	1.346572090465
H	-1.677210920797	5.321236188123	2.198986156232
C	-0.899629228181	6.234793520466	0.396523364618
H	-1.497387076255	7.144298418577	0.496705871916
C	-0.032314121870	6.076348158024	-0.679170889888
H	0.056657843418	6.861697175748	-1.434310339407
C	0.728092446208	4.910074520721	-0.812672540577
H	1.392230406984	4.810412076593	-1.671695232657
C	2.496956047814	2.462501686931	1.119215083036
C	3.423381351003	1.406840450533	1.059242499525
H	3.412540324141	0.729665948717	0.204561705946
C	4.386907985808	1.230290260485	2.050377314451
H	5.096225855714	0.402923524945	1.968135739301
C	4.447107040547	2.109026785447	3.131355220086
H	5.199151251491	1.974952565882	3.912876757344
C	3.547223607049	3.170634434202	3.193585275044
H	3.591862503400	3.879187675692	4.024702907533
C	2.585351947933	3.348793097531	2.195933918214
H	1.906406052274	4.198151227662	2.268990302864
C	1.845673619359	-2.283084361926	0.161838058738
C	1.361477476949	-3.333419523015	-0.847387566695
C	0.518730040471	-2.993011294317	-1.910833877706
H	0.129499851177	-1.975613191113	-1.995896980041
C	0.184530742002	-3.935357512538	-2.884925242898
H	-0.471907960499	-3.645113255329	-3.708030032231
C	0.683884912792	-5.233671556978	-2.810313441323
H	0.417659008093	-5.971925678972	-3.571022529680
C	1.535494153434	-5.579583270175	-1.759646039892
H	1.943746039098	-6.591448828206	-1.693361325850
C	1.876035032251	-4.636116774261	-0.793665172132
H	2.558983853247	-4.915467728196	0.012037263904
C	1.901561642121	-2.795596635362	1.606228894689
C	1.023727848525	-3.793989132081	2.052337464485
H	0.367574219634	-4.299342483882	1.341449697267
C	0.963801790189	-4.143335593523	3.399304356016
H	0.275001216130	-4.929020491548	3.720329366280
C	1.771682960665	-3.494378841895	4.334651614850
H	1.722847304206	-3.769572143274	5.391235893504
C	2.634953042541	-2.487291834594	3.907803302441
H	3.267125447000	-1.960501062627	4.627268051977

C	2.695334655151	-2.137533734775	2.559087163421
H	3.372687628309	-1.341320707356	2.247365587753
C	3.202875318941	-1.836985136370	-0.405692404329
C	3.220511104640	-0.958843351294	-1.495445990370
H	2.278217276846	-0.559394079715	-1.875138992377
C	4.420304859796	-0.582644509230	-2.093585048489
H	4.406482401410	0.117936919651	-2.931937573685
C	5.628399461573	-1.093199009279	-1.617492828647
H	6.573264682715	-0.796752243717	-2.079896698123
C	5.618286857218	-1.994407419810	-0.553579166623
H	6.556131161603	-2.415248207528	-0.181806458033
C	4.414425717106	-2.368464097030	0.045938611599
H	4.428211128536	-3.082836360277	0.870884893775

108

TS(3′a-3′b) (^{1}A, C_1): E_{tot}(RPBE0-D3(BJ)(Acetonitrile)/def2SVP) = -3332.57462682 $\langle S^2 \rangle$ = 0

P	-0.275276939316	0.953468014593	-2.130651954338
P	-0.970206828402	-1.427165561781	0.407280681063
P	-1.080314227459	1.530826322314	-0.185112491462
C	-1.926979950618	-0.006093000279	0.050287971009
C	0.590407713868	-0.687971623862	-0.006003432207
C	0.595211444158	0.748782093903	-0.333469385048
C	-3.445415692749	-0.072885311772	0.165403730318
C	-4.056329956008	-1.236038887310	-0.626589981268
C	-3.389673120958	-1.818973592490	-1.708821361040
H	-2.385450378584	-1.482738894625	-1.982073456667
C	-4.004642796227	-2.814588985000	-2.470298957062
H	-3.464576151688	-3.256548703506	-3.311623311439
C	-5.296504042246	-3.236354492471	-2.164870271485
H	-5.776637883575	-4.018525886548	-2.758335606869
C	-5.979016864838	-2.640853281691	-1.101874199237
H	-6.998941590666	-2.951401228821	-0.860990699858
C	-5.367400278885	-1.643977349330	-0.346523417486
H	-5.918789871514	-1.173196865498	0.471154482692
C	-3.534419580542	-0.240944386898	1.696803328620
C	-3.209099498971	0.845055995193	2.525922418696
H	-2.997303425230	1.822979525435	2.086823434117
C	-3.126092134719	0.690885688034	3.906456865233
H	-2.870669818269	1.551071054574	4.530235001419
C	-3.354135355266	-0.557608374952	4.487399583723
H	-3.282868914200	-0.682688346084	5.570728829821
C	-3.665450157366	-1.645466978268	3.673112554470
H	-3.839688392613	-2.629893754691	4.114623336334
C	-3.749357226091	-1.491574424530	2.289722045055
H	-3.972401076393	-2.361034687332	1.670143336261
C	-4.158718441316	1.181936141478	-0.361202871167
C	-3.884042427262	1.627286658223	-1.662543814915
H	-3.149805812136	1.097200678507	-2.273577734389
C	-4.552316637968	2.723739595404	-2.200721540172
H	-4.311901801898	3.056999856428	-3.213434148984
C	-5.529356529771	3.384646287245	-1.455253682009
H	-6.055588243294	4.245480525469	-1.875394141477
C	-5.838346815660	2.926301851512	-0.176302200719
H	-6.615132628532	3.421426450939	0.412071578024
C	-5.161068363165	1.832607623920	0.365313452718
H	-5.425207517837	1.487031837365	1.365896265087
C	1.671143608278	-1.796511545191	-0.208077619422
C	1.420906303435	-3.021027252577	0.715660223365
C	1.063323564076	-2.859080365086	2.062506885628
H	0.861961817746	-1.863727609716	2.462674518533
C	0.960273666011	-3.954644924427	2.916179526772
H	0.666176798559	-3.796727079120	3.956888753756
C	1.235331392007	-5.239837600330	2.449120531195
H	1.153836123722	-6.100687317287	3.117474274224
C	1.631081704212	-5.410019990603	1.123721287263
H	1.870418277566	-6.406711068108	0.744272077573
C	1.729666995359	-4.311359112408	0.270296935061
H	2.061027182485	-4.468313255411	-0.757677552408
C	1.388981245338	-2.152755182619	-1.692169159843
C	0.315575264689	-2.990038177385	-2.031493580135
H	-0.272897879866	-3.473176803524	-1.249614998604
C	-0.039083431337	-3.204848903543	-3.360980940937
H	-0.883036361721	-3.860127361941	-3.590684488256
C	0.671130882926	-2.584603855936	-4.389198508109
H	0.387563365878	-2.745043196258	-5.432405309717
C	1.747901950780	-1.761843220757	-4.067013068637
H	2.323070863704	-1.269174513341	-4.855233669886

C	2.097292261828	-1.551200812358	-2.733662285798
H	2.930093508320	-0.892172182851	-2.508907721218
C	3.149613886134	-1.528571698483	0.131179847493
C	3.478884287296	-1.000424791508	1.386144991859
H	2.692552125274	-0.621238923840	2.034165331681
C	4.791675506213	-0.971093420429	1.843773540936
H	5.005725283889	-0.544980343235	2.827369688990
C	5.822199748612	-1.482878961527	1.055949358384
H	6.855679157067	-1.463952689359	1.410854134701
C	5.510827411467	-2.032825587414	-0.185008489433
H	6.298961007040	-2.457299417314	-0.812280505081
C	4.190268300012	-2.067138362674	-0.636291231932
H	3.981766379039	-2.554840471099	-1.587994366908
C	1.732655765975	1.800124518474	0.012093596904
C	1.239296839147	3.254547785835	-0.336552986474
C	0.431521266978	3.947310093870	0.585150698851
H	0.162988123391	3.479825111667	1.533073795794
C	-0.036922657188	5.234561274263	0.331200086937
H	-0.666994183042	5.730329420136	1.073988045062
C	0.303404132322	5.885149674351	-0.853095185930
H	-0.055657986809	6.897824628650	-1.054056621016
C	1.123141701836	5.229539693435	-1.767561862675
H	1.417277719374	5.722966351853	-2.697471017633
C	1.582205582727	3.936403488903	-1.510584298170
H	2.233624149626	3.472855170029	-2.248212948168
C	3.038995181487	1.543912509667	-0.768937412624
C	4.312075308289	1.550611201394	-0.184470000575
H	4.421692713121	1.631638528651	0.893536477223
C	5.469735623505	1.430487207035	-0.951483383055
H	6.440813725131	1.430320966489	-0.450167659434
C	5.395116680219	1.304051748123	-2.337002600019
H	6.303462086046	1.216022865838	-2.938336860095
C	4.140084719178	1.283636433851	-2.939338359438
H	4.045981197833	1.173026177668	-4.022741530074
C	2.989120554053	1.394404247083	-2.161013117330
H	2.017492224208	1.378329692724	-2.664454360371
C	1.924470130974	1.838490425571	1.543629849513
C	2.822327076985	2.765982555943	2.099483503897
H	3.390819935230	3.428974355244	1.445211092836
C	2.980812328310	2.890225576044	3.476340095149
H	3.698483098406	3.614143275178	3.870859341611
C	2.215099901046	2.113851643010	4.348362494315
H	2.333867777157	2.214752551222	5.430073852288
C	1.280165462835	1.231024808578	3.817716059118
H	0.645085707563	0.632489577531	4.476013852238
C	1.136661835353	1.102998983544	2.434122996697
H	0.384807885559	0.404452272340	2.066922150387

108

TS(3′b-3″) (^{1}A, C_1): E_{tot}(RPBE0-D3(BJ)(Acetonitrile)/def2SVP) = -3332.59475478 $\langle S^2 \rangle$ = 0

P	-1.475239908816	1.389695033650	-1.651345099892
P	-0.956745308316	-1.318377268344	0.073468347426
P	-0.956336256803	1.627832558812	0.417319944979
C	-1.918560208666	0.166829155925	-0.225669052306
C	0.646634796015	-0.602703966673	0.062648897752
C	0.659664181740	0.802973203021	0.079653887676
C	-3.428411152705	-0.010693560803	0.137400857247
C	-4.189266312372	-0.600373145837	-1.058922883005
C	-3.545602858103	-1.268228707360	-2.106294943707
H	-2.457748852899	-1.342867633499	-2.135924044422
C	-4.274890749822	-1.846849427087	-3.148214170104
H	-3.741851860940	-2.360720865107	-3.952432555032
C	-5.664017359314	-1.764322659075	-3.165114605248
H	-6.235291799819	-2.212209096651	-3.982174032395
C	-6.320220560548	-1.099285868717	-2.126806904649
H	-7.410891641519	-1.026286319377	-2.124167102893
C	-5.590444559618	-0.525636459361	-1.089688787852
H	-6.119432970458	-0.012310884148	-0.283325883094
C	-3.482968502199	-0.994104470195	1.332077483373
C	-2.642314215574	-0.779069230894	2.442881152440
H	-2.005708795390	0.108428516304	2.483341238921
C	-2.605235774658	-1.675828002196	3.507984115346
H	-1.943283225516	-1.477243465856	4.354554602511
C	-3.405123939705	-2.818650432414	3.490511048798
H	-3.371199963926	-3.530288486761	4.319108056027
C	-4.245888940506	-3.040450695045	2.403286500147
H	-4.876090211320	-3.932934583932	2.370553245768

C	-4.286614826500	-2.136875862087	1.337937186713
H	-4.941939428118	-2.349761142992	0.493076282064
C	-4.050026096440	1.352712893221	0.515351304230
C	-4.347968057150	2.278190842389	-0.498917194215
H	-4.193006395566	2.002148941792	-1.541969306958
C	-4.851177192308	3.542189138925	-0.199315305661
H	-5.064633173011	4.241879627788	-1.011639663972
C	-5.091372020348	3.909193634232	1.125252909338
H	-5.487383549654	4.900252967481	1.361690933365
C	-4.839806200699	2.989137831722	2.139086162899
H	-5.042780601409	3.247445621044	3.181601043923
C	-4.326723595598	1.725564775106	1.835148349554
H	-4.152490671891	1.027880083832	2.653790460967
C	1.680086404757	-1.767000269235	-0.083772889036
C	1.757407819825	-2.568450317310	1.245629415830
C	0.979243242939	-2.304629715089	2.376061182239
H	0.258081505227	-1.487559414588	2.378832166942
C	1.107055763614	-3.064454448366	3.542964127594
H	0.483658591627	-2.821495355914	4.407238822827
C	2.018081795578	-4.112669764292	3.605281085458
H	2.118554484548	-4.708087293028	4.516206376204
C	2.810118769509	-4.387698643929	2.488096385105
H	3.537195269329	-5.203387895717	2.516207626150
C	2.686291547091	-3.621194848038	1.334546673648
H	3.332586651348	-3.841909219303	0.482485831984
C	1.055870491011	-2.658846384448	-1.199789085660
C	0.871771470155	-4.041835463920	-1.095164321352
H	1.186905958467	-4.576289437199	-0.199960395200
C	0.240552719961	-4.759886969511	-2.112843302367
H	0.092896734907	-5.836110593165	-1.991658412343
C	-0.213263657348	-4.118647957755	-3.263347800026
H	-0.713828454610	-4.685602525648	-4.052123439368
C	-0.025320895537	-2.742337351347	-3.392013292134
H	-0.371621603891	-2.215688647544	-4.284821909198
C	0.596461276913	-2.025703842833	-2.373181789970
H	0.719528034865	-0.947811077240	-2.483487674432
C	3.145995707849	-1.436625690191	-0.441311887878
C	4.028363271180	-1.029071456638	0.569861731730
H	3.650395146204	-0.853910795553	1.575145302393
C	5.392131039558	-0.890673957640	0.333614710680
H	6.048433322103	-0.574104113833	1.148624487240
C	5.918066531230	-1.174853825697	-0.926351232176
H	6.990885893557	-1.082543536878	-1.114249305734
C	5.056367216498	-1.580733559043	-1.941446990627
H	5.447176380968	-1.808762146670	-2.936409171222
C	3.687248640424	-1.710086271566	-1.701213022278
H	3.046486205888	-2.050447823637	-2.514715697103
C	1.843475456467	1.794755808160	-0.028536869534
C	1.266745486426	3.245752937781	-0.152812480048
C	0.840446829632	3.920546587977	1.004944194193
H	0.957280209636	3.449171496383	1.982409782502
C	0.256014415803	5.183447629664	0.936231097299
H	-0.071690288353	5.673683497019	1.856380069389
C	0.090029231524	5.814272083157	-0.295493589064
H	-0.367250698339	6.805215696805	-0.353554877311
C	0.525762529329	5.168521600542	-1.450125979375
H	0.416509244728	5.651181871579	-2.424711379377
C	1.109237081872	3.902783386252	-1.378573309504
H	1.442182668364	3.430988355214	-2.302140623303
C	2.695139128369	1.520968024437	-1.290625240396
C	4.078756025171	1.716494887888	-1.347081344379
H	4.648160675819	1.926752796087	-0.444713213916
C	4.770805117966	1.632340437440	-2.555360849449
H	5.852432198971	1.789713412741	-2.559332649098
C	4.102149606271	1.339894520028	-3.741816152275
H	4.648784824847	1.276315394811	-4.686202371882
C	2.728260664743	1.112538698448	-3.698199261322
H	2.177211171817	0.867959059487	-4.610408799777
C	2.042756253197	1.203219008586	-2.489177800879
H	0.958827002726	1.056882898519	-2.474929074078
C	2.634012716878	1.789095228785	1.295456295989
C	3.664014840405	2.722635110162	1.496021250286
H	3.906814662844	3.444043822316	0.714544081057
C	4.363961842416	2.777259378857	2.698028761644
H	5.170528373682	3.505353182761	2.816815827269
C	4.024935542132	1.926969222837	3.752394733270
H	4.571031445387	1.973442649203	4.697927460676

C	2.964079655770	1.041065778522	3.590657318266
H	2.660795808781	0.383694931060	4.409676673388
C	2.277044066883	0.980652062519	2.376892955892
H	1.450674820695	0.279458818309	2.280876693887

108

^{BS}**TS(3-3′)** (^{1}A, C_1): E_{tot}(UPBE0-D3(BJ)(Acetonitrile)/def2SVP) = -3332.58915134 $\langle S^2 \rangle$ = 0.9538

P	-0.485929042324	0.200452645928	-1.070710768524
P	1.407450216646	1.124422840857	0.982820422311
P	0.386693580770	-1.860935091817	0.805056688527
C	1.601092683868	-0.582097397647	0.339674239244
C	0.058605021295	1.544140091519	0.061176566479
C	-0.952173197263	-1.207761063717	-0.004564622681
C	3.039375143417	-1.056899654254	0.078182715462
C	3.102905471929	-2.267328432237	-0.861805927292
C	3.887232579189	0.013130855520	-0.621910275448
C	3.498608252209	-1.369268361675	1.511979231206
C	4.189404383548	-3.150839588541	-0.818591539875
C	3.302302543718	0.836629170462	-1.591236365798
C	3.292634296090	-2.639592089425	2.073715098414
C	2.142692072570	-2.441438456005	-1.864071393179
C	5.266360057549	0.115339081309	-0.405421033671
C	3.995354805677	-0.351859700366	2.343311714332
C	4.289907542612	-4.201358689835	-1.728231740334
C	4.061455431343	1.771469638779	-2.293124221082
C	3.594239081666	-2.888961073874	3.410997212680
C	2.241881691307	-3.491997396078	-2.776859813085
C	6.029321862629	1.046804521071	-1.108909269465
C	4.296147701721	-0.601732763339	3.680315600415
C	3.311794948467	-4.382152085386	-2.707770204906
C	5.429413814317	1.886508775616	-2.047818325796
C	4.099271191637	-1.872289021360	4.220927258824
H	4.968331229925	-3.019226616118	-0.064368987380
H	2.237814385071	0.730038266173	-1.810179486582
H	2.895470394438	-3.449650328306	1.459882650596
H	1.313550372502	-1.734246977797	-1.945730441017
H	5.754944641507	-0.536401822364	0.321728438883
H	4.154569256409	0.651057340650	1.943515441310
H	5.141700458083	-4.883982017429	-1.670837632660
H	3.577752441315	2.409680907186	-3.037173853317
H	3.431105729889	-3.888990066156	3.820608431866
H	1.475872297069	-3.609136916739	-3.547773306090
H	7.103434507041	1.115923402780	-0.918328434224
H	4.687693327610	0.206737086496	4.302820478302
H	3.389721147388	-5.208538497079	-3.418803205554
H	6.028084588778	2.620925373795	-2.592570764464
H	4.336656868389	-2.068574336877	5.269532082607
C	-0.582270658324	2.935471502643	0.059864459463
C	-2.077299991850	2.736938879332	0.362220760689
C	0.111016837963	3.799146404196	1.139103126197
C	-0.368266673131	3.678184888245	-1.272466818001
C	-3.101557871162	3.274474484912	-0.424739713801
C	1.441738347703	4.193642074186	0.912908860292
C	0.220053732182	3.104633559880	-2.400597050738
C	-2.438957867628	1.969177820805	1.480876622808
C	-0.508245059945	4.221889949290	2.317906346342
C	-0.737955203091	5.031751693388	-1.340925693601
C	-4.442679945221	3.081814833724	-0.086569851684
C	2.136206756661	4.954680340849	1.848040036380
C	0.414269661571	3.846493427974	-3.569686416641
C	-3.774749147120	1.787683663291	1.826863107936
C	0.185593262988	4.992395468580	3.255155151794
C	-0.551690372351	5.772869194961	-2.503066340107
C	-4.786161223107	2.346515308737	1.044867959112
C	1.510452684427	5.355408118649	3.030169573517
C	0.025808831617	5.180992634737	-3.629113356195
H	-2.863547905181	3.845854234189	-1.321727916612
H	1.938223784331	3.908385699164	-0.017882034480
H	0.553270879002	2.067040439596	-2.387015831849
H	-1.659021144883	1.505950804938	2.089865506436
H	-1.548371992163	3.963371505306	2.516974049112
H	-1.173457022491	5.514682925467	-0.462575748993
H	-5.221914880742	3.509212077901	-0.722798875665
H	3.171719468883	5.241771291530	1.647970156201
H	0.880826011206	3.368174141908	-4.434751587550
H	-4.026401745205	1.188225275960	2.703908205647
H	-0.325014316401	5.312828108722	4.167040378420

H	-0.852858350410	6.823334343738	-2.527486660494
H	-5.835386918086	2.194464466083	1.310130579614
H	2.051562866822	5.956678602405	3.765241654500
H	0.179105878640	5.762225129369	-4.541901811588
C	-2.303407353740	-1.943802890438	-0.077212006394
C	-2.028847188971	-3.462525119230	-0.136433062286
C	-3.168306494306	-1.646135878348	1.169273767989
C	-3.056781546068	-1.473599584249	-1.335317967907
C	-2.646010955639	-4.379819082231	0.717236751186
C	-2.605221329500	-1.473199103705	2.437365497292
C	-3.390159523445	-2.313470366840	-2.399610214042
C	-1.124771144175	-3.956239656116	-1.092483985682
C	-4.565825675149	-1.675639351509	1.069701465474
C	-3.432011278577	-0.122441033622	-1.416871823383
C	-2.361225936597	-5.746341761050	0.629339289347
C	-3.407468506767	-1.340938905435	3.570800742566
C	-4.052075835078	-1.815371778312	-3.526025561119
C	-0.842787400629	-5.314336068750	-1.185929280531
C	-5.371775414208	-1.538920511692	2.199023415912
C	-4.087562315492	0.377720197438	-2.539428728649
C	-1.459139819217	-6.219739371467	-0.318023074106
C	-4.796561808205	-1.372661740420	3.458515838026
C	-4.394732075356	-0.468221908763	-3.605858146924
H	-3.354157692870	-4.036667971201	1.472326615024
H	-1.520414427543	-1.447495656804	2.555573790022
H	-3.142279637059	-3.374468433946	-2.362488230458
H	-0.629934306091	-3.260716860414	-1.773177578636
H	-5.037802844453	-1.822163675083	0.096414068347
H	-3.242718304781	0.539478633993	-0.569859823624
H	-2.851120802250	-6.440513278694	1.317099567648
H	-2.937142301710	-1.207473511363	4.548439794306
H	-4.300618799934	-2.495319151355	-4.345007435523
H	-0.131759783097	-5.667474341976	-1.937443836567
H	-6.459093381289	-1.567833960427	2.090212909377
H	-4.363495800605	1.434279386762	-2.573864966831
H	-1.233508611088	-7.287273513408	-0.382431629452
H	-5.426603540085	-1.265174368653	4.345055054433
H	-4.908778635598	-0.079663231503	-4.488686417599

108

BSTS(3′-3′a) (^{1}A, C_1): E_{tot}(UPBE0-D3(BJ)(Acetonitrile)/def2SVP) = -3332.58748669 $\langle S^2 \rangle$ = 0.7110

P	0.969451639811	0.092084881305	1.374766436866
P	-1.060921979747	1.431798123232	-0.562843180018
P	-0.864589014375	-1.419559821549	0.638727384037
C	-1.785707695790	-0.072615626750	0.023555319203
C	0.453427960829	1.420775107234	0.219307468420
C	0.885787812053	-1.238508466759	0.223473914498
C	-3.310896341267	-0.212190886845	-0.127847595965
C	-3.891384978945	-1.428028562485	0.617575137760
C	-3.623758421763	-1.572764176499	1.988587906303
H	-2.979705443542	-0.849707157284	2.494672972253
C	-4.180709888901	-2.615094672968	2.723690569778
H	-3.947115855180	-2.709657671591	3.787248634859
C	-5.040037534059	-3.527030157936	2.108245604808
H	-5.480247699066	-4.345682010052	2.683198539778
C	-5.343417993962	-3.372084828410	0.757932173510
H	-6.030710888517	-4.065176759772	0.265899284331
C	-4.776987405497	-2.329990301403	0.020391699611
H	-5.040279397934	-2.223944812585	-1.032816023035
C	-3.439009128339	-0.313442413115	-1.659401028570
C	-3.833678314599	0.772101169670	-2.449940807959
H	-4.149502347930	1.704680468828	-1.980704824131
C	-3.816934353473	0.686717890393	-3.843456238749
H	-4.129866896538	1.549674549996	-4.436864631388
C	-3.401446335265	-0.483446236876	-4.473772229552
H	-3.390288752751	-0.550167753983	-5.564588566420
C	-2.989082232938	-1.568251134535	-3.696634534329
H	-2.650236399423	-2.490861032004	-4.174811315472
C	-2.997648495513	-1.478712303180	-2.308412951343
H	-2.642229471556	-2.326678051347	-1.718052690794
C	-4.082762860290	0.974406421343	0.471694963129
C	-3.520537567198	1.807770385806	1.443877622951
H	-2.485617021485	1.658235386839	1.758269453309
C	-4.271899740042	2.823724658640	2.038881489475
H	-3.808200368123	3.461915123556	2.795399848419
C	-5.602012257753	3.018346210466	1.675904005578
H	-6.189693722560	3.816072989707	2.137200286400

C	-6.182563288067	2.173580925051	0.727024578125
H	-7.230506481428	2.303413574092	0.444430773102
C	-5.433222333332	1.157718377392	0.140408079496
H	-5.907326476244	0.491802780638	-0.584827123389
C	1.400902604356	2.633884838929	0.068269057645
C	2.115702301137	2.662588580643	-1.303900696161
C	1.619945050301	2.035367805879	-2.448451256111
H	0.707369891479	1.439728340446	-2.397900368149
C	2.277023221795	2.147639354851	-3.676177096313
H	1.865392372165	1.638149271855	-4.551193982046
C	3.447806677084	2.893152994229	-3.784005306742
H	3.964752800486	2.978126211385	-4.743185207642
C	3.954942954567	3.527941655651	-2.648859946033
H	4.871540879734	4.120101083946	-2.712421460270
C	3.297169475054	3.411113641653	-1.427537301551
H	3.708366983211	3.919069943870	-0.552294679872
C	0.558356128299	3.914447649470	0.245320479745
C	-0.303935778425	4.015582249054	1.351210593952
H	-0.366946890505	3.187496558020	2.061311785627
C	-1.086701518696	5.146821309393	1.553796236433
H	-1.744864590131	5.199273110219	2.424853301821
C	-1.037887815957	6.206378623823	0.644144425288
H	-1.659205050547	7.092760649141	0.795353775255
C	-0.194175111043	6.116752188411	-0.458336045147
H	-0.149333955101	6.932936048556	-1.184150611193
C	0.599260683649	4.982156526437	-0.654459271373
H	1.245604399663	4.936256227894	-1.531523938704
C	2.495721572520	2.525930185853	1.153754029974
C	3.444885295100	1.494887220540	1.023628250559
H	3.437136630527	0.861694500395	0.133817419638
C	4.429910175720	1.291721538124	1.988103607161
H	5.154461110237	0.484435077160	1.854624067228
C	4.490545321264	2.120621117682	3.108424062929
H	5.259879065569	1.967071805765	3.869260766852
C	3.568626410758	3.156687410342	3.238749639494
H	3.613304442147	3.824540002568	4.102889367091
C	2.581771213381	3.359914519234	2.268855879122
H	1.880734339830	4.185025734979	2.395979403207
C	1.807436923034	-2.439685539663	0.123119664586
C	1.240058548644	-3.502234810349	-0.826418204162
C	0.394285100479	-3.133240858257	-1.879099534245
H	0.092003157579	-2.089011984419	-1.991459847435
C	-0.056302711097	-4.080157044497	-2.799197341787
H	-0.717478963234	-3.768486979355	-3.611832509484
C	0.337574999016	-5.412445275233	-2.685638380188
H	-0.017916522311	-6.156015169131	-3.403455371846
C	1.197678305515	-5.786149080828	-1.652133496051
H	1.523656569897	-6.825267823502	-1.557903324553
C	1.648002982654	-4.838736715368	-0.734540565316
H	2.327398542776	-5.146232077103	0.063479760278
C	1.959372671580	-2.914615969317	1.574675752859
C	1.058010960675	-3.833465030565	2.130236059024
H	0.305308909393	-4.308536870514	1.498148262555
C	1.099016622006	-4.142014141669	3.488314600766
H	0.388793452289	-4.865551871719	3.896833106333
C	2.034672607624	-3.529645741654	4.323257044219
H	2.065351331300	-3.771854430457	5.388573670543
C	2.924712186812	-2.600943309854	3.786123680483
H	3.657097099300	-2.104171508105	4.427738278599
C	2.884902942815	-2.293868474607	2.426613331206
H	3.584818529803	-1.558052640758	2.026420945497
C	3.133705104017	-2.014356850573	-0.524508061891
C	3.146358931675	-0.988045034955	-1.473813240374
H	2.220353245572	-0.454671656396	-1.699209595597
C	4.324427447357	-0.631467411261	-2.128784267736
H	4.309211747350	0.185688845930	-2.854591152269
C	5.510766217709	-1.308735557261	-1.850069697538
H	6.437886178918	-1.029453996145	-2.357028542319
C	5.502852601157	-2.351789298800	-0.922665998902
H	6.424234103779	-2.897586189488	-0.703262739251
C	4.323602483722	-2.704991953786	-0.267973788153
H	4.334373372890	-3.526123724490	0.452042913906

108

[BS]TS(3′a-3′b) (^{1}A, C_1): E_{tot}(UPBE0-D3(BJ)(Acetonitrile)/def2SVP) = -3332.59062442 $\langle S^2 \rangle$ = 0.9576

P	0.480464837259	-1.425754831431	-1.889524957837
P	1.000892033595	1.433299980232	-0.349350842495

P	1.120421331103	-1.548234803483	0.177520262975
C	1.973890672584	-0.000788811315	-0.168458724485
C	-0.582447492089	0.671055470306	-0.243912082750
C	-0.553662093708	-0.827248601363	-0.367790398219
C	3.479536083050	0.107303651385	0.089822734235
C	4.207873855951	1.040997371556	-0.887024048748
C	3.704259320696	1.314737363670	-2.162256333395
H	2.736765626286	0.909478363836	-2.465337976202
C	4.430369369738	2.093448286802	-3.065552280347
H	4.012567236966	2.297660501531	-4.054784984041
C	5.676549022190	2.604180419532	-2.710712318285
H	6.243690886751	3.217247910424	-3.415771646587
C	6.199333155992	2.316653025306	-1.448111335112
H	7.181608058487	2.699954390464	-1.159859814859
C	5.475087981868	1.537443663942	-0.550049817279
H	5.903236699891	1.309311630736	0.429040259112
C	3.445190424062	0.639141691321	1.537384572413
C	3.117873298804	-0.228159182428	2.591394396370
H	3.014955163751	-1.299753402960	2.405479554626
C	2.897906349242	0.257056351404	3.878712766654
H	2.640955522699	-0.440560520005	4.679920659718
C	2.998226855948	1.623705306751	4.141672024328
H	2.824004458943	2.006620601584	5.150345202716
C	3.317564265255	2.496194559931	3.101642298700
H	3.391739311383	3.570387895027	3.289288988175
C	3.533765238368	2.009930663081	1.812799413786
H	3.754978236942	2.714901099839	1.009950141984
C	4.210494922335	-1.236202787971	-0.054206483522
C	3.969050263884	-2.032509620738	-1.182419978453
H	3.239664480223	-1.709020162508	-1.928800109706
C	4.662202480909	-3.223801589320	-1.382718986814
H	4.446290900528	-3.830892091086	-2.265541168701
C	5.630937750686	-3.633413156509	-0.466126635406
H	6.176014220791	-4.568179368792	-0.619829921854
C	5.906323122870	-2.831304668478	0.640034985716
H	6.675041281873	-3.130315726403	1.357288537632
C	5.205312779525	-1.641927966718	0.842384024033
H	5.442241482387	-1.026372621858	1.711793276131
C	-1.692916015210	1.769132192196	-0.246806439683
C	-1.503252745784	2.773602638838	0.930129534482
C	-0.614961077021	2.608966303111	1.995917465776
H	0.056812705490	1.752620911282	2.038013778848
C	-0.559874031274	3.530011581959	3.047287836365
H	0.147960629399	3.361757215623	3.863104048168
C	-1.394262298017	4.641714368255	3.056263795575
H	-1.349892657355	5.363575084581	3.875702425497
C	-2.298946750433	4.816241967505	2.005663816872
H	-2.971805938657	5.677654813781	1.995898053235
C	-2.356965947432	3.890937058700	0.970229882067
H	-3.091533857582	4.033559003648	0.174364126478
C	-1.439552430010	2.484026348503	-1.601710684596
C	-0.964883597120	3.796312729525	-1.709042977823
H	-0.804503746174	4.400148914653	-0.816280635046
C	-0.655296492445	4.349794266416	-2.953709388264
H	-0.280118536530	5.375421160514	-3.002416266979
C	-0.809946248293	3.605343833747	-4.119787836468
H	-0.564432393114	4.040138104233	-5.092018621629
C	-1.274293771993	2.291667231131	-4.027592657983
H	-1.396391078326	1.684210777083	-4.928221701615
C	-1.576545480615	1.743488951744	-2.785876035241
H	-1.919076569978	0.711837032999	-2.728186298969
C	-3.180133695462	1.372971416563	-0.083692142403
C	-3.643126390762	1.010094256189	1.190659996255
H	-2.931550675735	0.888944938701	2.004897743828
C	-4.998411861395	0.857320227747	1.459710002465
H	-5.316927183053	0.576605113968	2.467163963139
C	-5.942340980550	1.084911610403	0.458106428632
H	-7.010376129383	0.982098046907	0.666702138546
C	-5.502431193384	1.452864720555	-0.809580486141
H	-6.224037919721	1.641631955892	-1.608674358415
C	-4.139073701251	1.596731639663	-1.076710492596
H	-3.838024559551	1.916776662646	-2.073454388386
C	-1.714970617662	-1.821871248961	0.019571955198
C	-1.219333795022	-3.295586256415	-0.207257833842
C	-0.516615365118	-3.966255586400	0.809289576982
H	-0.352528125652	-3.479968077908	1.771188642181
C	-0.016870046022	-5.253830304304	0.624203910579

H	0.529260649074	-5.737302813048	1.438120288111
C	-0.217043877445	-5.921031033083	-0.582939663625
H	0.169861067832	-6.932833068223	-0.728303110966
C	-0.931964310007	-5.284572128802	-1.593916375732
H	-1.116303026910	-5.792866539005	-2.543754274463
C	-1.426270394297	-3.992789024645	-1.406652933521
H	-1.993488488948	-3.538315737929	-2.217364927382
C	-2.958223785160	-1.624502301048	-0.880813488097
C	-4.271526831399	-1.787204487423	-0.423709033197
H	-4.475901136506	-1.909362755389	0.636581445792
C	-5.356842495997	-1.777277834635	-1.298435493409
H	-6.363881056465	-1.906541229948	-0.894006969444
C	-5.170425439156	-1.590477629089	-2.665749907095
H	-6.023049381950	-1.583595597753	-3.349387288645
C	-3.876373777882	-1.394718816469	-3.140240905012
H	-3.693285936240	-1.229790400900	-4.205130375710
C	-2.797731641874	-1.415539915961	-2.258391963729
H	-1.799158918566	-1.288781432777	-2.678422566526
C	-2.006028935806	-1.719847107117	1.528219707878
C	-2.903499315093	-2.613424396996	2.137405987793
H	-3.401310713195	-3.378077242916	1.539570794682
C	-3.142068369605	-2.581263724193	3.508531627464
H	-3.855243338913	-3.284947383207	3.945528844577
C	-2.458777269217	-1.677047208903	4.323919275626
H	-2.641119355761	-1.655618353634	5.401298533481
C	-1.521840933740	-0.825402090198	3.746841851591
H	-0.951886895576	-0.125699004122	4.363747436945
C	-1.300286747889	-0.855882623510	2.369010274505
H	-0.558867857109	-0.178010167817	1.951767696456

108

^{BS}**TS(3′b-3″)** (^{1}A, C_1): E_{tot}(UPBE0-D3(BJ)(Acetonitrile)/def2SVP) = -3332.60892474 $\langle S^2 \rangle$ = 0.8832

P	-1.382168077179	1.345338680192	-1.430415964185
P	-0.917590063463	-1.506104740925	-0.215290950259
P	-1.029233106379	1.402468745986	0.683507573256
C	-1.922947946932	0.025177084678	-0.181657515095
C	0.650638041933	-0.679194548384	-0.099517421311
C	0.580467766408	0.737842073307	0.021623454402
C	-3.444906998363	-0.152087220239	0.096137741966
C	-4.194647942425	-0.572677414971	-1.176382635155
C	-3.553287913327	-1.135970504838	-2.283548644980
H	-2.470233183287	-1.274065739339	-2.286886103344
C	-4.278292133942	-1.540354093367	-3.407159104415
H	-3.748337004234	-1.976022208763	-4.258084197169
C	-5.661437356326	-1.386976324462	-3.445083881399
H	-6.228731071112	-1.699027259878	-4.325670721202
C	-6.315741458965	-0.826515168557	-2.346255666097
H	-7.401251841599	-0.698557587086	-2.359286190832
C	-5.589239332732	-0.424473558277	-1.229012585109
H	-6.116077690461	0.012899531894	-0.377559198185
C	-3.582792215886	-1.244312760363	1.178248035619
C	-2.792580549199	-1.179542246943	2.340056093765
H	-2.102795948121	-0.345395387527	2.485416610083
C	-2.868131067428	-2.161028434863	3.324308078078
H	-2.242859601107	-2.077033709276	4.216690279032
C	-3.730700752748	-3.246870668620	3.168806300574
H	-3.787116791538	-4.023006201172	3.936207985813
C	-4.513614996185	-3.330304818941	2.021105906439
H	-5.189776777598	-4.177028207140	1.877220945786
C	-4.440352003829	-2.340090544970	1.038248606062
H	-5.057762791461	-2.441745453085	0.145561085277
C	-4.008961251391	1.204364962431	0.574717995567
C	-4.179679750225	2.235321706218	-0.365052606673
H	-4.012857914250	2.036085823227	-1.426122781064
C	-4.582182498557	3.510685979780	0.024093672745
H	-4.692135943184	4.294095746382	-0.730101325226
C	-4.853728456887	3.781471538502	1.365398264108
H	-5.172733103303	4.780251846565	1.674086180407
C	-4.730938811959	2.759001389512	2.302698334063
H	-4.960194799095	2.946541655664	3.354828060824
C	-4.312504712207	1.485095538012	1.911083135764
H	-4.228410350483	0.706842287014	2.669481439572
C	1.804519804952	-1.738788605922	-0.082237837765
C	1.816277054342	-2.432192029512	1.310549521388
C	0.923369435904	-2.145015483818	2.345735464934
H	0.137378982694	-1.402496353642	2.208815801783
C	1.011974979081	-2.790884512243	3.583301200225

H	0.301187617000	-2.531104408896	4.371857694765
C	1.992899515265	-3.749654262612	3.809936529124
H	2.061752815146	-4.256665842279	4.775687280697
C	2.894388697748	-4.050841587066	2.786045706851
H	3.675398473509	-4.799414776095	2.942480817422
C	2.810714093277	-3.394480109698	1.563281189265
H	3.539871633446	-3.630813802580	0.785365068541
C	1.419600924089	-2.772581441080	-1.176659983231
C	1.244292270296	-4.137774146292	-0.936541883620
H	1.385896937979	-4.546214267682	0.063344221440
C	0.846629725181	-5.004470304957	-1.959487876657
H	0.704912600745	-6.064732584501	-1.734275680118
C	0.620535335228	-4.526485402843	-3.245931091777
H	0.306709908706	-5.205369274122	-4.042921971765
C	0.790469135810	-3.163692575562	-3.502545372745
H	0.612294536770	-2.763603236778	-4.504052544424
C	1.176481270184	-2.304914022854	-2.480058188382
H	1.278780777452	-1.240163849898	-2.690552699329
C	3.271360107304	-1.281680626425	-0.301572608894
C	3.997015264866	-0.758945789283	0.779275914508
H	3.499491002562	-0.598269287155	1.732201058337
C	5.357667103451	-0.484980513231	0.687650041975
H	5.881830941435	-0.078861651953	1.556916684997
C	6.047788226236	-0.749234441902	-0.494518117546
H	7.120120653039	-0.551049137097	-0.569715730785
C	5.348501159480	-1.277934477049	-1.575467783348
H	5.867829575638	-1.499266957803	-2.511502491633
C	3.981043166209	-1.541267617080	-1.479241142369
H	3.480667316383	-1.977822030898	-2.342077662847
C	1.692845510244	1.819900397514	-0.072234201860
C	1.015226667775	3.215700047618	-0.300886748439
C	0.521202485636	3.940766313376	0.797473921278
H	0.666830144600	3.563593044613	1.810855260595
C	-0.168988095850	5.138938717836	0.627488334072
H	-0.550135673775	5.666477044805	1.505513543252
C	-0.371140107110	5.659760596395	-0.649727538733
H	-0.911150684325	6.600084963420	-0.786321683826
C	0.140180496861	4.972046071576	-1.746909098130
H	0.009294858938	5.370060954245	-2.756431926084
C	0.825603334595	3.767967551116	-1.573649113154
H	1.213561940315	3.261577165258	-2.456676145839
C	2.615788087661	1.558637052278	-1.282891536221
C	3.974967470827	1.887505980018	-1.302734638321
H	4.482298709417	2.202063725961	-0.393860356314
C	4.723741798731	1.803201357362	-2.476322475849
H	5.784506410711	2.065361326394	-2.449159171379
C	4.138216164418	1.379106800668	-3.666804541726
H	4.727415765480	1.316881379888	-4.585221361606
C	2.792608800682	1.018458188387	-3.659629544905
H	2.308073358575	0.667149007756	-4.574506812858
C	2.049879611165	1.108522599275	-2.484533987360
H	0.991477979069	0.845434031734	-2.508542885172
C	2.414683052150	1.944058750735	1.283015447014
C	3.360310077570	2.961166238096	1.492810891603
H	3.593193235419	3.664456013843	0.692147418311
C	3.982923113288	3.124516593053	2.727267860973
H	4.724693374688	3.917364078100	2.853700267510
C	3.646078250543	2.300719623326	3.803084630780
H	4.130277131643	2.433057482582	4.773915834854
C	2.666257557939	1.328006443731	3.627771191871
H	2.367484437905	0.685157156828	4.459862827282
C	2.058601603423	1.159962165755	2.382428930487
H	1.300493905311	0.386641649712	2.274041246851

2

Au-Cl ($^1\Sigma^+$, $C_{\infty v}$): E_{tot}(RPBE0-D3(BJ)(Acetonitrile)/def2SVP) = -595.663134220 $\langle S^2 \rangle$ = 0

Au	0.000000000000	0.000000000000	0.403457267165
Cl	0.000000000000	0.000000000000	-1.874889653295

110

ClAu-3 (^{1}A, C_1): E_{tot}(RPBE0-D3(BJ)(Acetonitrile)/def2SVP) = -3928.40925932 $\langle S^2 \rangle$ = 0

P	1.859182472462	-0.340926623222	1.794435209742
P	-0.769288594179	1.467451816105	2.023977853858
P	0.028735909459	0.066911442609	-0.060189170586
C	0.873088006767	1.153671441596	1.168164354024
C	-1.446030167840	0.322063683575	0.973517983531

C	1.137274552661	-1.260343207431	0.573536867411
C	1.780157633981	2.370413020959	0.793941070164
C	2.011178429012	3.240657278806	2.040792946508
C	3.125955063595	1.794985815413	0.303309065482
C	1.106768879525	3.247748643199	-0.279901698495
C	2.406139202638	4.576909086271	1.887118901725
C	3.178187683175	1.020222359565	-0.869454655957
C	-0.147263940522	3.818332048200	-0.017952084431
C	1.903255130179	2.732436240253	3.340531308196
C	4.309117870541	1.947096778363	1.035130237024
C	1.727639985372	3.585645831129	-1.488414009235
C	2.677774544710	5.378464751333	2.992957174813
C	4.363821360194	0.432472823912	-1.296452285409
C	-0.785691250581	4.634194398371	-0.947922841287
C	2.168192962010	3.534888702294	4.451774329542
C	5.501033985571	1.351728755529	0.612261785645
C	1.098556634289	4.414076367054	-2.419343761438
C	2.555251091713	4.861912793648	4.283615224904
C	5.536565155220	0.596193741396	-0.555498856831
C	-0.168989323688	4.928890124503	-2.163634493537
H	2.507212329871	4.997044577899	0.884130479627
H	2.277139129099	0.867163301777	-1.469117617965
H	-0.636476304565	3.658330358104	0.947738929830
H	1.624389334331	1.691108131363	3.517658769678
H	4.310974733486	2.532250905428	1.955467164574
H	2.725429453245	3.212540090491	-1.716851696933
H	2.984321224794	6.416987132459	2.843755344808
H	4.366634838344	-0.164762061050	-2.211564181322
H	-1.764987303175	5.055506307940	-0.709912614779
H	2.071155735579	3.111413302400	5.454551509841
H	6.407271764311	1.487105491775	1.207975260235
H	1.611410894602	4.652162993210	-3.354624420958
H	2.761791439887	5.491388333343	5.152821895988
H	6.468377196846	0.130923205026	-0.886381901218
H	-0.666629583302	5.569554565871	-2.895806081777
C	-2.906908429203	-0.073906740039	0.833781766176
C	-3.740877505098	1.210707103181	1.040634946788
C	-3.150975842424	-0.669490754974	-0.565548906926
C	-3.320874972796	-1.111959164481	1.901268753696
C	-4.793753166228	1.293789946232	1.954592970577
C	-2.483906932499	-1.852532404520	-0.909493484013
C	-2.758411618647	-1.125614447941	3.180592480016
C	-3.440441056770	2.344499008088	0.267654982783
C	-4.030078768528	-0.113077110822	-1.497631254858
C	-4.384398894696	-1.985954378754	1.639075880650
C	-5.525065920394	2.476891149727	2.097926485096
C	-2.671179110699	-2.449965221655	-2.153092134012
C	-3.246956274929	-1.976042272962	4.172819564133
C	-4.173848886284	3.518335221252	0.400989194548
C	-4.215159616505	-0.706482428707	-2.748969419934
C	-4.874512811822	-2.838325738996	2.627084882174
C	-5.220580110007	3.591872001406	1.323712638000
C	-3.533994251969	-1.873032556876	-3.085682873433
C	-4.310015306354	-2.836051726273	3.902334470639
H	-5.055222098827	0.434067943276	2.572447282797
H	-1.820775164396	-2.327248699184	-0.183196121393
H	-1.928383991610	-0.460261993584	3.424197549187
H	-2.620675862546	2.300786888095	-0.454421165322
H	-4.587420084072	0.792540790866	-1.258221151304
H	-4.851647592723	-1.991951651037	0.652631380413
H	-6.339050818095	2.518488142173	2.826305454972
H	-2.137510205130	-3.372963861827	-2.392473607273
H	-2.787052147158	-1.964178922700	5.164250137040
H	-3.927760254004	4.382738901652	-0.220662643937
H	-4.902924966746	-0.246129477943	-3.462884273508
H	-5.707937095203	-3.506568775803	2.395548721192
H	-5.793133227300	4.516130371769	1.435470836693
H	-3.679201858153	-2.336226594768	-4.064768332821
H	-4.692526410033	-3.504148203149	4.678030478325
C	1.463744164762	-2.700309967428	0.188845026838
C	0.251981844921	-3.602507004346	0.459780376768
C	1.846309009626	-2.741385866698	-1.306010851017
C	2.646817908560	-3.154681875994	1.070666498551
C	-0.077733957066	-4.665995580065	-0.386357759244
C	0.896126302515	-2.353159908874	-2.259284757702
C	2.583758379232	-4.284917312990	1.891042035415
C	-0.499433355643	-3.418029176485	1.626418466144

C	3.086352929776	-3.192015175042	-1.770949249552
C	3.845371324830	-2.418571241593	1.056451952995
C	-1.148628525089	-5.508871794386	-0.086629564093
C	1.181145685697	-2.367900150126	-3.621508015821
C	3.671535258010	-4.659365803791	2.683878948379
C	-1.561046471134	-4.265657191619	1.933073564026
C	3.377925730505	-3.212221078609	-3.137184017129
C	4.929555927344	-2.788271211903	1.847640009976
C	-1.897731671188	-5.309490817533	1.071835298443
C	2.433696632799	-2.789046911625	-4.069137213992
C	4.845748750813	-3.911191576306	2.671734475602
H	0.504005020454	-4.843733968877	-1.292260866408
H	-0.102695926323	-2.060531601872	-1.938633387617
H	1.678229772071	-4.891640906997	1.919374678091
H	-0.250887794766	-2.599043517382	2.305018072838
H	3.843827601360	-3.545160124226	-1.071918971557
H	3.945007243276	-1.552719378620	0.398960158311
H	-1.395539380689	-6.327948175572	-0.767043763868
H	0.415090073182	-2.051056997780	-4.333728876551
H	3.592330055597	-5.547292847827	3.316335584827
H	-2.138125869560	-4.096725389011	2.844283933608
H	4.357227681253	-3.566973177204	-3.468737876316
H	5.845805470193	-2.193161751065	1.814324715064
H	-2.740733870129	-5.964938709511	1.304690745314
H	2.665211484796	-2.800057723921	-5.137201735115
H	5.694140636618	-4.203321746383	3.295817194165
Au	-0.357727759638	0.692194139240	-2.190587680801
Cl	-0.863639905942	1.337602358682	-4.372053116211

110

ClAu-3a (^{1}A, C_1): E_{tot}(RPBE0-D3(BJ)(Acetonitrile)/def2SVP) = -3928.39986120 $\langle S^2 \rangle$ = 0

P	-1.272150229299	1.479707505250	-1.303945789156
P	1.378024691311	0.087146388126	-0.317350107586
P	-0.891829602797	0.040483067532	0.938569621985
C	0.102773959486	1.343248714841	0.005121316141
C	0.408653184402	-1.110093413538	0.344242486367
C	-2.124611378203	0.316934147758	-0.429223901449
C	0.505329452773	2.702257676338	0.662073046777
C	1.596351769028	3.378665713448	-0.180957483167
C	-0.757239416257	3.588651710285	0.689615935553
C	1.061787727750	2.431474334292	2.077242362897
C	2.444537575250	4.326402707531	0.407696756595
C	-1.917872870386	3.152450448015	1.354201798893
C	2.297916016215	1.778598882585	2.209965756013
C	1.746179695754	3.120341336323	-1.547868987202
C	-0.823528948632	4.805588896797	0.001993118332
C	0.416568124952	2.833146939653	3.252338290877
C	3.421736147489	4.978667267601	-0.339884344963
C	-3.090535938286	3.900647544471	1.335295626863
C	2.841562321961	1.496027134973	3.460183533452
C	2.726713931370	3.769679625164	-2.300389958840
C	-2.000645692467	5.557691068644	-0.023450162213
C	0.957458745314	2.551128302367	4.508516963003
C	3.572359019224	4.698000017855	-1.698807568942
C	-3.138695346232	5.111465643850	0.641969953573
C	2.166694297384	1.871258590266	4.621532637384
H	2.339859134848	4.557889541053	1.469890655641
H	-1.918976030899	2.205333648218	1.899192614092
H	2.880737409669	1.516046838130	1.323398813809
H	1.093878002593	2.409467820509	-2.059679232924
H	0.049462245853	5.177001169700	-0.535529632475
H	-0.518105331487	3.390343374616	3.206192974450
H	4.072953716372	5.710340127812	0.145158665622
H	-3.973500268280	3.527149183875	1.859896333188
H	3.808170965060	0.991091610061	3.522063666439
H	2.825923040200	3.540299614483	-3.364194193629
H	-2.020197783863	6.501334499168	-0.574510073892
H	0.423339408975	2.877785838843	5.404467366201
H	4.344481602626	5.202775467458	-2.284782379792
H	-4.059875957864	5.698998414068	0.619688297998
H	2.591020459464	1.651672410239	5.604453364385
C	0.736677048961	-2.572831682813	0.578529916892
C	2.235872223537	-2.630160561906	0.952305446386
C	-0.153206305749	-3.113004592481	1.709673735433
C	0.512827392226	-3.423287884967	-0.690424235384
C	3.168612021202	-3.379370594000	0.231144321341
C	-1.542924091921	-3.019222601650	1.564027412603

C	0.715004808325	-2.906379366805	-1.973566325056
C	2.694884594065	-1.862548384510	2.034717014970
C	0.352856108246	-3.740395311874	2.850266361815
C	0.240630586266	-4.791076783851	-0.562884893349
C	4.524052843029	-3.357231249179	0.573788695741
C	-2.405065572744	-3.509355247862	2.541351946944
C	0.663704010048	-3.732543046141	-3.096349967020
C	4.040770455741	-1.847407074933	2.383256689329
C	-0.509391516599	-4.237555930534	3.831150033240
C	0.189408916593	-5.619882402417	-1.682693495294
C	4.966378562163	-2.592517727805	1.648148535767
C	-1.889432248651	-4.118542094476	3.686302231077
C	0.405927747003	-5.095534830158	-2.956647455539
H	2.850527172961	-3.984421529998	-0.618202975684
H	-1.963434863229	-2.585793368901	0.653414315100
H	0.931680550075	-1.846145302352	-2.113707974763
H	1.986345422689	-1.257261683738	2.605520806211
H	1.429218011450	-3.854665463132	2.983816859684
H	0.083600830659	-5.222953559735	0.427384980935
H	5.234904729722	-3.942428120893	-0.015079823823
H	-3.485054622798	-3.418639514950	2.400912694286
H	0.827687777132	-3.301906786791	-4.087420026708
H	4.371739694958	-1.245093034850	3.232673947033
H	-0.090173824830	-4.723374887064	4.716068492795
H	-0.019233606407	-6.685162385415	-1.554571795670
H	6.026313811405	-2.572027613315	1.913607383531
H	-2.561559591975	-4.505542113271	4.456285831772
H	0.367108771651	-5.743810229894	-3.835624208606
C	-3.575375061022	-0.114645350733	-0.617981863894
C	-3.648708984785	-1.622926541438	-0.884284472113
C	-4.381278941797	0.235042906494	0.654269404726
C	-4.123559141674	0.661719906130	-1.834359001057
C	-4.663957136745	-2.421177817441	-0.348011520880
C	-3.946642274880	-0.213579940783	1.909355821887
C	-4.598706493785	0.030428190781	-2.987055130703
C	-2.713502912667	-2.213909918575	-1.741965684424
C	-5.594452147893	0.931185435821	0.600735967029
C	-4.146086769047	2.067928918084	-1.796290735174
C	-4.730937845662	-3.782724360650	-0.646812597637
C	-4.662663241186	0.064662077457	3.070528859088
C	-5.062146322492	0.775232569983	-4.074918463483
C	-2.786412552313	-3.569556301947	-2.050675163361
C	-6.320475246155	1.207837928871	1.761163269990
C	-4.606175828103	2.811616981773	-2.879165181167
C	-3.790805096436	-4.362957378639	-1.496472872095
C	-5.853225387740	0.788891127346	3.004272002231
C	-5.062682006683	2.166719617559	-4.029688136118
H	-5.411968624450	-1.981929555692	0.314039259605
H	-3.042131079249	-0.814958467239	1.996981057441
H	-4.615271812511	-1.058139067898	-3.048741062656
H	-1.918415854464	-1.601876313841	-2.174250278264
H	-5.995760629069	1.262651760316	-0.356843412342
H	-3.820893918869	2.592454436049	-0.895123722697
H	-5.526093939616	-4.391501211883	-0.208671774682
H	-4.287747186159	-0.297652730090	4.031173777369
H	-5.426472580792	0.254147466410	-4.964024815276
H	-2.042636837484	-4.010230584489	-2.717120158486
H	-7.262555720896	1.756740491417	1.683805663361
H	-4.609766348816	3.902974727067	-2.818077067404
H	-3.838777209968	-5.430245506430	-1.727007325188
H	-6.417547396381	1.012323682033	3.913184017216
H	-5.422879013519	2.747533065922	-4.882652406965
Au	3.476939897491	0.147964197158	-1.061485169261
Cl	5.662437239655	0.190066707521	-1.833301229140

110

ClAu-3b (^{1}A, C_1): E_{tot}(RPBE0-D3(BJ)(Acetonitrile)/def2SVP) = -3928.39932752 $\langle S^2 \rangle$ = 0

P	-1.283000895809	-0.021492721321	-0.299539788916
P	1.150353313452	1.858017698965	-1.078108577730
P	0.964021810090	0.194633221487	1.001979269024
C	-0.211614629699	1.396315807099	0.141978799792
C	2.079557850723	0.627983637347	-0.380391541567
C	-0.242292759977	-1.108478259797	0.430669061131
C	-0.936120130245	2.547257695940	0.908076429157
C	-1.649798974808	3.485379048884	-0.076096531985
C	-1.977497762897	1.871083386874	1.827452288574
C	0.120062983117	3.385770924644	1.659726455807

C	-2.168444529548	4.694585342824	0.412279144532
C	-1.576313638509	0.966227442827	2.826323828749
C	0.938139447156	4.248063878458	0.909845164254
C	-1.836793974903	3.186877038797	-1.427197981924
C	-3.354422751443	2.015401374021	1.606627591227
C	0.303280202600	3.364607049933	3.046062652728
C	-2.857204120302	5.569732515644	-0.420311660943
C	-2.505588102968	0.252737789357	3.578707779404
C	1.925171804333	5.022838811452	1.512482406802
C	-2.525614092021	4.065603669353	-2.269134912821
C	-4.288930901591	1.295693384110	2.353805838136
C	1.294143571469	4.139157971379	3.654650008008
C	-3.039610861389	5.257914596856	-1.770293809500
C	-3.871566806509	0.414031923003	3.346725116798
C	2.116946217567	4.964130485112	2.892815369518
H	-2.032944863319	4.949035163428	1.466599992886
H	-0.516550582973	0.791761382112	3.020478027913
H	0.779140273443	4.350716476789	-0.166135160374
H	-1.454168875970	2.260749260324	-1.860540524248
H	-3.715958448203	2.684194841684	0.825797338690
H	-0.339444633948	2.757388414556	3.681132177172
H	-3.254010230895	6.503527086980	-0.013825214611
H	-2.153063617045	-0.443560472702	4.343709694750
H	2.538971232103	5.684015566972	0.896587828230
H	-2.656044921830	3.805424032926	-3.322482841363
H	-5.353696646660	1.428163407494	2.146219859340
H	1.410642900087	4.097747184406	4.740646171973
H	-3.577908050027	5.945330362091	-2.427642526595
H	-4.602754946130	-0.151157382491	3.929968867145
H	2.889793528852	5.571158632308	3.371034631309
C	3.535666417375	0.261391925017	-0.620840948490
C	4.351490633319	1.566697460568	-0.747231099731
C	4.042495061725	-0.563716829693	0.573670493629
C	3.701154213615	-0.558225923921	-1.920587520363
C	5.286096854368	1.774314192751	-1.764998267358
C	3.356105986634	-1.738236295083	0.904962761560
C	2.993950025532	-0.226247322875	-3.081429612963
C	4.202598798692	2.561768711282	0.231369030726
C	5.172581563461	-0.223861116061	1.320963786198
C	4.673403246877	-1.561798297192	-2.006454612785
C	6.046628420153	2.946051089049	-1.810406033395
C	3.768279756671	-2.542417385030	1.963645028397
C	3.254050099221	-0.866429351745	-4.291498785489
C	4.967306626856	3.723423955633	0.194511095176
C	5.589529288139	-1.028086992954	2.385026283840
C	4.933868926655	-2.208059834705	-3.215298762600
C	5.892767324764	3.924011625651	-0.832294693205
C	4.888679016266	-2.185749978163	2.715186051440
C	4.227856543635	-1.862082470094	-4.365583211316
H	5.432871768567	1.019105560473	-2.538151095477
H	2.506426484739	-2.053121182383	0.295408113560
H	2.232493259329	0.556330724002	-3.057643291325
H	3.483067855343	2.420111267753	1.041455508624
H	5.744906084802	0.672056728626	1.077906321604
H	5.251818045671	-1.837336818825	-1.123266199382
H	6.765744303565	3.087273600097	-2.621465205076
H	3.212890270530	-3.454046824661	2.195706425916
H	2.687100054371	-0.583307665939	-5.182048406619
H	4.838965980686	4.477906310660	0.974288865565
H	6.475321995142	-0.741237417270	2.957746179505
H	5.699497430119	-2.987312584058	-3.253226678349
H	6.489671267778	4.838988243289	-0.865846753769
H	5.216733818783	-2.811821722350	3.548744456630
H	4.431368064648	-2.367463522486	-5.313010131343
C	-0.403312361429	-2.611474328941	0.578795238760
C	0.588979005278	-3.313058148500	-0.369548695242
C	-0.216361540239	-3.103333411471	2.028714177329
C	-1.864528775103	-2.935463661856	0.181045810525
C	1.330285261202	-4.435052057600	0.016034300751
C	0.487734517925	-2.403261214650	3.009728978145
C	-2.218892376443	-3.651946909626	-0.962662395217
C	0.735237845935	-2.841391754108	-1.682209597625
C	-0.766370579702	-4.347257509366	2.381470565212
C	-2.889398327710	-2.476685004028	1.024850352876
C	2.189371732585	-5.069087802285	-0.882770086259
C	0.635971742152	-2.918537117895	4.300404565699
C	-3.563285974528	-3.898808048550	-1.260439673042

C	1.573299504752	-3.487706547280	-2.586254405789
C	-0.613412053068	-4.869025461780	3.662174153059
C	-4.225925606977	-2.722721926698	0.732011137869
C	2.310979411048	-4.602853543930	-2.189309456983
C	0.087741202135	-4.152809450976	4.634317884018
C	-4.570295435186	-3.438648343393	-0.417324994924
H	1.252688820869	-4.821793859147	1.032193718635
H	0.939055796449	-1.436180791666	2.791104734198
H	-1.449638418861	-4.033719477131	-1.634663294111
H	0.179812690679	-1.958886323875	-2.007217957139
H	-1.324781266391	-4.920428350214	1.638205569621
H	-2.633340418341	-1.912072979784	1.924122332875
H	2.766316358544	-5.936367851550	-0.551653962817
H	1.189547406005	-2.340297080641	5.044632963944
H	-3.816802459064	-4.461945117772	-2.162255817491
H	1.661877383599	-3.105352400169	-3.604895798219
H	-1.050609249484	-5.841431628426	3.903089387096
H	-5.003168524130	-2.348479431484	1.403229852042
H	2.981447848851	-5.099687382268	-2.894933640806
H	0.202104539205	-4.555788920532	5.643787926386
H	-5.619692291149	-3.630691056682	-0.654213481904
Au	-3.253181867703	0.003469748531	-1.344807457083
Cl	-5.267166300369	0.101869310288	-2.490770318854

110

ClAu-3′ (^{1}A, C_1): E_{tot}(RPBE0-D3(BJ)(Acetonitrile)/def2SVP) = -3928.39806482 $\langle S^2 \rangle$ = 0

P	-0.992745219993	-0.010660402862	-0.034552350770
P	1.655141366161	1.547118828458	0.344659205875
P	1.646098845699	-1.604070032500	0.184167876424
C	-0.098166860837	-1.443219453183	0.215861321325
C	-0.062657416437	1.425127943627	0.224956205438
C	2.345244925476	-0.037202432848	0.082226802543
C	-0.829884167997	-2.814088108926	0.197244170265
C	-0.145575871996	-3.829806264423	1.136760417113
C	0.440480128741	-3.414976405764	2.340761318325
H	0.476864163819	-2.353522554466	2.597651542097
C	0.969358172623	-4.341425568830	3.237986943860
H	1.429581773834	-3.989218174011	4.164645893084
C	0.904335143791	-5.705795991613	2.958441245054
H	1.320297262149	-6.433819828566	3.659378271146
C	0.288458990759	-6.132194320044	1.782160883271
H	0.212129430301	-7.199072739436	1.557230275954
C	-0.236224513683	-5.203681915710	0.884242951691
H	-0.726947341458	-5.559454820339	-0.023686215270
C	-0.763602056471	-3.218263778029	-1.289049192616
C	-1.721657733947	-2.759656505634	-2.201466494047
H	-2.585938496273	-2.192757590673	-1.847537881585
C	-1.582786803968	-2.991577905153	-3.569358914160
H	-2.348284326876	-2.620663235878	-4.255732264984
C	-0.475500496242	-3.683352987663	-4.057715208577
H	-0.364581096561	-3.864687262162	-5.129765549832
C	0.493156090288	-4.134351671015	-3.161748647549
H	1.372556496578	-4.672167997148	-3.525291227236
C	0.351575707238	-3.900776624973	-1.794654290516
H	1.134795345840	-4.248914180635	-1.118733414260
C	-2.253174153192	-2.767122539688	0.782786857984
C	-2.514503521544	-2.006538396277	1.932807232567
H	-1.727844764713	-1.385825182215	2.368236393155
C	-3.767773920888	-2.028480848673	2.539133703969
H	-3.947429020389	-1.415494262350	3.425726603001
C	-4.786274649683	-2.825586271506	2.016405438680
H	-5.773477076221	-2.838248120704	2.484819571771
C	-4.526056843529	-3.616809795904	0.899123440688
H	-5.307206861679	-4.261642739388	0.488547543021
C	-3.268597195729	-3.595959096066	0.294764083990
H	-3.084374328337	-4.235534619300	-0.569751093082
C	-0.844981982884	2.767124827354	0.175625331517
C	-2.037590390072	2.807520908095	1.149207483804
C	-2.211023382889	1.879450658883	2.181651815869
H	-1.506644604683	1.055671931236	2.311491695724
C	-3.270326796022	1.994049698519	3.084305861133
H	-3.381279463267	1.249753018965	3.876833316989
C	-4.171266683120	3.049801063649	2.979558506621
H	-5.005403790944	3.137131544953	3.680198274147
C	-3.981755702248	4.010398721030	1.984121924435
H	-4.662867940770	4.861525456924	1.905017153149
C	-2.919252833736	3.897575570180	1.092155293848

H	-2.768709843239	4.676895266939	0.341782354991
C	0.042341216256	3.939414842141	0.651928896430
C	0.304931812085	5.066944697511	-0.128116869461
H	-0.101239720886	5.144427112257	-1.137142715452
C	1.089122480640	6.112080390405	0.369150896791
H	1.281381878096	6.983887608587	-0.261482988545
C	1.621530583990	6.046240856334	1.653221681912
H	2.240323972531	6.861133962938	2.037298739596
C	1.345015259135	4.933736148671	2.451819209615
H	1.743259956985	4.871560117054	3.467759381699
C	0.554863719946	3.900877083763	1.959803831926
H	0.331593283917	3.047251676520	2.604912942588
C	-1.244401405481	2.903447445748	-1.310154945858
C	-2.568698441913	2.922460507373	-1.757810099085
H	-3.391470614722	2.899376563089	-1.044351625534
C	-2.867260584468	2.959797932092	-3.122733400438
H	-3.913579715862	2.961905298230	-3.438412082503
C	-1.847106977578	2.982972954162	-4.068429539313
H	-2.081322773021	3.008862362950	-5.135631567581
C	-0.518880648222	2.965503561536	-3.636961258295
H	0.297884101958	2.979458232364	-4.363229969351
C	-0.226508735094	2.917015227105	-2.278026597723
H	0.820815482936	2.881538613274	-1.966789427270
C	3.898261758620	0.011317546014	0.010388905361
C	4.508550284813	-1.306599394565	-0.506592749569
C	4.015054724823	-1.863658239553	-1.698205372209
H	3.185990786173	-1.383408490095	-2.224006699928
C	4.574575528478	-3.018019870092	-2.237575458811
H	4.163135307673	-3.435404892504	-3.160135413675
C	5.660724427329	-3.630608732180	-1.609623106455
H	6.103364630173	-4.536450910747	-2.031440111097
C	6.184475440359	-3.064854991480	-0.450045441087
H	7.047394043358	-3.520504282581	0.042369121224
C	5.615219827135	-1.911431232357	0.095066556442
H	6.049327264199	-1.483637598481	0.999734224742
C	4.297003469011	0.350341303700	1.459265890474
C	4.155387399216	-0.627227351916	2.455703569946
H	3.848764278054	-1.641046278205	2.184637322157
C	4.374533665488	-0.325649573772	3.796455350383
H	4.261847812242	-1.109246935607	4.550150565906
C	4.721313528705	0.972289885859	4.177162971930
H	4.886537524653	1.213303022834	5.230291658107
C	4.840707366523	1.958188107159	3.201167857478
H	5.096217938663	2.983183358795	3.481979193491
C	4.629817832012	1.650463675376	1.855420863995
H	4.712535728380	2.446351941650	1.114175891136
C	4.392690869808	1.040614971315	-1.022512583884
C	3.592253072709	1.461045316139	-2.091544860273
H	2.565353383248	1.101533510102	-2.187346651713
C	4.093204973104	2.324698512846	-3.067429166888
H	3.444066814921	2.640408319507	-3.888305157240
C	5.409956074864	2.772802684421	-2.999584491418
H	5.802736162296	3.452164081719	-3.760231553252
C	6.228773702018	2.330999341747	-1.958464377951
H	7.270084980309	2.658361531775	-1.902062437827
C	5.727673908792	1.467621964020	-0.987869892752
H	6.388579991127	1.116001439844	-0.192322134589
Au	-3.190679337056	0.031065697393	-0.507353814122
Cl	-5.467481362031	0.057116015227	-0.974334467696

110

ClAu-3″ (^{1}A, C_1): E_{tot}(RPBE0-D3(BJ)(Acetonitrile)/def2SVP) = -3928.41229216 $\langle S^2 \rangle$ = 0

P	-1.458284790488	-0.853539896112	0.767074604198
P	-0.303206579196	1.032644757692	1.069844076293
P	-0.345080919012	-0.891272147613	-1.121577281678
C	-1.241233384412	0.561052861226	-0.431828866663
C	1.383458775376	0.419769112695	0.539405629379
C	1.352578433788	-0.561006164531	-0.398544172036
C	-2.280176414330	1.461789131959	-1.140011983325
C	-3.538953231165	0.662706460621	-1.510313787422
C	-3.421026306985	-0.554999879468	-2.197153838003
H	-2.436621165096	-0.931237868907	-2.485669685330
C	-4.542694744828	-1.302604589491	-2.545247689441
H	-4.413864627841	-2.252131382123	-3.070934605412
C	-5.820400577253	-0.840547849286	-2.228032785025
H	-6.703121627769	-1.427454748108	-2.493880702111
C	-5.955837194373	0.382138938839	-1.576261543423

H	-6.948879617563	0.767894983261	-1.331783031878
C	-4.827386157651	1.125999637389	-1.224345063977
H	-4.965284151056	2.082259543278	-0.719112707892
C	-1.610098099938	2.015857403045	-2.406634950216
C	-0.262820234205	2.402528338828	-2.374910639906
H	0.315782910401	2.287027146233	-1.456742081380
C	0.356297911429	2.947944944550	-3.496912509626
H	1.408675947527	3.238500127267	-3.443254876399
C	-0.363144979778	3.117881256837	-4.680699113281
H	0.120899213036	3.539930576131	-5.565008157779
C	-1.705542961872	2.746209520889	-4.721932734850
H	-2.282995917337	2.876748103038	-5.640789641863
C	-2.323973921399	2.202991212448	-3.594095836970
H	-3.377645602358	1.922822931542	-3.645378075307
C	-2.619377092728	2.609070328715	-0.170863739397
C	-3.167134424806	2.308688255426	1.088020650218
H	-3.403460885912	1.272448322820	1.346687493297
C	-3.427642618744	3.311044974522	2.016878943422
H	-3.847086937020	3.046665969734	2.990875521681
C	-3.155912409301	4.644850654490	1.705767404517
H	-3.356307348207	5.434043154104	2.434784784511
C	-2.633829360776	4.958294394108	0.454188844021
H	-2.424645629744	5.998230917894	0.190489699902
C	-2.368025511775	3.949062383698	-0.475510762383
H	-1.956142597871	4.221541512381	-1.447875462535
C	2.545187011833	1.091661499974	1.315558521572
C	1.958167082257	2.288242712085	2.125764904675
C	1.471987908276	3.399501177625	1.412747760065
H	1.564613506485	3.426520503666	0.323970692625
C	0.855971127894	4.461311501075	2.065400567591
H	0.469350183669	5.301110284235	1.483051105145
C	0.729974959306	4.450075201461	3.456417991084
H	0.243021331138	5.280564462320	3.973520185445
C	1.242846661778	3.375221019300	4.175957313841
H	1.168208506783	3.359264050548	5.266356337095
C	1.854142425852	2.303222977766	3.517206024349
H	2.237819192925	1.470824617913	4.107444753235
C	3.134857832868	0.053894097566	2.285232629915
C	4.497424719402	-0.095337897636	2.549502756191
H	5.235394333784	0.473620367194	1.987811528576
C	4.948394532464	-0.998557604235	3.513292745458
H	6.022456836988	-1.095793211529	3.690117920270
C	4.048445266562	-1.783524322214	4.228350987715
H	4.405462090705	-2.492933556874	4.979145163976
C	2.684102927424	-1.657932742791	3.965667961228
H	1.957363062013	-2.267361425409	4.508943418925
C	2.238346035229	-0.748388932369	3.010866096757
H	1.162816623615	-0.657995535883	2.839066625677
C	3.552484927696	1.782874972457	0.379748008772
C	4.626925528897	2.497068544183	0.934655447610
H	4.764178217300	2.522870958648	2.017034887034
C	5.510556318753	3.210051478522	0.129188781073
H	6.347717306116	3.741054178999	0.589563221967
C	5.316504707957	3.268909222363	-1.252536783703
H	6.005587058656	3.835048269318	-1.884249419832
C	4.217711809314	2.620228753060	-1.807952657288
H	4.029402761145	2.670550400071	-2.883681439127
C	3.347735072352	1.889908640245	-0.996308572690
H	2.496582394537	1.393142065996	-1.453890495836
C	2.324183584309	-1.633033293299	-0.965383173368
C	2.502847250433	-1.450652974463	-2.485521830761
C	1.901405203910	-0.423938289317	-3.216226696882
H	1.272728461277	0.321732018241	-2.726494586083
C	2.103538003826	-0.296421287380	-4.593244326756
H	1.618127643286	0.523057198030	-5.129336561519
C	2.919799686853	-1.198523162138	-5.266920114621
H	3.082386283240	-1.101437849442	-6.343252301040
C	3.538061271861	-2.225901130531	-4.549090549496
H	4.188151224519	-2.939682505014	-5.061458647086
C	3.336226277229	-2.344305467022	-3.178385370558
H	3.839700926331	-3.144472283049	-2.630825930710
C	1.528389371121	-2.935737895773	-0.664727936306
C	1.129901169559	-3.850604461219	-1.645246924906
H	1.409776405138	-3.699883433280	-2.687591481069
C	0.342674764084	-4.956043175517	-1.315173754138
H	0.039674297059	-5.649697366527	-2.103504949344
C	-0.065899522554	-5.168060104079	-0.001030797123

H	-0.687726067908	-6.030403604598	0.252199253461
C	0.321089959360	-4.262048016822	0.987950536261
H	0.004019858805	-4.404970191828	2.024035222791
C	1.102109450811	-3.160671676416	0.655626970813
H	1.375031317683	-2.445490300155	1.433265729205
C	3.748608099039	-1.732338992007	-0.370290000783
C	4.756233285983	-0.887225887683	-0.858074243353
H	4.510405974047	-0.129327753676	-1.598353257080
C	6.081497530651	-1.021164713606	-0.455446207185
H	6.835512406974	-0.339989053007	-0.858614354569
C	6.445740618103	-2.031911689680	0.433677095172
H	7.487429054653	-2.151604186679	0.742356896975
C	5.463165746366	-2.893821521052	0.911697613705
H	5.725970376343	-3.699740063350	1.601503974026
C	4.133267032445	-2.745422064974	0.515784062691
H	3.401988185829	-3.454662282299	0.899811897628
Au	-3.552536756625	-1.525535616243	1.254171445498
Cl	-5.707285318538	-2.238078589721	1.746576659281

110

ClAu-3″a (^{1}A, C_1): E_{tot}(RPBE0-D3(BJ)(Acetonitrile)/def2SVP) = -3928.42164839 $\langle S^2 \rangle$ = 0

P	-0.986022863122	1.001256480343	-2.253203569233
P	-0.920349488551	-0.396426985830	-0.567983523487
P	0.269322053720	2.108974704506	-0.808706922948
C	-1.349866352719	1.345322662076	-0.416395745883
C	0.864831946146	-0.468501787558	-0.029050459352
C	1.456358719355	0.724065634924	-0.269880203785
C	-2.500864401912	1.962447615543	0.383913720184
C	-2.872247578274	3.388269784450	-0.049703324958
C	-2.381755088171	4.018249844481	-1.194633543738
H	-1.667227885031	3.523325824854	-1.852349642543
C	-2.802353113351	5.304845839346	-1.544994685445
H	-2.398250596450	5.771276761068	-2.446944404214
C	-3.725754254504	5.982906058475	-0.756520989339
H	-4.053874005855	6.988897958626	-1.029904293954
C	-4.234235075525	5.359379221717	0.385972105532
H	-4.966194590797	5.874065116921	1.013722557439
C	-3.815202824083	4.079112313525	0.728846745144
H	-4.227221269623	3.602106063496	1.621526524104
C	-1.942119909293	1.944464954677	1.815828150981
C	-1.847100128351	0.732952776965	2.514059435147
H	-2.250890736637	-0.179106861812	2.069218604185
C	-1.243107696495	0.669466336647	3.767862933068
H	-1.185464163878	-0.287950855753	4.291974939171
C	-0.712969839903	1.820982938559	4.350683975729
H	-0.242195688164	1.774584229692	5.335839130633
C	-0.788128436602	3.028585224922	3.660315965544
H	-0.374078786841	3.939102830102	4.100430024125
C	-1.387941574246	3.088197793187	2.401290226612
H	-1.417246668326	4.041994080259	1.872340650059
C	-3.796985108235	1.151394737072	0.198579131343
C	-4.252988800979	0.901624240085	-1.104633763210
H	-3.666898564736	1.242806570011	-1.960355695746
C	-5.457935255590	0.243022549711	-1.332817710998
H	-5.783982690464	0.057416909425	-2.359352008132
C	-6.244083368646	-0.174905582255	-0.258485115312
H	-7.187834555562	-0.697208803458	-0.433992929835
C	-5.816196983204	0.091366694297	1.039769348134
H	-6.427794493374	-0.212545717123	1.893219557279
C	-4.608301820125	0.754378092404	1.266295487213
H	-4.310060641590	0.967685399580	2.293281419089
C	1.422971713639	-1.854752154215	0.367363305027
C	0.335559571579	-2.841221401639	0.875037203287
C	-0.504724563202	-2.451427210549	1.926949511343
H	-0.447171317764	-1.432395419744	2.309222460439
C	-1.393798422608	-3.345132755251	2.514140583032
H	-2.042742323534	-3.005433705784	3.325447659659
C	-1.448172146198	-4.668707704097	2.072939299420
H	-2.141795731465	-5.377088936030	2.532391253296
C	-0.605326826423	-5.075137440185	1.042029655810
H	-0.629013517155	-6.109252123283	0.688947140123
C	0.279401886560	-4.171192574909	0.448438905181
H	0.936005362796	-4.522482671654	-0.348102733898
C	2.065204551646	-2.388230990426	-0.919656689882
C	3.434738310648	-2.588972198138	-1.086230419809
H	4.122471630232	-2.391803159207	-0.265818561380
C	3.950088380272	-3.024276871508	-2.309979651023

H	5.028462608479	-3.168619638992	-2.412532743905
C	3.107013789631	-3.256666338574	-3.392224640671
H	3.514459840905	-3.592910668845	-4.348944885030
C	1.732835214684	-3.055253655567	-3.241423871462
H	1.052588511205	-3.231368018961	-4.078368478033
C	1.222896687031	-2.628747132249	-2.020325213566
H	0.141182526024	-2.495186727349	-1.922815019585
C	2.305705033802	-1.800008547757	1.624054062964
C	3.129864213028	-2.882922946415	1.958998698236
H	3.237733283426	-3.722889433517	1.270705976156
C	3.795992544905	-2.923026364895	3.181435182355
H	4.446720240929	-3.770734267050	3.410978214844
C	3.618340011242	-1.902366021199	4.117125196296
H	4.134106763441	-1.939898131466	5.079919134103
C	2.759304639521	-0.847582895089	3.815964935266
H	2.590357931890	-0.046948580144	4.540908754591
C	2.114728950126	-0.797958220293	2.581097041821
H	1.449435875742	0.037790491464	2.364976858350
C	2.885179950193	1.302904667832	-0.478733709435
C	3.080630419532	2.537543924027	0.418457328340
C	2.233541359767	2.827627895192	1.491018154209
H	1.365198616736	2.196915175751	1.698043323115
C	2.488789968955	3.911208713665	2.335442963690
H	1.810748828779	4.112593692524	3.167483025489
C	3.600876195391	4.719749086269	2.123106503676
H	3.801501755183	5.567316477132	2.783201586453
C	4.467322149126	4.427916651708	1.066619460469
H	5.353653874387	5.044304564993	0.896201204481
C	4.214335906762	3.343896879552	0.233045997674
H	4.918961880398	3.108210885805	-0.567906582199
C	2.778046711420	1.669914052405	-1.983909803022
C	2.824055164559	2.977824677637	-2.477118143300
H	3.002647283059	3.816251233429	-1.803966154081
C	2.611093354912	3.237505535223	-3.833225049448
H	2.646780078566	4.269445462226	-4.191190993436
C	2.342955008276	2.197704360340	-4.719151704974
H	2.170785828871	2.405385911808	-5.778240898389
C	2.287431236044	0.887567840466	-4.238703730740
H	2.068995195794	0.058127343458	-4.915983369408
C	2.496527466227	0.631598632140	-2.888495663773
H	2.422839616827	-0.393120871114	-2.521839336604
C	4.151825820656	0.453006917565	-0.227933816981
C	4.583616436231	0.238528863237	1.088388764158
H	3.987941073288	0.618280980423	1.915231701836
C	5.778426141819	-0.417150686856	1.366936016944
H	6.074570407469	-0.574420907535	2.407006095790
C	6.603540015913	-0.844739950863	0.325190261554
H	7.548256061658	-1.351440884431	0.538493946854
C	6.220829481213	-0.585196294235	-0.987274768924
H	6.865252467280	-0.882358178265	-1.818579327582
C	5.011091064234	0.056658609485	-1.259123842250
H	4.753160852019	0.247651024442	-2.299416998632
Au	-2.466266330829	-2.029391020758	-0.601212317484
Cl	-4.104519308343	-3.669072478937	-0.759961372555

110

ClAu-3″b (^{1}A, C_1): E_{tot}(RPBE0-D3(BJ)(Acetonitrile)/def2SVP) = -3928.42597235 $\langle S^2 \rangle$ = 0

P	1.006031061155	0.869162703180	-2.467313210005
P	-0.188450226249	2.083581215155	-1.042496631174
P	0.877481385859	-0.481670499117	-0.746838741782
C	1.394483236423	1.242203902075	-0.650875096741
C	-1.407111259084	0.816043447415	-0.337856699897
C	-0.927484885131	-0.444241111173	-0.257518607130
C	2.580604039086	1.889034189565	0.077785664963
C	3.963625007072	1.500913647768	-0.461583363148
C	4.198671754198	0.490623776219	-1.395635538644
H	3.380538856219	-0.090634876969	-1.819124000241
C	5.496739526081	0.183961565504	-1.815107006193
H	5.645743457463	-0.614040322914	-2.546881344722
C	6.585538697017	0.884284712736	-1.306493908881
H	7.600234846493	0.644035482050	-1.633874547809
C	6.365545476943	1.901713896574	-0.374889770497
H	7.208319735265	2.465318216207	0.033544448049
C	5.072045121714	2.205542608990	0.035773043788
H	4.915554814077	3.005038416029	0.764124852877
C	2.412638432879	1.430319331537	1.535583737850
C	1.248889651015	1.775177901175	2.240020712651

H	0.484372456277	2.389668807106	1.761602709458
C	1.050492568202	1.356295862121	3.552575642153
H	0.140792301543	1.652408165313	4.080404838544
C	2.004814255555	0.559039054479	4.186127189632
H	1.852957836993	0.231242029389	5.217502530731
C	3.143152920366	0.172265111270	3.483604850514
H	3.888637358887	-0.472544258617	3.955792432801
C	3.344230837305	0.602217914863	2.170046991803
H	4.244766434008	0.286541989715	1.642101285334
C	2.452191969041	3.413884336316	-0.104382392590
C	2.475519765708	3.920753512569	-1.413777420986
H	2.617578672552	3.238566837104	-2.256391266109
C	2.329924773057	5.282293343204	-1.659938517744
H	2.339531705057	5.649190451981	-2.689423847486
C	2.179247713446	6.174612960220	-0.596482192911
H	2.063081592021	7.244702428883	-0.785851938877
C	2.195965359358	5.687972199474	0.707760392534
H	2.101765056613	6.375945422434	1.551988101042
C	2.333182635452	4.318809110199	0.952587603003
H	2.348567838914	3.965536292338	1.983986348586
C	-2.801383326843	1.430959650660	-0.048551223071
C	-2.695545784626	2.980934766914	-0.196727544419
C	-1.996614617715	3.701676428813	0.787607583975
H	-1.603672041967	3.180082164758	1.663113707849
C	-1.775771362163	5.068579756915	0.661107487724
H	-1.209743374785	5.596784399351	1.432344887385
C	-2.271449659680	5.758822153725	-0.447091357271
H	-2.096689425284	6.832393752180	-0.553113209201
C	-3.002498684010	5.065348336222	-1.406732290093
H	-3.414610094354	5.593221556538	-2.270609835244
C	-3.215288903323	3.688830858762	-1.282426732599
H	-3.782900070177	3.172535505190	-2.056570948574
C	-3.778198195324	0.891247619859	-1.109560888801
C	-5.114898008029	0.575028681199	-0.860751608128
H	-5.508736155084	0.593582249188	0.153105769704
C	-5.970059024765	0.198641770536	-1.897592075984
H	-7.009141550743	-0.048865446554	-1.666393759904
C	-5.506938050741	0.116607370334	-3.207566556736
H	-6.178328339212	-0.182821571266	-4.016253960286
C	-4.169373376167	0.412049871170	-3.471010162599
H	-3.778679936198	0.348610580970	-4.489837121786
C	-3.323279449067	0.798366199266	-2.435488928782
H	-2.286550383421	1.047229195103	-2.675102882174
C	-3.233562077808	1.247100509266	1.416228202972
C	-4.441682332401	1.814837250120	1.853533283827
H	-5.078956754953	2.352502905023	1.149643698271
C	-4.831532229978	1.740178296309	3.187775766528
H	-5.786191202550	2.175934264945	3.493587821715
C	-3.999800834019	1.137357871573	4.133855812370
H	-4.302364742872	1.084331860371	5.182679851045
C	-2.768293973261	0.633794426275	3.726098964158
H	-2.085883725574	0.182490460623	4.451087870018
C	-2.392932125073	0.694485118039	2.382803423227
H	-1.423581515716	0.299152034059	2.092042219354
C	-1.482652745786	-1.880735674056	-0.062796921331
C	-0.929486185775	-2.516784795709	1.226597740361
C	-0.022224832535	-1.879945367307	2.076912811672
H	0.327196276129	-0.866247210606	1.869397416463
C	0.447888841524	-2.511476080299	3.232642599123
H	1.154694709603	-1.984405487371	3.875771779249
C	0.018965892676	-3.792924630762	3.559754163071
H	0.388886822199	-4.285762527893	4.462289925686
C	-0.899244248465	-4.436938005888	2.727795817842
H	-1.256910307677	-5.439989167854	2.973731831769
C	-1.370381329814	-3.801840460398	1.584141224444
H	-2.103430378102	-4.312784899586	0.956306566081
C	-0.940933975552	-2.629503671564	-1.313698855990
C	-0.275316115659	-3.859620868361	-1.263015837602
H	-0.114634315334	-4.362967364449	-0.310617204588
C	0.226041051321	-4.451954010129	-2.423152153558
H	0.752646279477	-5.406619649961	-2.348579406667
C	0.075648065975	-3.828391869962	-3.659921107120
H	0.477356837372	-4.291193628420	-4.564800223898
C	-0.590784528014	-2.605414527769	-3.728260786598
H	-0.721494713351	-2.098713311478	-4.687659664346
C	-1.089709602833	-2.017885182239	-2.568901206508
H	-1.602541994800	-1.056866175569	-2.637866981520

C	-3.023280746693	-2.040890210377	-0.011073497795
C	-3.694679361699	-1.884171841715	1.209897658427
H	-3.139381917493	-1.581966353221	2.094818014566
C	-5.054311317615	-2.154203386425	1.335418121575
H	-5.539944300971	-2.019627949044	2.305540131524
C	-5.781897839460	-2.616491090862	0.239638200303
H	-6.846293720771	-2.845556462796	0.336289545019
C	-5.129210537252	-2.790259618939	-0.977421185883
H	-5.677469000032	-3.156721233957	-1.848861514717
C	-3.769317982530	-2.504213573361	-1.101228536020
H	-3.294508752184	-2.672130139276	-2.066593714710
Au	2.303643272906	-2.140852883294	-0.215870734649
Cl	3.822479674135	-3.790873587923	0.395772780116

9

H3 (^{1}A′, C_s): E_{tot}(RPBE0-D3(BJ)(Acetonitrile)/def2SVP) = -1139.18498063 $\langle S^2 \rangle$ = 0

C	0.594181523388	-0.846604423688	0.000000000000
C	-0.350253707885	0.908425834385	1.428871655927
C	-0.350253707885	0.908425834385	-1.428871655927
P	0.756174632568	1.022320675389	0.000000000000
P	-0.350253707885	-0.767482018519	-1.614758787622
P	-0.350253707885	-0.767482018519	1.614758787622
H	1.450172516661	-1.538163227458	0.000000000000
H	-0.823612707162	1.703165090847	2.013377254336
H	-0.823612707162	1.703165090847	-2.013377254336

9

H3′ ($^1A_1'$, D_{3h}): E_{tot}(RPBE0-D3(BJ)(Acetonitrile)/def2SVP) = -1139.22166189 $\langle S^2 \rangle$ = 0

C	0.000000000000	1.593337751583	0.000000000000
C	1.379870969680	-0.796668875792	0.000000000000
C	-1.379870969680	-0.796668875792	0.000000000000
P	0.000000000000	-1.838708356432	0.000000000000
P	-1.592368146821	0.919354178216	0.000000000000
P	1.592368146821	0.919354178216	0.000000000000
H	0.000000000000	2.693127167111	0.000000000000
H	2.332316542340	-1.346563583556	0.000000000000
H	-2.332316542340	-1.346563583556	0.000000000000

9

H3″ (^{1}A, C_1): E_{tot}(RPBE0-D3(BJ)(Acetonitrile)/def2SVP) = -1139.22424485 $\langle S^2 \rangle$ = 0

C	0.065874698514	-1.110198559943	0.000000045413
C	-0.323369442728	1.223343389527	-0.000000178263
C	1.158991173533	1.144149557155	-0.000000059019
P	-1.110082405270	-0.116725756856	1.072229798796
P	-1.110082428364	-0.116725947899	-1.072229734243
P	1.799205626056	-0.407612638355	0.000000015854
H	-0.052110973746	-2.202241409129	0.000000162158
H	-0.798054244686	2.212316572722	-0.000000278692
H	1.755574756190	2.062123662620	0.000000061648

39

Ph3 (^{1}A, C_1): E_{tot}(RPBE0-D3(BJ)(Acetonitrile)/def2SVP) = -1831.13513613 $\langle S^2 \rangle$ = 0

C	3.178360631511	-0.018034735624	-0.059033335336
C	4.146177443634	-0.223560013236	0.936013709940
H	3.826922758266	-0.402565209398	1.967431026300
C	5.503602830420	-0.199465642420	0.622653144990
H	6.243993505858	-0.361226003935	1.410534363729
C	5.917118372570	0.029963212376	-0.690821859363
H	6.981795985668	0.047700208404	-0.937198178452
C	4.962127504085	0.236613493402	-1.686219114221
H	5.275400889917	0.417520470713	-2.717862122508
C	3.602943272032	0.214264061191	-1.373585053612
H	2.859454053702	0.377520098383	-2.158913108168
C	1.732215451367	-0.026224130477	0.315123380818
P	1.084799189604	-1.637452312921	1.048115246244
C	-1.539961343216	2.116574348935	0.044979710677
C	-2.380283703742	1.970743237083	-1.074952941396
H	-2.065859487624	1.331694016936	-1.905094043292
C	-3.595955721372	2.644643517880	-1.144315204261
H	-4.234796960036	2.520117219202	-2.022230015450
C	-3.996550326459	3.477621752803	-0.098626361344
H	-4.952247045242	4.005002424624	-0.152451297008
C	-3.173150427989	3.631499939786	1.020187991410
H	-3.485682956860	4.276788371373	1.845096386640
C	-1.960084599957	2.957128347657	1.094914990192

H	-1.329435042791	3.062196455289	1.982335509914
C	-0.278078303539	1.406318389674	0.119132880896
P	1.100704508816	1.568529496838	1.104505994597
P	0.297292382893	0.005485578791	-0.896697697629
C	-0.293272004283	-1.421854783765	0.071956734453
C	-1.564406318311	-2.113500594891	-0.022067253357
C	-1.993513990560	-2.980239019041	1.002614084550
C	-2.404726437453	-1.923138687834	-1.135323985776
C	-3.214814088219	-3.637313743890	0.909579590699
C	-3.628618547165	-2.579829935064	-1.222914606927
C	-4.037895607469	-3.439509412356	-0.202502865037
H	-1.363400974737	-3.119696415555	1.885661381162
H	-2.084140767062	-1.262648175446	-1.946101457091
H	-3.534075739557	-4.303318082950	1.715223176109
H	-4.267194374072	-2.420466668613	-2.095378837738
H	-5.000079580433	-3.953257762792	-0.270667760298

39

Ph3′ (^{1}A, C_1): E_{tot}(RPBE0-D3(BJ)(Acetonitrile)/def2SVP) = -1831.14660125 $\langle S^2 \rangle$ = 0

C	0.000000000000	3.115085569734	-0.000000000007
C	-0.810406002834	3.833735972073	0.896581205872
H	-1.428276515831	3.291356547218	1.617416205718
C	-0.809203686887	5.226569939293	0.894557098799
H	-1.439929716203	5.767335148727	1.604748736405
C	0.000000000009	5.928004239209	0.000000000010
H	0.000000000013	7.020931361065	0.000000000017
C	0.809203686898	5.226569939298	-0.894557098789
H	1.439929716216	5.767335148736	-1.604748736390
C	0.810406002836	3.833735972078	-0.896581205879
H	1.428276515829	3.291356547229	-1.617416205733
C	-0.000000000003	1.627956155168	-0.000000000014
P	1.590454096693	0.917483601293	-0.001890642787
C	-2.699218348455	-1.558560957252	-0.000551405352
C	-2.914370171420	-2.626894826356	0.888018720478
H	-2.133439623667	-2.897939246439	1.603531031247
C	-4.121797095556	-3.321205671168	0.884791582804
H	-4.272630886018	-4.143531487806	1.588781536734
C	-5.137074404958	-2.962678528465	-0.002742152071
H	-6.084004995301	-3.508316700042	-0.003577918382
C	-4.936467442179	-1.904059856017	-0.889362689729
H	-5.722793431417	-1.621285533171	-1.593830871823
C	-3.730033786524	-1.208019818714	-0.890358251732
H	-3.571303439657	-0.395948469658	-1.605035898587
C	-1.411070290549	-0.815448758071	0.000405734930
P	-1.590454096699	0.917483601294	0.001890642760
P	-0.000000000003	-1.837158036140	-0.000000000006
C	1.411070290543	-0.815448758072	-0.000405734949
C	2.699218348450	-1.558560957251	0.000551405344
C	3.730033786515	-1.208019818705	0.890358251726
C	2.914370171422	-2.626894826359	-0.888018720479
C	4.936467442171	-1.904059856006	0.889362689735
C	4.121797095559	-3.321205671170	-0.884791582795
C	5.137074404956	-2.962678528462	0.002742152086
H	3.571303439644	-0.395948469643	1.605035898573
H	2.133439623674	-2.897939246448	-1.603531031251
H	5.722793431405	-1.621285533152	1.593830871830
H	4.272630886028	-4.143531487810	-1.588781536720
H	6.084004995299	-3.508316700037	0.003577918406

39

Ph3″ (^{1}A, C_1): E_{tot}(RPBE0-D3(BJ)(Acetonitrile)/def2SVP) = -1831.16823164 $\langle S^2 \rangle$ = 0

P	1.605804291106	0.079649296241	-2.338242104876
P	0.910280045923	1.474838607249	-0.745287928263
P	0.863279425872	-1.437879268396	-0.875692051580
C	1.971990483780	-0.020843597897	-0.509414858437
C	-0.708083988787	0.696443941040	-0.285004429696
C	-0.736610830165	-0.654415500877	-0.367534524262
C	3.280414658634	-0.067776936594	0.178471620536
C	3.460848471176	-0.908805222568	1.284322725060
C	4.344952316150	0.742612328703	-0.240085769144
C	4.681518056115	-0.938604119299	1.957785098599
C	5.560662609224	0.717461165084	0.438446577300
C	5.733794144030	-0.124171977892	1.539482333267
H	2.633708546400	-1.543007146251	1.615694988253
H	4.213673122791	1.392274069531	-1.110556670377
H	4.809549785666	-1.602955844261	2.816367459870

H	6.382616697133	1.354160395210	0.100858285993
H	6.690171087537	-0.147241402017	2.068027862542
C	-1.777784353730	1.588365045117	0.197881807611
C	-2.064367282527	2.783734917518	-0.481170835674
C	-2.508534046062	1.278373779398	1.357309307979
C	-3.076298993171	3.630074851595	-0.031847240098
C	-3.513051126923	2.129577096561	1.807794066878
C	-3.804974014289	3.305044746053	1.112346118601
H	-1.499228640849	3.044620371108	-1.380815654518
H	-2.275942081153	0.365850741961	1.910603907627
H	-3.294522515266	4.550724970636	-0.578999372694
H	-4.070316875451	1.876199548513	2.713378381431
H	-4.595059169710	3.971324526126	1.467873762543
C	-1.893185133470	-1.540393761831	-0.149821289330
C	-3.156623330955	-1.214301544035	-0.673227617767
C	-1.747616397749	-2.747320100342	0.554231122503
C	-4.243138956947	-2.061144920901	-0.477921345266
C	-2.839759339879	-3.589849195650	0.753755729574
C	-4.091060460781	-3.249206297641	0.240633977545
H	-3.278823435669	-0.293085177881	-1.246978170136
H	-0.770985531622	-3.020532912526	0.963763423337
H	-5.217449849600	-1.794615573041	-0.895686614165
H	-2.709931945215	-4.519748723025	1.313225114663
H	-4.946470540549	-3.912225543743	0.392979111729
